# Werner helicase inhibitor for advanced microsatellite instability solid tumors: a phase 1 trial

**DOI:** 10.1038/s41591-026-04657-3

**Published:** 2026-09-29

**Authors:** Timothy A. Yap, Elisa Fontana, Natalie Cook, Marwan Fakih, Oliver Bechter, Erica S. Tsang, Emiliano Calvo, Kristoffer S. Rohrberg, Javier Ros, Ignacio Matos, John H. Strickler, Malaka Ameratunga, Meredith Pelster, Adam Sharp, Jaspreet Grewal, Susanna Ulahannan, Rajesh Belani, Matthew P. Patricelli, Sophia M. Blake, David Dejardin, Stephen Fowler, Christina Godfried Sie, Astrid Heller, Nina Henkel, Michael Hettich, Angelika Lahr, David Lamparter, Charlotte Lane, Ting Liu, Hélène Meistermann, Christophe Meille, Neil Parrott, Sotirios K. Sotiriou, Denise Reeves, Barbara Romagnoli, Piergiorgio Pettazzoni, Daniel Waterkamp, Matthias Wittwer, Yali Fu, Sophie Postel-Vinay

**Affiliations:** 1 University of Texas MD Anderson Cancer Center, Houston, TX USA; 2 Sarah Cannon Research Institute, London, UK; 3 The Christie NHS Foundation Trust and The University of Manchester, Manchester, UK; 4 City of Hope National Medical Center, Duarte, CA USA; 5 Universitair Ziekenhuis (UZ) Leuven, Leuven, Belgium; 6 Princess Margaret Cancer Centre, Toronto, Ontario Canada; 7 START Madrid-CIOCC, Centro Integral Oncológico Clara Campal, Madrid, Spain; 8 Rigshospitalet, Copenhagen, Denmark; 9 Vall d´Hebron Institut d’Oncologia (VHIO), Barcelona, Spain; 10 Clinica Universidad de Navarra, Madrid, Spain; 11 Duke University, Durham, NC USA; 12 The Alfred, Melbourne, Victoria Australia; 13 Sarah Cannon Research Institute, Nashville, TN USA; 14 Royal Marsden Hospital, London, Institute of Cancer Research, London, UK; 15 Norton Cancer Institute, Louisville, KY USA; 16 University of Oklahoma, Norman, OK USA; 17 Vividion Therapeutics, San Diego, CA USA; 18 Roche Pharma Research and Early Development, Roche Innovation Center Munich, Munich, Germany; 19 Roche Pharma Research and Early Development, Roche Innovation Center Basel, Basel, Switzerland; 20 Roche Products Ltd., Welwyn Garden City, UK; 21 Roche Pharma Research and Early Development, Roche Innovation Center New York, New York, NY USA; 22 Gustave Roussy, Villejuif and University College of London Cancer Institute, London, UK

**Keywords:** Colorectal cancer, Targeted therapies

## Abstract

Werner syndrome helicase (WRN) is a synthetic lethal target for microsatellite instability (MSI) cancers. In part 1 of this ongoing first-in-human, phase 1 trial, escalating doses of RO7589831 (VVD-133214), a first-in-class, WRN covalent inhibitor, were evaluated in 88 patients with MSI and/or deficient DNA mismatch repair advanced solid tumors. RO7589831 was administered once daily (150 mg), twice daily (BID; 150–1,000 mg) or three-times daily (TID; 200–600 mg). Primary objectives were to evaluate safety and tolerability, and identify the recommended phase 2 dose and schedule (RP2D). Secondary objectives included evaluation of antitumor activity (end points: disease control rate (DCR), objective response rate (ORR), duration of response (DOR), progression-free survival (PFS) and overall survival (OS)). One dose-limiting toxicity of grade 2 nausea occurred in the 600-mg TID cohort; however, the maximum tolerated dose was not identified per protocol. Treatment-emergent adverse events (TEAEs) occurring in ≥10% were mostly grade 1–2; events in ≥30% were nausea (54.5%, 48/88; grade 3+: 2.3%, 2/88), diarrhea (43.2%, 38/88; grade 3+: 1.1%, 1/88), fatigue (39.8%, 35/88; grade 3+: 2.3%, 2/88), anemia (37.5%, 33/88; grade 3+: 11.4%, 10/88), and vomiting (30.7%, 27/88, grade 3+: 1.1%, 1/88). TEAEs leading to RO7589831 discontinuation were uncommon (3.4%, 3/88). No grade 5 TEAEs occurred. The DCR among MSI efficacy-evaluable patients was 74.2% (49/66). Seven patients achieved confirmed RECIST v.1.1 partial responses (ORR 10.6%, 7/66); median DOR was 10.2+ months. Among MSI efficacy-evaluable patients, median PFS was 6.7 months (95% CI 4.1–8.5); median OS was 17.6 months (95% CI 17.6–not estimable). Although direct target engagement in tumor tissue could not be demonstrated, exploratory analyses of ctDNA molecular response and FDG-PET metabolic response supported biological activity. The 150-mg and 600-mg BID doses were selected as RP2Ds for dose optimization. These data suggest that RO7589831 has a manageable safety profile and warrants further clinical testing. ClinicalTrials.gov identifier: NCT06004245.

## Abstract (web summary)

In the dose-escalation stage of a phase 1 trial evaluating RO7589831, a first-in-class inhibitor of the synthetic lethal target Werner syndrome helicase, in patients with microsatellite instability and/or DNA mismatch repair-deficient solid tumors, the maximum tolerated dose was not reached and some partial responses were observed.

## Main

Microsatellite instability (MSI) is a molecular hallmark in a clinically important subset of solid tumors arising from deficiencies in the DNA mismatch repair (dMMR) pathway^1,2^. MSI/dMMR is a biomarker of response to immune-checkpoint inhibitors (ICIs)^3^ associated with an increase in tumor-infiltrating lymphocytes and high tumor mutational burden (TMB)^4,5^. In recent studies, the incidence of MSI/dMMR was 4–7% among patients with advanced colorectal cancer (CRC)^6,7^ and 15–25% among those with advanced endometrial cancer^8–10^.

ICIs have significantly improved outcomes in patients with MSI/dMMR tumors^11–13^; however, some patients do not respond, whereas others experience disease progression (PD) after initial benefit^11–17^. In addition, some MSI/dMMR tumor types respond less well to ICI therapy^16,17^, which may be complicated by limiting toxicities^11–13^. For patients who progress on ICIs or discontinue prematurely, approved later-line treatment options are limited and may be associated with low response rates and poor tolerability^18,19^. Targeted treatments that directly exploit the DNA damage response defect underlying MSI are also lacking. Collectively, these limitations highlight the unmet need for efficacious and well-tolerated new treatments for MSI/dMMR tumors.

Werner syndrome helicase (WRN), an enzyme belonging to the RecQ family of 3′–5′ DNA helicases, is critically required for DNA replication and repair to ensure genomic stability^20,21^. WRN has been identified as a novel synthetic lethal target for the treatment of MSI tumors, where WRN is essential for unwinding non-canonical DNA structures, such as cruciforms, thereby preventing replication fork collapse^22–26^. In preclinical models, WRN inhibition induces DNA damage and suppresses tumor growth in MSI tumors, while sparing microsatellite-stable (MSS) tumors^23,25,27^.

RO7589831 (VVD-133214) is a small molecule with a first-in-class, covalent mechanism of action (MOA) that selectively and irreversibly binds WRN via the Cys 727 residue. Binding results in potent inhibition of WRN activity, driving selective cytotoxicity in MSI tumors^27^. Herein, we report results from part 1 of a phase 1 study (NCT06004245) that aimed to evaluate the safety of escalating RO7589831 doses as monotherapy in patients with MSI and/or dMMR advanced solid tumors, and identify a recommended phase 2 dose and schedule (RP2D) for dose optimization.

## Results

NCT06004245 is an open-label, multicenter, first-in-human, phase 1 study of RO7589831 in patients with MSI and/or dMMR advanced solid tumors who have progressed on standard therapies, including ICIs where available. Part 1 evaluated escalating RO7589831 doses when given orally in 21-day cycles. The starting RO7589831 dose was 150 mg once daily (QD), with dose-escalation guided by a modified continual reassessment method with an escalation with overdose control. The dosing schedule could be modified in the event of unacceptable toxicity or unfavorable pharmacokinetics. Dosing regimens showing promising safety, pharmacokinetics and antitumor activity/disease control were selected for further evaluation in randomized backfill cohorts. Primary objectives were to evaluate safety, including determination of the maximum tolerated dose (MTD) via assessment of dose-limiting toxicities (DLTs), and identify the RP2D for RO7589831 monotherapy. Secondary objectives were to evaluate pharmacokinetics and antitumor activity (end points: disease control rate (DCR), objective response rate (ORR), duration of response (DOR), progression-free survival (PFS) and overall survival (OS)). Exploratory objectives included evaluation of pharmacodynamic effects and biomarkers associated with tumor response. Part 2 of the study is currently enrolling patients with advanced MSI-high or dMMR CRC and will evaluate two doses of RO7589831 in combination with bevacizumab.

### Patient disposition and characteristics

**Fig. 1:**
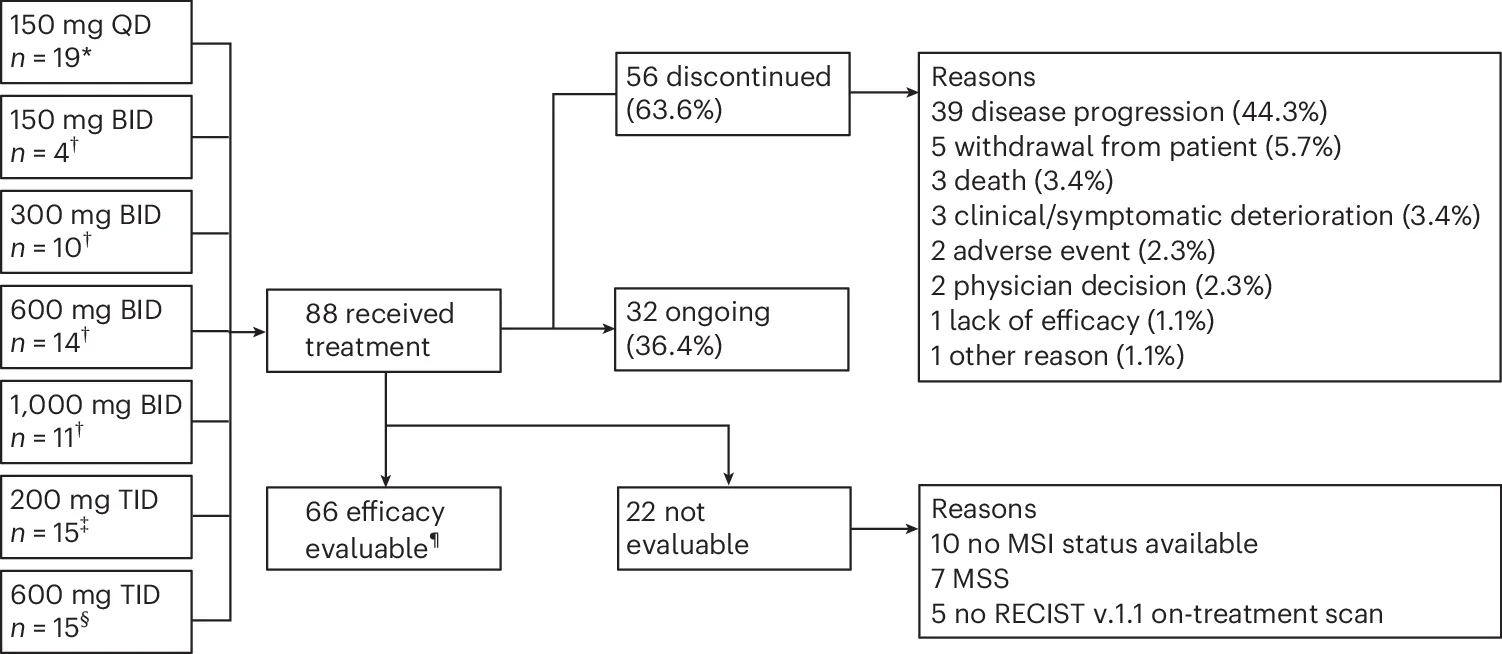
Patient disposition. *Five patients were enrolled during the dose-escalation stage and 14 during the randomized backfilling stage. ^†^All patients were enrolled during the dose-escalation stage. ^‡^All patients were enrolled during the randomized backfilling stage. ^§^One patient was enrolled during the dose-escalation stage and 14 during the randomized backfilling stage. ^¶^Efficacy-evaluable patients were those with MSI tumors and a valid baseline and post-baseline tumor assessment. RECIST, Response Evaluation Criteria in Solid Tumors.

Between 25 January 2024 (first patient in) and 30 June 2025 (last patient in), 88 patients with MSI and/or dMMR advanced solid tumors (determined as part of the routine diagnostic workup in a certified local laboratory) were enrolled at 17 centers in eight countries (Supplementary Table 1). All patients received ≥1 RO7589831 dose. At the cutoff (9 September 2025), 56 patients had discontinued treatment and 32 had ongoing treatment (Fig. 1). The most common reason for discontinuation was PD (*n* = 39 out of 56; 69.6%).

Among all patients, the median age was 60 years (range 27–86), 50 (56.8%) were male and 77 (87.5%) were white (Table 1). Sixty-nine (78.4%) patients had confirmed MSI/dMMR tumors by central laboratory methods (Table 1 and Extended Data Table 1). Overall, 57 (64.8%) patients had advanced CRC, 8 (9.1%) had advanced endometrial cancer and 23 (26.1%) had other tumor types (Table 1). The median number of previous lines of therapy was three (range 1–12). Eighty-four (95.5%) patients had received ≥1 previous ICI (Table 1).

**Table 1:** Baseline characteristics

*n* (%) unless stated	All patients (*N* = 88)
Age, years	
Median (range)	60 (27–86)
≥65 years	35 (39.8)
Sex	
Male	50 (56.8)
Female	38 (43.2)
Race	
White	77 (87.5)
Asian	5 (5.7)
Black or African American	2 (2.3)
Multiple	1 (1.1)
Unknown/not reported	3 (3.4)
Ethnicity	
Not Hispanic or Latino	76 (86.4)
Hispanic or Latino	5 (5.7)
Unknown/not reported	7 (8.0)
ECOG PS	
0	38 (43.2)
1	50 (56.8)
Time since initial diagnosis in years, median (range)	3.2 (0.4–12.8)
Type of cancer	
CRC	57 (64.8)
Non-CRC	31 (35.2)
Endometrial cancer	8 (9.1)
Gastric cancer	4 (4.5)
Prostate cancer	4 (4.5)
Pancreatic cancer	3 (3.4)
Glioma	2 (2.3)
Other^a^	10 (11.4)
MSI and/or dMMR confirmed	69 (78.4)
Number of previous lines of therapy, median (range)	3 (1–12)
Time since last cancer therapy in days, median (range)	55.5 (11 to 1,392)
Best response to previous cancer therapy	
CR	8 (9.1)
PR	24 (27.3)
SD	40 (45.5)
PD	9 (10.2)
Not evaluable/unknown	7 (8.0)
Previous immune-checkpoint inhibitor	84 (95.5)
Best response to previous immune-checkpoint inhibitor	
CR	8 (9.1)
PR	23 (26.1)
SD	38 (43.2)
PD	9 (10.2)
Not evaluable/unknown	6 (6.8)
Reason for discontinuation of previous immune-checkpoint inhibitor	
PD	66 (75.0)
Drug toxicity	12 (13.6)
Completed treatment	8 (9.1)
Physician decision	6 (6.8)
Withdrawal by patient	2 (2.3)
Previous PD-1	84 (95.5)
Previous CTLA-4	20 (22.7)
Baseline sum of diameters of target lesions (mm)^b^, median (range)	80 (15–394)^c^

^a^Including anal cancer, appendix cancer, bladder cancer, breast cancer, carcinoid tumor, esophageal cancer, jejunal adenocarcinoma, ovarian cancer, small intestine cancer and soft tissue sarcoma (all *n* = 1). ^b^By investigators. ^c^There is no correlation between baseline tumor size and body weight. CTLA-4, cytotoxic T-lymphocyte-associated protein 4; ECOG PS, Eastern Cooperative Oncology Group performance status; PD-1, programmed cell death protein 1.

### Dosing regimens and dose-limiting toxicity

In the dose-escalation stage, patients were enrolled into six dose-escalation cohorts: 150 mg QD (*n* = 5), 150 mg twice a day (BID; *n* = 4), 300 mg BID (*n* = 10), 600 mg BID (*n* = 14), 1,000 mg BID (*n* = 11) and 600 mg three times a day (TID; *n* = 1) (Fig. 1 and Extended Data Table 2). After reviewing the dose-escalation data, patients were randomized 1:1:1 (without stratification) into three backfill cohorts: 150 mg QD (*n* = 14), 200 mg TID (*n* = 15) and 600 mg TID (*n* = 14). Median treatment duration among all patients was 115 days (min–max, 2–575) (Extended Data Table 2).

No DLTs occurred in the dose-escalation cohorts. One DLT of grade 2 nausea occurred in the 600 mg TID backfill cohort (*n* = 1 out of 15; 6.7%). This event was considered substantial enough to qualify as a DLT by the investigator as it was intolerable and prolonged. The event resolved after 8 days following treatment with granisetron and RO7589831 dose reduction. The protocol-defined criteria for ending dose escalation were not met (Methods), and subsequent enrollment at this dose level did not confirm this dose as being above the MTD. The MTD of RO7589831 as monotherapy was therefore not identified per protocol.

### Safety

Treatment-emergent adverse events (TEAEs) of any grade occurred in 87 (98.9%) patients (Table 2 and Extended Data Table 3). Grade 3–4 TEAEs occurred in 41 (46.6%) patients and serious TEAEs in 29 (33.0%) patients. No grade 5 TEAEs occurred. TEAEs leading to RO7589831 discontinuation occurred in three (3.4%) patients.

**Table 2:** Adverse event summary

	150 mg QD *n* = 19	150 mg BID *n* = 4	300 mg BID *n* = 10	600 mg BID *n* = 14	1,000 mg BID *n* = 11	200 mg TID *n* = 15	600 mg TID *n* = 15	All patients *N* = 88
TEAE of any grade	18 (94.7)	4 (100)	10 (100)	14 (100)	11 (100)	15 (100)	15 (100)	87 (98.9)
TRAE of any grade	8 (42.1)	3 (75.0)	9 (90.0)	12 (85.7)	11 (100)	12 (80.0)	14 (93.3)	69 (78.4)
Grade 3–4 TEAE	8 (42.1)	1 (25.0)	5 (50.0)	6 (42.9)	7 (63.6)	6 (40.0)	8 (53.3)	41 (46.6)
Grade 3–4 TRAE	0	0	0	2 (14.3)	5 (45.5)	4 (26.7)	3 (20.0)	14 (15.9)
Serious TEAE	6 (31.6)	2 (50.0)	4 (40.0)	5 (35.7)	4 (36.4)	3 (20.0)	5 (33.3)	29 (33.0)
Serious TRAE	0	0	0	0	1 (9.1)	1 (6.7)	1 (6.7)	3 (3.4)
Grade 5 (fatal) TEAE	0^a^	0	0	0	0	0	0	0
TEAE leading to treatment discontinuation	0	0	0	2 (14.3)	1 (9.1)	0	0	3 (3.4)
TRAE leading to treatment discontinuation	0	0	0	1 (7.1)	1 (9.1)	0	0	2 (2.3)
TEAE leading to dose interruption	6 (31.6)	1 (25.0)	5 (50.0)	13 (92.9)	5 (45.5)	3 (20.0)	9 (60.0)	42 (47.7)
TRAE leading to dose interruption	0	0	1 (10.0)	7 (50.0)	4 (36.4)	2 (13.3)	6 (40.0)	20 (22.7)
TEAE leading to dose reduction	0	0	1 (10.0)	5 (35.7)	6 (54.5)	2 (13.3)	7 (46.7)	21 (23.9)
TRAE leading to dose reduction	0	0	1 (10.0)	5 (35.7)	6 (54.5)	2 (13.3)	7 (46.7)	21 (23.9)

^a^A single grade 5 TEAE of renal failure was reported in the 150 mg QD cohort. The event was later confirmed to be due to disease progression and not reportable as an adverse event per protocol.

Treatment-related adverse events (TRAEs) of any grade occurred in 69 (78.4%) patients (Table 2); events in ≥10% were nausea (48.9%), fatigue (28.4%), diarrhea (27.3%), vomiting (25.0%), anemia (19.3%) and decreased appetite (12.5%) (Extended Data Table 4). Grade 3–4 TRAEs occurred in 14 (15.9%) patients; events in ≥3 patients were alanine aminotransferase (ALT) increased (5.7%) and aspartate aminotransferase (AST) increased (3.4%). Two patients experienced grade 4 TRAEs (febrile neutropenia and neutrophil count decreased in one patient and lipase increased in another). Grade 3–4 TRAEs in 12 out of 14 (85.7%) patients required dose interruption or reduction, and events in 13 out of 14 (92.9%) had recovered or were recovering. TRAEs leading to RO7589831 discontinuation occurred in two (2.3%) patients (grade 2 diarrhea in one patient in the 600 mg BID cohort and grade 3 ALT increased and grade 3 AST increased in one in the 1,000-mg BID cohort). TRAEs of any grade leading to dose interruption and dose reduction occurred in 20 (22.7%) and 21 (23.9%) patients, respectively. Gastrointestinal TRAEs were the most common reason for dose interruption (11.4%) and dose reduction (17.0%). Nausea and vomiting (4.5% each) were the most common TRAEs leading to dose interruption. Nausea (12.5%) was the most common TRAE leading to dose reduction.

TRAEs occurred in 42.1% (*n* = 8 out of 19) of patients in the 150-mg QD cohort. In contrast, TRAEs occurred in 75.0–100% of patients in all other cohorts. No TRAEs leading to dose interruption or dose reduction occurred in the 150-mg QD or 150-mg BID cohorts (Table 2). Conversely, TRAEs leading to dose interruption occurred in 50.0%, 36.4% and 40.0% of patients in the 600-mg BID, 1,000-mg BID and 600-mg TID cohorts, whereas TRAEs leading to dose reduction occurred in 35.7%, 54.5% and 46.7%, respectively (Table 2). Gastrointestinal TRAEs leading to dose interruption and/or dose reduction were confined to the 600-mg BID, 1,000-mg BID and 600-mg TID cohorts, with interruptions in 28.6%, 27.3% and 20.0% and reductions in 35.7%, 45.5% and 33.3%, respectively. As noted above, TRAEs leading to RO7589831 discontinuation were confined to the 600-mg BID (*n* = 1) and 1,000-mg BID cohorts (*n* = 1).

### Pharmacokinetics

**Fig. 2:**
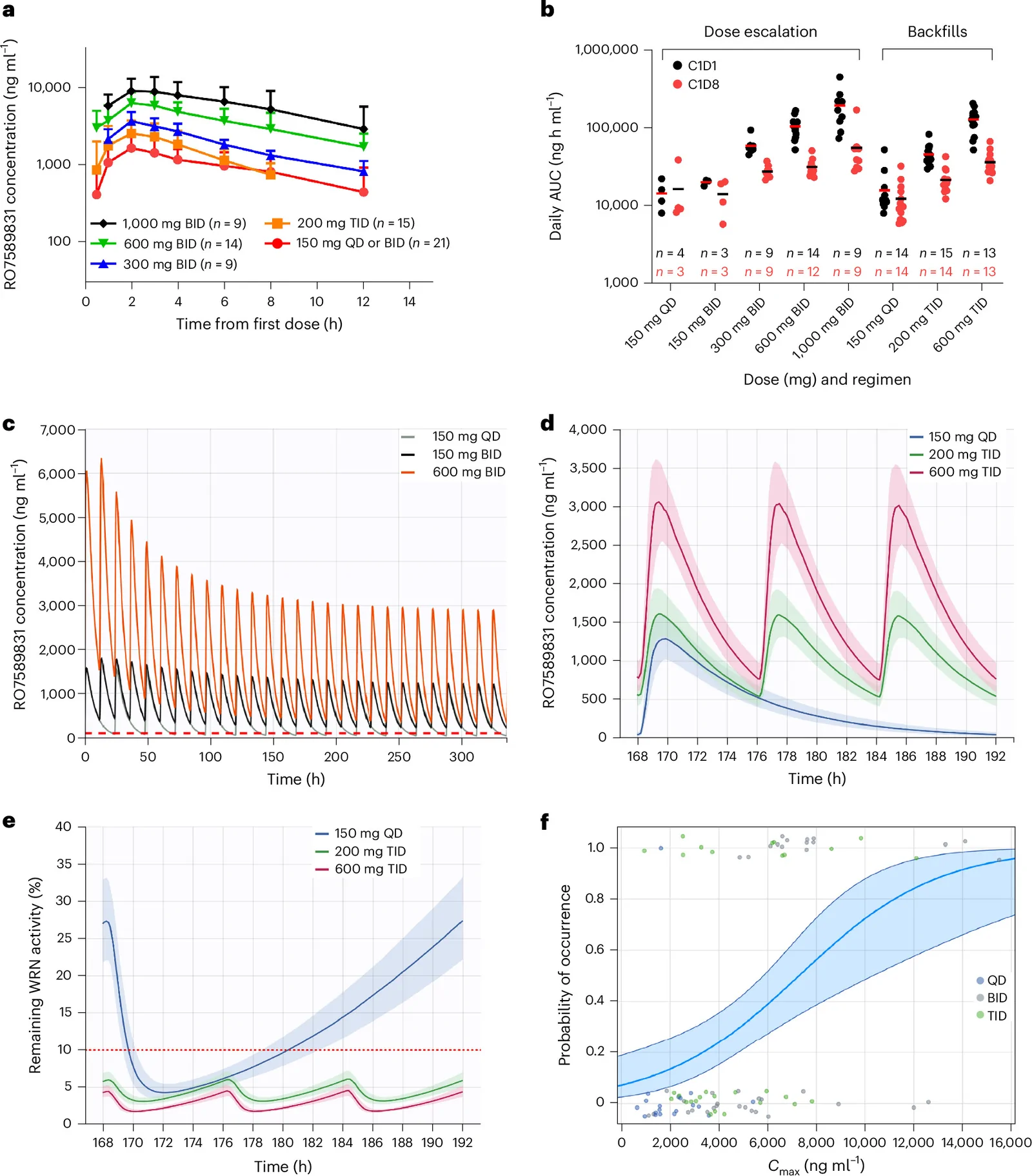
RO7589831 pharmacokinetics. **a**, Mean (±s.d.) pharmacokinetic profiles of RO7589831 following first dose administration. **b**, RO7589831 exposure on C1D1 (black circles) and C1D8 (red circles) by cohort. Individual dots represent individual patients and mean values with lines. **c**, Median pharmacokinetic profiles predicted by population pharmacokinetic modeling over the first 14 days of administration for 150-mg QD (gray), 150-mg BID (black) and 600-mg BID (red) regimens showing the degree of exposure adaptation with time at different doses. **d**, Predicted steady state (C2D1) median pharmacokinetic profiles for 150-mg QD (blue), 200-mg TID (green) and 600-mg TID (red) regimens that were selected for backfill cohorts illustrating the degree of pharmacokinetic differentiation. Colored bands represent the IQR (25th–75th percentiles). **e**, Predicted steady state (C2D1) median target engagement (remaining WRN activity) profiles for 150-mg QD (blue), 200-mg TID (green) and 600-mg TID (red) regimens that were selected for backfill cohorts illustrating the impact of more frequent RO7589831 administration. **f**, Association (predicted probability and 95% CI) between *C*_max_ at C1D1 and grade 2+ gastrointestinal toxicity. Statistical significance was determined by chi-squared test (*P* = 0.0002), indicating a robust relationship between *C*_max_ concentration and gastrointestinal safety events. AUC, area under the curve; IQR, interquartile range.

RO7589831 was rapidly absorbed and eliminated (Fig. 2a). After the first RO7589831 dose, time to maximum plasma concentration was 2.7 h (s.d. ±1.7 h, *n* = 83) and terminal half-life was 4.6 h (s.d. ±3.9 h, *n* = 82). Due to the short half-life, dose escalation was principally performed using BID dosing. There was no indication of a solubility limitation on exposure in the cycle (C) 1 day (D) 1 pharmacokinetic data following single doses of up to 1,000 mg. RO7589831 exposure was higher on C1D1 than on C1D8, especially at higher doses, likely due to dose-dependent metabolism auto-induction (Fig. 2b). Calculated mean (±s.d.) C1D8/C1D1 exposure ratios were 0.90 ± 0.40 for 150 mg QD (*n* = 17), 0.47 ± 0.08 (*n* = 8) for 300 mg BID and 0.38 ± 0.20 for 1,000 mg BID (*n* = 8).

Population pharmacokinetic modeling indicated that steady state was reached after 14 days (Fig. 2c) and that all doses/regimens apart from 150 mg QD resulted in in vivo concentrations of more than the 94 ng ml^−1^ concentration associated with efficacy in in vitro models (not corrected for protein binding). Doses of 150 mg QD, 200 mg TID and 600 mg TID were selected for testing in backfill cohorts based on population pharmacokinetic and pharmacokinetic-target engagement modeling of dose-escalation data, tolerability considerations and minimization of predicted exposure overlap between tested doses. The predicted pharmacokinetic profiles for the selected backfill doses are shown in Fig. 2d to illustrate how these doses were differentiated. Figure 2e shows the corresponding target engagement levels that were predicted using the population pharmacokinetic model, with predicted averages of minimal inactivation (at *C*_trough,ss_) of WRN at C2D1 of 72% (95% prediction interval (PI) 50‒90%) for 150 mg QD, 93% (95% PI 88‒97%) for 200 mg TID and 95% (95% PI 91‒98%) for 600 mg TID.

A significant association between *C*_max_ at C1D1 and gastrointestinal toxicity (grade 2+) was observed (*P* = 0.0002; Fig. 2f). No clear relationship between exposure within the tested dose range and best overall response (BOR) could be established.

### Efficacy

Among MSI efficacy-evaluable patients (*n* = 66), the DCR (proportion of patients with BOR of complete response (CR) or partial response (PR) or stable disease (SD) for ≥6 weeks) after the first RO7589831 dose was 74.2% (95% CI 63.9–82.9). The clinical benefit rate at 3 months (proportion of patients with a BOR of CR or PR, or SD for ≥3 months) after the first RO7589831 dose was 54.5% (95% CI 43.7–65.1), while the clinical benefit rate at 6 months was 33.3% (95% CI 23.8–44.1).

**Fig. 3:**
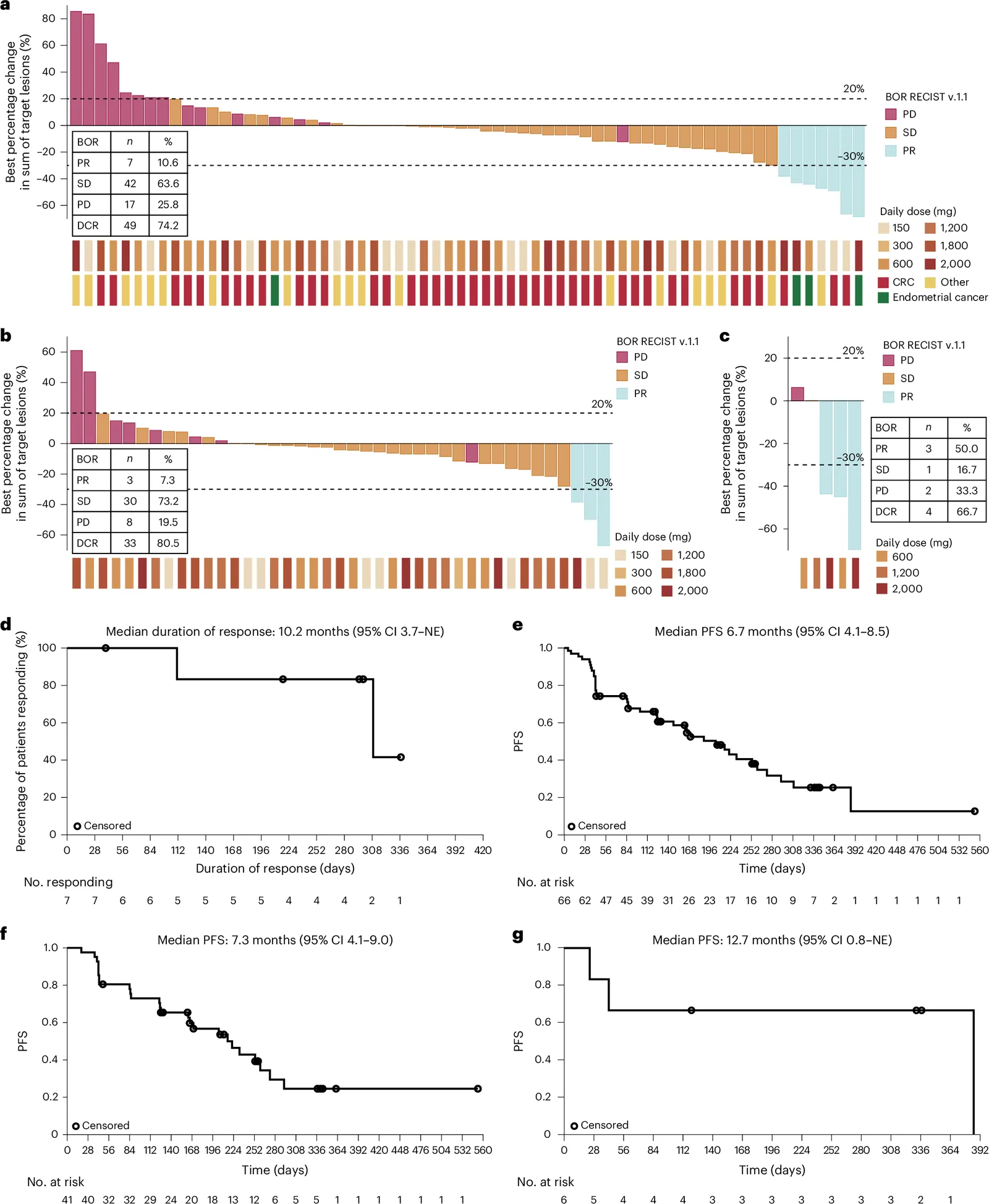
Efficacy by RECISTv1.1 in patients with MSI tumors. **a**–**c**, RECIST v.1.1 waterfall plot showing best percentage change from baseline for all MSI efficacy-evaluable patients (**a**),* efficacy-evaluable patients with MSI CRC (**b**) and efficacy-evaluable patients with MSI endometrial cancer (**c**).^†^ **d**, DOR for the seven responders. **e**–**g**, PFS in all MSI efficacy-evaluable patients (**e**), efficacy-evaluable patients with MSI CRC (**f**) and efficacy-evaluable patients with MSI endometrial cancer (**g**). *Two patients are not shown, as they do not have a valid SLD on treatment. ^†^One patient is not shown, as they do not have a valid SLD on treatment. SLD, sum of target lesion diameters.

Overall, 10.6% of MSI efficacy-evaluable patients (*n* = 7) had a RECIST v.1.1 confirmed PR (cPR), 63.6% (*n* = 42) had RECIST v.1.1 SD (including one patient with an unconfirmed PR), and 25.8% (*n* = 17) had PD as their BOR (Fig. 3a). Of the seven patients who achieved a RECIST v.1.1 cPR, three had MSI CRC (150 mg QD, *n* = 2; 1,000 mg BID, *n* = 1), three had MSI endometrial cancer (300 mg BID, *n* = 1; 1,000 mg BID, *n* = 2) and one had MSI ovarian cancer (150 mg QD). Notably, one patient with MSI CRC who progressed on first-line pembrolizumab after nearly 2 years on treatment and who received 1,000 mg BID RO7589831 as second-line treatment had RECIST v.1.1 SD from week 6–36 and RECIST v.1.1 cPR from week 42–46 despite stopping RO7589831 on C3D10 due to grade 3 AST/ALT increase.

The median number of previous lines of therapy among the seven MSI efficacy-evaluable patients who achieved RECIST v.1.1 cPR was two (range 1–4). All seven had previously received ICI therapy, three with PR as their best response, two with SD and one with PD (for one patient BOR was unknown). Notably, one patient (who achieved a PR) completed ICI treatment, while six discontinued due to toxicity or PD.

The DCR for the 41 efficacy-evaluable patients with MSI CRC was 80.5% (95% CI 67.5–89.9); 3 (7.3%) achieved a RECIST v.1.1 cPR, 30 (73.2%) had RECIST v.1.1 SD and 8 (19.5%) had PD (Fig. 3b). The DCR for the six efficacy-evaluable patients with MSI endometrial cancer was 66.7% (95% CI 27.1–93.7); three (50.0%) achieved a RECIST v.1.1 cPR, one (16.7%) had RECIST v.1.1 SD and two (33.3%) had PD (Fig. 3c).

For the seven patients who achieved a RECIST v.1.1 cPR, the median DOR was 10.2+ months (95% CI 3.7–not estimable (NE); Fig. 3d), with five of seven patients still in response at the cutoff. At 6 months, the Kaplan–Meier estimate of DOR was 83.3% (95% CI 27.3–97.5). DORs for individual patients were 1.3+ months (CRC; 1,000 mg BID), 7.2+ months (CRC; 150 mg QD), 11.1+ months (CRC; 150 mg QD), 9.7+ months (endometrial cancer; 300 mg BID), 9.8+ months (endometrial cancer; 1,000 mg BID), 10.2 months (endometrial cancer; 1,000 mg BID) and 3.6 months (ovarian cancer; 150 mg QD).

**Extended Data Fig. 1:**
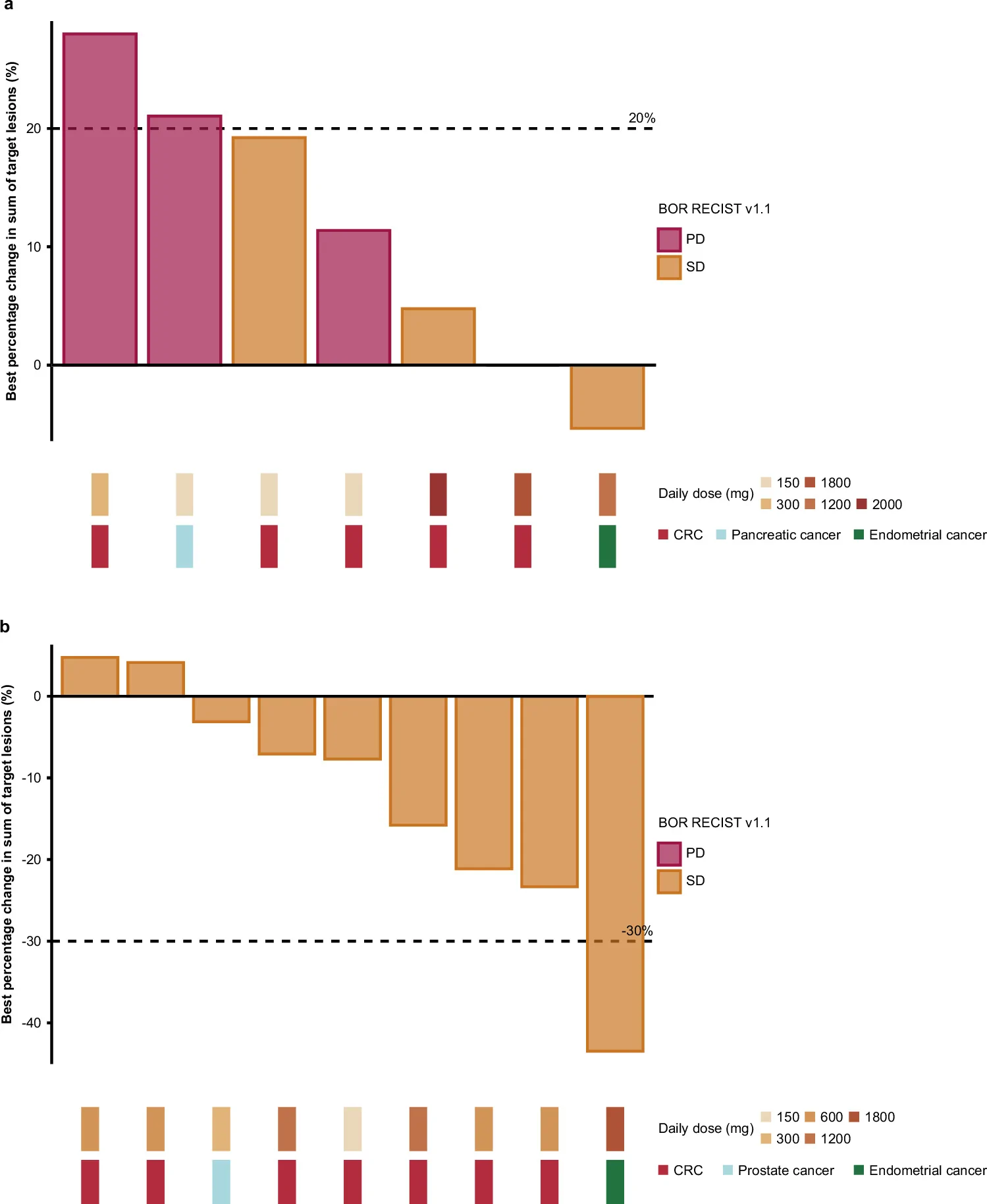
Efficacy by RECISTv1.1 in patients with MSS tumors and in those with unknown MSI status. RECISTv1.1 waterfall plots in (**a**) efficacy-evaluable patients with MSS tumors as determined by central assessment and (**b**) efficacy-evaluable patients with unknown MSI status (neither local or central assessment). Note that one additional patient with unknown MSI status was also not efficacy-evaluable. BOR, best overall response; CRC, colorectal cancer; PD, progressive disease; RECIST, Response Evaluation Criteria in Solid Tumors; SD, stable disease; MSI, microsatellite instability; MSS, microsatellite stable.

Median PFS was 6.7 months (95% CI 4.1–8.5) among MSI efficacy-evaluable patients, 7.3 months (95% CI 4.1–9.0) among those with MSI CRC and 12.7 months (95% CI 0.8–NE) among those with MSI endometrial cancer (Fig. 3e–g). Median OS was 17.6 months (95% CI 17.6–NE) among MSI efficacy-evaluable patients and was NE at cutoff in those with MSI CRC and MSI endometrial cancer; 12-month OS rates in these subgroups were 78.3% (95% CI 55.0–90.5) and 80.0% (95% CI 20.4–96.9), respectively. No RECIST v.1.1 responses were observed in seven efficacy-evaluable patients with MSS tumors per central assessment (Extended Data Fig. 1a) or nine efficacy-evaluable patients with no evaluable MSI status (Extended Data Fig. 1b).

No obvious associations between baseline clinical characteristics and overall response were observed, possibly due to the limited number of patients enrolled and heavily pretreated and heterogeneous nature of the population.

Among MSI efficacy-evaluable patients who had SD as their BOR (*n* = 42), median change from baseline in the sum of target lesions was −4.78% (range −30.6 to 19.4). Among the corresponding subgroup with MSI CRC (*n* = 30), median change was also −4.78% (range −28.2 to 19.4). In the single patient with MSI endometrial cancer who had SD, the sum of target lesions remained unchanged.

Among MSI efficacy-evaluable patients who had SD as their BOR (*n* = 42), 21 patients (50%) had ongoing SD at the data cutoff, while the median duration of SD among the 21 patients (50%) who progressed or died was 6.3 months (95% CI 4.3–7.6). Among the corresponding subgroup with MSI CRC (*n* = 30), 13 patients (43.3%) had ongoing SD, and the median duration of SD among the 17 patients (56.7%) who progressed or died was also 6.3 months (95% CI 4.3–7.6). In the single patient with MSI endometrial cancer who had SD, the duration of SD was 2.8 months.

DCRs among the subgroups of MSI evaluable patients who had and had not responded to previous ICI were comparable (72.7% (16 out of 22) and 75.0% (33 out of 44), respectively). cPR rates were also comparable (13.6% (3 out of 22) and 9.1% (4 out of 44)).

### Exploratory pharmacodynamic analyses

#### Metabolic response

**Fig. 4:**
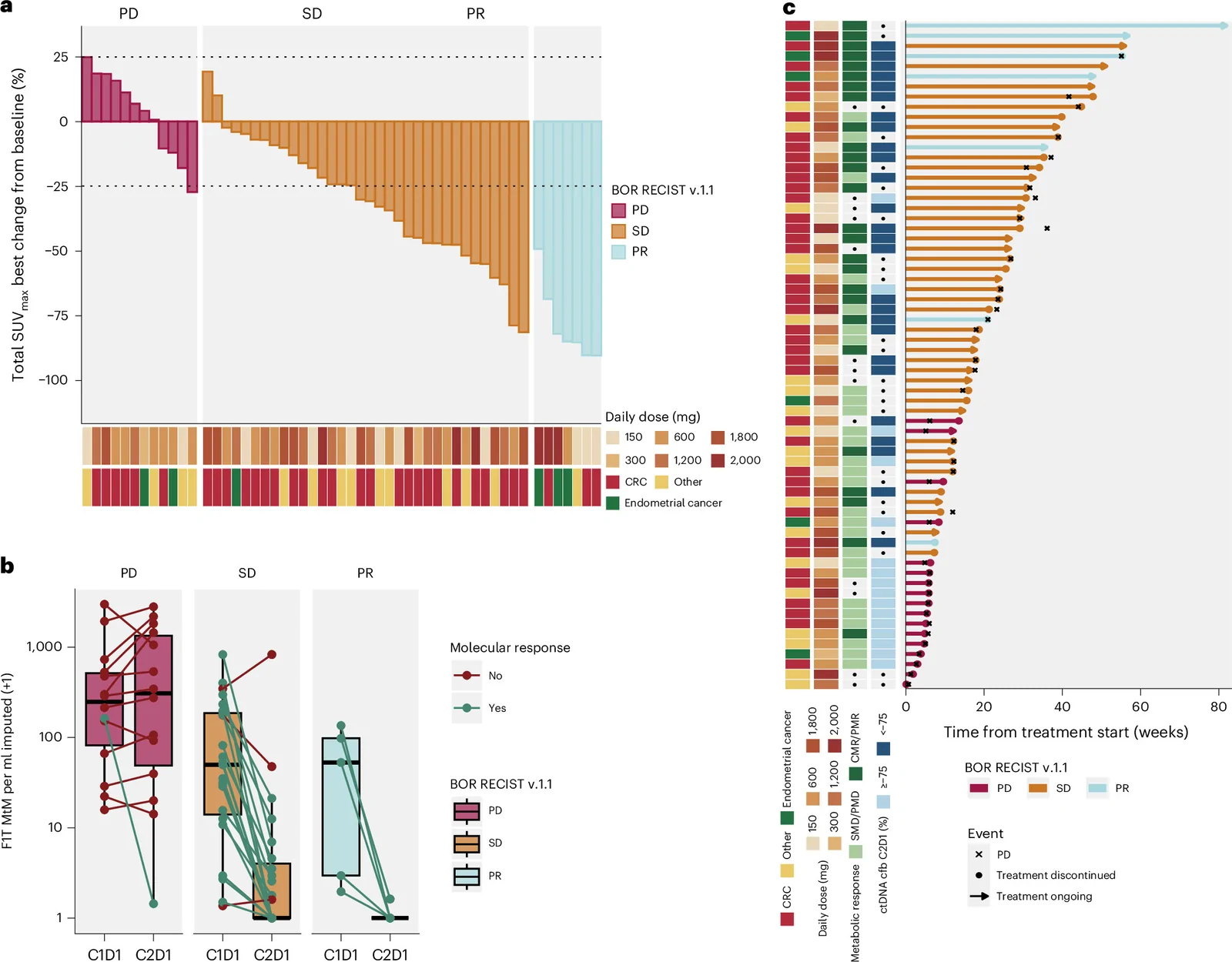
Metabolic and molecular measures of efficacy. **a**, Metabolic response by FDG-PET (best change from baseline) stratified by BOR for efficacy-evaluable patients with confirmed MSI tumors. **b**, ctDNA response stratified by BOR (PD, *n* = 14; SD, *n* = 23; PR, *n* = 5). MTM per ml was imputed from ctDNA tumor fraction (TF) derived from F1LCDx, where applicable (Methods), shown on a log_2_(*x*+1) scale. Green lines indicate the reduction of ctDNA on C2D1 by ≥75% (molecular responders); red lines show the ctDNA reduction of less than 75% by C2D1. Box plots show the median (center line); boxes represent the IQR (25th–75th percentiles); whiskers extend to the most extreme data points within 1.5 × IQR; points beyond the whiskers are plotted individually as outliers. **c**, Treatment duration for efficacy-evaluable patients with confirmed MSI tumors. cfb, change from baseline; F1LCDx, FoundationOne Liquid CDx; F1T, FoundationOne Tracker; SUV_max_, maximum standardized uptake value.

Metabolic responses assessed with (^18^F)fluoro-2-deoxy-D-glucose positron emission tomography (FDG-PET) were available for 53 MSI efficacy-evaluable patients. Reduced metabolic activity was observed in 81.1% (*n* = 43) of patients across all dose levels, with 49.1% (*n* = 26) achieving a partial metabolic response (PMR) or better (reduction ≥25%); 47.2% (*n* = 25) achieved a PMR and 1.9% (*n* = 1) achieved a complete metabolic response (CMR). The greatest reductions in metabolic activity were associated with RECIST v.1.1 PRs. There was no clear association between metabolic response and RO7589831 dose or cancer type (Fig. 4a).

#### Circulating tumor DNA molecular response

**Extended Data Fig. 2:**
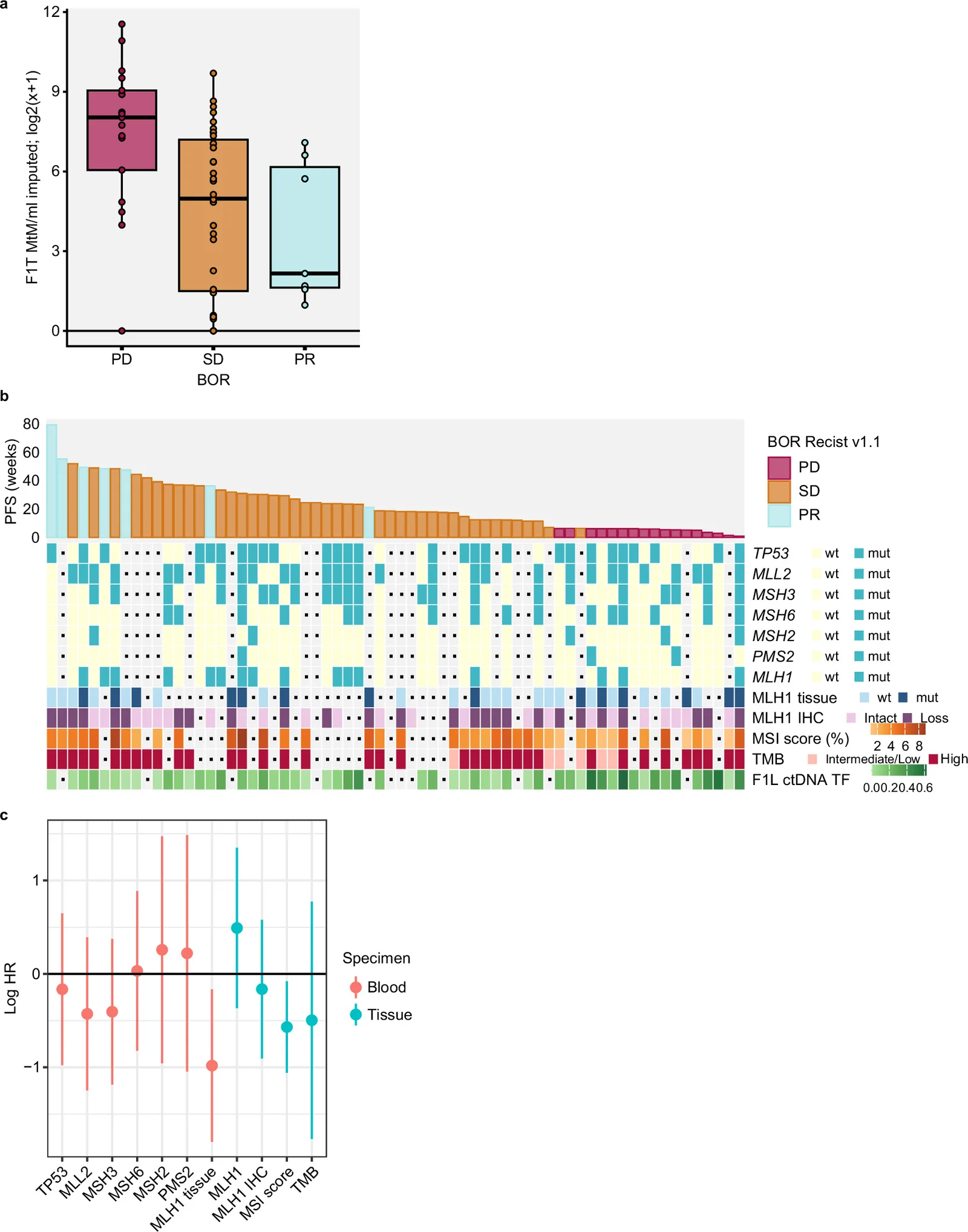
Assessment of exploratory predictive biomarkers. (**a**) Baseline ctDNA levels by BOR (PD, n = 17; SD, n = 35; PR, n = 7). Box plots show the median (center line); boxes represent the IQR (25th–75th percentiles); whiskers extend to the most extreme data points within 1.5 times the IQR; points beyond the whiskers are plotted individually as outliers. (**b**) Baseline biomarkers with respect to outcome (PFS, BOR) for efficacy-evaluable patients. Mutation patterns of *TP53* and the primary genes associated with dMMR based on NGS variant profiling on ctDNA for samples with ctDNA TF > 0.01 are shown (rows 1–7). Further, of tissue derived biomarkers, MLH1 variants based on NGS, MLH1 calls based on IHC, MSI score, and TMB based on NGS are shown. Black dots indicate data not available. (**c**) Shown are the effect size estimates for Cox regression association between PFS and the biomarkers highlighted in **b** within efficacy-evaluable patients (liquid derived markers, n = 44; MLH1 tissue, n = 34; MLH1 IHC, n = 49; MSI score, n = 41; TMB, n = 43). Tissue based biomarkers were fitted in a univariate model whereas NGS ctDNA based biomarkers were modeled bivariately controlling for ctDNA levels and removing samples with ctDNA TF < 0.01. MSI score was standardized prior to fitting such that one unit change corresponded to a shift of one standard deviation. Points indicate the estimated regression coefficients (log HR). Error bars represent 95% CIs. BOR, best overall response; CI, confidence interval; ctDNA, circulating tumor DNA; dMMR, deficient DNA mismatch repair; HR, hazard ratio; IHC, immunohistochemistry; IQR, interquartile range; MMR, mismatch repair; MSI, microsatellite instability; MTM, mean tumor molecules; mut, mutant; NGS, next-generation sequencing; PD, progressive disease; PFS, progression-free survival; PR, partial response; RECIST, Response Evaluation Criteria in Solid Tumors; SD, stable disease; TF, tumor fraction; TMB, tumor mutational burden; wt, wild type.

**Extended Data Fig. 3:**
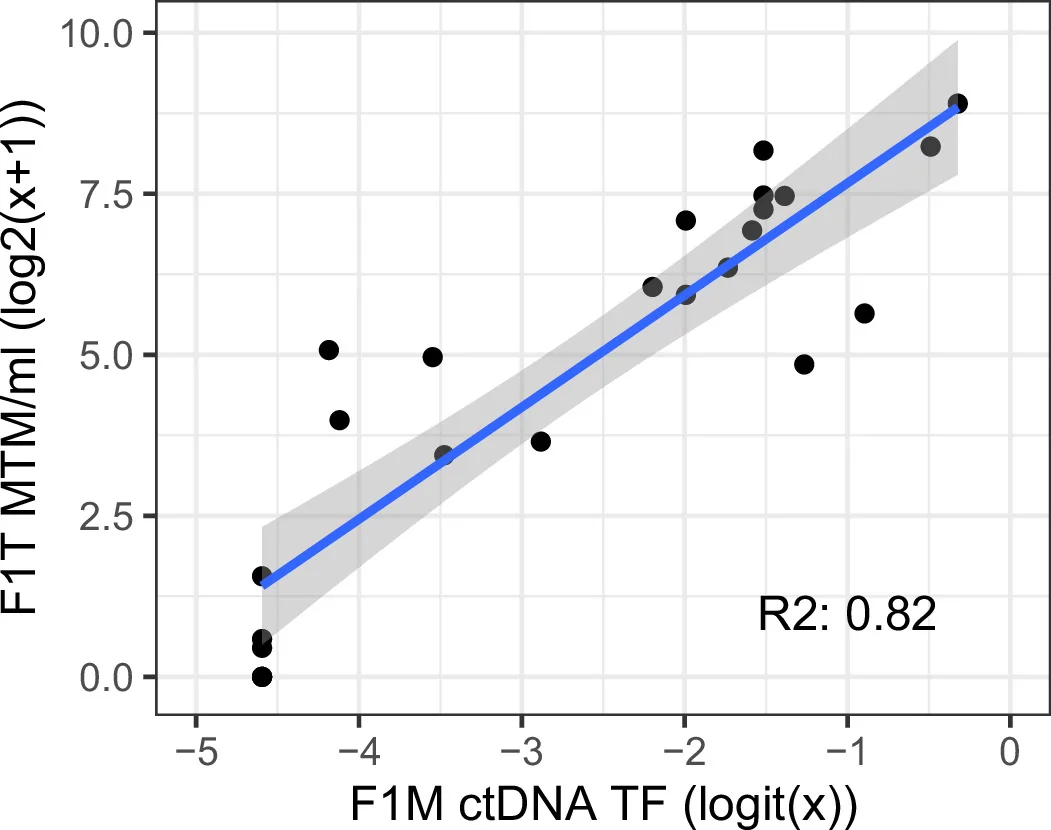
Comparison of F1T and F1M ctDNA assay. Transformed baseline measurements for F1T and F1M, respectively. This linear regression model was used to convert ctDNA TF to imputed MTM/ml using 22 samples. To accommodate zero measurements of ctDNA TF, measurements were capped at 0.01 from below. The solid line represents the fitted linear regression, and the shaded band indicates the pointwise 95% CI around the estimated mean response. CI, confidence interval; ctDNA, circulating tumor DNA; MTM, mean tumor molecules; TF, tumor fraction.

Baseline circulating tumor DNA (ctDNA) values were obtained for 59 efficacy-evaluable patients and were significantly associated with disease control (logistic regression of baseline log-mean tumor molecules (MTM) per ml versus PD; odds ratio (OR) 0.72 per unit increase; 95% CI 0.54–0.91; *P* = 0.012; Extended Data Fig. 2a). A multivariable regression model was fitted including both baseline and C2D1 ctDNA from paired samples (*n* = 41; Extended Data Table 5). After adjusting for baseline ctDNA, C2D1 ctDNA remained associated with disease control (OR 0.52 per unit increase; 95% CI 0.32–0.72; *P* = 0.001). Finally, molecular response, defined as a reduction of ≥75% in ctDNA levels by C2D1, measured as imputed MTM per ml of patients with C1D1 levels above the lower limit of quantification (LLoQ) (*n* = 42; Methods and Extended Data Fig. 3), was associated with RECIST v.1.1 disease control (OR 108.3; 95% CI 14.7–2386.9; *P* = 0.0001; Fig. 4b). All patients who achieved a BOR of RECIST v.1.1 PR and had available baseline and C2D1 ctDNA (*n* = 5) samples, and 25 out of 28 patients with radiological disease control (RECIST v.1.1 PR or SD), achieved molecular responses. In contrast, 12 out of 13 patients with a BOR of RECIST v.1.1 PD did not achieve deep ctDNA reductions, but instead had a median increase of +28% (first quartile (Q1) −0.25%; third quartile (Q3) +57%) in ctDNA compared to their baseline level (Fig. 4b).

### Reverse translational studies

To explore the association between clinical response (PFS) and translational parameters, including MSI score, TMB, TP53, MLH1 and MMR status, retrospective Cox regression analyses based on next-generation sequencing (NGS) and immunohistochemistry (IHC) tissue profiling were performed using baseline samples (Extended Data Fig. 2b). There was a nominal association between the MSI score at baseline and PFS (per 1 × s.d. increase, hazard ratio (HR) 0.56; 95% CI 0.35–0.93; *P* = 0.02) (Extended Data Fig. 2c). As preclinical reports have suggested that TA repeat expansions may confer sensitivity to WRN inhibition^26,28^, we assessed whether the nature of the reported unstable microsatellites could further strengthen this association. Unfortunately, the Foundation Medicine assay architecture precluded assessment of large-scale expansions and the remaining features did not refine the MSI score association. We also observed a nominal association between *MLH1* mutation status derived from baseline ctDNA and PFS (HR 0.38; 95% CI 0.166–0.83; *P* = 0.019). This association was not observed when analyzing tissue MLH1 status by IHC or variant calling, however. In addition, neither *TP53* mutation status nor TMB analysis revealed a clear association with RECIST v.1.1 responses (Extended Data Fig. 2c). Taken together, this suggests that beyond the predictive value of the microsatellite status, the current study was underpowered to establish tumor intrinsic biomarkers predictive of outcome.

**Extended Data Fig. 4:**
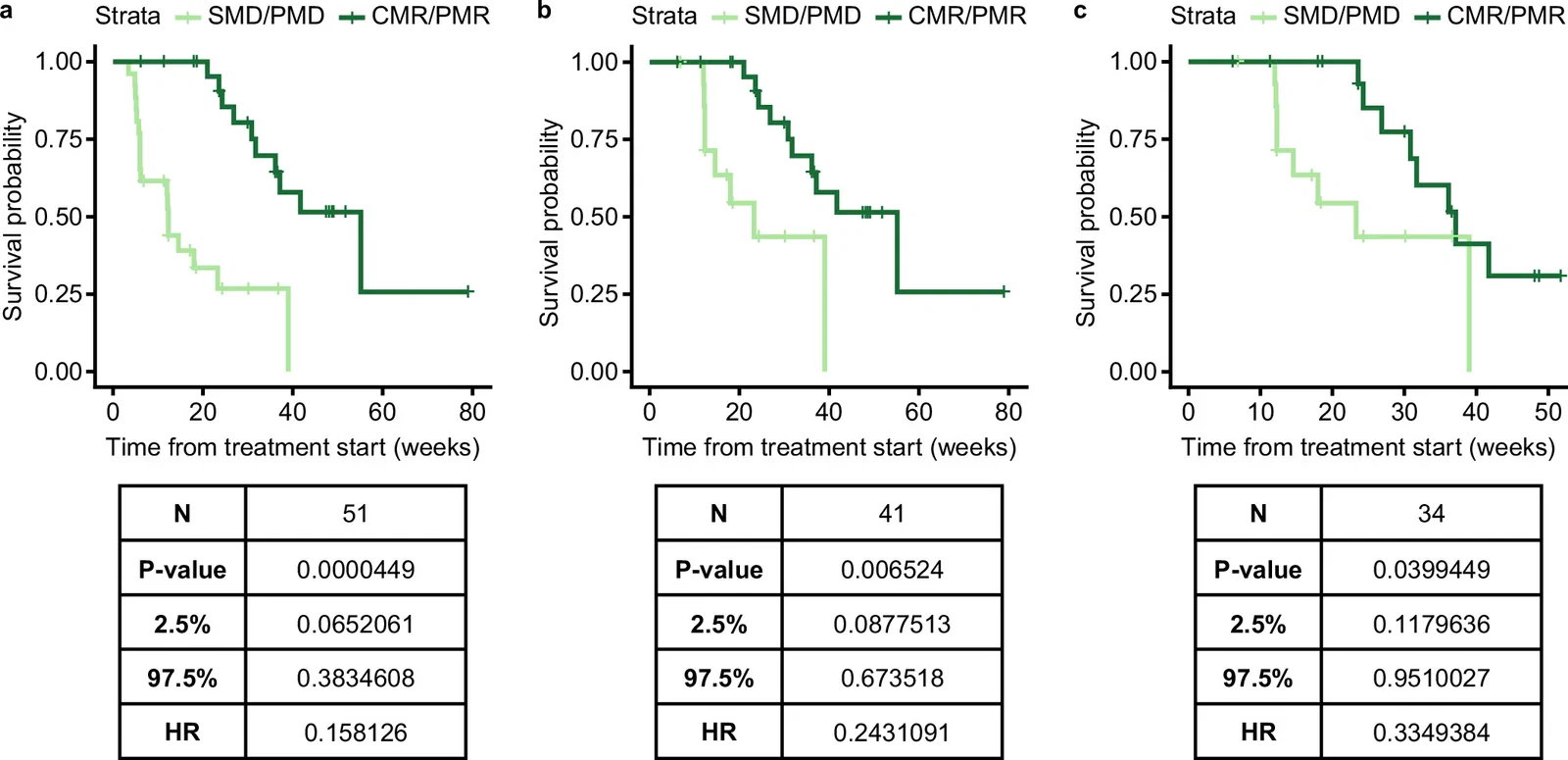
Comparison of time to progression and metabolic response. Kaplan–Meier curves stratified by metabolic response in (**a**) efficacy-evaluable patients, (**b**) patients with disease control (for example PR or SD), and (**c**) patients with SD according to RECISTv1.1. CI, confidence interval; CMR, complete metabolic response; HR, hazard ratio; PMD, progressive metabolic disease; PMR, partial metabolic response; PR, partial response; RECIST, Response Evaluation Criteria in Solid Tumors; SD, stable disease; SMD, stable metabolic disease; 2.5%, 95% CI lower bound; 97.5%, 95% CI upper bound.

For the 62 patients with RECIST v.1.1 assessments available after their first restaging assessment at week 6, there was a trend for the association of FDG-PET metabolic responses with longer treatment duration (median of 36 and 12 weeks for PMR and stable molecular disease (SMD) versus progressive molecular disease (PMD), respectively; Fig. 4c). In a retrospective Cox proportional hazards regression analysis evaluating PFS across all efficacy-evaluable patients with RECIST v.1.1 scans at week 6 on trial, metabolic response (CMR/PMR versus SMD/PMD) was associated with a HR of 0.16 (95% CI 0.07‒0.38). When restricting the analysis to patients with PR and SD according to RECIST v.1.1, the HR was 0.24 (95% CI 0.09‒0.67). When further restricting the analysis to patients with SD, the HR was 0.33 (95% CI 0.12‒0.95) (Extended Data Fig. 4a‒c). There was no clear association between duration of treatment and indication or dose (Fig. 4c).

WRN target engagement by RO7589831 was evaluated in paired baseline and on-treatment fresh tumor biopsies. Only five (19.2%) of the 26 biopsy pairs collected contained sufficient tumor cell content for evaluation (Extended Data Table 6), which was insufficient for a conclusive analysis and was likely due to the high prevalence of mucinous histology in the tumor samples (Supplementary Fig. 1). Notably, a high percentage of sample analysis failed due to signal interference or lack of signal detection in both baseline and on-treatment biopsy samples (Supplementary Table 2). This was unforeseen, as WRN had been readily detected in fresh-frozen human tissue resections (Supplementary Table 3) and consistent WRN target engagement by RO7589831 had been observed in ex vivo tumor lysates derived from an HCT-116 xenograft mouse model using a liquid chromatography–tandem mass spectrometry (LC–MS/MS) assay (Supplementary Fig. 2).

## Discussion

RO7589831 is a first-in-class, covalent WRN inhibitor in clinical development for the treatment of advanced MSI and/or dMMR solid tumors. In the current study, RO7589831 monotherapy showed evidence of biological activity and disease control in a heavily pretreated population with advanced MSI solid tumors, 95% of whom had received previous ICIs. Disease control was achieved in 74.2% of patients, with 10.6% achieving a RECIST v.1.1 cPR and 63.6% RECIST v.1.1 SD. Clinical benefit was observed regardless of the underlying histology, suggesting that WRN inhibition affects the biology of MSI/dMMR-driven tumors in a tumor-agnostic fashion.

Although direct target engagement could not be demonstrated due to an overwhelming presence of mucins in MSI tumors, pharmacodynamic metabolic changes and ctDNA molecular responses were observed in most patients. Overall, these data are supportive of an early clinical proof of concept for a synthetic lethal strategy in MSI tumors by effectively drugging WRN, although definitive confirmation of direct pharmacological inhibition within the tumor microenvironment is pending.

Despite one patient in the 600-mg TID backfill cohort experiencing a DLT of grade 2 nausea, the MTD of RO7589831 was not identified. Overall, the incidence of grade 3–4 TRAEs was low and TRAEs leading to RO7589831 discontinuation were infrequent. No grade 5 events occurred. TRAEs were managed with dose interruptions, dose reductions and supportive medications as required. Collectively, these data indicate that RO7589831 has a manageable safety profile. A similar safety profile was recently reported for HRO761, a reversible WRN inhibitor that binds a similar pocket on WRN as RO7589831 and is currently in phase 1 development in patients with MSI-H/dMMR advanced solid tumors^29^. Modest ORRs (8.6% overall and 10.5% in patients with CRC) and high DCRs (68.6% overall and 84.2% in patients with CRC) were also reported, consistent with those observed in the current study.

While tolerability was closely monitored via clinical assessment in the current study, patient-reported outcomes (PROs) were not evaluated in part 1. Given the potential clinical significance of chronic low-grade gastrointestinal toxicity with long-term dosing, incorporating formal PRO assessments may be an important component of future trials evaluating WRN inhibitors.

The 1,000-mg BID schedule was not considered for further development due to tolerability issues leading to dose interruption in 5 of 11 patients and dose reduction in 6 of 11 patients. The 600-mg TID regimen provided a similar cumulative daily RO7589831 dose to the 1,000-mg BID regimen, but a reduced *C*_max_ (high *C*_max_ correlated with gastrointestinal adverse events) and increased *C*_trough_ (resulting in improved predicted target engagement). Despite a reduction in acute nausea, tolerability remained suboptimal, leading to dose interruption in 9 of 15 patients and dose reduction in 7 of 15 patients. In addition, one patient experienced a DLT (grade 2 nausea) and three patients experienced grade 3 TRAEs. TID schedules were examined extensively in the backfill cohorts, with the aim of maximizing WRN inhibition and improving efficacy; however, there was no noticeable improvement in efficacy, and RO7589831 exposure was not superior to the equivalent daily doses in a BID schedule. Furthermore, the TID schedule increased the pill administration and compliance burden on the patients in the study. Therefore, TID schedules were not proposed for further clinical development. The flat dose-response profile created a range of viable RP2Ds. The 150-mg and 600-mg BID doses were selected as the recommended doses for formal clinical dose optimization per Project Optimus, enabling further exploration of doses at the low and high known efficacious exposure range using the same administration regimen with predicted continual WRN activity suppression^30^. Both doses are clinically active, well tolerated, differentiated in pharmacokinetic exposures, provide *C*_trough_ concentrations above the predicted threshold for activity and achieve a predicted median target engagement at steady state below or equal to 10% remaining activity.

RO7589831 showed dose-proportional exposures after the first dose and reached steady state after 14 days of administration. The combination of observed pharmacokinetic data and preclinical WRN inhibition kinetics enabled pharmacokinetic and target engagement predictions to be made for different dose levels and regimens (Fig. 2d,e). These predictions suggested that BID or TID schedules would be optimal to ensure continuous WRN inhibition given the short half-life of RO7589831 in vivo. Despite this, disease control and patient benefit were observed across all dose levels and schedules tested. A clear exposure–efficacy relationship could not be established at this time. Higher C1D1 *C*_max_ was significantly associated (chi-squared test, *P* = 0.0002) with increased grade 2+ gastrointestinal toxicity, with a model-predicted 50% risk threshold identified at 7,286 ng ml^−1^ (Fig. 2f).

FDG-PET was used as an exploratory, noninvasive, sensitive imaging biomarker for assessing pharmacodynamic changes in tumor metabolic activity at 3 and 6 weeks after RO7589831 initiation. Approximately half of all FDG-PET-evaluable patients (26 out of 53) showed a dose- and indication-independent reduction in tumor metabolic activity consistent with PMR (−25%, according to European Organisation for Research and Treatment of Cancer (EORTC) criteria). Patients who achieved RECIST v.1.1 cPR consistently showed deep metabolic responses (>50% reduction) early in the course of treatment, while achieving CMR/PMR at any FDG-PET visit was associated with marked clinical benefit that was characterized by durable responses and extended PFS.

Pharmacodynamic molecular response to RO7589831 was assessed by exploratory ctDNA measurements. Molecular responses were observed in 63.4% (26 out of 41) of ctDNA-evaluable patients after RO7589831 treatment. Notably, molecular responses were observed in all ctDNA-evaluable patients who achieved RECIST v.1.1 cPR (*n* = 5) and in 86.9% (20 out of 23) of ctDNA-evaluable patients with RECIST v.1.1 SD; there was no difference in the depth of molecular response between patients with cPR and those with SD. In contrast, ctDNA levels did not achieve molecular response in all but one patient with PD (Fig. 4b). Collectively, these data suggest that early ctDNA dynamics may be used as an exploratory measure of clinical response to RO7589831, although further investigation is needed to verify this.

A main limitation of the study centered around the challenges of demonstrating the pharmacokinetic–pharmacodynamic relationships that had been established in preclinical studies. This is a common challenge for first-in-class molecules, for which human efficacy and biomarker paradigms have yet to become established. Although paired tumor biopsies were collected from 26 patients across different dose levels and indications, direct assessment of pharmacodynamic biomarker and target engagement data from MSI tumor tissue yielded no interpretable results. This was primarily due to the strong presence of mucins and lack of tumor tissue in most samples. As WRN had been readily detected in fresh-frozen human tissue resections and consistent WRN target engagement by RO7589831 had been observed in ex vivo tumor lysates, this complication was unforeseen. These pathological features, which were unique to the patient biopsies, also precluded the characterization of TA repeat expansions, which are potential drivers of increased sensitivity to WRN inhibition within the context of MSI^26,28^.

In this study, most MSI efficacy-evaluable heavily pretreated patients (74.2%) achieved disease control, predominantly as SD, while objective responses were more infrequent (10.6%). This pattern suggests that WRN inhibition primarily suppresses tumor metabolic activity and growth, rather than inducing rapid tumor regression. The high prevalence of mucinous histology, as observed in tumor biopsy samples collected in this trial, may have contributed to this observation, as substantial acellular mucin can mask reductions in viable tumor cell burden on radiological imaging. Mechanistically, preclinical studies demonstrate that WRN inhibition modulates DNA replication stress and cell-cycle progression, with effects that may be restricted to specific cell-cycle phases (S phase, and S/G2 transition) to induce a progressive, *trans*-cell-cycle accumulation of DNA damage^31^. Moreover, synthetic lethal interactions do not uniformly lead to acute cytotoxicity^32^ and may instead produce proliferative arrest or gradual attrition of tumor cells, potentially manifesting clinically as durable disease stabilization rather than RECIST v.1.1-defined responses. In addition, tumor heterogeneity, which could be characterized by variable TA repeat expansion lengths and co-occurring mutations within MSI and/or dMMR tumors, may facilitate the emergence of treatment-resistant subclones less sensitive to WRN inhibition^33^. At this stage, it is unclear whether asynchronous tumor cell loss, nonuniform SL cytotoxicity or inherent tumor heterogeneity are the underlying cause of the predominantly observed disease stabilization in this trial; however, together, these findings suggest that alternative, exploratory trial end points might be investigated, capturing sustained disease control, or molecular ctDNA or metabolic FDG-PET responses, which may more accurately reflect the clinical activity of WRN inhibitors than tumor shrinkage alone.

To build on these insights, future clinical evaluation will be instrumental in defining how this first-in-class mechanism translates across the broader therapeutic landscape. Larger, stratified cohorts exploring outcomes across different tumor types and previous immunotherapy histories may help decipher the exact determinants of the depth and duration of disease stabilization observed in this study. Furthermore, overcoming the technical complexities of quantifying large-scale TA repeat expansions from confounded clinical specimens represents a translational objective, which is required to optimize patient selection. Given the potential for intratumoral heterogeneity to influence long-term efficacy and drive subclonal evasion, evaluating rational combination strategies represents a logical next step. For instance, pairing WRN inhibitors with immune-checkpoint therapies is a promising avenue, as the accumulation of DNA double-strand breaks could theoretically enhance tumor immunogenicity, a concept that is currently being explored clinically with several drug candidates targeting WRN^29,34–38^.

A recent publication by Picco and colleagues highlighted the potential of treating MSI tumors sequentially with different WRN inhibitors^39^. They observed that, under in vitro selective pressure from different WRN inhibitors, some of the acquired *WRN* mutations conferred broad cross-resistance to many different WRN inhibitors, whereas others caused resistance to specific inhibitors only. Crucially, these resistance mutations preserved the tumor’s sensitivity to alternative WRN inhibitors that operate via a distinct MOA. One may speculate that once the tumor acquires an on-target mutation to a certain WRN inhibitor, sequentially administering a second WRN inhibitor that utilizes a distinct MOA that is unaffected by the initial resistance mutation may allow for continued effective tumor treatment. This concept, however, is based on preclinical observations and warrants further investigation in a clinical setting.

Based on the clinical data from part 1, which is most robust in patients with CRC and shows encouraging PFS and OS data compared to published clinical and real-world data, part 2 of the study is currently enrolling patients with advanced MSI-high or dMMR CRC and will evaluate two doses of RO7589831 in combination with bevacizumab. Evaluation of RO7589831 in combination with pembrolizumab in patients with previously untreated CRC is also planned.

In conclusion, this first-in-human, phase 1 study identified a range of RO7589831 dosing regimens that had manageable safety, favorable pharmacokinetics, pharmacodynamic responses and disease control in patients with MSI advanced solid tumors who had progressed on standard therapies. These data are supportive of an early clinical proof of concept for a synthetic lethal strategy in MSI tumors by effectively drugging WRN. Based on the totality of these data, the 150-mg BID and 600-mg BID doses were selected as the RP2Ds for dose optimization.

## Methods

NCT06004245 was conducted in accordance with the International Council for Harmonisation guideline for Good Clinical Practice and the principles of the Declaration of Helsinki. The protocol was approved by the institutional review board/ethics committee at each participating center (Supplementary Table 1). All patients provided written informed consent and were not compensated.

### Patients

Eligible patients were those aged ≥18 years with MSI and/or dMMR advanced solid tumors (determined as part of the routine diagnostic workup in a certified local laboratory) who had received ≥1 (United States/Canada/United Kingdom) or all available standard therapies (European Union) in the advanced setting or standard therapy that was proven to be ineffective or intolerable. Where ICIs were available as standard of care, all patients should have received and progressed on or following a PD-1 or a programmed death-ligand 1 (PD-L1) inhibitor for the treatment of metastatic or locally advanced cancer. Patients had measurable disease according to RECIST v.1.1, an ECOG performance status score of 0 or 1 and a life expectancy of 12 or more weeks. All patients provided written informed consent.

#### Detailed inclusion criteria

Patients were eligible to be included in the study only if all of the following criteria applied:

1. Willing and able to provide written informed consent for the trial.
2. Be ≥18 years of age on the date of signing informed consent.
3. Life expectancy of ≥12 weeks.
4. ECOG performance status score of 0 or 1.
5. Have an MSI and/or dMMR, histologically or cytologically documented advanced (unresectable and/or metastatic):

- Solid tumor and have received and then progressed following, or are intolerant to, at least one standard treatment regimen in the advanced setting, including standard chemotherapy or checkpoint inhibitors according to tumor type. Where checkpoint inhibitor is available as the standard of care, all patients should have received and progressed on, or following, a PD-1 or a PD-L1 inhibitor for the treatment of metastatic (stage IV) or locally advanced disease, unless such therapies are contraindicated for that patient. Reason for contraindication should be documented.
- Patients will be enrolled based on documented MSI and/or dMMR tumor status determined as part of the routine diagnostic workup in a certified laboratory. MSI and/or dMMR tumor status may also be assessed in a certified laboratory with a test that is compliant with applicable requirements or as part of an approved performance study with an investigational in vitro diagnostic if applicable. MSI and/or dMMR tumor status will be defined as:

(i) dMMR status as assessed by IHC for MMR protein expression (MLH1, MSH2, MSH6 and PMS2) where the loss of one or more proteins indicates dMMR, or
(ii) MSI phenotype as determined by PCR or by tissue NGS.

- Based on emerging data, patients may be enrolled based on MSI tumor status only.

(6) Availability of formaldehyde-fixed paraffin-embedded (FFPE) archival tumor tissue for submission to Sponsor/central laboratory:

- Confirmation of available archival tumor tissue sample is required during screening.
- For participants without archival tissue, a biopsy from either primary or metastatic tumor lesion, deemed medically feasible, must be taken.

(7) Presence of measurable disease according to RECIST v.1.1:

- Previously irradiated lesions can be considered as target lesions only if progressive disease has been unequivocally documented at that site since radiation.
- If a target lesion is selected for biopsies, the target lesion must be of a size that allows RECIST v.1.1 assessments over the course of the study.

(8) For part I backfill cohorts only:

- Accessible lesion(s) that permit at least two biopsies (pretreatment and on treatment) without unacceptable risk of a notable procedural complication. Biopsies will only be conducted if deemed clinically safe and feasible by the investigator.
- If multiple lesions are available, it is preferable to obtain the on-treatment biopsy from the same lesion (or organ) as the pretreatment biopsy, to avoid introduction of heterogeneity related to site of metastasis.
- Based on emerging data, enrollment in the backfill cohorts might be restricted to certain tumor types.

(9) Adequate hematologic and end-organ function, defined using the following laboratory results obtained within 14 days before first dose of study treatment:

- Absolute neutrophil count ≥1.5 × 10^9^ l^−1^ without granulocyte colony-stimulating factor support. Patients with benign ethnic neutropenia with an absolute neutrophil count of ≥ 1,000 are eligible.
- Platelet count ≥75 × 10^9^ l^−1^ without transfusion (within 14 days before first dose of study treatment).
- Hemoglobin ≥9 g dl^−1^ without transfusion (within 14 days before first dose of study treatment).
- Total bilirubin ≤1.5× upper limit of normal (ULN) (except patients with Gilbert syndrome).

(i) Note that participants with Gilbert’s syndrome will not be excluded from the study. The diagnosis of Gilbert’s syndrome is suspected in people who have persistent, slightly elevated levels of unconjugated bilirubin levels (≤3 × ULN) without any other apparent cause.

- AST and ALT ≤ 2.5 × ULN. Participants with documented liver metastases: AST and ALT ≤ 5 × ULN.
- Creatinine clearance ≥50 ml min^−1^ based on the Cockcroft–Gault estimation.
- For participants not receiving therapeutic anticoagulation, international normalized ratio or activated partial thromboplastin time ≤1.5 × ULN.
- Patients receiving therapeutic anticoagulation should be on a stable dose for at least 1 week before C1D1.

(10) Adequate cardiovascular function:

- New York Heart Association (NYHA) heart failure class ≤2. Patients with NYHA class 2 must have left ventricular ejection fraction ≥45%.
- Resting systolic blood pressure ≤150 mm Hg and diastolic blood pressure 100 mm Hg (average of ≥3 readings on ≤2 sessions with short break between sessions) (or no clinically notable hypertension).
- Resting heart rate between 45 and 100 bpm (or no clinically notable tachycardia).

##### Contraception requirements

The contraception and abstinence requirements are intended to prevent exposure of an embryo to the study treatment. The reliability of sexual abstinence for male and/or female enrollment eligibility needs to be evaluated in relation to the duration of the clinical study and the preferred and usual lifestyle of the participant. Periodic abstinence (for example calendar, ovulation, symptothermal or post-ovulation methods) and withdrawal are not acceptable methods of preventing pregnancy.

(11) Participants of childbearing potential (POCBP):
  A POCBP is eligible to participate if they are not pregnant, not breastfeeding and at least one of the following conditions applies:
    (i) A participant of non-childbearing potential (PONCBP).
    (ii) A POCBP, who:
      1. Agrees to remain abstinent (refrain from heterosexual intercourse) or use two nonhormonal contraceptive methods including at least one method that results in a failure rate of <1% per year during the treatment period and for at least 1 month after the final dose of RO7589831. POCBP must refrain from donating eggs during this same period. Biological female participants must refrain from donating eggs during this same period. The investigator should evaluate with the participant the best contraceptive method based on anatomy and physiology.
      2. Examples of contraceptive methods with a failure rate of <1% per year include bilateral tubal occlusion/ligation, male sexual partner who is sterilized, and copper intrauterine devices. Barrier methods (male or female condom with or without spermicide, cap, diaphragm or sponge with spermicide) are not highly effective contraceptive methods and must be used alongside a highly effective method. Hormonal contraceptive methods are not considered acceptable.
(12) Biological male PONCBP:
  During the treatment period and for at least 1 month after the final dose of RO7589831, agreement to:
    (i) Remain abstinent (refrain from heterosexual intercourse) or use contraceptive measures such as a condom with a female partner of childbearing potential. The investigator should evaluate with the participant the best contraceptive method based on anatomy and physiology.
    (ii) With a pregnant female partner, remain abstinent (refrain from heterosexual intercourse) or use contraceptive measures such as a condom to avoid exposing the embryo.
    (iii) Refrain from donating semen.

#### Detailed exclusion criteria

Patients were excluded from the study if any of the following criteria applied:

##### Medical conditions

1. Inability or unwillingness to swallow pills.
2. Malabsorption syndrome or another condition that would interfere with enteral absorption.
3. Known hypersensitivity or intolerance to ingredients from the study drug formulation, including patients with rare genetic disorders such as galactosemia, glucose–galactose intolerance or congenital lactase deficiency.
4. Known uncontrolled central nervous system metastases (progressing or requiring anticonvulsants or corticosteroids for symptomatic control) and/or carcinomatous meningitis.
  Participants with previously treated brain metastases may participate provided they are stable (without evidence of progression by imaging for at least 4 weeks before the first dose of study treatment and any neurologic symptoms have returned to baseline), have no evidence of new or enlarging brain metastases, are clinically stable off corticosteroids for at least 14 days before study treatment and have measurable disease, per RECIST v.1.1, outside the central nervous system. This exception does not include carcinomatous meningitis, as individuals with carcinomatous meningitis are excluded regardless of clinical stability.
5. Known active or uncontrolled bacterial, viral, fungal, mycobacterial (including but not limited to tuberculosis and atypical mycobacterial disease), parasitic or other infection (excluding fungal infections of nail beds), or any major episode of infection requiring treatment with intravenous antibiotics or hospitalization within 2 weeks before the start of drug administration (related to the completion of the course of antibiotics, except if for tumor fever) or 6 months for any intracranial abscess.
  Participants receiving prophylactic antibiotics (for example to prevent a urinary tract infection or chronic obstructive pulmonary disease exacerbation) are eligible for the study. Note that antibiotics known to be moderate and strong CYP3A inhibitors or inducers are not allowed.
6. Has a positive test for hepatitis B virus (defined as positive for hepatitis B surface antigen or hepatitis B virus DNA) or hepatitis C virus (HCV; defined as positive for HCV antibody or HCV RNA) per local diagnostic standard and in accordance with local laws and regulations, indicating acute or chronic infection.
7. Positive HIV test at screening per local diagnostic standard and in accordance with local laws and regulations (unless testing is not allowed by local health authority) or taking protease inhibitors as antiretroviral therapy for HIV.
8. Uncontrolled diabetes or symptomatic hyperglycemia (well controlled is defined as a screening hemoglobin A1c < 8% with no urinary ketoacidosis).
9. Notable cardiovascular/cerebrovascular disease within 6 months before day 1 of study drug administration, including any of the following:
  Hypertensive crisis/encephalopathy.
  Unstable angina.
  NYHA class III or IV cardiac disease or congestive heart failure requiring medication.
  Serious cardiac arrhythmia requiring treatment (exceptions are atrial fibrillation, paroxysmal supraventricular tachycardia).
  Baseline-corrected QT (through use of Fridericia’s formula (QTcF)) interval >470 ms based on average of the screening triplicate 12-lead ECG.
  History of thromboembolic events (such as myocardial infarction, stroke or pulmonary embolism).
    (i) Patients with history of pulmonary embolism may be eligible if event is considered resolved in the opinion of the investigator and as per local guidelines.
10. History of malignancy other than the one under investigation, within 2 years before screening, with the exception of:
  Malignancies with a negligible risk of metastasis or death (for example 5-year OS rate >90%) and no anticipated requirement for anticancer therapy during the duration of the study, such as adequately treated carcinoma in situ of the cervix, nonmelanoma skin carcinoma, localized prostate cancer (Gleason score grade <7), ductal carcinoma in situ or stage I uterine cancer.
  Patients with multiple malignancies in the past or at the screening due to Lynch syndrome are not excluded.
11. Alcohol or drug dependence or abuse.
12. Patients with known WRN syndrome.
13. Any medical condition or abnormality in clinical laboratory tests that, in the investigator’s judgment, precludes the participant’s safe participation in and completion of the study, or which could affect compliance with the protocol or interpretation of results.

##### Previous and concomitant therapy

(14) Radiotherapy or major surgical procedure other than for diagnosis within 3 weeks before initiation of study treatment, or anticipation of need for a major surgical procedure during the study. Participants should be recovered from the effects of radiation and/or surgery.
  Palliative radiotherapy is allowed if completed within 2 weeks before start of study treatment.
(15) Previous treatment with any WRN helicase inhibitor.
(16) Systemic anticancer therapy beside the one allowed in the protocol, including but not limited to chemotherapy, investigational drugs, hormonal therapy and antibody based agents (including checkpoint inhibitors) within 28 days or at least five half-lives, whichever is shorter, before start of study treatment.
(17) Treatment-related toxicities from previous cancer therapy that have not resolved to grade ≤1 CTCAE before study treatment except for:
  Immune-related adverse events such as hypothyroidism and adrenal insufficiency on a stable dosage of hormone replacement therapy.
  The following grade 2 toxicities: alopecia, peripheral neuropathy and any laboratory changes that still lie within the inclusion criteria defined above.
(18) Treatment with moderate or strong CYP3A4 inducers within 14 days before initiation of study treatment.
(19) Treatment with moderate or strong CYP3A4 inhibitors within 7 days before initiation of study treatment.

##### Other exclusions

(20) Pregnancy, breastfeeding or intention of becoming pregnant during the study.
  Note that a POCBP must have a negative serum pregnancy test result within 1 week before initiation of study treatment and have a negative urine or serum pregnancy test at C1D1 pre-dose.

### Study design

This was an open-label, multicenter, first-in-human, phase 1 study (NCT06004245). In part 1, escalating doses of RO7589831 were administered orally in 21-day cycles at a starting QD dose of 150 mg. Dose escalation was guided by a modified continual reassessment method with an escalation with overdose control. The dosing schedule could be modified in the event of unacceptable toxicity or unfavorable pharmacokinetics. Due to the short half-life of the drug, dosing was switched to BID, and later, TID regimens, rather than continuing with QD. The DLT period was from C1D1 until C1D21; subsequent dose cohorts were only opened after three DLT-evaluable patients were observed. Selection of dose levels for the randomized backfill cohorts and number of patients allocated to each cohort was based on emerging safety, pharmacokinetic/pharmacodynamic and early response data.

Concomitant therapies were administered at the discretion of the investigator in keeping with community standards of medical care. All adverse events were graded according to the National Cancer Institute Common Terminology Criteria for Adverse Events v.5.0. General recommendations for the management of RO7589831-related adverse events were provided in the study protocol. Specific guidelines for the management of gastrointestinal adverse events were provided to the investigators. Prophylactic administration of antiemetics for the mitigation of nausea and vomiting was not permitted before initial study treatment; however, pre-emptive availability of at-home antiemetics was recommended for timely symptom management. A light meal was also recommended when taking the study drug following investigator feedback of reduced nausea and vomiting. Treatment interruption was recommended for persistent grade 1–2 events or grade 3 events, until resolution to grade 1 or better, with optional dose reduction by one dose level on resumption of study treatment.

Patients with MSI were assessed for response by the investigators according to RECIST v.1.1, whereas those with MSS or without MSI results were excluded from the efficacy analysis. Patients with missing or no on-treatment response assessments were considered not response-evaluable. Pharmacokinetic parameters were derived from plasma concentrations via blood samples taken after single and multiple dosing.

### Study objectives

Primary objectives of the study were to evaluate safety, including determination of the MTD via assessment of DLTs, and identify the RP2D for RO7589831 monotherapy. Secondary objectives were to evaluate pharmacokinetics and antitumor activity of RO7589831 monotherapy. Exploratory objectives included evaluation of pharmacodynamic effects and biomarkers associated with tumor response.

### Pharmacokinetic methods

Blood samples for pharmacokinetic determinations were collected in FC mix tubes (Greiner Bio-one, 454514) containing sodium EDTA and stabilizers sodium fluoride, citric acid and sodium citrate. To prevent hemolysis, samples were inverted a minimum of ten times and centrifuged within 20 min of collection. Plasma concentrations of RO7589831-002 were determined by Labcorp using a validated LC–MS/MS method. A 25.0-µl plasma aliquot was combined with 25.0 µl of internal standard working solution ((D_6_)-RO7589831 in 30:70 acetonitrile:water, 100 ng ml^−1^ final concentration in analyzed samples), vortex mixed for 5 min at 800 revolutions per minute (rpm), and precipitated with 250 µl acetonitrile, again vortex mixed at 800 rpm for 5 min. Samples were then centrifuged at 3,000*g* for 5 min at room temperature, then 30.0 µl of the supernatant was transferred and diluted with 125 µl of water:formic acid (100:0.1), then vortex mixed at 1,500 rpm for 2 min. A 5-µl injection was performed onto a Waters Acquity BEH C18 50 × 2.1 mm, 1.7-µm column maintained at 30 °C. The HPLC mobile phase consisted of water:formic acid (100:0.1) (A) and acetonitrile (B) at a flow rate of 0.75 ml min^−1^. The linear gradient began at 30% B, increasing to 32% B over 5.00 min, followed by a high-organic wash at 98% B for 30 s before re-equilibration. RO7589831-002 eluted at approximately 3.72 min and was detected using a Sciex API 6500+ mass spectrometer in positive electrospray mode. The monitored mass transitions were ml 438.1 > 263.1 for the analyte and *m*/*z* 444.2 > 269.1 for the internal standard. The validated LLoQ was 1.00 ng ml^−1^ in human plasma.

### Population pharmacokinetic and target engagement modeling

A population pharmacokinetic model was developed early on during the study. Pharmacokinetic data was best described by a one compartmental model^40^. Observed auto-induction impact on the pharmacokinetic profile was described by an enzymatic compartment with proportional effect to RO7589831 concentration to the enzymatic production^20^. Enzymatic status did have both effects on relative bioavailability and clearance. Absorption was best described with a delayed zero-order absorption.

Target engagement simulations were based upon a turnover model of WRN protein using a half-life of 16.6 h (ref. ^41^). The inactivation kinetics parameters (binding affinity, *K*_I_ and the rate of inactivation, *k*_inact_) were derived from in vitro experiments performed in HCT-116 cells.

### Gastrointestinal toxicity exposure-response analysis

A logistic regression model (SAS v.9.4) characterized the relationship between C1D1 *C*_max_ and grade 2+ gastrointestinal toxicity. The analysis included 88 patients (29 events), ensuring *C*_max_ occurred before toxicity onset. Significance was assessed via a Wald chi-squared test. Results were grouped by regimen (QD, BID and TID) and visualized with 95% confidence bands.

### FoundationOne CDx tissue mutation profiling

MSI status and mutational profiling from tumor tissue were determined using the FoundationOne CDx (F1CDx) assay^42^. Besides the standard genomic readouts, genomic signatures including TMB and MSI are also reported. To determine MSI status, F1CDx employs a fraction based MSI algorithm to categorize a specimen as MSI-High or MSS by calculating the fraction of >2,000 microsatellite loci determined to be unstable, reported as MSI score.

### FoundationOne Liquid CDx ctDNA mutation profiling

Mutational profiling from blood was determined using the F1LCDx assay, a US Food and Drug Administration-approved NGS assay^43^. Besides standard genomic readouts, genomic signatures, including bTMB, MSI and ctDNA TF, are also evaluated by Foundation Medicine using proprietary algorithms. ctDNA TF is estimated based on aneuploidy or, when undetectable, from somatic alterations^44^. Mutation status of short variants reported as known or likely pathogenic and copy number losses were included in our analysis.

### ctDNA

ctDNA was quantified at C1D1 and C2D1 by either a tumor-informed or a tumor-agnostic assay. A tumor-informed F1T utilized data from F1CDx to identify patient-specific somatic alterations^45^. A proprietary algorithm selected monitorable alterations that are highly likely somatic and not germline. Monitorable alterations were then quantified in plasma using a multiplex PCR assay adapted from the validated Signatera assay^46^. The LLoQ for F1T was estimated at 0.001%. Alternatively, the tumor-agnostic ctDNA fraction (ctDNA TF) derived from F1LCDx was used instead if tumor-informed ctDNA was not available due to missing baseline samples and discontinuation of F1T during the ongoing study. The LLoQ for ctDNA TF was estimated at 0.01.

To utilize results from the two assays in the same analysis, we transformed MTM per ml from F1T by applying log_2_(*x* + 1) and ctDNA TF from F1LCDx by applying logit(*x*), where *x* was capped at 0.01 from below, defined as the LLoQ of F1LCDx. Baseline measures for which results from both assays were available (*n* = 22) were fitted via linear regression (*R*^2^ = 0.82; Extended Data Fig. 3). The resulting regression coefficients were used to scale the F1LCDx measures to their tracker prediction to obtain imputed MTM per ml. Back transforming the resulting value and combining with tracker scores resulted in the extended imputed ctDNA dataset used for analysis. Of note, for change from baseline analysis, data from paired samples was required to be derived from the same assay.

### MMR testing

To assess patients’ dMMR tumor status, baseline FFPE tumor samples (either archival or fresh) were tested for mismatch repair proteins (MLH1, PMS2, MSH2 and MSH6) using qualitative IHC assays by VENTANA. The IHC assays include the following VENTANA primary monoclonal antibodies: mouse anti-MLH1 (M1), mouse anti-PMS2 (A16-4), mouse anti-MSH2 (G219-1129) and rabbit anti-MSH6 (SP93). The OptiView DAB IHC Detection kit is used for MLH1, MSH2 and MSH6, and the OptiView DAB IHC Detection kit with the OptiView Amplification kit is used for PMS2 on a VENTANA BenchMark ULTRA instrument.

### FDG-PET

Metabolic activity was investigated by FDG-PET at baseline, C2D1 (day 21) and C3D1 (day 42). Baseline and follow-up FDG-PET scans were conducted using the same scanner and imaging conditions. FDG-PET scans were accompanied by low-dose computed tomography scans for attenuation correction. Images were assessed based on EORTC guidelines^47^. At baseline, up to five lesions were selected for quantitative analysis. Tumor volumes of interest were defined using manual or semiautomatic tools to derive the SUV_max_ for each lesion. Changes in the sum of individual SUV_max_ (total SUV_max_) between baseline and follow-up visits were calculated for each patient.

### Statistical analysis

The sample sizes for the dose-escalation and backfill cohorts were based on the occurrence of DLTs and the number of dose levels required to determine the MTD and/or RP2D. A maximum of 60 patients was needed to provide a safe and accurate estimation of the MTD. A maximum of 40 patients were required to characterize the dose–pharmacodynamic relationship.

The Medidata RAVE electronic data capture system was used to capture, manage and clean the trial data. Tables were generated using SAS (v.9.4). Figures were generated using R (v.4.5.1). Population pharmacokinetic modeling was performed using Monolix Suite (2024R1).

Safety analyses were performed in all patients who received ≥1 dose of study treatment. Pharmacokinetic analyses were carried out using data from all patients who received ≥1 dose of study treatment and had data from ≥1 post-dose sample. Pharmacokinetic data points inconsistent with the individual’s pharmacokinetic profile were considered outliers and removed manually. Efficacy analyses were performed in all patients with MSI tumors (by central testing and/or pre-existing local testing) who had a valid baseline tumor assessment and ≥1 post-baseline tumor assessment. Exact 95% CIs using the Clopper–Pearson method were provided for overall response rate and DCR. DOR was analyzed using Kaplan–Meier methods. Sex was based on self-reported demographics obtained at screening. No sex-based analyses were performed, as this was a phase I study.

A dose decision team, comprising members of the Sponsor’s study team and investigators, was established to review the emerging data and to make decisions on dose escalation.

Dose escalation was performed using doses of 150 mg QD, 150 mg BID, 300 mg BID, 600 mg BID and 1,000 mg BID. Tolerability issues (gastrointestinal TRAEs of nausea and vomiting) at the 1,000 mg BID dose level resulted in dose reductions in seven patients (45.5%), the Sponsor therefore decided to stop dose escalation at this level. Backfill cohorts were subsequently recruited at 150 mg QD, 200 mg TID and 600 mg TID. The third patient recruited at the 600 mg TID dose had a DLT. In accordance with the protocol, additional patients were recruited at this dose level. No further DLTs were observed, resulting in a final DLT rate of 1 of 15 patients (6.7%). MTD was therefore not determined in this study. The protocol methodology is provided below:

A model-based modified continual reassessment method was used to determine the MTD. Toxicity data from all enrolled evaluable patients were used to update the dose–toxicity curve and make recommendations for the next dose to be tested and evaluate whether the dose escalation could be stopped, for example, if sufficient information to define the MTD had already been collected. The algorithm would recommend ending dose escalation if any of the following criteria were met:

- Enough information on the MTD: a minimum of 12 patients evaluated and at least six patients accrued near the MTD (differing from the MTD by no more than 20%) and posterior probability above 50% that the MTD was within the target toxicity interval.
- Maximum dose was safe: at least six patients had been observed at or near the maximum dose (differing from the MTD by no more than 20%) and it was at least 50% likely that the probability of a DLT for the maximum dose was below 0.2.
- Maximum number of participants: the maximum sample size of 60 DLT-evaluable patients was reached. Biopsy cohort patients were not included in the number of patients in the stopping rule.

### Reporting summary

Further information on research design is available in the Nature Portfolio Reporting Summary linked to this article.

## Online content

Any methods, additional references, Nature Portfolio reporting summaries, source data, extended data, supplementary information, acknowledgements, peer review information; details of author contributions and competing interests; and statements of data and code availability are available at https://doi.org/10.1038/s41591-026-04657-3.

## Supplementary information

Supplementary Information Supplementary tables and figures, output listing all TEAEs, output listing all TRAEs, BP44474 study protocol and amendment summary.

Reporting Summary

## Figures and Tables

**Extended Data Table 1:**
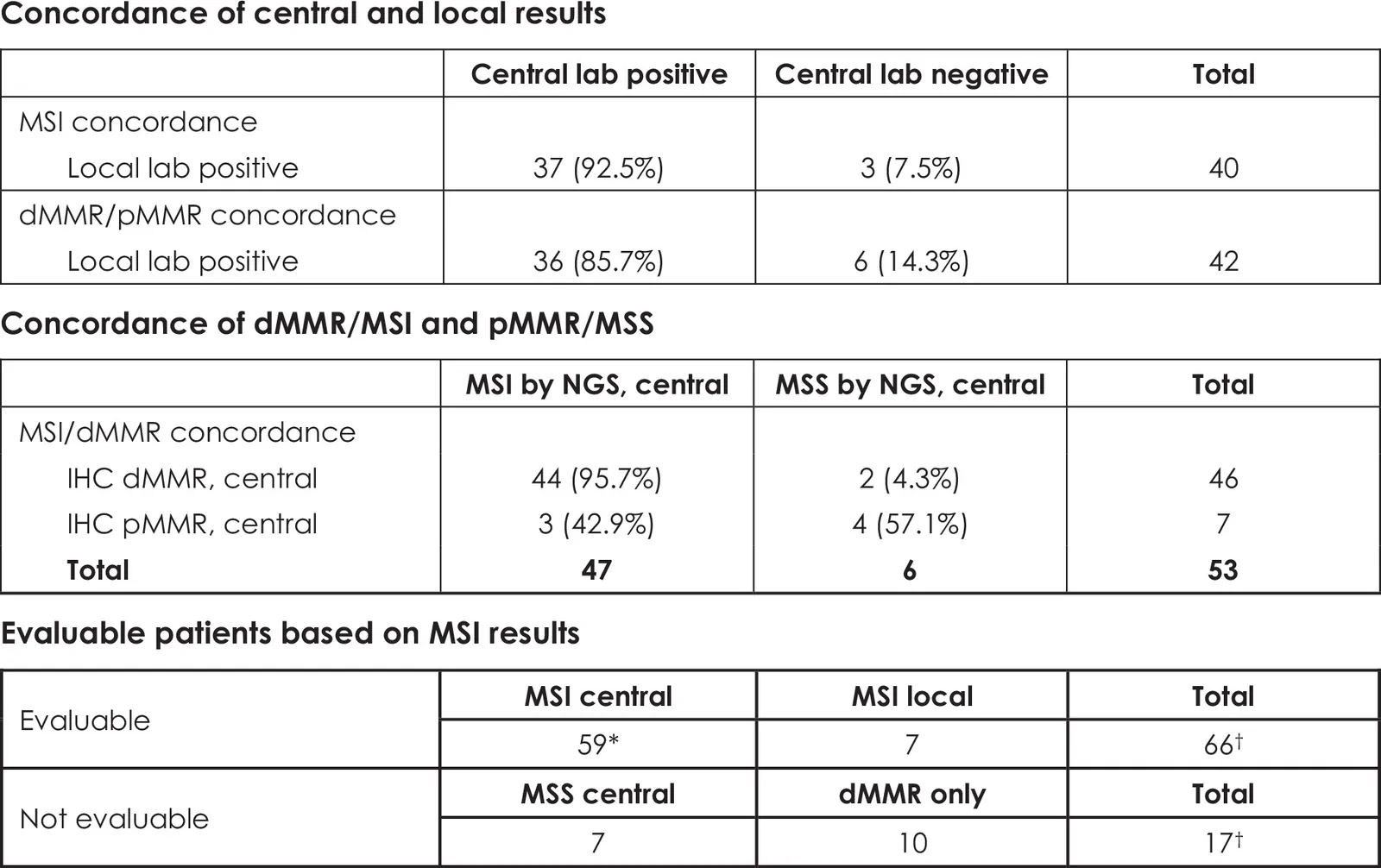
Overview of dMMR and MSI results from local and central testing (concordance)

Central confirmation of local results was 92.5% for MSI and 85.7% for dMMR in patients with both results available. Furthermore, 95.7% of dMMR tumors (central results only) were also MSI, which is consistent with mismatch repair deficiency leading to MSI. For 10 patients, where neither a local nor central MSI result was obtained, enrollment was based on local dMMR results. *Two patients were MS-equivocal per central assessment, but had dMMR and were included. ^†^Five additional patients had no RECISTv1.1 scan on-treatment with centrally confirmed MSI. dMMR, mismatch repair deficient; IHC, immunohistochemistry; MS, microsatellite; MSI, microsatellite instability; MSS, microsatellite stable; NGS, next-generation sequencing; pMMR, proficient mismatch repair.

**Extended Data Table 2:**
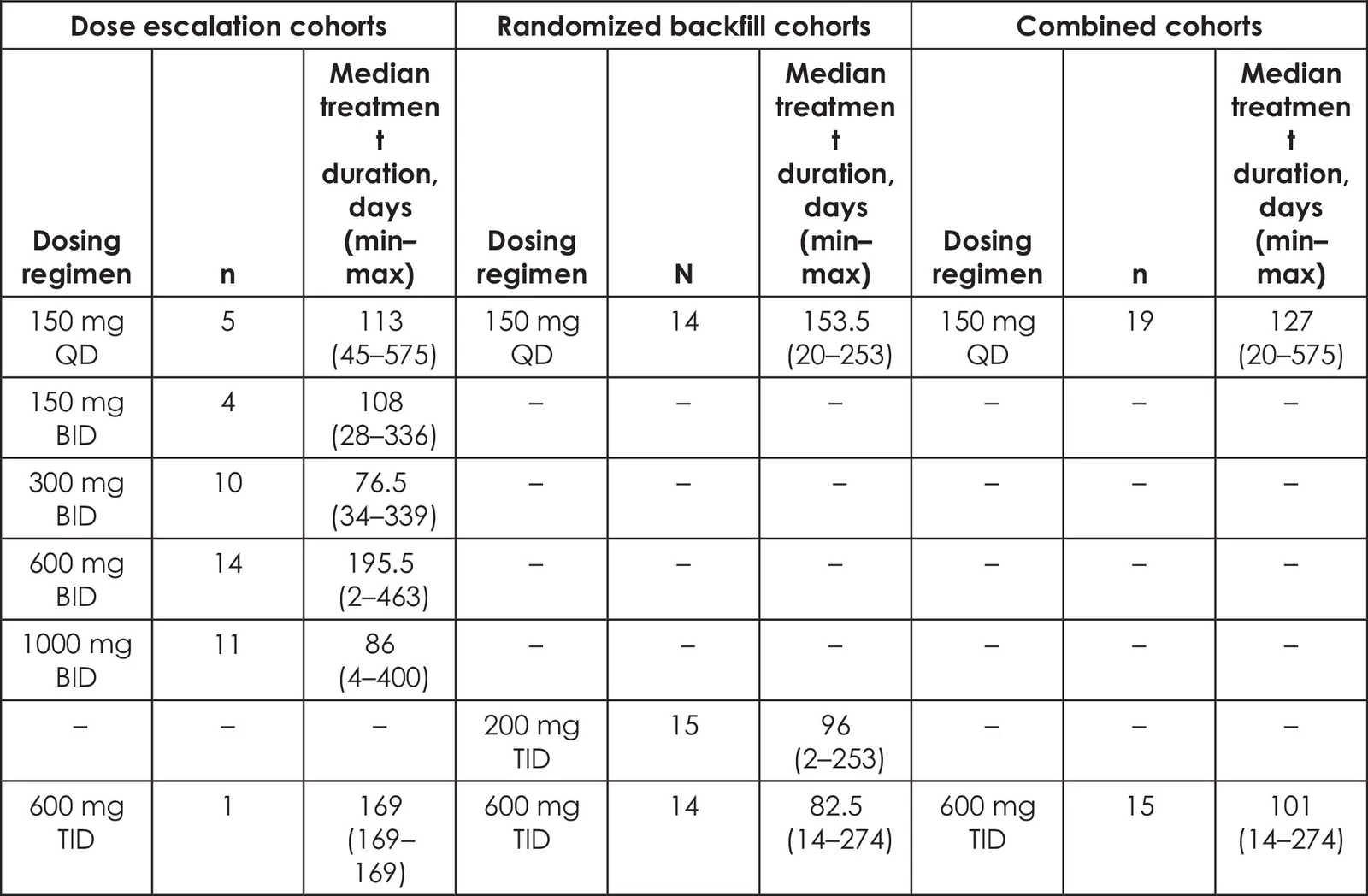
Overview of enrollment and treatment duration in the dose escalation and randomized backfill cohorts

A total of 88 patients were enrolled across all cohorts. Median treatment duration across all cohorts combined was 115 days (min–max: 2–575). BID, twice a day; QD, once a day; TID, three times a day.

**Extended Data Table 3:**
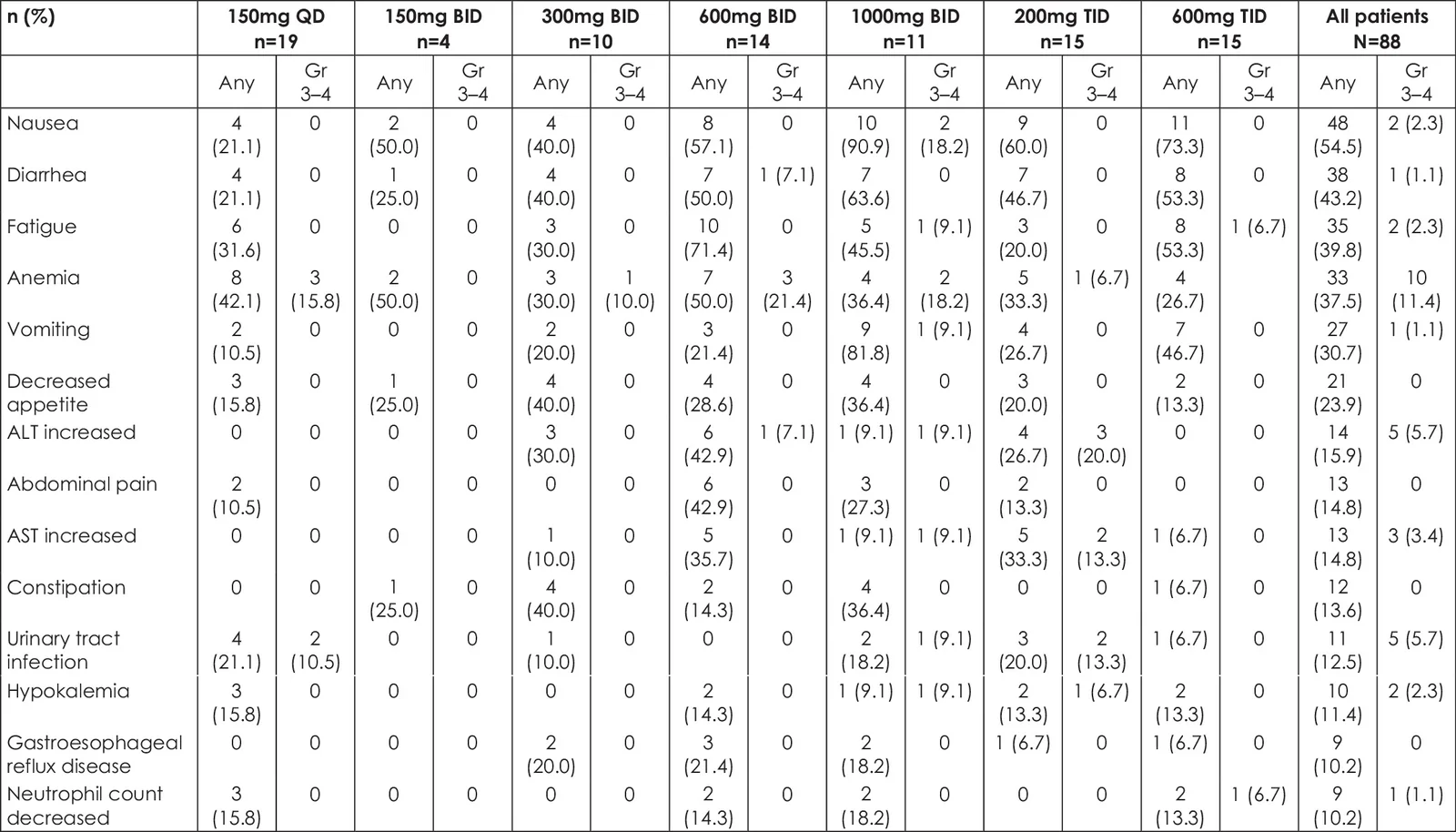
Treatment-emergent adverse events in ≥ 10% patients who received RO7589831 monotherapy

ALT, alanine aminotransferase; AST, aspartate aminotransferase; BID, twice a day; Gr, grade; QD, once a day; TID, three times a day.

**Extended Data Table 4:**
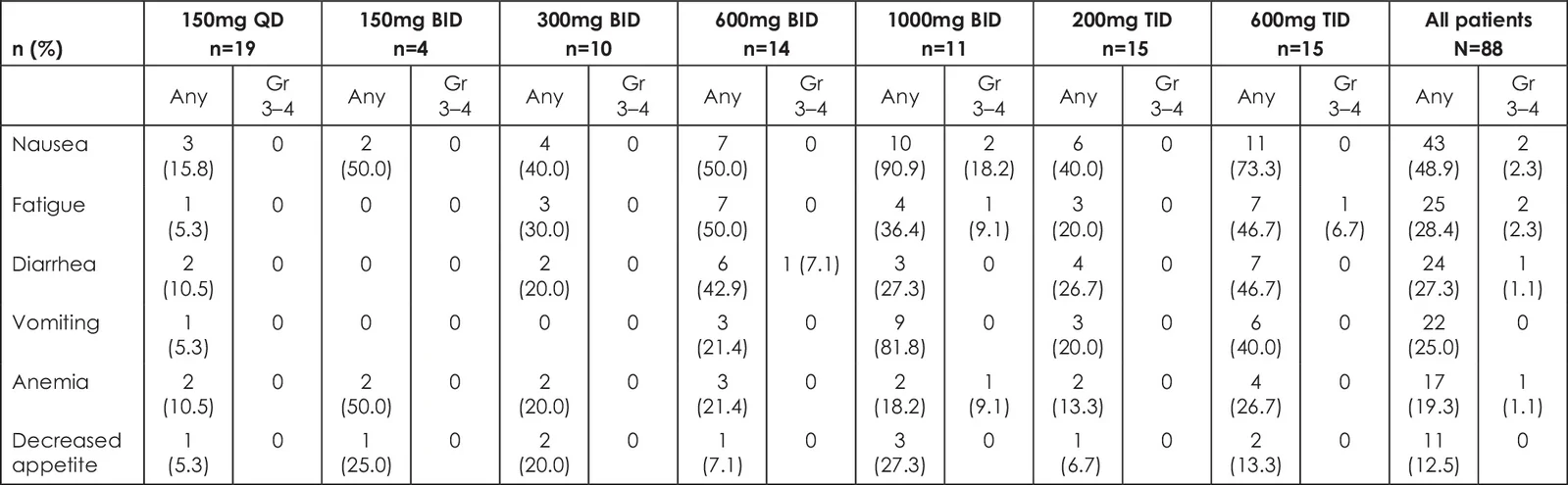
Treatment-related adverse events in ≥ 10% patients who received RO7589831 monotherapy

BID, twice a day; Gr, grade; QD, once a day; TID, three times a day.

**Extended Data Table 5:**
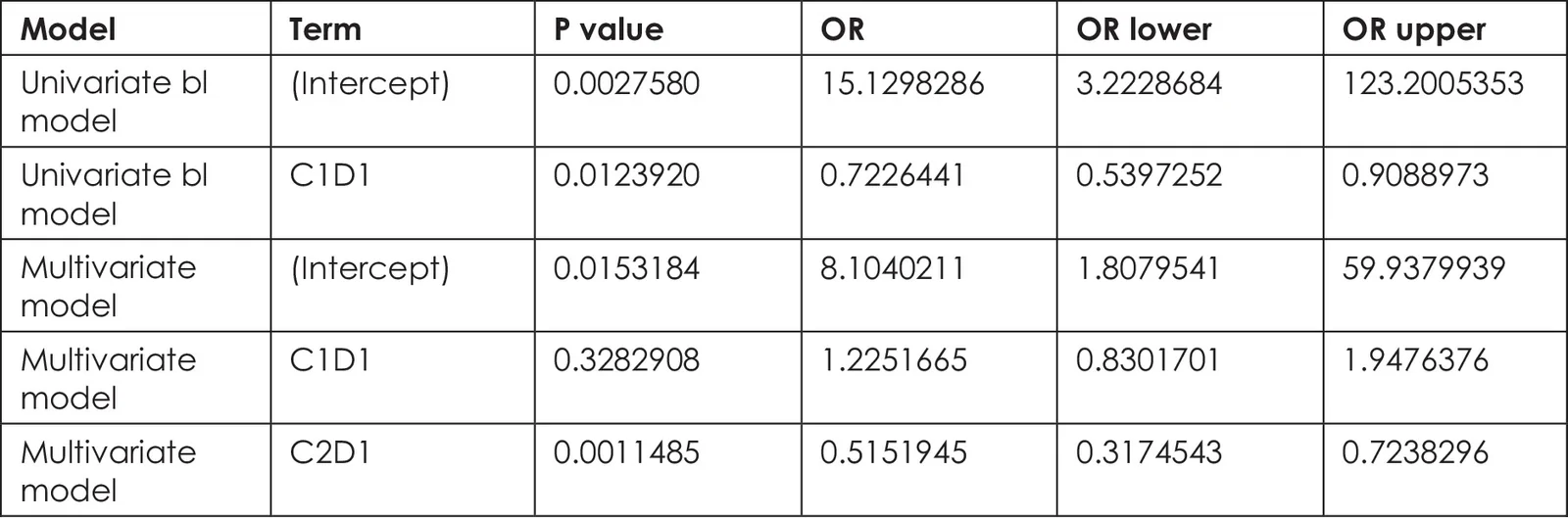
Logistic regression analysis on ctDNA timepoint association with DCR

Shown are the results from associating C1D1 and C2D1 ctDNA levels for samples, where both measurements were available with disease control response outcome using logistic regression. The two evaluated models were either univariable using the C1D1 ctDNA data or multivariable using both C1D1 and C2D1 ctDNA data. C, Cycle; ctDNA, circulating tumor DNA; D, Day; DCR, disease control rate; OR, odds ratio.

**Extended Data Table 6:**
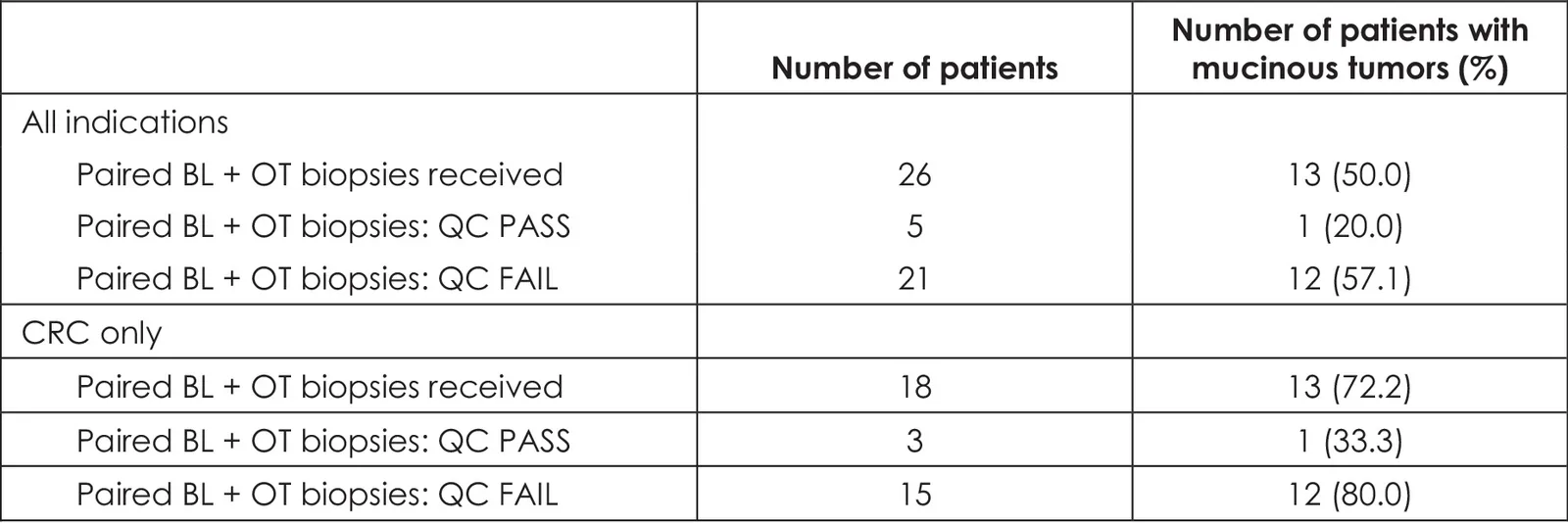
Assessment of biopsy tissue quality and mucin status for all biopsies received and for CRC only

BL, baseline; CRC, colorectal cancer; OT, on-treatment; QC, quality check.

## Data Availability

For eligible studies, qualified researchers may request access to individual patient-level clinical data via the cross-industry request site https://vivli.org. Full details of Roche’s approach to sharing individual patient-level clinical data via the Vivli site can be found at https://vivli.org/ourmember/roche. In summary, access requests are assessed by an Independent Review Panel managed by Vivli. The panel considers the scientific merit of each application and compliance with patient consent and data-sharing policies, with the timeline for request approval (usually 2–5 months) influenced by the number of data contributors, the number of studies and the requestor’s availability to respond to comments. Once approved, the data are anonymized and made available for a minimum of 12 months. For up-to-date details on Roche’s Global Policy on the Sharing of Clinical Information and how to request access to related clinical study documents, see https://go.roche.com/data_sharing. Anonymized records for individual patients across more than one data source external to Roche cannot, and should not, be linked due to a potential increase in risk of patient re-identification.
