# Supplementary material for: Werner helicase inhibitor for advanced microsatellite instability solid tumors: a phase 1 trial
Source: Nat Med. 2026 Sep 29;32(11):4068–78. doi: 10.1038/s41591-026-04657-3 (PMC13768166; doi:10.1038/s41591-026-04657-3)
Supplement: Supplementary file 1 [file 41591_2026_4657_MOESM1_ESM.pdf]

nature medicine

Supplementary information

https://doi.org/10.1038/s41591-026-04657-3

Werner helicase inhibitor for advanced microsatellite instability solid tumors: a phase 1 trial

In the format provided by the authors and unedited

Supplementary Figure 1. Representative examples of biopsy tissue samples showing minimal viable tumor cell areas and extensive mucinous tumor areas. (A) Matched baseline and on-treatment biopsy samples from Patient F shown alongside the corresponding quantification tables (values in mm^2). (B) Baseline biopsy sample from Patient N formatted as in (A).

Whole-slide H&E-stained images of FFPE tumor biopsies were manually annotated to delineate total tumor area and distinct histological sub-compartments. Digital tissue masks highlight specific tumor and non-tumor regions by color (pink: viable tumor cells; green: tumor stroma; brown: mucinous tumor; turquoise: necrosis; grey: non-tumor tissue). Paired images display the annotated overlay mask (left panel) and original H&E stain (right panel). BL, baseline; FFPE, formalin-fixed paraffin-embedded; oT, on-treatment.

(A)

Pat ID	Time	viable tumor cell area (mm2)	tumor stroma area (mm2)	mucinous tumor area (mm2)	total tumor area (mm2)	necrosis area (mm2)
F	BL	0.33	0.54	3.94	4.81	0
F	oT	0.18	1.2	2.05	3.43	0

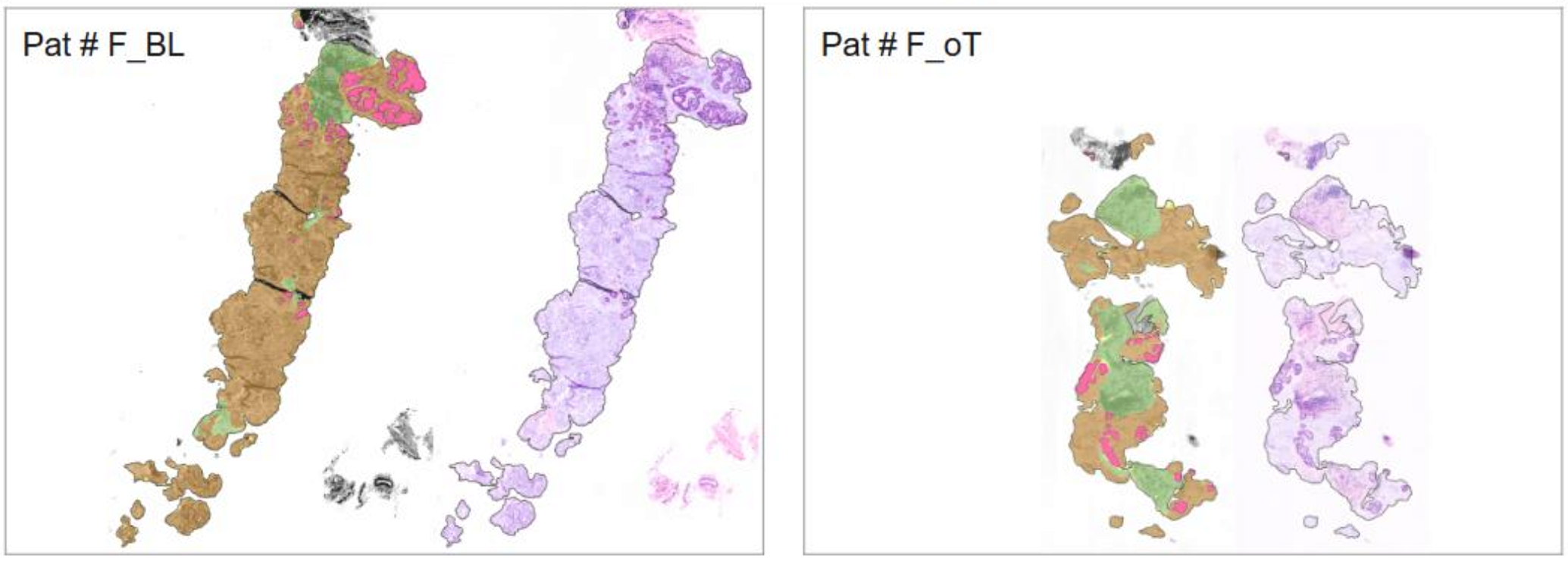

(B)

Pat ID	Time	viable tumor cell area (mm2)	tumor stroma area (mm2)	mucinous tumor area (mm2)	total tumor area (mm2)	necrosis area (mm2)
N	BL	0.2	1.08	4.27	5.55	0.59

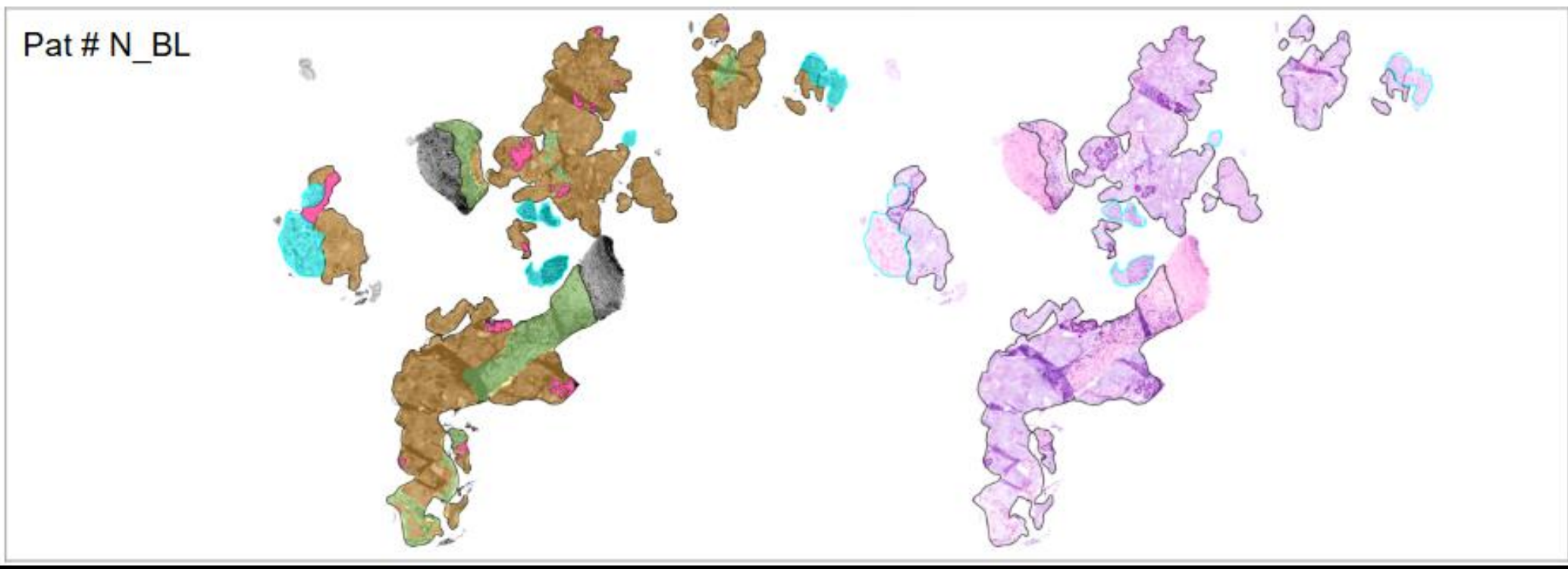

Supplementary Figure 2. WRN target engagement in HCT-116-derived xenografts after treatment of tumor-bearing mice with RO7589831. WRN target engagement by RO7589831 was determined using LC-MS/MS in ex vivo tumor lysates derived from an HCT-116 xenograft mouse model of human CRC. HCT-116 tumor-bearing mice were treated with vehicle or RO7589831 at 1, 3, 10 and 30 mg/kg. WRN target engagement was assessed at 1, 3 and 7 hours post-dose. The minimum number of biological replicates/animals per dose and time point was 3 and the maximum was 4. Results demonstrate that RO7589831 rapidly binds to WRN in a dose-dependent manner. Mass spectrometric detection was carried out on a Sciex Triple quad 6500+ mass spectrometer using scheduled multiple reaction monitoring in the positive ion mode. RO7589831 selectively and irreversibly binds WRN via the cysteine 727 residue. Because covalent modification alters the molecular mass of the corresponding tryptic peptide, target engagement was monitored indirectly through the relative reduction in free, unmodified peptide. Reference peptides from WRN outside the binding site served as control metrics for target protein abundance. Analytical variability was controlled by adding synthetic stable isotope-labelled analogs of each peptide as internal standards. Mean target (±SD) engagement is indirectly estimated by monitoring the level of unengaged cysteine-containing peptide A722 normalized to other peptides not involved in the binding to RO7589831, in percentage, against the level observed on average in all vehicle-treated animals (% unengaged peptide from all vehicle-treated animals normalized to 4 WRN control peptides). CRC, colorectal cancer; LC-MS/MS, liquid chromatography-tandem mass spectrometry; SD, standard deviation; WRN, Werner syndrome helicase.

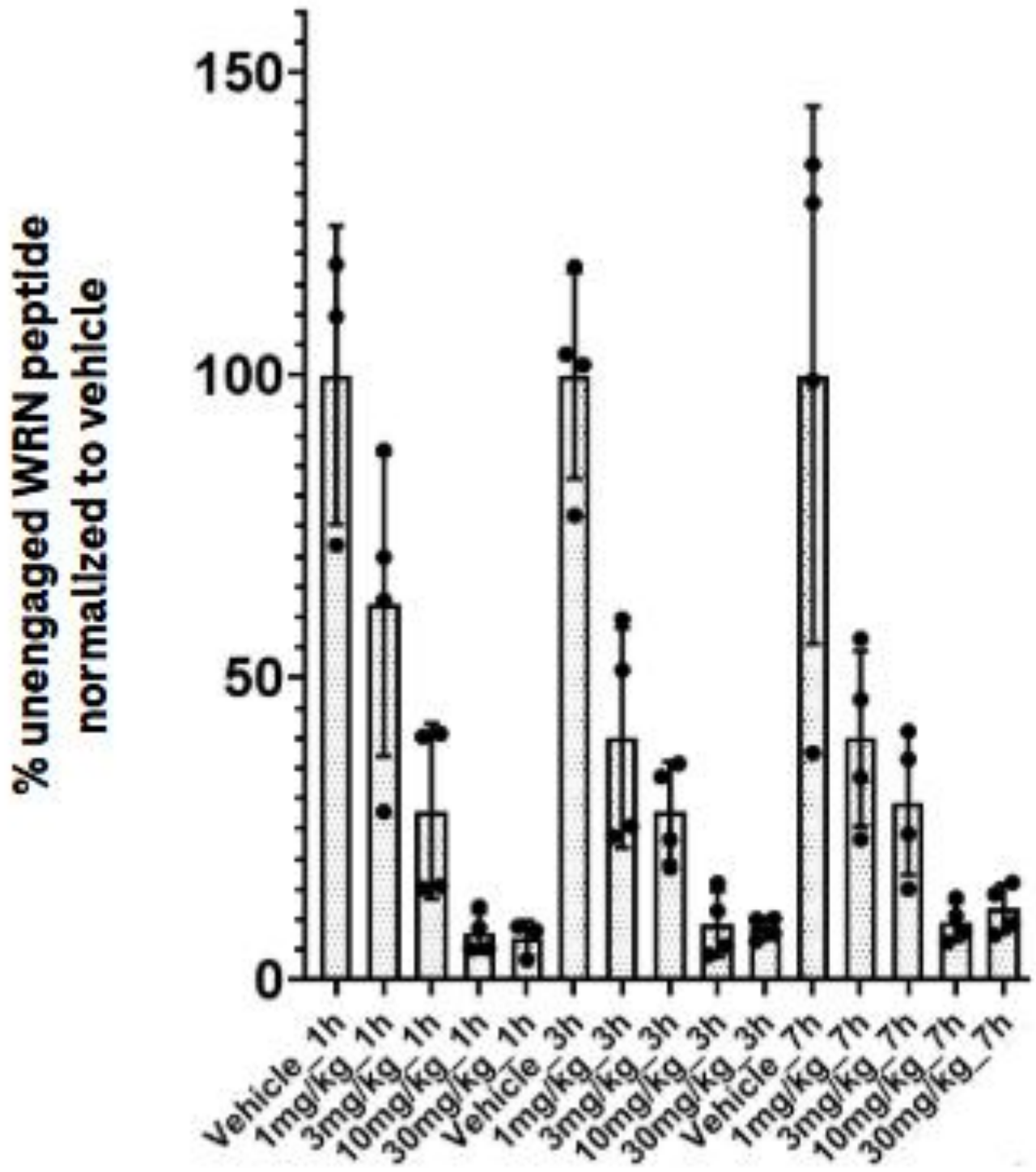

% unengaged WRN peptide normalized to vehicle
150
100
50
0
Vehicle_1h
1mg/kg_1h
3mg/kg_1h
10mg/kg_1h
30mg/kg_1h
Vehicle_3h
1mg/kg_3h
3mg/kg_3h
10mg/kg_3h
30mg/kg_3h
Vehicle_7h
1mg/kg_7h
3mg/kg_7h
10mg/kg_7h
30mg/kg_7h

SUPPLEMENTARY TABLES

Supplementary Table 1. Study sites, locations and investigators.

**Investigator**	**Site**	**Location**
Malaka Ameratunga	The Alfred Hospital	Melbourne, VIC, Australia
Oliver Bechter	Universitair Ziekenhuis (UZ) Leuven	Leuven, Belgium
Emiliano Calvo	START Madrid-CIOCC, Centro Integral Oncológico Clara Campal	Madrid, Spain
Natalie Cook	The Christie NHS Foundation Trust	Manchester, UK
Marwan Fakih	City of Hope – Santa Clarita	Santa Clarita, CA, US
Elisa Fontana	Sarah Cannon Research Institute	London, UK
Jaspreet Grewal	Norton Cancer Institute	Louisville, KY, US
Ignacio Matos	Clinica Universidad de Navarra	Pamplona, Spain
Meredith Pelster	Sarah Cannon Research Institute	Nashville, TN, US
Sophie Postel-Vinay	Institut Gustave Roussy	Villejuif, France
Kristoffer S. Rohrberg	Rigshospitalet	Copenhagen, Denmark
Javier Ros	Vall d'Hebron Institut d'Oncologia (VHIO)	Barcelona, Spain
Adam Sharp	Institute of Cancer Research	London, UK
John H. Strickler	Duke University Medical Center	Durham, NC, US
Erica S. Tsang	Princess Margaret Cancer Centre	Toronto, ON, Canada
Susanna Ulahannan	University of Oklahoma Health Sciences Center – Oklahoma City	Oklahoma City, OK, US
Timothy A. Yap	[1]University of Texas MD Anderson Cancer Center	Houston, TX, US

Supplementary Table 2. Overview of tissue biopsy samples received and their mass spectrometry tumor engagement assay performance. BL, baseline; BLQ, below the limit of quantification (<0.06 fmol of WRN protein/µg of total protein); NOR, no valid results (batch failed; decision not to repeat); oT, on-treatment; TND/INT, target not detected/interference; WRN, Werner syndrome helicase.

**Number of patients**		21	
**Number of fresh frozen biopsy samples received**		36	
**Number of quantitative results**		4	
**Number of results BLQ**		17	
**Number of results with TND/INT**		31	
**Patient**	**Visit**	**Total WRN concentration in fmol/µg of total protein**	**Unengaged WRN concentration in fmol/µg of total protein**
A	BL	TND/INT	TND/INT
A	oT	TND/INT	TND/INT
B	BL	TND/INT	0.100
B	oT	TND/INT	TND/INT
C	BL	TND/INT	TND/INT
C	oT	TND/INT	TND/INT
D	BL	TND/INT	TND/INT
E	BL	0.111	0.132
F	BL	BLQ	BLQ
F	oT	BLQ	TND/INT
G	BL	BLQ	BLQ
H	BL	TND/INT	TND/INT
H	oT	BLQ	TND/INT
I	BL	TND/INT	TND/INT
J	BL	TND/INT	TND/INT
K	BL	BLQ	BLQ
K	oT	BLQ	TND/INT
L	BL	BLQ	TND/INT
L	oT	0.075	BLQ
M	BL	TND/INT	BLQ
M	oT	TND/INT	TND/INT
N	BL	TND/INT	BLQ
N	oT	BLQ	BLQ
O	BL	BLQ	BLQ
O	oT	TND/INT	TND/INT

P	BL	TND/INT	TND/INT
Q	BL	NOR	NOR
Q	oT	NOR	NOR
R	BL	NOR	NOR
R	oT	NOR	NOR
S	BL	NOR	NOR
S	oT	NOR	NOR
T	BL	NOR	NOR
T	oT	NOR	NOR
U	BL	NOR	NOR
U	oT	NOR	NOR

Supplementary Table 3. WRN detection in 11 human fresh frozen tumor blocks. Both peptides of WRN (A722* and A977†) were robustly detected across all fresh frozen human tumor resections, including primary colon carcinomas and peritoneal/liver metastasis. As these baseline specimens were untreated with a covalent WRN inhibitor, it reflects endogenous target protein expression, which is quite consistent across the diverse tumor types (AUC ratio of the two peptides A722/A977 with an overall coefficient of variation of 32%). *Amino acid sequence: NPQITCTGFDRPNLYLEVR. †Amino acid sequence: FGIGLPILFLR. AUC, area under the concentration-time curve; CRC, colorectal cancer; WRN, Werner syndrome helicase.

Tumor ID	Type	Organ	AUC ratio A722	AUC ratio A977	A722/A977
1B217	Metastasis	Peritoneal metastasis of CRC	0.00327	0.00057	5.781
1B613	Metastasis	Peritoneal metastasis of CRC	0.00806	0.00114	7.074
A3127	Metastasis	Peritoneal metastasis of CRC	0.01233	0.00211	5.834
A2194	Metastasis	Peritoneal metastasis of CRC	0.00926	0.00196	4.719
1B467	Metastasis	Peritoneal metastasis of CRC	0.01538	0.00293	5.246
B2830	Primary	Colon	0.00911	0.00184	4.943
B2830	Metastasis	Liver metastasis of CRC	0.01037	0.00090	11.573
H1499	Primary	Colon	0.02218	0.00464	4.783
H1499	Metastasis	Liver metastasis of CRC	0.01882	0.00248	7.573
Z684	Primary	Colon	0.00851	0.00168	5.062
Z684	Metastasis	Liver metastasis of CRC	0.00449	0.00071	6.366

Table 14.3.2.1.1
Treatment-Emergent Adverse Events by Preferred Term – Part 1a: Dose Escalation + Backfill
Safety Evaluable Set

	Dose Escalation						
Preferred Term	150mg QD (N=5)	150mg BID (N=4)	300mg BID (N=10)	600mg BID (N=14)	1000mg BID (N=11)	600mg TID (N=1)	Total (N=45)
Total # of TEAEs	29	25	73	188	158	8	481
Participants with ≥1 TEAE	5 (100)	4 (100)	10 (100)	14 (100)	11 (100)	1 (100)	45 (100)
Nausea	0	2 (50.0)	4 (40.0)	8 (57.1)	10 (90.9)	1 (100)	25 (55.6)
Diarrhoea	2 (40.0)	1 (25.0)	4 (40.0)	7 (50.0)	7 (63.6)	1 (100)	22 (48.9)
Fatigue	0	0	3 (30.0)	10 (71.4)	5 (45.5)	0	18 (40.0)
Anaemia	1 (20.0)	2 (50.0)	3 (30.0)	7 (50.0)	4 (36.4)	0	17 (37.8)
Vomiting	0	0	2 (20.0)	3 (21.4)	9 (81.8)	1 (100)	15 (33.3)
Decreased appetite	0	1 (25.0)	4 (40.0)	4 (28.6)	4 (36.4)	0	13 (28.9)
Alanine aminotransferase increased	0	0	3 (30.0)	6 (42.9)	1 (9.1)	0	10 (22.2)
Abdominal pain	0	0	0	6 (42.9)	3 (27.3)	0	9 (20.0)
Aspartate aminotransferase increased	0	0	1 (10.0)	5 (35.7)	1 (9.1)	0	7 (15.6)
Constipation	0	1 (25.0)	4 (40.0)	2 (14.3)	4 (36.4)	0	11 (24.4)
Urinary tract infection	1 (20.0)	0	1 (10.0)	0	2 (18.2)	0	4 (8.9)
Hypokalaemia	2 (40.0)	0	0	2 (14.3)	1 (9.1)	0	5 (11.1)
Gastrooesophageal reflux disease	0	0	2 (20.0)	3 (21.4)	2 (18.2)	0	7 (15.6)
Neutrophil count decreased	1 (20.0)	0	0	2 (14.3)	2 (18.2)	0	5 (11.1)
Arthralgia	0	0	0	1 (7.1)	0	1 (100)	2 (4.4)
Blood creatinine increased	0	2 (50.0)	0	2 (14.3)	2 (18.2)	0	6 (13.3)
Dyspepsia	1 (20.0)	0	0	3 (21.4)	1 (9.1)	0	5 (11.1)
Dyspnoea	1 (20.0)	0	1 (10.0)	2 (14.3)	2 (18.2)	0	6 (13.3)
Headache	0	0	2 (20.0)	1 (7.1)	0	0	3 (6.7)
Platelet count decreased	1 (20.0)	1 (25.0)	0	4 (28.6)	1 (9.1)	0	7 (15.6)
Pyrexia	0	1 (25.0)	0	3 (21.4)	1 (9.1)	1 (100)	6 (13.3)
Dizziness	0	0	0	2 (14.3)	1 (9.1)	0	3 (6.7)
Paraesthesia	1 (20.0)	0	1 (10.0)	1 (7.1)	2 (18.2)	0	5 (11.1)
Back pain	0	0	0	1 (7.1)	1 (9.1)	0	2 (4.4)

AE=adverse event; MedDRA=Medical Dictionary for Regulatory Activities; PT=MedDRA preferred term; TEAE=treatment-emergent adverse event.
Note: Version 28.0 of MedDRA was used to code AEs. A participant reporting the same AE multiple times will be counted once within each PT. The PT will first be sorted by descending frequency (number of participants in each PT in the total column), then alphabetically if ties exist.

Table 14.3.2.1.1
Treatment-Emergent Adverse Events by Preferred Term – Part 1a: Dose Escalation + Backfill
Safety Evaluable Set

	Backfill				Dose Escalation + Backfill		Total
Preferred Term	150mg QD (N=14)	200mg TID (N=15)	600mg TID (N=14)	Total (N=43)	150mg QD (N=19)	600mg TID (N=15)	(N=88)
Total # of TEAEs	116	102	134	352	145	142	833
Participants with ≥1 TEAE	13 (92.9)	15 (100)	14 (100)	42 (97.7)	18 (94.7)	15 (100)	87 (98.9)
Nausea	4 (28.6)	9 (60.0)	10 (71.4)	23 (53.5)	4 (21.1)	11 (73.3)	48 (54.5)
Diarrhoea	2 (14.3)	7 (46.7)	7 (50.0)	16 (37.2)	4 (21.1)	8 (53.3)	38 (43.2)
Fatigue	6 (42.9)	3 (20.0)	8 (57.1)	17 (39.5)	6 (31.6)	8 (53.3)	35 (39.8)
Anaemia	7 (50.0)	5 (33.3)	4 (28.6)	16 (37.2)	8 (42.1)	4 (26.7)	33 (37.5)
Vomiting	2 (14.3)	4 (26.7)	6 (42.9)	12 (27.9)	2 (10.5)	7 (46.7)	27 (30.7)
Decreased appetite	3 (21.4)	3 (20.0)	2 (14.3)	8 (18.6)	3 (15.8)	2 (13.3)	21 (23.9)
Alanine aminotransferase increased	0	4 (26.7)	0	4 (9.3)	0	0	14 (15.9)
Abdominal pain	2 (14.3)	2 (13.3)	0	4 (9.3)	2 (10.5)	0	13 (14.8)
Aspartate aminotransferase increased	0	5 (33.3)	1 (7.1)	6 (14.0)	0	1 (6.7)	13 (14.8)
Constipation	0	0	1 (7.1)	1 (2.3)	0	1 (6.7)	12 (13.6)
Urinary tract infection	3 (21.4)	3 (20.0)	1 (7.1)	7 (16.3)	4 (21.1)	1 (6.7)	11 (12.5)
Hypokalaemia	1 (7.1)	2 (13.3)	2 (14.3)	5 (11.6)	3 (15.8)	2 (13.3)	10 (11.4)
Gastrooesophageal reflux disease	0	1 (6.7)	1 (7.1)	2 (4.7)	0	1 (6.7)	9 (10.2)
Neutrophil count decreased	2 (14.3)	0	2 (14.3)	4 (9.3)	3 (15.8)	2 (13.3)	9 (10.2)
Arthralgia	2 (14.3)	0	4 (28.6)	6 (14.0)	2 (10.5)	5 (33.3)	8 (9.1)
Blood creatinine increased	2 (14.3)	0	0	2 (4.7)	2 (10.5)	0	8 (9.1)
Dyspepsia	0	2 (13.3)	1 (7.1)	3 (7.0)	1 (5.3)	1 (6.7)	8 (9.1)
Dyspnoea	1 (7.1)	0	1 (7.1)	2 (4.7)	2 (10.5)	1 (6.7)	8 (9.1)
Headache	1 (7.1)	1 (6.7)	3 (21.4)	5 (11.6)	1 (5.3)	3 (20.0)	8 (9.1)
Platelet count decreased	1 (7.1)	0	0	1 (2.3)	2 (10.5)	0	8 (9.1)
Pyrexia	1 (7.1)	0	1 (7.1)	2 (4.7)	1 (5.3)	2 (13.3)	8 (9.1)
Dizziness	4 (28.6)	0	0	4 (9.3)	4 (21.1)	0	7 (8.0)
Paraesthesia	0	0	2 (14.3)	2 (4.7)	1 (5.3)	2 (13.3)	7 (8.0)
Back pain	2 (14.3)	1 (6.7)	1 (7.1)	4 (9.3)	2 (10.5)	1 (6.7)	6 (6.8)

AE=adverse event; MedDRA=Medical Dictionary for Regulatory Activities; PT=MedDRA preferred term; TEAE=treatment-emergent adverse event.
Note: Version 28.0 of MedDRA was used to code AEs. A participant reporting the same AE multiple times will be counted once within each PT. The PT will first be sorted by descending frequency (number of participants in each PT in the total column), then alphabetically if ties exist.

Table 14.3.2.1.1
Treatment-Emergent Adverse Events by Preferred Term – Part 1a: Dose Escalation + Backfill
Safety Evaluable Set

	Dose Escalation						
Preferred Term	150mg QD (N=5)	150mg BID (N=4)	300mg BID (N=10)	600mg BID (N=14)	1000mg BID (N=11)	600mg TID (N=1)	Total (N=45)
Hypomagnesaemia	0	0	0	1 (7.1)	0	0	1 (2.2)
Lipase increased	0	0	1 (10.0)	1 (7.1)	0	0	2 (4.4)
Oedema peripheral	0	0	0	2 (14.3)	1 (9.1)	0	3 (6.7)
Peripheral sensory neuropathy	0	0	0	2 (14.3)	1 (9.1)	0	3 (6.7)
Cough	1 (20.0)	0	0	3 (21.4)	1 (9.1)	0	5 (11.1)
Dysgeusia	1 (20.0)	0	0	1 (7.1)	1 (9.1)	0	3 (6.7)
Insomnia	0	0	2 (20.0)	0	0	0	2 (4.4)
Rash maculo-papular	1 (20.0)	1 (25.0)	0	1 (7.1)	0	0	3 (6.7)
Abdominal distension	0	0	1 (10.0)	1 (7.1)	0	0	2 (4.4)
Asthenia	0	1 (25.0)	1 (10.0)	0	1 (9.1)	0	3 (6.7)
Blood bilirubin increased	0	0	0	1 (7.1)	0	0	1 (2.2)
Blood creatine phosphokinase increased	0	0	1 (10.0)	1 (7.1)	1 (9.1)	0	3 (6.7)
Dysphagia	0	0	1 (10.0)	1 (7.1)	0	0	2 (4.4)
Hot flush	0	0	1 (10.0)	3 (21.4)	0	0	4 (8.9)
Hyperglycaemia	0	0	0	1 (7.1)	0	0	1 (2.2)
Nasal congestion	1 (20.0)	0	0	0	0	0	1 (2.2)
Proteinuria	0	0	1 (10.0)	2 (14.3)	0	0	3 (6.7)
Rash	0	0	0	2 (14.3)	0	0	2 (4.4)
Tumour pain	1 (20.0)	0	0	2 (14.3)	1 (9.1)	0	4 (8.9)
Amylase increased	0	0	0	1 (7.1)	0	0	1 (2.2)
Blood alkaline phosphatase increased	0	0	0	2 (14.3)	0	0	2 (4.4)
Fall	0	0	1 (10.0)	1 (7.1)	0	0	2 (4.4)
Flushing	0	0	0	0	1 (9.1)	0	1 (2.2)
Groin pain	0	1 (25.0)	2 (20.0)	0	0	0	3 (6.7)
Muscular weakness	0	0	1 (10.0)	0	1 (9.1)	0	2 (4.4)
Myalgia	0	0	1 (10.0)	0	0	0	1 (2.2)

AE=adverse event; MedDRA=Medical Dictionary for Regulatory Activities; PT=MedDRA preferred term; TEAE=treatment-emergent adverse event.
Note: Version 28.0 of MedDRA was used to code AEs. A participant reporting the same AE multiple times will be counted once within each PT. The PT will first be sorted by descending frequency (number of participants in each PT in the total column), then alphabetically if ties exist.

Table 14.3.2.1.1
Treatment-Emergent Adverse Events by Preferred Term – Part 1a: Dose Escalation + Backfill
Safety Evaluable Set

	Backfill				Dose Escalation + Backfill		Total
Preferred Term	150mg QD (N=14)	200mg TID (N=15)	600mg TID (N=14)	Total (N=43)	150mg QD (N=19)	600mg TID (N=15)	(N=88)
Hypomagnesaemia	2 (14.3)	1 (6.7)	2 (14.3)	5 (11.6)	2 (10.5)	2 (13.3)	6 (6.8)
Lipase increased	2 (14.3)	2 (13.3)	0	4 (9.3)	2 (10.5)	0	6 (6.8)
Oedema peripheral	0	1 (6.7)	2 (14.3)	3 (7.0)	0	2 (13.3)	6 (6.8)
Peripheral sensory neuropathy	0	1 (6.7)	2 (14.3)	3 (7.0)	0	2 (13.3)	6 (6.8)
Cough	0	0	0	0	1 (5.3)	0	5 (5.7)
Dysgeusia	0	1 (6.7)	1 (7.1)	2 (4.7)	1 (5.3)	1 (6.7)	5 (5.7)
Insomnia	0	1 (6.7)	2 (14.3)	3 (7.0)	0	2 (13.3)	5 (5.7)
Rash maculo-papular	1 (7.1)	0	1 (7.1)	2 (4.7)	2 (10.5)	1 (6.7)	5 (5.7)
Abdominal distension	0	2 (13.3)	0	2 (4.7)	0	0	4 (4.5)
Asthenia	0	0	1 (7.1)	1 (2.3)	0	1 (6.7)	4 (4.5)
Blood bilirubin increased	1 (7.1)	2 (13.3)	0	3 (7.0)	1 (5.3)	0	4 (4.5)
Blood creatine phosphokinase increased	0	1 (6.7)	0	1 (2.3)	0	0	4 (4.5)
Dysphagia	2 (14.3)	0	0	2 (4.7)	2 (10.5)	0	4 (4.5)
Hot flush	0	0	0	0	0	0	4 (4.5)
Hyperglycaemia	1 (7.1)	0	2 (14.3)	3 (7.0)	1 (5.3)	2 (13.3)	4 (4.5)
Nasal congestion	1 (7.1)	1 (6.7)	1 (7.1)	3 (7.0)	2 (10.5)	1 (6.7)	4 (4.5)
Proteinuria	0	1 (6.7)	0	1 (2.3)	0	0	4 (4.5)
Rash	0	1 (6.7)	1 (7.1)	2 (4.7)	0	1 (6.7)	4 (4.5)
Tumour pain	0	0	0	0	1 (5.3)	0	4 (4.5)
Amylase increased	1 (7.1)	1 (6.7)	0	2 (4.7)	1 (5.3)	0	3 (3.4)
Blood alkaline phosphatase increased	0	1 (6.7)	0	1 (2.3)	0	0	3 (3.4)
Fall	0	0	1 (7.1)	1 (2.3)	0	1 (6.7)	3 (3.4)
Flushing	0	1 (6.7)	1 (7.1)	2 (4.7)	0	1 (6.7)	3 (3.4)
Groin pain	0	0	0	0	0	0	3 (3.4)
Muscular weakness	0	1 (6.7)	0	1 (2.3)	0	0	3 (3.4)
Myalgia	1 (7.1)	0	1 (7.1)	2 (4.7)	1 (5.3)	1 (6.7)	3 (3.4)

AE=adverse event; MedDRA=Medical Dictionary for Regulatory Activities; PT=MedDRA preferred term; TEAE=treatment-emergent adverse event.
Note: Version 28.0 of MedDRA was used to code AEs. A participant reporting the same AE multiple times will be counted once within each PT. The PT will first be sorted by descending frequency (number of participants in each PT in the total column), then alphabetically if ties exist.

Table 14.3.2.1.1
Treatment-Emergent Adverse Events by Preferred Term – Part 1a: Dose Escalation + Backfill
Safety Evaluable Set

	Dose Escalation						
Preferred Term	150mg QD (N=5)	150mg BID (N=4)	300mg BID (N=10)	600mg BID (N=14)	1000mg BID (N=11)	600mg TID (N=1)	Total (N=45)
Pruritus	0	0	1 (10.0)	1 (7.1)	0	0	2 (4.4)
Pulmonary embolism	1 (20.0)	0	0	0	1 (9.1)	0	2 (4.4)
Small intestinal obstruction	2 (40.0)	0	0	0	1 (9.1)	0	3 (6.7)
Uncoded	0	0	0	0	0	0	0
Vaginal haemorrhage	0	0	0	2 (14.3)	0	0	2 (4.4)
Abdominal pain lower	0	0	1 (10.0)	0	0	0	1 (2.2)
Abdominal pain upper	0	0	0	0	0	0	0
Abnormal faeces	0	0	0	0	0	0	0
Anal incontinence	0	0	0	2 (14.3)	0	0	2 (4.4)
Bronchitis	0	0	0	1 (7.1)	1 (9.1)	0	2 (4.4)
COVID-19	0	0	0	0	0	1 (100)	1 (2.2)
Chills	0	0	0	0	2 (18.2)	0	2 (4.4)
Depression	0	0	1 (10.0)	1 (7.1)	0	0	2 (4.4)
Device related infection	1 (20.0)	0	0	0	0	0	1 (2.2)
Dry mouth	0	0	0	0	1 (9.1)	0	1 (2.2)
Dysuria	1 (20.0)	0	0	0	1 (9.1)	0	2 (4.4)
Embolism	0	0	0	0	1 (9.1)	0	1 (2.2)
Flank pain	0	0	1 (10.0)	0	0	0	1 (2.2)
Gastrointestinal disorder	0	0	0	1 (7.1)	1 (9.1)	0	2 (4.4)
Haematuria	0	0	0	0	0	0	0
Hydronephrosis	0	0	0	0	0	0	0
Hyperkalaemia	0	0	1 (10.0)	0	0	0	1 (2.2)
Hypernatraemia	0	0	0	0	1 (9.1)	0	1 (2.2)
Hypertension	0	0	0	1 (7.1)	0	0	1 (2.2)
Hypertriglyceridaemia	0	0	0	1 (7.1)	0	0	1 (2.2)
Hypoalbuminaemia	1 (20.0)	0	0	0	0	0	1 (2.2)

AE=adverse event; MedDRA=Medical Dictionary for Regulatory Activities; PT=MedDRA preferred term; TEAE=treatment-emergent adverse event.
Note: Version 28.0 of MedDRA was used to code AEs. A participant reporting the same AE multiple times will be counted once within each PT. The PT will first be sorted by descending frequency (number of participants in each PT in the total column), then alphabetically if ties exist.

Table 14.3.2.1.1
Treatment-Emergent Adverse Events by Preferred Term – Part 1a: Dose Escalation + Backfill
Safety Evaluable Set

	Backfill				Dose Escalation + Backfill		Total
Preferred Term	150mg QD (N=14)	200mg TID (N=15)	600mg TID (N=14)	Total (N=43)	150mg QD (N=19)	600mg TID (N=15)	(N=88)
Pruritus	1 (7.1)	0	0	1 (2.3)	1 (5.3)	0	3 (3.4)
Pulmonary embolism	0	0	1 (7.1)	1 (2.3)	1 (5.3)	1 (6.7)	3 (3.4)
Small intestinal obstruction	0	0	0	0	2 (10.5)	0	3 (3.4)
Uncoded	2 (14.3)	0	1 (7.1)	3 (7.0)	2 (10.5)	1 (6.7)	3 (3.4)
Vaginal haemorrhage	1 (7.1)	0	0	1 (2.3)	1 (5.3)	0	3 (3.4)
Abdominal pain lower	0	0	1 (7.1)	1 (2.3)	0	1 (6.7)	2 (2.3)
Abdominal pain upper	0	2 (13.3)	0	2 (4.7)	0	0	2 (2.3)
Abnormal faeces	0	0	2 (14.3)	2 (4.7)	0	2 (13.3)	2 (2.3)
Anal incontinence	0	0	0	0	0	0	2 (2.3)
Bronchitis	0	0	0	0	0	0	2 (2.3)
COVID-19	0	0	1 (7.1)	1 (2.3)	0	2 (13.3)	2 (2.3)
Chills	0	0	0	0	0	0	2 (2.3)
Depression	0	0	0	0	0	0	2 (2.3)
Device related infection	1 (7.1)	0	0	1 (2.3)	2 (10.5)	0	2 (2.3)
Dry mouth	0	0	1 (7.1)	1 (2.3)	0	1 (6.7)	2 (2.3)
Dysuria	0	0	0	0	1 (5.3)	0	2 (2.3)
Embolism	0	0	1 (7.1)	1 (2.3)	0	1 (6.7)	2 (2.3)
Flank pain	0	0	1 (7.1)	1 (2.3)	0	1 (6.7)	2 (2.3)
Gastrointestinal disorder	0	0	0	0	0	0	2 (2.3)
Haematuria	1 (7.1)	1 (6.7)	0	2 (4.7)	1 (5.3)	0	2 (2.3)
Hydronephrosis	1 (7.1)	0	1 (7.1)	2 (4.7)	1 (5.3)	1 (6.7)	2 (2.3)
Hyperkalaemia	1 (7.1)	0	0	1 (2.3)	1 (5.3)	0	2 (2.3)
Hypernatraemia	0	0	1 (7.1)	1 (2.3)	0	1 (6.7)	2 (2.3)
Hypertension	0	1 (6.7)	0	1 (2.3)	0	0	2 (2.3)
Hypertriglyceridaemia	1 (7.1)	0	0	1 (2.3)	1 (5.3)	0	2 (2.3)
Hypoalbuminaemia	0	1 (6.7)	0	1 (2.3)	1 (5.3)	0	2 (2.3)

AE=adverse event; MedDRA=Medical Dictionary for Regulatory Activities; PT=MedDRA preferred term; TEAE=treatment-emergent adverse event.
Note: Version 28.0 of MedDRA was used to code AEs. A participant reporting the same AE multiple times will be counted once within each PT. The PT will first be sorted by descending frequency (number of participants in each PT in the total column), then alphabetically if ties exist.

Table 14.3.2.1.1
Treatment-Emergent Adverse Events by Preferred Term – Part 1a: Dose Escalation + Backfill
Safety Evaluable Set

	Dose Escalation						
Preferred Term	150mg QD (N=5)	150mg BID (N=4)	300mg BID (N=10)	600mg BID (N=14)	1000mg BID (N=11)	600mg TID (N=1)	Total (N=45)
Hypophosphataemia	0	0	0	2 (14.3)	0	0	2 (4.4)
Hypotension	0	0	0	0	1 (9.1)	0	1 (2.2)
Ileus	0	0	0	0	1 (9.1)	0	1 (2.2)
Iron deficiency	0	0	0	0	0	0	0
Large intestinal obstruction	0	0	1 (10.0)	0	1 (9.1)	0	2 (4.4)
Lymphocyte count decreased	0	0	0	1 (7.1)	0	0	1 (2.2)
Lymphopenia	0	0	0	0	0	0	0
Nasopharyngitis	0	0	0	0	0	1 (100)	1 (2.2)
Neutropenia	0	0	0	0	0	0	0
Oropharyngeal pain	0	0	0	1 (7.1)	1 (9.1)	0	2 (4.4)
Pain	1 (20.0)	0	0	0	0	0	1 (2.2)
Pelvic pain	1 (20.0)	0	0	0	1 (9.1)	0	2 (4.4)
Pelvic venous thrombosis	0	0	0	0	0	0	0
Pleural effusion	0	0	0	2 (14.3)	0	0	2 (4.4)
Sepsis	0	0	0	0	0	0	0
Sinusitis	0	0	0	0	0	0	0
Skin laceration	0	0	0	0	0	0	0
Stomatitis	0	0	1 (10.0)	0	1 (9.1)	0	2 (4.4)
Transient ischaemic attack	0	1 (25.0)	0	1 (7.1)	0	0	2 (4.4)
Urinary retention	0	0	0	0	1 (9.1)	0	1 (2.2)
Weight decreased	0	0	1 (10.0)	0	0	0	1 (2.2)
White blood cell count decreased	0	0	0	1 (7.1)	0	0	1 (2.2)
Abdominal infection	0	0	0	0	0	0	0
Abdominal wall infection	0	0	0	0	0	0	0
Abscess	0	0	1 (10.0)	0	0	0	1 (2.2)
Anaemia macrocytic	0	0	0	0	0	0	0

AE=adverse event; MedDRA=Medical Dictionary for Regulatory Activities; PT=MedDRA preferred term; TEAE=treatment-emergent adverse event.
Note: Version 28.0 of MedDRA was used to code AEs. A participant reporting the same AE multiple times will be counted once within each PT. The PT will first be sorted by descending frequency (number of participants in each PT in the total column), then alphabetically if ties exist.

Table 14.3.2.1.1
Treatment-Emergent Adverse Events by Preferred Term – Part 1a: Dose Escalation + Backfill
Safety Evaluable Set

	Backfill				Dose Escalation + Backfill		Total
Preferred Term	150mg QD (N=14)	200mg TID (N=15)	600mg TID (N=14)	Total (N=43)	150mg QD (N=19)	600mg TID (N=15)	(N=88)
Hypophosphataemia	0	0	0	0	0	0	2 (2.3)
Hypotension	0	0	1 (7.1)	1 (2.3)	0	1 (6.7)	2 (2.3)
Ileus	0	1 (6.7)	0	1 (2.3)	0	0	2 (2.3)
Iron deficiency	1 (7.1)	0	1 (7.1)	2 (4.7)	1 (5.3)	1 (6.7)	2 (2.3)
Large intestinal obstruction	0	0	0	0	0	0	2 (2.3)
Lymphocyte count decreased	1 (7.1)	0	0	1 (2.3)	1 (5.3)	0	2 (2.3)
Lymphopenia	0	2 (13.3)	0	2 (4.7)	0	0	2 (2.3)
Nasopharyngitis	0	0	1 (7.1)	1 (2.3)	0	2 (13.3)	2 (2.3)
Neutropenia	0	1 (6.7)	1 (7.1)	2 (4.7)	0	1 (6.7)	2 (2.3)
Oropharyngeal pain	0	0	0	0	0	0	2 (2.3)
Pain	0	0	1 (7.1)	1 (2.3)	1 (5.3)	1 (6.7)	2 (2.3)
Pelvic pain	0	0	0	0	1 (5.3)	0	2 (2.3)
Pelvic venous thrombosis	1 (7.1)	0	1 (7.1)	2 (4.7)	1 (5.3)	1 (6.7)	2 (2.3)
Pleural effusion	0	0	0	0	0	0	2 (2.3)
Sepsis	0	0	2 (14.3)	2 (4.7)	0	2 (13.3)	2 (2.3)
Sinusitis	1 (7.1)	0	1 (7.1)	2 (4.7)	1 (5.3)	1 (6.7)	2 (2.3)
Skin laceration	1 (7.1)	0	1 (7.1)	2 (4.7)	1 (5.3)	1 (6.7)	2 (2.3)
Stomatitis	0	0	0	0	0	0	2 (2.3)
Transient ischaemic attack	0	0	0	0	0	0	2 (2.3)
Urinary retention	0	0	1 (7.1)	1 (2.3)	0	1 (6.7)	2 (2.3)
Weight decreased	1 (7.1)	0	0	1 (2.3)	1 (5.3)	0	2 (2.3)
White blood cell count decreased	0	0	1 (7.1)	1 (2.3)	0	1 (6.7)	2 (2.3)
Abdominal infection	0	0	1 (7.1)	1 (2.3)	0	1 (6.7)	1 (1.1)
Abdominal wall infection	0	0	1 (7.1)	1 (2.3)	0	1 (6.7)	1 (1.1)
Abscess	0	0	0	0	0	0	1 (1.1)
Anaemia macrocytic	0	1 (6.7)	0	1 (2.3)	0	0	1 (1.1)

AE=adverse event; MedDRA=Medical Dictionary for Regulatory Activities; PT=MedDRA preferred term; TEAE=treatment-emergent adverse event.
Note: Version 28.0 of MedDRA was used to code AEs. A participant reporting the same AE multiple times will be counted once within each PT. The PT will first be sorted by descending frequency (number of participants in each PT in the total column), then alphabetically if ties exist.

Table 14.3.2.1.1
Treatment-Emergent Adverse Events by Preferred Term – Part 1a: Dose Escalation + Backfill
Safety Evaluable Set

	Dose Escalation						
Preferred Term	150mg QD (N=5)	150mg BID (N=4)	300mg BID (N=10)	600mg BID (N=14)	1000mg BID (N=11)	600mg TID (N=1)	Total (N=45)
Anal fistula	0	0	0	0	0	0	0
Angina pectoris	0	0	0	0	0	0	0
Anxiety	0	0	0	0	0	0	0
Atrial fibrillation	0	0	0	0	1 (9.1)	0	1 (2.2)
Bacteraemia	0	0	0	0	0	0	0
Basal cell carcinoma	0	0	0	0	0	0	0
Blood cholesterol increased	0	0	0	1 (7.1)	0	0	1 (2.2)
Blood creatine phosphokinase decreased	0	0	0	0	0	0	0
Blood lactate dehydrogenase decreased	0	0	0	0	0	0	0
Blood magnesium decreased	0	0	0	0	0	0	0
Blood potassium increased	0	0	0	0	1 (9.1)	0	1 (2.2)
Blood prolactin increased	0	1 (25.0)	0	0	0	0	1 (2.2)
Blood urea decreased	0	0	0	0	0	0	0
Brachiocephalic vein thrombosis	0	0	0	0	0	0	0
Breath odour	0	0	0	0	0	0	0
Cardiac failure	0	0	0	0	1 (9.1)	0	1 (2.2)
Cholecystitis	0	0	0	1 (7.1)	0	0	1 (2.2)
Confusional state	0	0	0	0	0	0	0
Deep vein thrombosis	1 (20.0)	0	0	0	0	0	1 (2.2)
Dehydration	0	0	0	0	1 (9.1)	0	1 (2.2)
Delirium	0	0	0	0	0	0	0
Depressed mood	0	0	1 (10.0)	0	0	0	1 (2.2)
Dermatitis	0	1 (25.0)	0	0	0	0	1 (2.2)
Discouragement	0	0	0	0	0	0	0
Dry skin	1 (20.0)	0	0	0	0	0	1 (2.2)
Dysphonia	0	0	0	0	1 (9.1)	0	1 (2.2)

AE=adverse event; MedDRA=Medical Dictionary for Regulatory Activities; PT=MedDRA preferred term; TEAE=treatment-emergent adverse event.
Note: Version 28.0 of MedDRA was used to code AEs. A participant reporting the same AE multiple times will be counted once within each PT. The PT will first be sorted by descending frequency (number of participants in each PT in the total column), then alphabetically if ties exist.

Table 14.3.2.1.1
Treatment-Emergent Adverse Events by Preferred Term – Part 1a: Dose Escalation + Backfill
Safety Evaluable Set

	Backfill				Dose Escalation + Backfill		Total
Preferred Term	150mg QD (N=14)	200mg TID (N=15)	600mg TID (N=14)	Total (N=43)	150mg QD (N=19)	600mg TID (N=15)	(N=88)
Anal fistula	1 (7.1)	0	0	1 (2.3)	1 (5.3)	0	1 (1.1)
Angina pectoris	0	0	1 (7.1)	1 (2.3)	0	1 (6.7)	1 (1.1)
Anxiety	0	0	1 (7.1)	1 (2.3)	0	1 (6.7)	1 (1.1)
Atrial fibrillation	0	0	0	0	0	0	1 (1.1)
Bacteraemia	0	0	1 (7.1)	1 (2.3)	0	1 (6.7)	1 (1.1)
Basal cell carcinoma	0	0	1 (7.1)	1 (2.3)	0	1 (6.7)	1 (1.1)
Blood cholesterol increased	0	0	0	0	0	0	1 (1.1)
Blood creatine phosphokinase decreased	1 (7.1)	0	0	1 (2.3)	1 (5.3)	0	1 (1.1)
Blood lactate dehydrogenase decreased	0	1 (6.7)	0	1 (2.3)	0	0	1 (1.1)
Blood magnesium decreased	1 (7.1)	0	0	1 (2.3)	1 (5.3)	0	1 (1.1)
Blood potassium increased	0	0	0	0	0	0	1 (1.1)
Blood prolactin increased	0	0	0	0	0	0	1 (1.1)
Blood urea decreased	1 (7.1)	0	0	1 (2.3)	1 (5.3)	0	1 (1.1)
Brachiocephalic vein thrombosis	1 (7.1)	0	0	1 (2.3)	1 (5.3)	0	1 (1.1)
Breath odour	0	0	1 (7.1)	1 (2.3)	0	1 (6.7)	1 (1.1)
Cardiac failure	0	0	0	0	0	0	1 (1.1)
Cholecystitis	0	0	0	0	0	0	1 (1.1)
Confusional state	0	1 (6.7)	0	1 (2.3)	0	0	1 (1.1)
Deep vein thrombosis	0	0	0	0	1 (5.3)	0	1 (1.1)
Dehydration	0	0	0	0	0	0	1 (1.1)
Delirium	1 (7.1)	0	0	1 (2.3)	1 (5.3)	0	1 (1.1)
Depressed mood	0	0	0	0	0	0	1 (1.1)
Dermatitis	0	0	0	0	0	0	1 (1.1)
Discouragement	1 (7.1)	0	0	1 (2.3)	1 (5.3)	0	1 (1.1)
Dry skin	0	0	0	0	1 (5.3)	0	1 (1.1)
Dysphonia	0	0	0	0	0	0	1 (1.1)

AE=adverse event; MedDRA=Medical Dictionary for Regulatory Activities; PT=MedDRA preferred term; TEAE=treatment-emergent adverse event.
Note: Version 28.0 of MedDRA was used to code AEs. A participant reporting the same AE multiple times will be counted once within each PT. The PT will first be sorted by descending frequency (number of participants in each PT in the total column), then alphabetically if ties exist.

Table 14.3.2.1.1
Treatment-Emergent Adverse Events by Preferred Term – Part 1a: Dose Escalation + Backfill
Safety Evaluable Set

	Dose Escalation						
Preferred Term	150mg QD (N=5)	150mg BID (N=4)	300mg BID (N=10)	600mg BID (N=14)	1000mg BID (N=11)	600mg TID (N=1)	Total (N=45)
Early satiety	0	0	0	1 (7.1)	0	0	1 (2.2)
Electrocardiogram PR prolongation	0	0	0	0	0	0	0
Electrocardiogram QT prolonged	0	0	0	0	0	0	0
Epigastric discomfort	0	0	0	1 (7.1)	0	0	1 (2.2)
Epilepsy	0	0	0	1 (7.1)	0	0	1 (2.2)
Escherichia urinary tract infection	0	0	0	0	1 (9.1)	0	1 (2.2)
Extrapyramidal disorder	0	0	0	1 (7.1)	0	0	1 (2.2)
Eye pruritus	0	0	0	0	1 (9.1)	0	1 (2.2)
Face oedema	0	0	0	0	0	0	0
Febrile neutropenia	0	0	0	0	0	0	0
Gait disturbance	0	0	0	0	1 (9.1)	0	1 (2.2)
Gamma-glutamyltransferase increased	0	0	0	0	0	0	0
Gastric varices	0	0	0	0	0	0	0
Gastrointestinal viral infection	0	0	0	0	0	0	0
Haemoglobin decreased	0	0	0	0	0	0	0
Head injury	0	0	0	1 (7.1)	0	0	1 (2.2)
Herpes simplex	0	0	0	1 (7.1)	0	0	1 (2.2)
Hiccups	0	0	0	0	0	0	0
Hypercholesterolaemia	0	0	0	1 (7.1)	0	0	1 (2.2)
Hyperhidrosis	0	0	0	0	0	0	0
Hypoglycaemia	0	0	0	1 (7.1)	0	0	1 (2.2)
Hyponatraemia	0	0	0	0	0	0	0
Hypothermia	0	0	0	0	1 (9.1)	0	1 (2.2)
Impaired gastric emptying	0	0	0	0	0	0	0
Infection	0	1 (25.0)	0	0	0	0	1 (2.2)
Influenza like illness	0	0	0	0	0	0	0

AE=adverse event; MedDRA=Medical Dictionary for Regulatory Activities; PT=MedDRA preferred term; TEAE=treatment-emergent adverse event.
Note: Version 28.0 of MedDRA was used to code AEs. A participant reporting the same AE multiple times will be counted once within each PT. The PT will first be sorted by descending frequency (number of participants in each PT in the total column), then alphabetically if ties exist.

Table 14.3.2.1.1
Treatment-Emergent Adverse Events by Preferred Term – Part 1a: Dose Escalation + Backfill
Safety Evaluable Set

	Backfill				Dose Escalation + Backfill		Total
Preferred Term	150mg QD (N=14)	200mg TID (N=15)	600mg TID (N=14)	Total (N=43)	150mg QD (N=19)	600mg TID (N=15)	(N=88)
Early satiety	0	0	0	0	0	0	1 (1.1)
Electrocardiogram PR prolongation	0	0	1 (7.1)	1 (2.3)	0	1 (6.7)	1 (1.1)
Electrocardiogram QT prolonged	0	1 (6.7)	0	1 (2.3)	0	0	1 (1.1)
Epigastric discomfort	0	0	0	0	0	0	1 (1.1)
Epilepsy	0	0	0	0	0	0	1 (1.1)
Escherichia urinary tract infection	0	0	0	0	0	0	1 (1.1)
Extrapyramidal disorder	0	0	0	0	0	0	1 (1.1)
Eye pruritus	0	0	0	0	0	0	1 (1.1)
Face oedema	1 (7.1)	0	0	1 (2.3)	1 (5.3)	0	1 (1.1)
Febrile neutropenia	0	0	1 (7.1)	1 (2.3)	0	1 (6.7)	1 (1.1)
Gait disturbance	0	0	0	0	0	0	1 (1.1)
Gamma-glutamyltransferase increased	0	0	1 (7.1)	1 (2.3)	0	1 (6.7)	1 (1.1)
Gastric varices	1 (7.1)	0	0	1 (2.3)	1 (5.3)	0	1 (1.1)
Gastrointestinal viral infection	1 (7.1)	0	0	1 (2.3)	1 (5.3)	0	1 (1.1)
Haemoglobin decreased	1 (7.1)	0	0	1 (2.3)	1 (5.3)	0	1 (1.1)
Head injury	0	0	0	0	0	0	1 (1.1)
Herpes simplex	0	0	0	0	0	0	1 (1.1)
Hiccups	0	1 (6.7)	0	1 (2.3)	0	0	1 (1.1)
Hypercholesterolaemia	0	0	0	0	0	0	1 (1.1)
Hyperhidrosis	0	0	1 (7.1)	1 (2.3)	0	1 (6.7)	1 (1.1)
Hypoglycaemia	0	0	0	0	0	0	1 (1.1)
Hyponatraemia	0	0	1 (7.1)	1 (2.3)	0	1 (6.7)	1 (1.1)
Hypothermia	0	0	0	0	0	0	1 (1.1)
Impaired gastric emptying	1 (7.1)	0	0	1 (2.3)	1 (5.3)	0	1 (1.1)
Infection	0	0	0	0	0	0	1 (1.1)
Influenza like illness	1 (7.1)	0	0	1 (2.3)	1 (5.3)	0	1 (1.1)

AE=adverse event; MedDRA=Medical Dictionary for Regulatory Activities; PT=MedDRA preferred term; TEAE=treatment-emergent adverse event.
Note: Version 28.0 of MedDRA was used to code AEs. A participant reporting the same AE multiple times will be counted once within each PT. The PT will first be sorted by descending frequency (number of participants in each PT in the total column), then alphabetically if ties exist.

P:\Vividion\VVD-133214-01\Biostats\Programs\TLFs\t-aept.sas 13NOV2025 13:17

Table 14.3.2.1.1
Treatment-Emergent Adverse Events by Preferred Term – Part 1a: Dose Escalation + Backfill
Safety Evaluable Set

	Dose Escalation						
Preferred Term	150mg QD (N=5)	150mg BID (N=4)	300mg BID (N=10)	600mg BID (N=14)	1000mg BID (N=11)	600mg TID (N=1)	Total (N=45)
Intestinal perforation	0	0	0	0	0	0	0
Memory impairment	0	0	0	0	0	0	0
Mouth ulceration	0	0	1 (10.0)	0	0	0	1 (2.2)
Muscle spasms	0	0	0	0	0	0	0
Musculoskeletal chest pain	0	0	0	0	1 (9.1)	0	1 (2.2)
Musculoskeletal pain	0	0	1 (10.0)	0	0	0	1 (2.2)
Nail disorder	0	0	0	0	0	0	0
Neuropathy peripheral	0	0	0	1 (7.1)	0	0	1 (2.2)
Obstruction gastric	0	0	0	0	0	0	0
Oedema	0	0	0	0	1 (9.1)	0	1 (2.2)
Oesophagitis	0	0	0	0	1 (9.1)	0	1 (2.2)
Pain in extremity	0	0	1 (10.0)	0	0	0	1 (2.2)
Pericardial effusion	0	0	0	1 (7.1)	0	0	1 (2.2)
Pneumonia	0	0	0	1 (7.1)	0	0	1 (2.2)
Post procedural haemorrhage	0	0	0	0	0	0	0
Proctalgia	0	0	1 (10.0)	0	0	0	1 (2.2)
Prostatic specific antigen increased	0	0	0	1 (7.1)	0	0	1 (2.2)
Rash pustular	0	1 (25.0)	0	0	0	0	1 (2.2)
Rectal haemorrhage	0	0	0	0	0	0	0
Renal failure	0	0	0	0	0	0	0
Renal fusion anomaly	0	0	0	0	0	0	0
Rhinitis allergic	0	0	0	0	0	0	0
Sciatica	0	0	0	0	0	0	0
Serum ferritin decreased	0	0	0	0	0	0	0
Sinus tachycardia	0	0	0	1 (7.1)	0	0	1 (2.2)
Skin abrasion	0	0	1 (10.0)	0	0	0	1 (2.2)

AE=adverse event; MedDRA=Medical Dictionary for Regulatory Activities; PT=MedDRA preferred term; TEAE=treatment-emergent adverse event.
Note: Version 28.0 of MedDRA was used to code AEs. A participant reporting the same AE multiple times will be counted once within each PT. The PT will first be sorted by descending frequency (number of participants in each PT in the total column), then alphabetically if ties exist.

Table 14.3.2.1.1
Treatment-Emergent Adverse Events by Preferred Term – Part 1a: Dose Escalation + Backfill
Safety Evaluable Set

	Backfill				Dose Escalation + Backfill		Total
Preferred Term	150mg QD (N=14)	200mg TID (N=15)	600mg TID (N=14)	Total (N=43)	150mg QD (N=19)	600mg TID (N=15)	(N=88)
Intestinal perforation	1 (7.1)	0	0	1 (2.3)	1 (5.3)	0	1 (1.1)
Memory impairment	0	1 (6.7)	0	1 (2.3)	0	0	1 (1.1)
Mouth ulceration	0	0	0	0	0	0	1 (1.1)
Muscle spasms	1 (7.1)	0	0	1 (2.3)	1 (5.3)	0	1 (1.1)
Musculoskeletal chest pain	0	0	0	0	0	0	1 (1.1)
Musculoskeletal pain	0	0	0	0	0	0	1 (1.1)
Nail disorder	0	0	1 (7.1)	1 (2.3)	0	1 (6.7)	1 (1.1)
Neuropathy peripheral	0	0	0	0	0	0	1 (1.1)
Obstruction gastric	0	0	1 (7.1)	1 (2.3)	0	1 (6.7)	1 (1.1)
Oedema	0	0	0	0	0	0	1 (1.1)
Oesophagitis	0	0	0	0	0	0	1 (1.1)
Pain in extremity	0	0	0	0	0	0	1 (1.1)
Pericardial effusion	0	0	0	0	0	0	1 (1.1)
Pneumonia	0	0	0	0	0	0	1 (1.1)
Post procedural haemorrhage	1 (7.1)	0	0	1 (2.3)	1 (5.3)	0	1 (1.1)
Proctalgia	0	0	0	0	0	0	1 (1.1)
Prostatic specific antigen increased	0	0	0	0	0	0	1 (1.1)
Rash pustular	0	0	0	0	0	0	1 (1.1)
Rectal haemorrhage	1 (7.1)	0	0	1 (2.3)	1 (5.3)	0	1 (1.1)
Renal failure	1 (7.1)	0	0	1 (2.3)	1 (5.3)	0	1 (1.1)
Renal fusion anomaly	1 (7.1)	0	0	1 (2.3)	1 (5.3)	0	1 (1.1)
Rhinitis allergic	1 (7.1)	0	0	1 (2.3)	1 (5.3)	0	1 (1.1)
Sciatica	0	0	1 (7.1)	1 (2.3)	0	1 (6.7)	1 (1.1)
Serum ferritin decreased	1 (7.1)	0	0	1 (2.3)	1 (5.3)	0	1 (1.1)
Sinus tachycardia	0	0	0	0	0	0	1 (1.1)
Skin abrasion	0	0	0	0	0	0	1 (1.1)

AE=adverse event; MedDRA=Medical Dictionary for Regulatory Activities; PT=MedDRA preferred term; TEAE=treatment-emergent adverse event.
Note: Version 28.0 of MedDRA was used to code AEs. A participant reporting the same AE multiple times will be counted once within each PT. The PT will first be sorted by descending frequency (number of participants in each PT in the total column), then alphabetically if ties exist.

Table 14.3.2.1.1
Treatment-Emergent Adverse Events by Preferred Term – Part 1a: Dose Escalation + Backfill
Safety Evaluable Set

	Dose Escalation						
Preferred Term	150mg QD (N=5)	150mg BID (N=4)	300mg BID (N=10)	600mg BID (N=14)	1000mg BID (N=11)	600mg TID (N=1)	Total (N=45)
Skin infection	0	0	0	0	0	0	0
Subclavian vein thrombosis	0	0	0	0	0	0	0
Subileus	0	0	0	0	1 (9.1)	0	1 (2.2)
Superinfection	0	0	1 (10.0)	0	0	0	1 (2.2)
Temperature intolerance	0	0	0	0	1 (9.1)	0	1 (2.2)
Thrombocytopenia	0	0	0	1 (7.1)	0	0	1 (2.2)
Tooth abscess	0	0	0	0	0	0	0
Toothache	0	0	0	0	1 (9.1)	0	1 (2.2)
Tumour haemorrhage	0	0	0	1 (7.1)	0	0	1 (2.2)
Upper respiratory tract infection	0	0	0	0	0	0	0
Ureteral stent removal	0	0	1 (10.0)	0	0	0	1 (2.2)
Urine analysis abnormal	1 (20.0)	0	0	0	0	0	1 (2.2)
Urticaria	0	0	0	1 (7.1)	0	0	1 (2.2)
Visual acuity reduced	0	0	0	0	0	0	0
Vitreous floaters	0	0	0	0	0	0	0
Weight increased	0	0	0	0	0	0	0

AE=adverse event; MedDRA=Medical Dictionary for Regulatory Activities; PT=MedDRA preferred term; TEAE=treatment-emergent adverse event.
Note: Version 28.0 of MedDRA was used to code AEs. A participant reporting the same AE multiple times will be counted once within each PT. The PT will first be sorted by descending frequency (number of participants in each PT in the total column), then alphabetically if ties exist.

P:\Vividion\VVD-133214-01\Biostats\Programs\TLFs\t-aept.sas 13NOV2025 13:17

Table 14.3.2.1.1
Treatment-Emergent Adverse Events by Preferred Term – Part 1a: Dose Escalation + Backfill
Safety Evaluable Set

	Backfill				Dose Escalation + Backfill		Total
Preferred Term	150mg QD (N=14)	200mg TID (N=15)	600mg TID (N=14)	Total (N=43)	150mg QD (N=19)	600mg TID (N=15)	(N=88)
Skin infection	0	0	1 (7.1)	1 (2.3)	0	1 (6.7)	1 (1.1)
Subclavian vein thrombosis	1 (7.1)	0	0	1 (2.3)	1 (5.3)	0	1 (1.1)
Subileus	0	0	0	0	0	0	1 (1.1)
Superinfection	0	0	0	0	0	0	1 (1.1)
Temperature intolerance	0	0	0	0	0	0	1 (1.1)
Thrombocytopenia	0	0	0	0	0	0	1 (1.1)
Tooth abscess	0	0	1 (7.1)	1 (2.3)	0	1 (6.7)	1 (1.1)
Toothache	0	0	0	0	0	0	1 (1.1)
Tumour haemorrhage	0	0	0	0	0	0	1 (1.1)
Upper respiratory tract infection	0	1 (6.7)	0	1 (2.3)	0	0	1 (1.1)
Ureteral stent removal	0	0	0	0	0	0	1 (1.1)
Urine analysis abnormal	0	0	0	0	1 (5.3)	0	1 (1.1)
Urticaria	0	0	0	0	0	0	1 (1.1)
Visual acuity reduced	0	1 (6.7)	0	1 (2.3)	0	0	1 (1.1)
Vitreous floaters	1 (7.1)	0	0	1 (2.3)	1 (5.3)	0	1 (1.1)
Weight increased	1 (7.1)	0	0	1 (2.3)	1 (5.3)	0	1 (1.1)

AE=adverse event; MedDRA=Medical Dictionary for Regulatory Activities; PT=MedDRA preferred term; TEAE=treatment-emergent adverse event.
Note: Version 28.0 of MedDRA was used to code AEs. A participant reporting the same AE multiple times will be counted once within each PT. The PT will first be sorted by descending frequency (number of participants in each PT in the total column), then alphabetically if ties exist.

P:\Vividion\VVD-133214-01\Biostats\Programs\TLFs\t-aept.sas 13NOV2025 13:17

Table 14.3.2.4.1
Treatment-Emergent Adverse Events Related to Study Treatment by Preferred Term – Part 1a: Dose Escalation + Backfill
Safety Evaluable Set

	Dose Escalation						
Preferred Term	150mg QD (N=5)	150mg BID (N=4)	300mg BID (N=10)	600mg BID (N=14)	1000mg BID (N=11)	600mg TID (N=1)	Total (N=45)
Total # of TEAEs	3	15	25	94	76	3	216
Participants with ≥1 TEAE	2 (40.0)	3 (75.0)	9 (90.0)	12 (85.7)	11 (100)	1 (100)	38 (84.4)
Nausea	0	2 (50.0)	4 (40.0)	7 (50.0)	10 (90.9)	1 (100)	24 (53.3)
Fatigue	0	0	3 (30.0)	7 (50.0)	4 (36.4)	0	14 (31.1)
Diarrhoea	2 (40.0)	0	2 (20.0)	6 (42.9)	3 (27.3)	1 (100)	14 (31.1)
Vomiting	0	0	0	3 (21.4)	9 (81.8)	1 (100)	13 (28.9)
Anaemia	0	2 (50.0)	2 (20.0)	3 (21.4)	2 (18.2)	0	9 (20.0)
Decreased appetite	0	1 (25.0)	2 (20.0)	1 (7.1)	3 (27.3)	0	7 (15.6)
Gastrooesophageal reflux disease	0	0	1 (10.0)	3 (21.4)	2 (18.2)	0	6 (13.3)
Alanine aminotransferase increased	0	0	0	3 (21.4)	1 (9.1)	0	4 (8.9)
Aspartate aminotransferase increased	0	0	0	3 (21.4)	1 (9.1)	0	4 (8.9)
Dyspepsia	0	0	0	3 (21.4)	1 (9.1)	0	4 (8.9)
Dysgeusia	1 (20.0)	0	0	1 (7.1)	1 (9.1)	0	3 (6.7)
Lipase increased	0	0	1 (10.0)	1 (7.1)	0	0	2 (4.4)
Abdominal pain	0	0	0	4 (28.6)	0	0	4 (8.9)
Hot flush	0	0	1 (10.0)	3 (21.4)	0	0	4 (8.9)
Insomnia	0	0	2 (20.0)	0	0	0	2 (4.4)
Platelet count decreased	0	1 (25.0)	0	2 (14.3)	0	0	3 (6.7)
Asthenia	0	1 (25.0)	1 (10.0)	0	0	0	2 (4.4)
Flushing	0	0	0	0	1 (9.1)	0	1 (2.2)
Headache	0	0	0	1 (7.1)	0	0	1 (2.2)
Neutrophil count decreased	0	0	0	0	1 (9.1)	0	1 (2.2)
Peripheral sensory neuropathy	0	0	0	1 (7.1)	0	0	1 (2.2)
Pruritus	0	0	1 (10.0)	1 (7.1)	0	0	2 (4.4)
Rash maculo-papular	0	1 (25.0)	0	1 (7.1)	0	0	2 (4.4)
Abnormal faeces	0	0	0	0	0	0	0

AE=adverse event; MedDRA=Medical Dictionary for Regulatory Activities; PT=MedDRA preferred term; TEAE=treatment-emergent adverse event.
Note: Version 28.0 of MedDRA was used to code AEs. A participant reporting the same AE multiple times will be counted once within each PT. The PT will first be sorted by descending frequency (number of participants in each PT in the total column), then alphabetically if ties exist.

Table 14.3.2.4.1
Treatment-Emergent Adverse Events Related to Study Treatment by Preferred Term – Part 1a: Dose Escalation + Backfill
Safety Evaluable Set

Preferred Term	Backfill				Dose Escalation + Backfill		Total
	150mg QD (N=14)	200mg TID (N=15)	600mg TID (N=14)	Total (N=43)	150mg QD (N=19)	600mg TID (N=15)	(N=88)
Total # of TEAEs	22	47	74	143	25	77	359
Participants with ≥1 TEAE	6 (42.9)	12 (80.0)	13 (92.9)	31 (72.1)	8 (42.1)	14 (93.3)	69 (78.4)
Nausea	3 (21.4)	6 (40.0)	10 (71.4)	19 (44.2)	3 (15.8)	11 (73.3)	43 (48.9)
Fatigue	1 (7.1)	3 (20.0)	7 (50.0)	11 (25.6)	1 (5.3)	7 (46.7)	25 (28.4)
Diarrhoea	0	4 (26.7)	6 (42.9)	10 (23.3)	2 (10.5)	7 (46.7)	24 (27.3)
Vomiting	1 (7.1)	3 (20.0)	5 (35.7)	9 (20.9)	1 (5.3)	6 (40.0)	22 (25.0)
Anaemia	2 (14.3)	2 (13.3)	4 (28.6)	8 (18.6)	2 (10.5)	4 (26.7)	17 (19.3)
Decreased appetite	1 (7.1)	1 (6.7)	2 (14.3)	4 (9.3)	1 (5.3)	2 (13.3)	11 (12.5)
Gastrooesophageal reflux disease	0	1 (6.7)	1 (7.1)	2 (4.7)	0	1 (6.7)	8 (9.1)
Alanine aminotransferase increased	0	3 (20.0)	0	3 (7.0)	0	0	7 (8.0)
Aspartate aminotransferase increased	0	3 (20.0)	0	3 (7.0)	0	0	7 (8.0)
Dyspepsia	0	1 (6.7)	1 (7.1)	2 (4.7)	0	1 (6.7)	6 (6.8)
Dysgeusia	0	1 (6.7)	1 (7.1)	2 (4.7)	1 (5.3)	1 (6.7)	5 (5.7)
Lipase increased	1 (7.1)	2 (13.3)	0	3 (7.0)	1 (5.3)	0	5 (5.7)
Abdominal pain	0	0	0	0	0	0	4 (4.5)
Hot flush	0	0	0	0	0	0	4 (4.5)
Insomnia	0	0	2 (14.3)	2 (4.7)	0	2 (13.3)	4 (4.5)
Platelet count decreased	1 (7.1)	0	0	1 (2.3)	1 (5.3)	0	4 (4.5)
Asthenia	0	0	1 (7.1)	1 (2.3)	0	1 (6.7)	3 (3.4)
Flushing	0	1 (6.7)	1 (7.1)	2 (4.7)	0	1 (6.7)	3 (3.4)
Headache	0	0	2 (14.3)	2 (4.7)	0	2 (13.3)	3 (3.4)
Neutrophil count decreased	0	0	2 (14.3)	2 (4.7)	0	2 (13.3)	3 (3.4)
Peripheral sensory neuropathy	0	0	2 (14.3)	2 (4.7)	0	2 (13.3)	3 (3.4)
Pruritus	1 (7.1)	0	0	1 (2.3)	1 (5.3)	0	3 (3.4)
Rash maculo-papular	0	0	1 (7.1)	1 (2.3)	0	1 (6.7)	3 (3.4)
Abnormal faeces	0	0	2 (14.3)	2 (4.7)	0	2 (13.3)	2 (2.3)

AE=adverse event; MedDRA=Medical Dictionary for Regulatory Activities; PT=MedDRA preferred term; TEAE=treatment-emergent adverse event.
Note: Version 28.0 of MedDRA was used to code AEs. A participant reporting the same AE multiple times will be counted once within each PT. The PT will first be sorted by descending frequency (number of participants in each PT in the total column), then alphabetically if ties exist.

Table 14.3.2.4.1
Treatment-Emergent Adverse Events Related to Study Treatment by Preferred Term – Part 1a: Dose Escalation + Backfill
Safety Evaluable Set

	Dose Escalation						
Preferred Term	150mg QD (N=5)	150mg BID (N=4)	300mg BID (N=10)	600mg BID (N=14)	1000mg BID (N=11)	600mg TID (N=1)	Total (N=45)
Amylase increased	0	0	0	1 (7.1)	0	0	1 (2.2)
Anal incontinence	0	0	0	2 (14.3)	0	0	2 (4.4)
Arthralgia	0	0	0	1 (7.1)	0	0	1 (2.2)
Blood alkaline phosphatase increased	0	0	0	1 (7.1)	0	0	1 (2.2)
Blood creatine phosphokinase increased	0	0	1 (10.0)	1 (7.1)	0	0	2 (4.4)
Blood creatinine increased	0	1 (25.0)	0	0	1 (9.1)	0	2 (4.4)
Constipation	0	0	1 (10.0)	0	0	0	1 (2.2)
Dizziness	0	0	0	0	1 (9.1)	0	1 (2.2)
Gastrointestinal disorder	0	0	0	1 (7.1)	1 (9.1)	0	2 (4.4)
Lymphopenia	0	0	0	0	0	0	0
Neutropenia	0	0	0	0	0	0	0
Paraesthesia	0	0	0	0	1 (9.1)	0	1 (2.2)
Pyrexia	0	1 (25.0)	0	1 (7.1)	0	0	2 (4.4)
Rash	0	0	0	1 (7.1)	0	0	1 (2.2)
White blood cell count decreased	0	0	0	1 (7.1)	0	0	1 (2.2)
Abdominal distension	0	0	0	0	0	0	0
Abdominal pain upper	0	0	0	0	0	0	0
Anxiety	0	0	0	0	0	0	0
Blood bilirubin increased	0	0	0	0	0	0	0
Breath odour	0	0	0	0	0	0	0
Chills	0	0	0	0	1 (9.1)	0	1 (2.2)
Dehydration	0	0	0	0	1 (9.1)	0	1 (2.2)
Depressed mood	0	0	1 (10.0)	0	0	0	1 (2.2)
Dry mouth	0	0	0	0	0	0	0
Dysphonia	0	0	0	0	1 (9.1)	0	1 (2.2)
Dyspnoea	0	0	0	1 (7.1)	0	0	1 (2.2)

AE=adverse event; MedDRA=Medical Dictionary for Regulatory Activities; PT=MedDRA preferred term; TEAE=treatment-emergent adverse event.
Note: Version 28.0 of MedDRA was used to code AEs. A participant reporting the same AE multiple times will be counted once within each PT. The PT will first be sorted by descending frequency (number of participants in each PT in the total column), then alphabetically if ties exist.

Table 14.3.2.4.1
Treatment-Emergent Adverse Events Related to Study Treatment by Preferred Term – Part 1a: Dose Escalation + Backfill
Safety Evaluable Set

	Backfill				Dose Escalation + Backfill		Total
Preferred Term	150mg QD (N=14)	200mg TID (N=15)	600mg TID (N=14)	Total (N=43)	150mg QD (N=19)	600mg TID (N=15)	(N=88)
Amylase increased	1 (7.1)	0	0	1 (2.3)	1 (5.3)	0	2 (2.3)
Anal incontinence	0	0	0	0	0	0	2 (2.3)
Arthralgia	0	0	1 (7.1)	1 (2.3)	0	1 (6.7)	2 (2.3)
Blood alkaline phosphatase increased	0	1 (6.7)	0	1 (2.3)	0	0	2 (2.3)
Blood creatine phosphokinase increased	0	0	0	0	0	0	2 (2.3)
Blood creatinine increased	0	0	0	0	0	0	2 (2.3)
Constipation	0	0	1 (7.1)	1 (2.3)	0	1 (6.7)	2 (2.3)
Dizziness	1 (7.1)	0	0	1 (2.3)	1 (5.3)	0	2 (2.3)
Gastrointestinal disorder	0	0	0	0	0	0	2 (2.3)
Lymphopenia	0	2 (13.3)	0	2 (4.7)	0	0	2 (2.3)
Neutropenia	0	1 (6.7)	1 (7.1)	2 (4.7)	0	1 (6.7)	2 (2.3)
Paraesthesia	0	0	1 (7.1)	1 (2.3)	0	1 (6.7)	2 (2.3)
Pyrexia	0	0	0	0	0	0	2 (2.3)
Rash	0	0	1 (7.1)	1 (2.3)	0	1 (6.7)	2 (2.3)
White blood cell count decreased	0	0	1 (7.1)	1 (2.3)	0	1 (6.7)	2 (2.3)
Abdominal distension	0	1 (6.7)	0	1 (2.3)	0	0	1 (1.1)
Abdominal pain upper	0	1 (6.7)	0	1 (2.3)	0	0	1 (1.1)
Anxiety	0	0	1 (7.1)	1 (2.3)	0	1 (6.7)	1 (1.1)
Blood bilirubin increased	0	1 (6.7)	0	1 (2.3)	0	0	1 (1.1)
Breath odour	0	0	1 (7.1)	1 (2.3)	0	1 (6.7)	1 (1.1)
Chills	0	0	0	0	0	0	1 (1.1)
Dehydration	0	0	0	0	0	0	1 (1.1)
Depressed mood	0	0	0	0	0	0	1 (1.1)
Dry mouth	0	0	1 (7.1)	1 (2.3)	0	1 (6.7)	1 (1.1)
Dysphonia	0	0	0	0	0	0	1 (1.1)
Dyspnoea	0	0	0	0	0	0	1 (1.1)

AE=adverse event; MedDRA=Medical Dictionary for Regulatory Activities; PT=MedDRA preferred term; TEAE=treatment-emergent adverse event.
Note: Version 28.0 of MedDRA was used to code AEs. A participant reporting the same AE multiple times will be counted once within each PT. The PT will first be sorted by descending frequency (number of participants in each PT in the total column), then alphabetically if ties exist.

Table 14.3.2.4.1
Treatment-Emergent Adverse Events Related to Study Treatment by Preferred Term – Part 1a: Dose Escalation + Backfill
Safety Evaluable Set

	Dose Escalation						
Preferred Term	150mg QD (N=5)	150mg BID (N=4)	300mg BID (N=10)	600mg BID (N=14)	1000mg BID (N=11)	600mg TID (N=1)	Total (N=45)
Electrocardiogram QT prolonged	0	0	0	0	0	0	0
Epigastric discomfort	0	0	0	1 (7.1)	0	0	1 (2.2)
Face oedema	0	0	0	0	0	0	0
Febrile neutropenia	0	0	0	0	0	0	0
Haematuria	0	0	0	0	0	0	0
Hypercholesterolaemia	0	0	0	1 (7.1)	0	0	1 (2.2)
Hyperhidrosis	0	0	0	0	0	0	0
Hypoglycaemia	0	0	0	1 (7.1)	0	0	1 (2.2)
Hypokalaemia	0	0	0	0	0	0	0
Lymphocyte count decreased	0	0	0	0	0	0	0
Musculoskeletal chest pain	0	0	0	0	1 (9.1)	0	1 (2.2)
Nail disorder	0	0	0	0	0	0	0
Neuropathy peripheral	0	0	0	1 (7.1)	0	0	1 (2.2)
Urticaria	0	0	0	1 (7.1)	0	0	1 (2.2)
Weight decreased	0	0	0	0	0	0	0

AE=adverse event; MedDRA=Medical Dictionary for Regulatory Activities; PT=MedDRA preferred term; TEAE=treatment-emergent adverse event.
Note: Version 28.0 of MedDRA was used to code AEs. A participant reporting the same AE multiple times will be counted once within each PT. The PT will first be sorted by descending frequency (number of participants in each PT in the total column), then alphabetically if ties exist.

P:\Vividion\VVD-133214-01\Biostats\Programs\TLFs\t-aept.sas 13NOV2025 13:17

Table 14.3.2.4.1
Treatment-Emergent Adverse Events Related to Study Treatment by Preferred Term – Part 1a: Dose Escalation + Backfill
Safety Evaluable Set

	Backfill				Dose Escalation + Backfill		Total
Preferred Term	150mg QD (N=14)	200mg TID (N=15)	600mg TID (N=14)	Total (N=43)	150mg QD (N=19)	600mg TID (N=15)	(N=88)
Electrocardiogram QT prolonged	0	1 (6.7)	0	1 (2.3)	0	0	1 (1.1)
Epigastric discomfort	0	0	0	0	0	0	1 (1.1)
Face oedema	1 (7.1)	0	0	1 (2.3)	1 (5.3)	0	1 (1.1)
Febrile neutropenia	0	0	1 (7.1)	1 (2.3)	0	1 (6.7)	1 (1.1)
Haematuria	1 (7.1)	0	0	1 (2.3)	1 (5.3)	0	1 (1.1)
Hypercholesterolaemia	0	0	0	0	0	0	1 (1.1)
Hyperhidrosis	0	0	1 (7.1)	1 (2.3)	0	1 (6.7)	1 (1.1)
Hypoglycaemia	0	0	0	0	0	0	1 (1.1)
Hypokalaemia	0	0	1 (7.1)	1 (2.3)	0	1 (6.7)	1 (1.1)
Lymphocyte count decreased	1 (7.1)	0	0	1 (2.3)	1 (5.3)	0	1 (1.1)
Musculoskeletal chest pain	0	0	0	0	0	0	1 (1.1)
Nail disorder	0	0	1 (7.1)	1 (2.3)	0	1 (6.7)	1 (1.1)
Neuropathy peripheral	0	0	0	0	0	0	1 (1.1)
Urticaria	0	0	0	0	0	0	1 (1.1)
Weight decreased	1 (7.1)	0	0	1 (2.3)	1 (5.3)	0	1 (1.1)

AE=adverse event; MedDRA=Medical Dictionary for Regulatory Activities; PT=MedDRA preferred term; TEAE=treatment-emergent adverse event.
Note: Version 28.0 of MedDRA was used to code AEs. A participant reporting the same AE multiple times will be counted once within each PT. The PT will first be sorted by descending frequency (number of participants in each PT in the total column), then alphabetically if ties exist.

PROTOCOL

**TITLE:**	**A PHASE I, OPEN-LABEL STUDY TO EVALUATE THE SAFETY, TOLERABILITY, PHARMACOKINETICS, PHARMACODYNAMICS, AND ANTI-TUMOR ACTIVITY OF RO7589831** ***AS MONOTHERAPY AND IN COMBINATION WITH PEMBROLIZUMAB*** **IN PARTICIPANTS WITH ADVANCED SOLID TUMORS HARBORING MICROSATELLITE INSTABILITY (MSI) AND/OR DEFICIENT MISMATCH REPAIR (DMMR)**
**PROTOCOL NUMBER:**	BP44474
**VERSION:**	*3*
**REGULATORY AGENCY IDENTIFIER NUMBERS:**	**IND Number**: 164294<br>**EU Trial Number**: 2023-503170-20-01<br>***CIV ID****: To be determined*
**TEST PRODUCT:**	RO7589831, *pembrolizumab*
**SPONSOR:**	F. Hoffmann-La Roche Ltd
**DATE FINAL:**	Version 1: 24 March 2023<br>Version 2: *20 June 2023*
**DATE AMENDED**	*Version 3: See electronic signature and date stamp on the final page of this document*

CONFIDENTIALITY STATEMENT

The information contained in this document, especially any unpublished data, is the property of F. Hoffmann-La Roche Ltd (or under its control) and therefore, is provided to you in confidence as an Investigator, potential Investigator, or consultant, for review by you, your staff, and an applicable Ethics Committee or Institutional Review Board. It is understood that this information will not be disclosed to others without written authorization from Roche except to the extent necessary to obtain informed consent from persons to whom the drug may be administered.

Use of this document is governed by the terms of use on the first page of this document.

PROTOCOL ACCEPTANCE FORM

**TITLE:**	**A PHASE I, OPEN-LABEL STUDY TO EVALUATE THE SAFETY, TOLERABILITY, PHARMACOKINETICS, PHARMACODYNAMICS, AND ANTI-TUMOR ACTIVITY OF RO7589831** ***AS MONOTHERAPY AND IN COMBINATION WITH PEMBROLIZUMAB*** **IN PARTICIPANTS WITH ADVANCED SOLID TUMORS HARBORING MICROSATELLITE INSTABILITY (MSI) AND/OR DEFICIENT MISMATCH REPAIR (DMMR)**
**PROTOCOL NUMBER:**	BP44474
**VERSION NUMBER:**	*3*
**REGULATORY AGENCY IDENTIFIER NUMBERS:**	**IND Number**: 164294<br>**EU Trial Number**: 2023-503170-20-01<br>***CIV ID***: *To be determined*
**TEST PRODUCT:**	RO7589831, *pembrolizumab*
**SPONSOR:**	F. Hoffmann-La Roche Ltd

I agree to conduct the study in accordance with the current protocol.

____

Principal Investigator's Name (print)

____ __________________

Principal Investigator's Signature Date

Please keep the signed original form in your study files, and return a copy to your local Site Monitor.

Use of this document is governed by the terms of use on the first page of this document.

PROTOCOL AMENDMENT, VERSION 3
RATIONALE

Study BP44474 plans to evaluate the safety, pharmacokinetics, pharmacodynamics and preliminary activity of RO7589831 as monotherapy and in combination with anti-programmed death-1(PD-1)/ programmed death-ligand 1 (PD-L1) therapy in patients with MSI and/or solid cancer.

With this amendment the details on the design and conduct of [redacted]

- Pembrolizumab was specified as the combination treatment and replaced PD-1/PD-L1 therapy throughout the document (Section 1, Section 3, Section 4.1.5, Section 6, Section 6.4.1 (including in the study title)
- The Schematic of study design has been [redacted])
- SoA sampling for RO7589831 in combination with pembrolizumab treatment added (Section 1.3)
- [redacted]
- [redacted]
- [redacted]
- Communication strategy in Section 4.1.6.5, Administrative Structure in Section 4.1.7, [redacted]
- The rationale and benefit-risk for the combination treatment of RO7589831 with pembrolizumab was included (Section 2.3 and 4.2.8, respectively)
- Inclusion criteria were updated to include information regarding [redacted]
- Additional Exclusion criteria [redacted]
- [redacted]
- Medications given with precaution due to effects related to pembrolizumab were included (Section 1.1 and Section 6.5.2.3)
- [redacted]
- [redacted]

Use of this document is governed by the terms of use on the first page of this document.

- [redacted]
- [redacted]
- [redacted]
- [redacted]
- [redacted]
- [redacted]
- Pembrolizumab was added to the list of investigational medicinal product and non-investigational medicinal product designations for use in European Economic Area and United Kingdom (Appendix 6)

Additional changes to the protocol are summarized below:

- The protocol has been aligned with the IB v2.0 where the genotoxicity potential risk, which was previously added based on the positive results from an in vitro Ames assay with short-lived metabolite (RO7589830) and a positive in vitro micronucleus test with RO7589831, has been removed, supported by the negative in vivo genotoxicity results. The corresponding changes in the protocol are reflected in the Benefit -Risk assessment Section 2.3 and in the contraception requirements in the eligibility criteria Section 5.1
- Small modifications to the PK collection timepoints have been made in order to better define the absorption phase of the PK profile. These changes have no impact on the number of samples collected or blood volume collected (Section 1.3).
- The RBR specific samples are no longer required to be collected because the sample derivates can be obtained from existing samples (Section 1.3 and 8.8.2)
- The collection of urine (PK) samples on Day 1 has been included to enable renal clearance assessment (Section 1.3). The Rationale for Urine Collection and Analysis has also been added (Section 4.2.10 and Section 4.2.5)
- [redacted] dosing is now explicitly defined as an option throughout the protocol. Minor modifications have been made to the SoAs and considerations for potential comedications that are sensitive P-GP substrates with narrow therapeutic index have been added in case [redacted] dosing would be implemented (Section 1.3 and throughout the protocol as applicable)
- Hospitalization after Cycle 1 Day 1 dose has been made optional to be implemented at Investigator discretion to allow completion of the visit and sampling scheduled on Cycle 1 Day 1 (Section 1.3)

Use of this document is governed by the terms of use on the first page of this document.

- Clarification that the ICF can be signed by the patient before the 28 days screening window (Section 1.3)
- To minimize the loss of relevant pre-dose and post dose PK samples and assessment on Cycle 1 Day 8 visit day, the following were added in Section 1.3:
    - Recommendation to the site to contact the patient on the day before the clinic visit as reminder to ensure the morning dose of RO7589831 is held on the clinic visit Day
    - Possibility to reschedule the Cycle 1 Day 8 visit and all associated assessments the day after in case patient does not hold morning dose until clinic visit day as scheduled
- Clarification that the tumor assessment visit where radiographic disease progression has been documented can be used as the treatment discontinuation visit (Section 1.3)
- The dose limiting toxicities criteria Section 4.1.6.3 has been updated to add a clarification to the dose-limiting toxicities criteria exception for pancreatitis and removal of Alopecia Grade 3 (because not applicable per CTCAE criteria)
- Dose escalation decision criteria for Part I was updated to allow patients to undergo intra-patient dose escalation if the patient experiences disease progression during study treatment. This option is only available if the Investigator deems the patient might benefit from higher dose of RO7589831 and the benefit/risk of this option has been thoroughly discussed with the patient (Section 4.1.1)
- Clarifications were added to the discontinuation for study treatment for Part I [redacted] (Section 7.1)
- Rationale for biomarker assessment was updated to include information regarding the assessment of MSI tumor status as required by local regulations (Section 4.2.2, Section 8.6)
- [redacted]
- The proposed maximum daily RO7589831 dose has been raised from [redacted] (Section 4.3) and the modified continual reassessment method escalation with overdose control design description was updated to account for the change in Max dose (Appendix 12)
- Inclusion criteria have been updated to 1) clarify that for Backfill cohorts the same tumor lesion should be biopsied if possible to avoid introduction of heterogeneity related to site of metastasis; 2) change the calculated creatinine clearance threshold from 60 to 50 mL/min (for rationale please refer to separate memo) (Section 5.1).
- Exclusion criteria have been updated to 1) clarify that patients with pulmonary embolism within 6 months from enrollment in the study may be eligible if event is considered resolved in the opinion of the Investigator and per local guidelines; 2) clarify that patients with Lynch syndrome with multiple tumors are not excluded (Section 5.2).

Use of this document is governed by the terms of use on the first page of this document.

- Clarification on the administration of RO7589831 and allowed time window has been added in Section 6.4.
- Clarification on the continuation of tumor assessments in participants who discontinue study treatment in the absence of radiographic disease progression has been added (Section 8.1.1)
- The time point for the first on-treatment [18F]-FDG PET scan has been modified to decouple the scan window from the on-treatment biopsy time point and keep the window more consistent allowing for better cross-cohort comparisons (Section 8.1.2)
- Information regarding the management of adverse events was updated (Section 8.3.9)
- Information regarding collection and reporting of medical device adverse events was added as needed per local regulations (Appendix 1, Appendix 2)
- The list of clinical laboratory test was updated (Appendix 4)
- Contraceptive and barrier methods guidance was updated (Appendix 5)

Additional minor changes have been made to improve clarity and consistency Substantial new information appears in *Book Antiqua* italics. This amendment represents cumulative changes to the original protocol.

Use of this document is governed by the terms of use on the first page of this document.

TABLE OF CONTENTS

PROTOCOL ACCEPTANCE FORM 2

1. PROTOCOL SUMMARY 14
   1.1 Synopsis 14
   1.2 Schematic of Study Design 18
   1.3 Schedule of Activities 20

2. INTRODUCTION 34
   2.1 Study Rationale 34
   2.2 Background 35
   2.3 Benefit–Risk Assessment 35
   2.3.1 *Additional Considerations on Benefit-risk for the combination with pembrolizumab* 36
   2.3.2 Additional Considerations on Benefit-risk in the Setting of COVID-19 Pandemic 37

3. OBJECTIVES AND ENDPOINTS 38

4. STUDY DESIGN 40
   4.1 Overall Design 40
   4.1.1 Part I Dose Escalation and Backfill Cohorts 41
   [redacted] 42
   4.1.3 Assessment of Food Effect 42
   [redacted] 42
   43
   4.1.6 Dose Escalation 44
   4.1.6.1 Dose-Escalation Decision Criteria *for Part I* 44
   [redacted] 45
   4.1.6.3 Dose-Limiting Toxicities 46
   4.1.6.4 Stopping Rules Criteria 47
   4.1.6.5 Communication Strategy 48
   4.1.7 Administrative Structure 49
   4.2 Scientific Rationale for Study Design 49
   4.2.1 Rationale for Study Population 49
   4.2.2 Rationale for Biomarker Assessments 50

Use of this document is governed by the terms of use on the first page of this document.

4.2.3 Rationale for Tumor Biopsy and Backfill Cohorts 51
4.2.4 Rationale for [^{18}F] FDG-PET Imaging 53
4.2.5 Rationale for Pharmacokinetic Assessments 53
4.2.6 Rationale for Food Effect Assessment.................................... 54
4.2.7 Rationale for Central ECG Reading....................................... 54
[redacted] 55
4.2.9 Rationale for Drug-Drug Interaction assessments 56
4.2.10 *Rationale for Urine Collection and Analysis* 56
4.3 Justification for Dose 57
4.4 Duration of Participation 59
4.5 End of Study Definition 59

5. STUDY POPULATION 59
5.1 Inclusion Criteria 60
5.2 Exclusion Criteria 63
5.3 Lifestyle Considerations... 67
5.4 Meals and Dietary Restrictions 67
5.4.1 For Participants enrolled for Food Effect assessment 67
5.4.2 Alcohol 68
5.5 Recruitment Procedures 68
5.6 Screen Failures... 68

6. TREATMENTS 69
6.1 Treatments Administered... 69
6.2 Preparation, Handling, Storage AND Accountability 70
6.3 Measures to Minimize Bias: Randomization and Blinding 71
6.3.1 Method of Treatment Assignment.. 71
6.3.2 Blinding 72
6.4 Treatment *administration and* Compliance 72
6.4.1 *Administration of Pembrolizumab* 73
6.5 Concomitant Therapy 74
6.5.1 Rescue Medicine 74
6.5.2 Permitted Therapy 74
6.5.2.1 Medications Given with Precaution or with a Time Window Restriction.. 75

Use of this document is governed by the terms of use on the first page of this document.

6.5.2.2 Administration of Vaccines Including SARS-CoV-2 (COVID-19) Vaccines ... 75
6.5.2.3 *Medications Given with Precaution due to Effects Related to Pembrolizumab* ... 76
6.5.3 Prohibited Therapy ... 77
6.6 [redacted] ... 78
6.6.1 Temporary Interruption ... 79
[redacted] ... 79
6.7 Treatment after the End of the Study ... 80
7. DISCONTINUATION OF STUDY, STUDY TREATMENT, AND PARTICIPANT DISCONTINUATION/WITHDRAWAL ... 80
7.1 Discontinuation of Study Treatment ... 80
7.1.1 *For Part I* [redacted] ... 80
[redacted] ... 81
[redacted] ... 81
7.1.3 Participants Replacement ... 82
7.2 Participant Discontinuation OR Withdrawal from the Study ... 82
7.3 Lost to Follow-Up ... 83
8. STUDY ASSESSMENTS AND PROCEDURES ... 83
8.1 Efficacy Assessments ... 84
8.1.1 Tumor and Response Evaluations ... 84
8.1.2 [^{18}F] FDG-PET Imaging ... 85
8.1.3 Serologic Tumor Markers ... 86
8.2 Safety Assessments ... 86
8.2.1 Physical Examinations ... 86
8.2.2 Vital Signs ... 87
8.2.3 Electrocardiograms ... 87
8.2.4 Clinical Safety Laboratory Assessments ... 88
8.2.5 Eastern Cooperative Oncology Group Performance Status ... 89
8.2.6 Clinical Outcome Assessments ... 89
8.2.7 Medical History and Demographic Data ... 91
8.3 Adverse Events and Serious Adverse Events ... 92

Use of this document is governed by the terms of use on the first page of this document.

8.3.1 Period and Frequency for Collecting Adverse Event and Serious Adverse Event Information 92
8.3.2 Method of Detecting Adverse Events and Serious Adverse Events.. 93
8.3.3 Follow-up of Adverse Events and Serious Adverse Events 93
8.3.3.1 Investigator Follow-Up 93
8.3.3.2 Sponsor Follow-Up 93
8.3.4 Regulatory Reporting Requirements for Serious Adverse Events.. 93
8.3.4.1 Emergency Medical Contacts 94
8.3.5 Pregnancy.. 94
8.3.6 Adverse Events of Special Interest 94
8.3.7 Dose-Limiting Toxicities (Immediately Reportable to the Sponsor) 95
8.3.8 Disease-Related Events and/or Disease-Related Outcomes Not Qualifying as Adverse Events or Serious Adverse Events.. 95
8.3.9 Management of Adverse Events 95
8.3.9.1 General recommendations for the management of RO7589831-related AEs.. 95
[redacted] 96
8.4 Treatment of Overdose 97
8.4.1 *Treatment of RO7589831 overdose* 97
[redacted]............ 97
8.5 Pharmacokinetics 97
8.6 Biomarkers Analyses 98
8.6.1 Exploratory Markers for PD Analysis and Response Prediction... 99
8.6.2 Endogenous CYP3A Activity Marker Analyses 99
8.6.3 Genetic and Genomic Analyses... 100
8.6.3.1 Clinical Genotyping.. 100
8.6.3.2 Targeted DNA Analysis 100
8.6.3.3 Transcriptome Analysis 101
8.6.4 Epigenetic Analysis... 101
8.6.5 Proteomics and Metabolomics Analyses 101

Use of this document is governed by the terms of use on the first page of this document.

8.6.5.1 Proteome Research ... 101
8.6.5.2 Metabolomic Research ... 102
8.6.6 Additional Analysis on Blood Samples ... 102
8.7 Biomarker Samples ... 102
8.7.1 Mandatory Samples ... 103
8.7.1.1 Archival Tumor Samples ... 103
8.7.1.2 Blood Sampling ... 103
8.7.1.3 Fresh Tumor Biopsies ... 104
8.7.2 Optional Samples ... 105
8.7.2.1 Optional Paired Tumor Biopsies ... 105
8.7.2.2 Optional Tumor Biopsy as Clinically Feasible ... 105
8.8 Samples for Research Biosample Repository ... 105
8.8.1 Overview of the Research Biosample Repository ... 105
8.8.2 Sample Collection ... 106
8.9 Medical Resource Utilization and Health Economics ... 107
8.10 Timing of Study Assessments ... 107
8.10.1 Screening and Pre-treatment Assessments ... 107
8.10.2 Assessments During Treatment ... 108
8.10.3 Assessments at End of Treatment Visit ... 108
8.10.4 Follow-Up Assessments ... 108
8.10.5 Assessments at Unscheduled Visits ... 109
8.10.6 Exceptional Measures During COVID-19 Pandemic ... 109

9. STATISTICAL CONSIDERATIONS ... 109
9.1 Statistical Hypotheses ... 109
9.2 Sample Size Determination ... 109
9.3 Analysis Sets ... 111
9.4 Statistical Analyses ... 111
9.4.1 Demographics and Baseline Characteristics ... 111
9.4.2 Efficacy Analyses ... 112
9.4.3 Safety Analyses ... 113
9.4.4 Pharmacokinetic Analyses ... 113
9.4.5 Pharmacodynamic Analyses ... 114
9.4.6 Pharmacokinetic/ Pharmacodynamic Relationships ... 114

Use of this document is governed by the terms of use on the first page of this document.

9.4.7 Other Analyses ... 114
9.4.7.1 Endogenous CYP3A Activity Marker Analysis ... 114
9.4.7.2 [18F] FDG-PET Imaging ... 115
9.4.7.3 Analyses of EORTC QLQ-F17 [redacted] ... 115
9.4.7.4 Analyses of PRO-CTCAE and EORTC-IL46 ... 115
9.5 Interim Analyses ... 116
9.6 Summaries of Conduct of Study ... 116

10. REFERENCES ... 117

11. SUPPORTING DOCUMENTATION AND OPERATIONAL CONSIDERATIONS ... 123

LIST OF TABLES

Table 1 Schedule of Activities ... 21
Table 2 Pharmacokinetic, Biomarker, and Electrocardiogram Schedule for Participants not Undergoing Paired Tumor Biopsy Collection *(Part I* [redacted] ... 24
Table 3 Pharmacokinetic, Biomarker, and Electrocardiogram Schedule for Participants Undergoing Paired Tumor Biopsy Collection and On-treatment Tumor Biopsy Collection Post-dosing *(Part I* [redacted] ... 26
Table 4 Pharmacokinetic, Biomarker, and Electrocardiogram Schedule for Participants Undergoing Paired Tumor Biopsy Collection and Time of On-treatment Tumor Biopsy is Changed to Pre-dosing *(Part I* [redacted] ... 28
[redacted] ... 30
... 32
Table 7 Objectives and Endpoints ... 38
Table 8 Study Parts and Participants Population ... 40
Table 9 Key Study Data Supporting Starting Dose ... 58
Table 10 Simulated Steady State PK Parameters Following Oral Administration of Different [redacted] Doses of RO7589831 ... 58
Table 11 Summary of Treatments Administered ... 69
Table 12 General recommendations for the management of RO7589831-related AEs ... 96
Table 13 Analysis Sets ... 111
Table 14 Efficacy Statistical Analysis Methods ... 113

Use of this document is governed by the terms of use on the first page of this document.

Table 15 Safety Statistical Analysis Methods 113

LIST OF FIGURES

Figure 1 Overview of Study Design 19

LIST OF APPENDICES

Appendix 1 Regulatory, Ethical, and Study Oversight Considerations........ 124
Appendix 2 Adverse Events: Definitions and Procedures for Evaluating, Follow-up and Reporting 134
Appendix 3 Procedures for Recording Adverse Events 146
Appendix 4 Clinical Laboratory Tests 151
Appendix 5 Contraceptive and Barrier Methods Guidance 154
Appendix 6 Investigational Medicinal Product and Non-Investigational Medicinal Product Designations for Use in United Kingdom 159
Appendix 7 Guidance for Fluoro-deoxy-glucose Positron Emission Tomography 160
Appendix 8 Response Evaluation Criteria in Solid Tumors, Version 1.1: Excerpt from Original Publication.......................... 162
Appendix 9 European Organization for Research and Treatment of Cancer- Function 17 173
Appendix 10 Patient-Reported Outcomes Common Terminology Criteria for Adverse Events.. 174
Appendix 11 European Organisation for Research and Treatment of Cancer Item List 46 178
[redacted]... 179
Appendix 13 Modified Continual Reassessment Method Escalation with Overdose Control Design Description 180
Appendix 14 Drugs that Should be Administered with Caution and Monitoring, Should be Administered with Time Separation or are Prohibited 187
Appendix 15 Composition of High Fat Meal 189
Appendix 16 *Preexisting Autoimmune Diseases and Immune Deficiencies for Eligibility* 190
Appendix 17 *Anaphylaxis Precautions* 191
Appendix 18 Abbreviations 192

Use of this document is governed by the terms of use on the first page of this document.

1. PROTOCOL SUMMARY

1.1 SYNOPSIS

**PROTOCOL TITLE:**	**A PHASE I, OPEN-LABEL STUDY TO EVALUATE THE SAFETY, TOLERABILITY, PHARMACOKINETICS, PHARMACODYNAMICS, AND ANTI-TUMOR ACTIVITY OF RO7589831** ***AS MONOTHERAPY AND IN COMBINATION WITH PEMBROLIZUMAB*** **IN PARTICIPANTS WITH ADVANCED SOLID TUMORS HARBORING MICROSATELLITE INSTABILITY (MSI) AND/OR DEFICIENT MISMATCH REPAIR (DMMR)**
**SHORT TITLE**	**A PHASE I STUDY OF RO7589831 IN PARTICIPANTS WITH ADVANCED SOLID TUMORS**
**PROTOCOL NUMBER:**	BP44474
**VERSION:**	*3*
**REGULATORY AGENCY IDENTIFIER NUMBERS:**	**IND Number**: 164294<br>**EU Trial Number**: 2023-503170-20-01<br>***CIV ID**: To be determined*
**TEST PRODUCT:**	RO7589831, *pembrolizumab*
**PHASE:**	I

RATIONALE

Deficient mismatch repair (dMMR) is caused by the inactivation of genes of the DNA repair machinery that leads to DNA mismatches in microsatellites and variations in the length of tandem nucleotide repeats across the genome, known as microsatellite instability (MSI).

The prevalence of MSI and/or dMMR has been reported to be approximately 30% for endometrial cancer, 20% for colon or gastric cancer, and less than 5% for most other tumor types, with a higher prevalence often found in Stage I to III compared to Stage IV disease.

Treatment standards for MSI and/or dMMR tumors have shifted in recent years with the approval of several checkpoint inhibitors. While responses to immune checkpoint blockade in MSI and/or dMMR solid tumors are often durable, 40% to 70% of such cancers do not respond to immune checkpoint blockade or develop secondary resistance, and the use of checkpoint inhibitors can be limited by toxicity. Thus, novel therapies are needed for patients with MSI and/or dMMR tumors.

Werner syndrome (WRN) helicase is a bi-functional enzyme belonging to the RecQ family that is required for DNA replication and DNA repair to ensure genomic stability and has been identified as a novel synthetic lethal target in MSI cancer.

Depletion of WRN helicase induces double-stranded DNA breaks, promoting apoptosis and cell cycle arrest selectively in MSI and not in microsatellite stable (MSS) preclinical models, indicating WRN helicase as a promising drug target for MSI tumors.

The agent investigated under this protocol, RO7589831, is a novel covalent, irreversible inhibitor of WRN helicase that causes DNA damage accumulation and has selective antitumor activity in MSI cell lines and in vivo mouse models, including patient-derived models.

This entry-into-human (EiH) study will assess the safety, pharmacokinetics (PK), pharmacodynamics (PD) and preliminary clinical anti-tumor activity of RO7589831 monotherapy in participants with MSI and/or dMMR advanced solid tumors *and in combination with*

Use of this document is governed by the terms of use on the first page of this document.

pembrolizumab in participants with MSI and/or dMMR advanced colorectal adenocarcinoma (CRC).

OBJECTIVES AND ENDPOINTS

Objectives	Endpoints
**Primary**	
• To evaluate the safety and tolerability of RO7589831 as monotherapy *and in combination with pembrolizumab*	• Incidence, nature, and severity of adverse events (AEs) graded according to the National Cancer Institute (NCI) Common Terminology Criteria for Adverse Events (CTCAE) v5
• To determine the maximum tolerated dose (MTD) and/or the recommended dose for expansion (RDE) or the recommended Phase 2 dose (RP2D) for RO7589831 monotherapy *and in combination with pembrolizumab*	• Occurrence of dose-limiting toxicities (DLTs), nature and frequency of AEs, anti-tumor activity endpoints and pharmacokinetics (PK)
**Secondary**	
• To characterize the pharmacokinetics (PK) profile of RO7589831 as monotherapy *and in combination with pembrolizumab*	• PK profiles and parameters derived for RO7589831 including but not limited to: C_{max}, T_{max}, C_{min}, CL/*F*, $T_{1/2}$, V_{ss}, and AUC for various time intervals following single and multiple doses of RO7589831
• To evaluate the anti-tumor activity of RO7589831 as monotherapy *and in combination with pembrolizumab*	• Objective response rate (ORR), disease control rate (DCR), duration of response (DOR), progression-free survival (PFS) according to Response Evaluation Criteria in Solid Tumors (RECIST) v1.1 as assessed by the Investigators<br>• Overall survival (OS)

Objectives related to [redacted] are described in the protocol body text.

OVERALL DESIGN AND STUDY POPULATION

This is a first in human Phase I, open-label, multicenter, dose-escalation and dose expansion study to evaluate the safety, tolerability, pharmacokinetics (PK), pharmacodynamics (PD) and preliminary anti-tumor activity of RO7589831 monotherapy in participants with MSI and/or dMMR advanced solid tumors *and in combination with pembrolizumab in participants with MSI and/or dMMR advanced colorectal adenocarcinoma (CRC)*.

The study population consists of participants with:

- Any MSI and/or dMMR advanced solid tumors (Part I [redacted]
- [redacted]

The study consists of the following parts:

Study Part	Purpose	Participants Population
**Part I**	Dose escalation (and backfill cohorts) to determine the maximum tolerated dose (MTD) and/or the recommended dose(s) for expansion (RDE) *or recommended Phase 2 dose (RP2D)* for RO7589831 monotherapy	MSI and/or dMMR advanced solid tumors

Use of this document is governed by the terms of use on the first page of this document.

Study Part	Purpose	Participants Population
[redacted]		

A Dose Decision Team (DDT), which includes Sponsor representatives and Investigators, will review emerging study data and make decisions regarding dose escalation *in Part I* [redacted]

Several key aspects of the study design and study population are summarized below.

**Phase:**	Phase I	**Population Type:**	Patients
**Control Method:**	Not applicable	**Population Diagnosis or Condition:**	Advanced solid tumors
**Interventional Model:**	Sequential	**Population Age:**	≥ 18 years of age
**Test Compound:**	RO7589831<br>*Pembrolizumab* [redacted]	**Site Distribution:**	Multicenter
**Active Comparator:**	Not Applicable	**Study Intervention Assignment Method:**	Open-label
**Number of Arms:**	Up to *8*	**Number of Participants to be Enrolled:**	Approximately *295* (without [redacted])

Use of this document is governed by the terms of use on the first page of this document.

STUDY TREATMENT

The investigational medicinal product*s* (IMP*s*) for this study *are* RO7589831 *and pembrolizumab*. *RO7589831* administration will take place both at the study center under supervision of qualified site staff or self-administered at the participant's home or at a different location. [redacted]

The starting dose of [redacted] *of RO7589831* was determined based on preclinical PK data, toxicokinetic data, toxicology studies and anti-tumor activity in preclinical models.

RO7589831 will be administered orally (po) [redacted] in 3-week cycles. If the [redacted] regimen shows unacceptable toxicity or unfavorable PK characteristics, the dosing schedule may be changed, e.g., [redacted] *or* [redacted] The cohorts with the new dosing schedule will use the total daily dose equivalent proposed by the mCRM EWOC model.

[redacted]

DURATION OF PARTICIPATION

The study duration per participant from screening until safety follow-up visit may be up to approximately [redacted] (not including long-term follow-up [LTFU]).

- Screening: Days -28 to -1.
- Treatment period: continuous treatment *with RO7589831* until disease progression *(unless participants are undergoing intra-participant dose-escalation)*, unacceptable toxicity, participant withdrawal of consent, or death, whichever occurs first. [redacted]
- Safety follow-up visit: within 30 to 37 days after the last RO7589831 dose.
- LTFU: After the safety follow-up visit, participants will be followed up with every 90 (± 14) days after the last RO7589831 dose to assess subsequent anti-cancer therapies, and survival status.

COMMITTEES

No formal third-party data monitoring committee is planned.

A Dose Decision Team (DDT), which includes Sponsor representatives and Investigators, will review emerging study data and make decisions regarding dose escalation in Part I [redacted] medical data review will be performed regularly by an Internal Monitoring Committee (IMC) composed of key Sponsor functional representatives. To ensure the safety of the participant during the study, the IMC will make recommendations regarding study conduct on the basis of trial safety data and emerging benefit-risk, including evaluation of the totality of the data consisting of the available safety, efficacy, PK and PD profile at the time of each interim analysis. For details, see the IMC charter.

CONCOMITANT MEDICATIONS

All treatments that the Investigator considers necessary for a participant's welfare may be administered at the discretion of the Investigator in keeping with the community standards of medical care.

Medications Given with Precaution or with a Time Window Restriction *due to effects related to RO7589831*

RO7589831 was shown to be a moderate cytochrome P450 3A4 (CYP3A) and cytochrome P450 2B6 (CYP2B6) inducer in vitro. Sensitive CYP2B6 and CYP3A substrates (as described in the prescribing information) should be used with precaution, or selection of an alternate concomitant medicinal product should be considered.

Use of this document is governed by the terms of use on the first page of this document.

RO7589831 might inhibit P-glycoprotein (P-gp) in the gastrointestinal tract during its absorption. Comedications known to be sensitive P-gp substrates of narrow therapeutic index should therefore be administered at least 2 hours before or 6 hours after RO7589831 administration to minimize the risk of increased exposure of these comedications. *In the event that* [redacted] *dosing would be implemented, drugs known to be sensitive P-gp substrates of narrow therapeutic index should not be co-administered.*

Chemotherapy (e.g., methotrexate) required for autoimmune disease is to be avoided during the dose-limiting toxicity (DLT) assessment window and caution should be exercised when given outside the DLT window.

In general, non-live vaccines are allowed during the study. It is strongly recommended not to administer non-live vaccine during the DLT observation window, where possible.

[redacted]

Prohibited Therapies

- Investigational therapy (other than protocol-mandated study treatment)
- Live attenuated vaccines
- Concomitant therapy*, besides the ones allowed in the protocol,* intended for the treatment of cancer whether approved by the local Health Authority or experimental, including chemotherapy, radiotherapy, immunotherapy, biologic therapy, herbal therapy, or hormonal therapy with some exceptions (Section 6.5.3)
- Initiation of hematopoietic colony-stimulating factors (CSF) during DLT assessment window
  - After the DLT assessment window has been completed CSFs may be administered per standard clinical practice
- Any moderate or strong inhibitor of CYP3A taken within 7 days prior to first study drug administration and up to the safety follow-up visit.
- Any moderate or strong inducer of CYP3A taken within 14 days prior to first study drug administration and up to the safety follow-up visit.
- Taking any nutrients known to modulate CYP3A activity (e.g., grapefruit juice) within two days prior to first study drug administration and up to the safety follow-up visit.

Participants who require any of these agents might be discontinued from study treatment after discussion with the Medical Monitor.

For information regarding medications that are contraindicated with pembrolizumab, please refer to the local prescribing information.

1.2 SCHEMATIC OF STUDY DESIGN

An overview of the study design is provided in Figure 1.

Use of this document is governed by the terms of use on the first page of this document.

Figure 1 Overview of Study Design

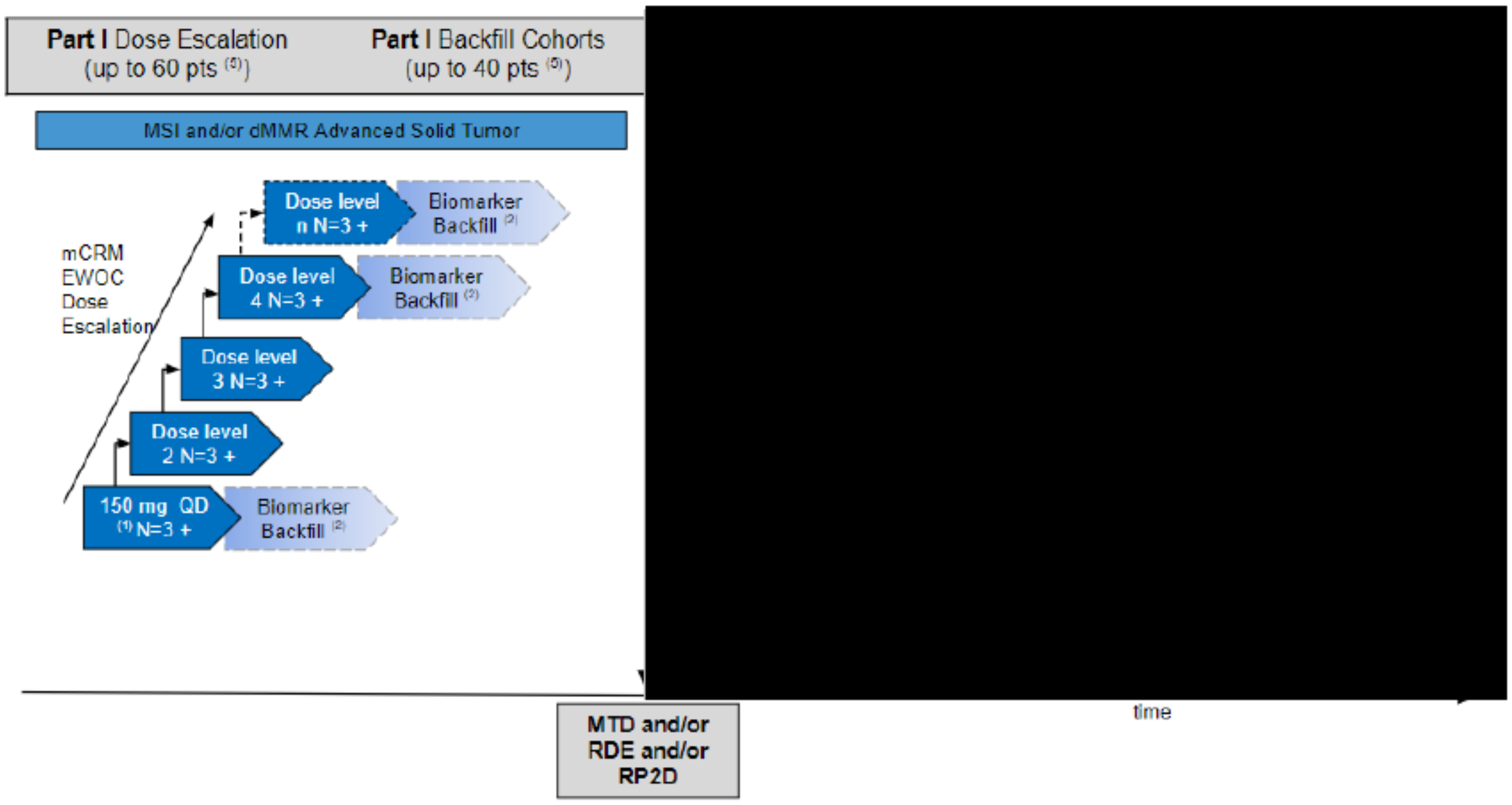

CRC = colorectal adenocarcinoma, DDI = drug-drug interaction, dMMR = deficient mismatch repair, MTD = maximum tolerated dose, mCRM EWOC: modified continual reassessment method with escalation with overdose control, MSI = microsatellite instability, N = number of participants; PD-1: programmed death-1, PDL1: programmed death-ligand 1, RDE = recommended dose for expansion.

1 If the *RO7589831* [redacted] regimen shows unacceptable toxicity or unfavorable PK characteristics, the dosing schedule may be changed, e.g., [redacted] *or* [redacted]

2 The backfill cohorts in the figure are illustrative. *RO7589831 dose* levels for backfill cohorts will be selected based on emerging PK, pharmacodynamics, safety and anti-tumor activity data.

3 Up to two cohorts at different *RO7589831* doses and/or dose schedules may be opened. Option to further expand via protocol amendment if relevant clinical activity is observed.

4 Details on design and participant population will be introduced via a protocol amendment.

5 Based on emerging data, some participants might be allocated within the planned sample size to certain cohorts, [redacted]

Use of this document is governed by the terms of use on the first page of this document.

1.3 SCHEDULE OF ACTIVITIES

The following schedules of activities (SoAs) apply:

Table 1	Main SoA
Table 2	Pharmacokinetic, biomarker, and electrocardiogram (ECG) schedule for participants not undergoing paired tumor biopsy collection
Table 3	Pharmacokinetic, Biomarker, and Electrocardiogram Schedule for Participants Undergoing Paired Tumor Biopsy Collection and On-treatment Tumor Biopsy Collection Post-dosing
Table 4	Pharmacokinetic, biomarker, and ECG schedule for participants undergoing paired tumor biopsy collection and time of on-treatment tumor biopsy is changed to predosing (Section 8.7.1.3)
[redacted]	[redacted]
[redacted]	[redacted]

Use of this document is governed by the terms of use on the first page of this document.

Table 1 Schedule of Activities

Protocol Sections	Each cycle=21 days	Screening	Cycle 1						Cycle 2			Cycle 3 onwards (1)		End of Treatment/Treatment Discontinuation visit	Safety Follow-Up	Long-Term Follow-up	Unscheduled (Safety)(2)
	Study visit window				± 1 day			± 1 day (time window not applicable for participants with FE assessment)		± 1 day	± 1 day	± 1 day	± 1 day			± 14 days	
	Day (=D; relative to study drug administration)	D-28 to D-1	D1	D2 (3)	D5 (only Part I)	D8	D9 (3)	D15	D1	D8	D15	D1	D8 (only C3, Part I)				
	[redacted]		Predose	Predose	Predose	Predose	Predose	Predose	Predose	Predose	Predose	Predose	Predose	within 14 days from last study drug	within 30 to 37 Days after last study drug	Every 90 Days after last study drug, in person or via phone call	
			Hospitalisation (4)														
		Clinical Assessments															
Appendix 1	Informed consent	x *(10)*															
5.1; 5.2	Eligibility	x															
8.2.7	Demography	x															
8.2.7	Medical & cancer history	x															
8.2.3	ECG-12 lead (triplicate)	See Table 2 for participants not undergoing tumor paired biopsy collection; Table 3 for participants undergoing paired biopsy collection (Table 4 if on treatment tumor biopsy is changed to predose); Table 5 for participants undergoing FE assessment; *Table 6 for participants enrolled in Part IV*															
8.2.1	Complete physical examination	x	x			x		x									
8.2.1	Targeted physical examination								x	x	x	x	x	x	x		x
8.2.1	Weight (BMI)	x	x (5)			x			x			x		x	x		x
8.2.1	Height	x															
8.2.5	ECOG performance status	x	x						x			x		x	x		x
8.2.2	Vital signs	x	x	x		x		x	x	x	x	x	x	x	x		x
8.3	Adverse events	x (prior to 1st study drug administration only SAEs related to protocol mandated procedures to be reported)															x
6.5	Previous and concomitant treatments	x														Anti-cancer treatment only	x
8.2.6	PRO-CTCAE (6)		x			x		x	x	x	x	x		x	x		
8.2.6	EORTC IL46 (6)					x		x	x	x	x	x		x	x		
8.2.6	EORTC QLQ-F17 (6)		x			x		x	x	x	x	x		x	x		
[redacted]																	
		RO7589831 administration															
6.1; 6.2; 6.4	RO7589831 oral dispensation		x (Once daily (QD) - [redacted]														
6.4	Study Drug accountability (Patient diary for study drug administration)		Continuous														
[redacted]																	

Use of this document is governed by the terms of use on the first page of this document.

Table 1 Schedule of Activities (cont.)

Protocol Sections	Each cycle=21 days	Screening	Cycle 1						Cycle 2			Cycle 3 onwards (1)		End of Treatment/Treatment Discontinuation visit	Safety Follow-Up	Long-Term Follow-up	Unscheduled (Safety)(2)
	Study visit window				± 1 day			± 1 day (time window not applicable for participants with FE assessment)		± 1 day	± 1 day	± 1 day	± 1 day			± 14 days	
	Day (=D; relative to study drug administration)	D-28 to D-1	D1	D2 (3)	D5 (only Part I)	D8	D9 (3)	D15	D1	D8	D15	D1	D8 (only C3, Part I)				
	Timepoint [redacted]		Predose	Predose	Predose	Predose	Predose	Predose	Predose	Predose	Predose	Predose	Predose	within 14 days from last study drug	within 30 to 37 Days after last study drug	Every 90 Days after last study drug, in person or via phone call	
			Hospitalisation (4)														
		Response Assessment															
8.1.1	Tumor Assessment RECIST v1.1 (CT/MRI of chest, abdomen pelvis and other regions as clinically indicated)	x	Every 6 weeks ± 7 days from C1D1 for the first 48 weeks and every 12 weeks ± 7 days thereafter, until disease progression, initiation of subsequent anti-cancer therapy, withdrawal or death														
8.1.3	Tumor Markers *tested per local clinical practice for the tumor type under investigation in this study (eg CA125, PSA, CEA etc)*	x							x			x		x			
8.1.2	FDG PET *(not applicable for Part IV)*	x							x (up to 5 days before C2D1, *before biopsy if biopsy is scheduled*)			x (up to 5 days before first RECIST assessment)					
		Local Laboratory Assessment															
5.1	MSI and/or dMMR status	x															
Appendix 4	Hematology blood	x (-14 to -1)	x (7)	x	x		x	x	x (7)	x	x	x	x	x	x		x
	Clinical Chemistry blood	x (-14 to -1)	x (7)	x	x		x	x	x (7)	x	x	x	x	x	x		x
	Lipid Panel		x (7)						x (7)			x		x			
	Thyroid function: TSH, free T3, and free T4 (only for Part IV)	x										*x (C5 and every 4 cycles thereafter)*					
	Coagulation blood	x (-14 to -1)	x (7)	x	x		x	x	x (7)	x	x	x	x	x	x		x
	Urine analysis	x (-14 to -1)	x (7)	x	x		x	x	x (7)	x	x	x	x	x	x		x
	Viral serology (HIV, HBV, HCV) blood	x															
	Chemistry Serum for Pregnancy testing (8)	x (-7 to -1)	(x)						(x)			(x)		(x)	(x)		
	Chemistry Urine for Pregnancy testing (9)		x						x			x		x	x	x *(12)*	
		Central Laboratory assessment															
8.7.1.1	Biomarker archival tumor tissue for biomarker analysis	Refer to Table 2 for participants not undergoing tumor paired biopsy collection; Table 3 for participants undergoing paired biopsy collection (or Table 4 if on treatment tumor biopsy schedule is changed to predose); [redacted]															
8.7.1.3; 8.7.2.2; 8.7.2.3	Biomarker fresh tumor tissue(s) for biomarker analysis																
8.7.1.2	Biomarker Blood for response prediction																
8.7.1.2	Biomarker Blood for ctDNA analysis																
8.5	PK plasma for RO7589831																
8.7.1.2	Biomarker blood for Clinical Genotyping analysis																
8.7.1.2	Part I only: Biomarker plasma for CYP induction status endogenous marker analysis																
8.7.1.2	Biomarker plasma for exploratory biomarker analysis																
8.5	*PK urine collection for RO7589831*																

Use of this document is governed by the terms of use on the first page of this document.

AE = adverse event; [redacted] BMI = body-mass index; C = Cycle; CT/MRI = computed tomography/magnetic resonance imaging; CTCAE = Common Terminology Criteria for Adverse Events; ctDNA = circulating tumor deoxyribonucleic acid; CYP = cytochrome P; D = Day; dMMR = deficient mismatch repair; DNA = deoxyribonucleic acid; ECG = electrocardiogram; ECOG = Eastern Cooperative Oncology Group; EORTC-IL46 = European Organisation for Research and Treatment of Cancer item list 46; EORTC QLC-F17 = European Organisation for Research and Treatment of Cancer Quality-of-life Questionnaire-Function 17; ePRO = electronic patient-reported outcome; FDG = fluoro deoxy glucose; FE = food effect; HBV = hepatitis B virus; HCV = hepatitis C virus; HIV = human immunodeficiency virus; MSI = microsatellite instability; PET = positron emission tomography; PRO = patient-reported outcome; [redacted] RBR = Research Biosample Repository; RECIST = Response Evaluation Criteria in Solid Tumors; RNA = ribonucleic acid; SAEs = serious adverse events; [redacted]

1 *In Part I* [redacted]*, f*rom C9, visits frequency can be reduced to once every two cycles (e.g., C9D1, C11D1 etc.). In this case sufficient *RO7589831* drug tablets will have to be dispensed to the participants to last for two cycles.

2 In case of dose-limiting toxicities, Grade ≥ 3 adverse event (AE) leading to dose reduction or dose delay, or clinically significant ECG changes

3 D2 and D9 visits of Cycle 1 may not be required in [redacted] of the study based on emerging data from Part I.

4 *Overnight hospitalization may be offered at Investigator discretion to allow completion of the visit/sampling scheduled on Cycle 1 Day 1.*

5 Within 24 hours prior to RO7589831 administration.

6 Electronic patient reported outcomes (ePROs) will be administered before the participant received any information on disease status, prior to the performance of non-PRO assessments (when possible), and prior to *RO7589831 with or without pembrolizumab* administration and adverse event evaluation. See Section 8.2.6 for further details on how to administer PRO instruments. If PROs are administered and *RO7589831 with or without pembrolizumab* administration is missed for any reason, the PRO is not re-administered and the next assessment comes at the next scheduled assessment.

7 May be obtained up to 72 hours prior to scheduled clinic visit day.

8 Serum pregnancy test is required during screening. After screening, serum pregnancy test is only required if a urine pregnancy test is positive

9 If per local standard only serum pregnancy test is done, the urine pregnancy test may be replaced by serum pregnancy test at all required timepoints.

10 *May be obtained > 28 days before the start of study treatment.*

11 [redacted]

12 [redacted]

Use of this document is governed by the terms of use on the first page of this document.

Table 2 Pharmacokinetic, Biomarker, and Electrocardiogram Schedule for Participants not Undergoing Paired Tumor Biopsy Collection *(Part I* [redacted]

Sample Category				ECG (triplicate)	PK	Biomarker	Biomarker	Biomarker	Biomarker	Biomarker	Part I only Biomarker	Biomarker	*PK*
Sample Type					Plasma	Archival tumor tissue (4)	Fresh tumor tissue	Blood	Blood	Blood	Plasma	Plasma	*Urine collection (9)*
Sample Intended purpose					For RO7589831	For biomarker analysis	for biomarker analysis	For response prediction	For ctDNA analysis	For Clinical Genotyping analysis	For CYP induction status endogenous marker analysis	For exploratory biomarker analysis	*For RO7589831*
Cycle length: 21 days	Day (1)	Scheduled time (H=hours) (2) (3)	Time window										
**Screening**	Day -28 to Day -1			x		x (5)							
Cycle 1 [redacted]	Day 1	**Predose**		x	x			x (within 72 hours from C1D1 dosing)	x (within 72 hours from C1D1 dosing)	x (within 72 hours from C1D1 dosing)	x (within 72 hours from C1D1 dosing)	x	*x*
		0.5 H Postdose	*±10 min*	*x*	*x*								
		**1H Postdose**	±15 min	x	x								
		**2H Postdose**	±15 min	x	x								
		**3H Postdose**	±15 min	x	x								
		**4H Postdose**	±15 min	x	x							x	
		**6H Postdose**	±30 min		x								
		8H Postdose	±30 min	x	x								
		12H Postdose	±30 min	x	***x (not needed if*** [redacted] ***is implemented)***								
	Day 8 *(8)*	**Predose**		x	x						x	x	
		0.5 H Postdose	*±10 min*	*x*	*x*								
		**1H Postdose**	±15 min	x	x								
		**2H Postdose**	±15 min	x	x								
		**3H Postdose**	±15 min	x	x								
		**4H Postdose**	±15 min	x	x							x	
		**6H Postdose**	±30 min		x								
		8H Postdose	±30 min	x	x								
	**Day 15 (only part I)**	**Predose**		x	x						x		
Cycle 2	Day 1	**Predose**		x	x				x		x	x	
		2H Postdose	± 30 min		x								
		4H Postdose	±30 min		x							x	
		8H Postdose	±120 min		x								
	**Day 8 (only Part I)**	Predose		x	x								
	**Day 15 (only Part I)**	Predose		x	x								
Subsequent Cycles (6)	**Day 1**	**Predose**		x	**x**				x (C3, C5 and every 4 cycles thereafter eg C9 etc)		**x**	**x**	
		**2H Postdose**	±15 min		x (C3 and every 2 cycles thereafter eg C5, C7 etc)								
		**4H Postdose**	±30 min		x (C3 and every 2 cycles thereafter eg C5, C7 etc)							x (C3 and every 2 cycles thereafter eg C5, C7 etc)	
At time of progression								**x**	x			**x**	
EOT/Discontinuation Visit				x					x (Not needed if sample was collected at time of progression)			x (Not needed if sample was collected at time of progression)	
If tumor tissue collected per SOC (e.g. surgery) (optional, collection of tumor tissue sample)							x						
Unscheduled Biopsy eg at time of PR, SD or disease progression (optional, if deemed clinically feasible by the Investigator)							x						
**Unscheduled PK for specific events (7)**					x								

Use of this document is governed by the terms of use on the first page of this document.

[redacted] C = Cycle; ctDNA = circulating tumor deoxyribonucleic acid; CYP = cytochrome P; D = Day; DNA = deoxyribonucleic acid; ECG = electrocardiogram; eCRF = electronic case report form; EOT = end of treatment; H = hours; min = minutes; PK = pharmacokinetics; PR = partial response; [redacted] RBR = Research Biosample Repository; RNA = ribonucleic acid; SD = stable disease; SOC = standard of care.

1 For study visit window see Table 1.

2 "Predose" is a sample to be collected shortly before (preferably no more than 2 hours before, unless otherwise stated) the time of drug administration ("Dosing") of RO7589831. RO7589831 should be taken preferably at the same time of each day. Scheduled timepoints refer to times in hours after the first morning dosing of RO7589831. Actual time of PK sample collection needs to be recorded and entered in the eCRF. If a [redacted] dosing schedule is implemented, "predose" and "postdose" refer to the first dosing in the day and the [redacted] sample should be taken prior to the second dose. [redacted]

3 Sampling timepoints might be modified based on emerging data.

4 If archival tumor tissue is not available, fresh tumor biopsy must be obtained.

5 If participants are re-screened, tumor tissue collection does not need to be repeated if it was already collected during previous screening.

6 From C9, visits frequency can be reduced to once every two cycles (e.g., C9D1, C11D1 etc.).

7 Additional PK sample should be collected at an unscheduled visit in case of suspected overdose *RO7589831*, dose-limiting toxicities or adverse events leading to dose reduction, dose interruption or clinically significant ECG changes. The PK samples should be taken as soon as possible (i.e., ideally at the time of the development of such an event).

8 *It is strongly recommended that participants are reminded on the day before the clinic visit to hold the morning dose on the day of the clinic visit to allow accurate PK/PD sampling and ECG measurements. If participant did not hold morning daily dose until the clinic visit, then the PK, PD sampling and associated ECG will not be collected on this day but will be shifted to the next day and the participant will be reminded to hold off the planned morning dose accordingly. All the other visits assessment (see Table 1) will be carried out as per original schedule.*

9 *In selected Part I* [redacted] *cohorts urine may be collected over a period of* [redacted] *if* [redacted] *following administration of RO7589831 on day 1 of the study. A single pool of all urine collected during this time should be made. The total urine volume should be recorded.*

Use of this document is governed by the terms of use on the first page of this document.

Table 3 Pharmacokinetic, Biomarker, and Electrocardiogram Schedule for Participants Undergoing Paired Tumor Biopsy Collection and On-treatment Tumor Biopsy Collection Post-dosing *(Part I* [redacted]

Sample Category				ECG (triplicate)	PK	Biomarker	Biomarker	Biomarker	Biomarker	Biomarker	Part I only Biomarker	Biomarker	*PK*
Sample Type					Plasma	Archival tumor tissue (4)	Fresh tumor tissue	Blood	Blood	Blood	Plasma	Plasma	*Urine collection (10)*
Sample Intended purpose					For RO7589831	For biomarker analysis	For biomarker analysis	For response prediction	For ctDNA analysis	For Clinical Genotyping analysis	For CYP induction status endogenous marker analysis	For exploratory biomarker analysis	*For RO7589831*
Cycle length: 21 days	Day(1)	Scheduled time (H=hours) (2) (3)	Time window										
**Screening**	Day -28 to Day -1			x		x (5)	x (6)						
Cycle 1 [redacted]	Day 1	Predose		x	x			x (within 72 hours from C1D1 dosing)	x (within 72 hours from C1D1 dosing)	x (within 72 hours from C1D1 dosing)	x (within 72 hours from C1D1 dosing)	x	*x*
		0.5 H Postdose	*±10 min*	*x*	*x*								
		1H Postdose	±15 min	x	x								
		2H Postdose	±15 min	x	x								
		3H Postdose	±15 min	x	x								
		4H Postdose	±15 min	x	x							x	
		6H Postdose	±30 min		x								
		8H Postdose	±30 min	x	x								
		12H Postdose	±30 min	x	*x (not needed if* [redacted] *is implemented)*								
	Day 8 *(11)*	Predose		x	x						x	x	
		0.5 H Postdose	*±10 min*	*x*	*x*								
		1H Postdose	±15 min	x	x								
		2H Postdose	±15 min	x	x								
		3H Postdose	±15 min	x	x								
		4H Postdose	±15 min	x	x							x	
		6H Postdose	±30 min		x								
		8H Postdose	±30 min	x	x								
	Day 15 (only part I)	Predose		x	x						x		
Cycle 2	Day 1 or Day 2 or Day 3 (7)<br><br>ECG, blood and plasma samples must be collected on the same day of biopsy eg if biopsy is collected on C2D2 also the ECG and all blood and plasma samples must be collected on C2D2<br><br>NOTE: Dosing and visit schedule on Cycle 2 Day 1 are to be followed according to Table 1 (even if biopsy is collected on Day 2 or Day 3)	Predose		x	x				x		x	x	
		2H Postdose (prebiopsy)	± 30 min		x								
		3H to 7H Postdose					x						
		Post biopsy (optional)	Up to 6 hours after biopsy		x								
	Day 8 (only Part I)	Predose		x	x								
	Day 15 (only Part I)	Predose		x	x								
Subsequent Cycles (8)	Day 1	Predose		x	x				x (C3, C5 and every 4 cycles thereafter eg C9 etc)		x	x	
		2H Postdose	±15 min		x (C3 and every 2 cycles thereafter eg C5, C7 etc)								
		4H Postdose	±30 min		x (C3 and every 2 cycles thereafter eg C5, C7 etc)							x (C3 and every 2 cycles thereafter eg C5, C7 etc)	
At time of progression								x	x			x	
EOT/Discontinuation Visit				x					x (Not needed if sample was collected at time of progression)			x (Not needed if sample was collected at time of progression)	
If tumor tissue collected per SOC (e.g. surgery) (optional, collection of tumor tissue sample)							x						
Unscheduled Biopsy eg at time of PR, SD or disease progression (optional, if deemed clinically feasible by the Investigator)							x						
Unscheduled PK for specific events (9)					x								

Use of this document is governed by the terms of use on the first page of this document.

[redacted] C = Cycle; ctDNA = circulating tumor deoxyribonucleic acid; CYP = cytochrome P; D = Day; DNA = deoxyribonucleic acid; ECG = electrocardiogram; eCRF = electronic case report form; EOT = end of treatment; H = hours; min = minutes; PK = pharmacokinetics; PR = partial response; [redacted] RBR = Research Biosample Repository; RNA = ribonucleic acid; SD = stable disease; SOC = standard of care.

1 For study visit window see Table 1.

2 "Predose" is a sample to be collected shortly before (preferably no more than 2 hours before, unless otherwise stated) the time of drug administration ("Dosing") of RO7589831. RO7589831 should be taken preferably at the same time of each day. Scheduled timepoints refer to times in hours after the first morning dosing of RO7589831. Actual time of PK sample collection needs to be recorded and entered in the eCRF. If a [redacted] dosing schedule is implemented, "predose" and "postdose" refer to the first dosing in the day and the [redacted] sample should be taken prior to the second dose. [redacted]

3 Sampling timepoints might be modified based on emerging data.

4 If archival tumor tissue is not available, fresh tumor biopsy from screening may be used instead (see Section 8.7.1.3 for tissue requirements).

5 If participants are re-screened, tumor tissue collection does not need to be repeated if it was already collected during previous screening.

6 If tumor tissue sample was collected within 12 weeks from screening or if participants are re-screened, tumor biopsy does not need to be repeated as long as the tumor tissue was collected as required per protocol (Section 8.7.1.3) and no systemic anti-cancer treatment was given in the meantime.

7 If not feasible as scheduled, biopsy may be obtained at a later visit after consultation with the Sponsor, when the biopsy is deemed clinically safe and feasible by the Investigator, and all samples must be collected on the same day, e.g., if biopsy is collected on C2D15 also the ECG assessment, PK samples, biomarker and ctDNA blood samples must be collected on C2D15. If biopsy cannot be collected on the scheduled day and cannot be rescheduled, the ECG assessment, PK, biomarker and ctDNA blood samples should be collected as described in Table 2 for participants not undergoing biopsy on C2D1.

8 From C9, visits frequency can be reduced to once every two cycles (e.g., C9D1, C11D1 etc.).

9 Additional PK sample should be collected at an unscheduled visit in case of suspected overdose *of RO7589831*, dose-limiting toxicity or adverse events leading to dose reduction, dose interruption or clinically significant ECG changes. The PK samples should be taken as soon as possible (i.e., ideally at the time of the development of such an event or, if not feasible, at the earliest possible convenience.

10 *In selected Part I* [redacted] *cohorts urine may be collected over a period of* [redacted] *if* [redacted]*) following administration of RO7589831 on day 1 of the study. A single pool of all urine collected during this time should be made. The total urine volume should be recorded.*

11 *It is strongly recommended that participants are reminded on the day before the clinic visit to hold the morning dose on the day of the clinic visit to allow accurate PK/PD sampling and ECG measurements. If patient did not hold morning daily dose until the clinic visit, then the PK, PD sampling and associated ECG will not be collected on this day but will be shifted to the next day and the patient will be reminded to hold off the planned morning dose accordingly. All the other visits assessment (see Table 1) will be carried out as per original schedule.*

Use of this document is governed by the terms of use on the first page of this document.

Table 4 Pharmacokinetic, Biomarker, and Electrocardiogram Schedule for Participants Undergoing Paired Tumor Biopsy Collection and Time of On-treatment Tumor Biopsy is Changed to Pre-dosing *(Part I* [redacted]

Sample Category				ECG (triplicate)	PK	Biomarker	Biomarker	Biomarker	Biomarker	Biomarker	Part I only Biomarker	Biomarker	*PK*
Sample Type					Plasma	Archival tumor tissue (4)	Fresh tumor tissue	Blood	Blood	Blood	Plasma	Plasma	*Urine collection*
Sample Intended purpose					For RO7589831	For biomarker analysis	For biomarker analysis	For response prediction	For ctDNA analysis	For Clinical Genotyping analysis	For CYP induction status endogenous marker analysis	For exploratory biomarker analysis	*For RO7589831*
Cycle length: 21 days	Day(1)	Scheduled time (H=hours) (2) (3)	Time window										
**Screening**	Day -28 to Day -1			x		x (5)	x (6)						
Cycle 1 [redacted]	Day 1	Predose		x	x			x (within 72 hours from C1D1 dosing)	x (within 72 hours from C1D1 dosing)	x (within 72 hours from C1D1 dosing)	x (within 72 hours from C1D1 dosing)	x	*x*
		0.5 H Postdose	*±10 min*	*x*	*x*								
		1H Postdose	±15 min	x	x								
		2H Postdose	±15 min	x	x								
		3H Postdose	±15 min	x	x								
		4H Postdose	±15 min	x	x							x	
		6H Postdose	±30 min		x								
		8H Postdose	±30 min	x	x								
		12H Postdose	±30 min	x	x *(not needed if* [redacted] *is implemented)*								
	Day 8 *(11)*	Predose		x	x						x	x	
		0.5 H Postdose	*±10 min*	*x*	*x*								
		1H Postdose	±15 min	x	x								
		2H Postdose	±15 min	x	x								
		3H Postdose	±15 min	x	x								
		4H Postdose	±15 min	x	x							x	
		6H Postdose	±30 min		x								
		8H Postdose	±30 min	x	x								
	Day 15 (only Part I)	Predose		x	x						x		
Cycle 2	Day 1 or Day 2 or Day 3 (7)<br><br>ECG, blood and plasma samples must be collected on the same day of biopsy eg if biopsy is collected on C2D2 also the ECG and all blood and plasma samples must be collected on C2D2<br><br>NOTE: Dosing and visit schedule on Cycle 2 Day 1 are to be followed according to Table 1 (even if biopsy is collected on Day 2 or Day 3)	Predose and pre-biopsy		x	x				x		x	x	
		Predose					x						
	Day 8 (only Part I)	Predose		x	x								
	Day 15 (only Part I)	Predose		x	x								
Subsequent Cycles (8)	Day 1	Predose		x	x				x<br>(C3, C5 and every 4 cycles thereafter eg C9 etc)		x	x	
		2H Postdose	±15 min		x<br>(C3 and every 2 cycles thereafter eg C5, C7 etc)								
		4H Postdose	±30 min		x<br>(C3 and every 2 cycles thereafter eg C5, C7 etc)							x<br>(C3 and every 2 cycles thereafter eg C5, C7 etc)	
At time of progression								x	x			x	
EOT/Discontinuation Visit				x					x<br>(Not needed if sample was collected at time of progression)			x<br>(Not needed if sample was collected at time of progression)	
If tumor tissue collected per SOC (e.g. surgery) (optional, collection of tumor tissue sample)							x						
Unscheduled Biopsy<br>eg at time of PR, SD or disease progression (optional, if deemed clinically feasible by the Investigator)							x						
**Unscheduled PK for specific events (9)**					x								

Use of this document is governed by the terms of use on the first page of this document.

[redacted] C = Cycle; ctDNA = circulating tumor deoxyribonucleic acid; CYP = cytochrome P; D = Day; DNA = deoxyribonucleic acid; ECG = electrocardiogram; eCRF = electronic case report form; EOT = end of treatment; H = hours; min = minutes; PK = pharmacokinetics; PR = partial response; [redacted] RBR = Research Biosample Repository; RNA = ribonucleic acid; SD = stable disease; SOC = standard of care.

1 For study visit window see Table 1.

2 "Predose" is a sample to be collected shortly before (preferably no more than 2 hours before, unless otherwise stated) the time of drug administration ("Dosing") of RO7589831. RO7589831 should be taken preferably at the same time of each day. Scheduled timepoints refer to times in hours after the first morning dosing of RO7589831. Actual time of PK sample collection needs to be recorded and entered in the eCRF. If a [redacted] dosing schedule is implemented, "predose" and "postdose" refers to the first dosing in the day and [redacted] sample should be taken prior to the second dose. [redacted]

3 Sampling timepoints might be modified based on emerging data.

4 If participants are re-screened, tumor tissue collection does not need to be repeated if it was already collected during previous screening.

5 If archival tumor tissue is not available, fresh tumor biopsy from screening may be used instead (see Section 8.7.1.3 for tissue requirements).

6 If tumor tissue sample was collected within 12 weeks from screening or if participants are re-screened, tumor biopsy does not need to be repeated as long as the tumor tissue was collected as required per protocol (Section 8.7.1.3) and no systemic anti-cancer treatment was given in the meantime.

7 If not feasible as scheduled, biopsy may be obtained at a later visit after consultation with the Sponsor, when the biopsy is deemed clinically safe and feasible by the Investigator, and all samples must be collected on the same day, e.g., if biopsy is collected on C2D15 also the ECG assessment, PK samples, biomarker and ctDNA blood samples must be collected on C2D15. If biopsy cannot be collected on the scheduled day and cannot be rescheduled, the ECG assessment, PK samples, biomarker and ctDNA blood samples should be collected as described in Table 2 for participants not undergoing biopsy on C2D1.

8 From C9, visits frequency can be reduced to once every two cycles (e.g., C9D1, C11D1 etc.).

9 Additional PK sample should be collected as unscheduled visit in case of suspected overdose, in case of dose-limiting toxicity or in case of adverse events leading dose reduction, dose interruption or clinically significant ECG changes. The PK samples should be taken as soon as possible (i.e., ideally at the time of the development of such an event or, if not feasible, at the earliest possible convenience).

10 *In selected Part I* [redacted] *cohorts urine may be collected over a period of* [redacted] *if* [redacted]*) following administration of RO7589831 on day 1 of the study. A single pool of all urine collected during this time should be made. The total urine volume should be recorded.*

11 *It is strongly recommended that participants are reminded on the day before the clinic visit to hold the morning dose on the day of the clinic visit to allow accurate PK/PD sampling and ECG measurements. If participant did not hold morning daily dose until the clinic visit, then the PK, PD sampling and associated ECG will not be collected on this day but will be shifted to the next day and the participant will be reminded to hold off the planned morning dose accordingly. All the other visits assessment (see Table 1) will be carried out as per original schedule.*

Use of this document is governed by the terms of use on the first page of this document.

[redacted]

Use of this document is governed by the terms of use on the first page of this document.

[redacted]

2 [redacted]

3 [redacted]

4 [redacted]

5 [redacted]

6 [redacted]

7 [redacted]

Use of this document is governed by the terms of use on the first page of this document.

[redacted]

Use of this document is governed by the terms of use on the first page of this document.

[redacted]

Use of this document is governed by the terms of use on the first page of this document.

2. INTRODUCTION

A synthetic lethal interaction occurs between two genes when the alteration of either gene alone is viable but the alteration of both genes simultaneously results in cell death. Targeting the protein product of a gene that has a synthetic lethal interaction with a tumor-specific somatic mutation should specifically kill cancer cells while sparing normal cell (O'Neil et al. 2017). Werner syndrome (WRN) helicase has been identified as a novel synthetic lethal target in microsatellite instability (MSI) cancer (Kaelin 2005).

RO7589831 is a novel covalent, irreversible inhibitor of WRN helicase, and this is the first clinical study of RO7589831. The study will enroll participants with advanced MSI and/or deficient mismatch repair (dMMR) solid tumors.

Herein, the term "MSI" is used to indicate the microsatellite instability status of a tumor and "MSS" to indicate microsatellite stable status. The terms "MSI-high" or "MSI-low" are avoided as per the European Society For Medical Oncology (ESMO) recommendations (Luchini et al. 2019) and according to the revised Bethesda guidelines for colorectal cancers (Umar et al. 2004).

2.1 STUDY RATIONALE

Deficient mismatch repair is caused by the inactivation of genes of the DNA repair machinery that leads to DNA mismatches in microsatellites and variations in the length of tandem nucleotide repeats across the genome, known as MSI (Ellegren 2004).

The prevalence of MSI and/or dMMR has been reported to be approximately 30% for endometrial cancer, 20% for colon or gastric cancer, and less than 5% for most other tumor types, with a higher prevalence often found in Stage I to III compared to Stage IV disease (Hause et al. 2016, Le et al. 2017, Bonneville et al. 2017).

Treatment standards for MSI and/or dMMR tumors have shifted in recent years with the approval of several checkpoint inhibitors. While responses to immune checkpoint blockade in MSI and/or dMMR solid tumors are often durable, 40% to 70% of such cancers do not respond to immune checkpoint blockade or develop secondary resistance, and the use of checkpoint inhibitors can be limited by toxicity (Le et al. 2017, Marabelle et al. 2020, Diaz et al. 2022, Berton et al. 2021, Overman et al. 2018, Lenz et al. 2022, Oaknin et al. 2022, Hochster et al. 2017). Thus, novel therapies are needed for patients with MSI and/or dMMR tumors.

WRN helicase is a bi-functional enzyme belonging to the RecQ family that is required for DNA replication and DNA repair to ensure genomic stability and has been identified as a novel synthetic lethal target in MSI cancer.

Depletion of WRN helicase induces DNA double-stranded breaks (DSBs), promoting apoptosis and cell cycle arrest selectively in MSI and not in microsatellite stable (MSS)

Use of this document is governed by the terms of use on the first page of this document.

preclinical models, indicating WRN helicase as a promising drug target for MSI tumors (Chan et al. 2019, Lieb et al. 2019).

The agent investigated under this protocol, RO7589831, is a novel covalent, irreversible inhibitor of WRN helicase that causes DNA damage accumulation and has selective antitumor activity in MSI cell lines and in vivo mouse models, including patient-derived models (for details see RO7589831 Investigator's Brochure).

This entry-into-human (EiH) study will assess the safety, pharmacokinetics (PK), pharmacodynamics (PD) and preliminary clinical anti-tumor activity of RO7589831 monotherapy in participants with MSI and/or dMMR advanced solid tumors *and in combination with pembrolizumab in participants with MSI and/or dMMR advanced colorectal adenocarcinoma (CRC).*

2.2 BACKGROUND

RO7589831 is a novel covalent, irreversible inhibitor of WRN helicase that is being developed for the treatment of patients with MSI and/or dMMR advanced solid tumors.

A detailed description of the disease, current therapies, unmet medical need as well as the chemistry, pharmacology, and safety of RO7589831 is provided in the Investigator's Brochure.

2.3 BENEFIT–RISK ASSESSMENT

The management of patients with MSI and/or dMMR advanced solid tumors has recently shifted with the approval of anti-PD1/PD-L1 inhibitors in some of these tumor types. For those indications where checkpoint inhibitor therapy is not approved, and/or following immune checkpoint therapy, treatment options for patients with MSI and/or dMMR metastatic or locally advanced tumors vary by tumor type but overall benefit to patients is limited. Thus, novel therapies are needed to improve outcomes for these patients.

WRN helicase represents a novel synthetic lethal target in MSI cancer. Synthetic lethality provides a conceptual framework for the development of cancer-specific cytotoxic agents with minimal or no impact to normal cells (Kaelin 2005). Poly adenosine diphosphate-ribose polymerase (PARP) inhibitors have been the first agents successfully exploiting the dependency of PARP in breast cancer gene (BRCA) 1/2-mutated cancer and showing a favorable safety profile (Benard et al. 2012).

RO7589831 is a novel covalent, irreversible inhibitor of WRN helicase that has been shown to have selective antitumor activity in MSI cell lines and in vivo mouse models. Knock-down of WRN helicase in MSS cancer and non-transformed cells had very mild or no effect on viability, suggesting that pharmacological inhibition of WRN helicase might allow for an MSI cancer-directed therapy that spares normal cells and tissues (Lieb et al. 2019).

Use of this document is governed by the terms of use on the first page of this document.

Genetic homozygous loss-of-function mutations of WRN helicase are causative events of Werner syndrome, a progeroid disease with premature ageing phenotype. Symptoms generally do not manifest before the third decade of patients' lives indicating that loss of WRN helicase function does not cause acute toxicity in healthy tissue. This may allow for a relevant therapeutic window using WRN helicase inhibitors as anti-cancer therapy.

Currently, the clinical safety risks of RO7589831 are unknown as this is an EiH study. Potential risks for RO7589831 have been identified based on the nature of covalent inhibitor, the mode of action, as well as nonclinical data in relevant models. Potential risks include effects associated with long-term inhibition of Werner helicase, formation of drug/metabolite-protein adducts *and* enhanced sensitivity to chemotherapy toxicities are detailed in the RO7589831 Investigator's Brochure.

These potential risks have been taken into account in the risk mitigation measures for this EiH study protocol, which include the careful definition of eligibility criteria (Section 5.1 and Section 5.2), predefined approaches for dose escalation (Section 4.1.6) and enrollment staggering (Section 4.1.6). Close monitoring of adverse events (AEs), frequent safety assessments, and rules for treatment interruption/discontinuation are part of protocol-mandated interventions. Emerging safety data will be reviewed on an ongoing basis by Sponsor representatives and the Investigators throughout the study (See Section 4.1.7), and additional safety measures will be implemented if warranted.

In conclusion, due to the unmet medical need in patients with MSI and/or dMMR advanced tumors, the anticipated mechanism of action of RO7589831, as well as the risk mitigation strategies implemented in this EiH study protocol, the overall benefit-risk profile is considered favorable for participating patients.

Detailed information about the known and expected benefits in the context of potential risks and reasonably expected AEs of RO7589831 is provided in the Investigator's Brochure.

2.3.1 *Additional Considerations on Benefit-risk for the combination with pembrolizumab*

[redacted]

[redacted]

Use of this document is governed by the terms of use on the first page of this document.

[redacted]

2.3.2 Additional Considerations on Benefit-risk in the Setting of COVID-19 Pandemic

In the setting of the coronavirus disease 2019 (COVID-19) pandemic, participants with comorbidities, including those with solid tumors, represent a more vulnerable population. Infection with severe acute respiratory syndrome coronavirus 2 (SARS-CoV-2) has been associated with higher morbidity and mortality in patients with cancer in some retrospective analyses (Dai et al. 2020, Shah et al. 2020, Yang et al. 2020, Robilotti et al. 2020). It is, however, unclear whether or how cancer therapies such as chemotherapy, targeted therapy, or immunotherapy affect the incidence or severity of COVID-19.

Considering the early stage of development of this study and the limited number of participants, a conservative approach has been adopted. Patients with symptomatic disease (including COVID-19) at screening will be excluded.

Investigators should not administer RO7589831 to participants with suspected

Use of this document is governed by the terms of use on the first page of this document.

SARS-CoV-2 infection and investigational sites will report any occurrences of COVID-19. The risk of SARS-CoV-2 infection will be minimized by complying with local guidelines and hospital policies. Participants may receive non-live vaccines, such as vaccines against SARS-CoV-2, prior to and during the course of the study (Section 6.4.1).

3. OBJECTIVES AND ENDPOINTS

The objectives and corresponding endpoints are provided in Table 7.

Table 7 Objectives and Endpoints

Objectives	Endpoints
**Primary**	
• To evaluate the safety and tolerability of RO7589831 as monotherapy *and in combination with pembrolizumab*	• Incidence, nature, and severity of adverse events (AEs) graded according to the National Cancer Institute (NCI) Common Terminology Criteria for Adverse Events (CTCAE) v5
• To determine the maximum tolerated dose (MTD) and/or the recommended dose for expansion (RDE) or the recommended Phase 2 dose (RP2D) for RO7589831 monotherapy *and for RO7589831 in combination with pembrolizumab*	• Occurrence of dose-limiting toxicities (DLTs), nature and frequency of AEs, anti-tumor activity endpoints and pharmacokinetics (PK)
**Secondary**	
• To characterize the PK profile of RO7589831 as monotherapy *and in combination with pembrolizumab*	• PK profiles and parameters derived for RO7589831 including but not limited to: C_{max}, T_{max}, C_{min}, CL/*F*, $T_{1/2}$, V_{ss}, and AUC for various time intervals following single and multiple doses of RO7589831
• To evaluate the anti-tumor activity of RO7589831 as monotherapy *and in combination with pembrolizumab*	• Objective response rate (ORR), disease control rate (DCR), duration of response (DOR) and progression-free survival (PFS) according to Response Evaluation Criteria in Solid Tumors (RECIST) v1.1 as assessed by the Investigators<br>• Overall survival (OS)
**Exploratory**	
• To assess the pharmacodynamic (PD) effects of RO7589831 by changes of metabolic activity	• Change in imaging parameters such as standardized uptake value (SUV) and number of metabolic responses assessed by fluorodeoxyglucose positron emission tomography/ computed tomography (FDG-PET/CT) at different dose levels
• To explore biomarkers associated with tumor response	• Retrospective assessment of biomarkers including, but not limited to microsatellite instability (MSI) and mismatch repair (MMR) status and additional exploratory biomarkers from tumor and blood samples

Use of this document is governed by the terms of use on the first page of this document.

• To evaluate PD markers in tumor tissue and/*or* peripheral blood to support proof of mechanism, response prediction and/*or* RP2D selection	• Baseline expression and/or change in levels of target engagement and/*or* PD markers related to the mode of action of RO7589831 at different dose levels
• To evaluate the effect of RO7589831 administration on endogenous CYP3A biomarker	• Plasma concentrations, percentage change from baseline and ratios of hydroxycholesterol, and total cholesterol (TC)
• To determine the effect of food on the PK profile of RO7589831	• Effect of a meal on RO7589831 PK parameters such as C_{max}, T_{max}, AUC, and $T_{1/2}$ following oral administration of RO7589831 compared to fasting conditions
• To evaluate the effect of RO7589831 as monotherapy on electrocardiogram (ECG) parameters	• Change from baseline in ECG readings from central reading
• To explore the effect of inhibitors or inducers of CYP450 enzymes on PK of RO7589831 [a]	• Effect of inhibitors or inducers of CYP450 enzymes on RO7589831 PK parameters such as C_{max}, T_{max}, AUC, and $T_{1/2}$ following oral administration of RO7589831
• To explore the effect of RO7589831 as an inducer of CYP450 enzymes [a]	• Effect of RO7589831 as inducer of CYP450 enzymes on PK parameters such as C_{max}, T_{max}, AUC, and $T_{1/2}$ of CYP substrates following oral administration of RO7589831
• To evaluate the clinical activity of RO7589831 as monotherapy	• Objective response rate (ORR), disease control rate (DCR), duration of response according to Response Evaluation Criteria in Solid Tumors (RECIST) v1.1 as assessed by blinded independent central review (BICR)<br>• Progression-free survival (PFS) as assessed by BICR
• To evaluate the health-related quality of life of participants treated with RO7589831 as monotherapy and in combination with *pembrolizumab*	• Change from baseline in functioning scale as assessed through use of the European Organisation for Research and Treatment of Cancer Quality-of-life Questionnaire-Function 17 (EORTC QLQ-F17)
• [redacted]	• [redacted]

Use of this document is governed by the terms of use on the first page of this document.

• To assess tolerability of participants treated with RO7589831 as monotherapy and in combination with *pembrolizumab*	• Presence, frequency of occurrence, severity, and/or degree of interference with daily function of symptomatic treatment toxicities as assessed through use of the NCI Patient-Reported Outcomes (PRO)-CTCAE<br>• Change from baseline in symptomatic treatment toxicities, as assessed through use of the NCI PRO-CTCAE<br>• Frequency of participants' response of the degree they are troubled with treatment symptoms, as assessed through use of the single-item European Organisation for Research and Treatment of Cancer Item List 46 (EORTC IL46)
• To investigate the presence of RO7589831-derived metabolite(s) in plasma *and urine as well as the presence of RO7589831 in urine.*	• Relative abundance and PK parameters of circulating RO7589831-derived metabolite(s).<br>• *Amount of RO7589831 recovered unchanged in urine and potential identification of renally excreted metabolites.*

[a] Details on design and participant population for [redacted] DDI cohort will be added via a protocol amendment and are not part of the current protocol

4. STUDY DESIGN

4.1 OVERALL DESIGN

This is a first in human Phase I, open-label, multicenter, dose-escalation and dose expansion study to evaluate the safety, tolerability, PK, PD and preliminary anti-tumor activity of RO7589831 monotherapy in participants with MSI and/or dMMR advanced solid tumors *and in combination with pembrolizumab in participants with MSI and/or dMMR advanced colorectal adenocarcinoma (CRC)* (Figure 1).

The study consists of the following parts (Table 8):

Table 8 Study Parts and Participants Population

Study Part	Participants Population
**Part I**	
• **Dose Escalation and Backfill cohorts**	MSI and/or dMMR advanced solid tumors
[redacted]	

Use of this document is governed by the terms of use on the first page of this document.

[redacted]; dMMR = deficient mismatch repair; MSI = microsatellite instability

1 [redacted]

2 [redacted]

3 [redacted]

4.1.1 Part I Dose Escalation and Backfill Cohorts

The overall aim of the dose-escalation Part I is to determine the maximum tolerated dose (MTD) and/or the recommended dose(s) for expansion (RDE) *or recommended Phase 2 dose (RP2D)* of RO7589831 monotherapy and will enroll participants with any MSI and/or dMMR advanced solid tumors.

The starting dose of [redacted] was determined based on preclinical PK data, toxicokinetic data, toxicology studies and anti-tumor activity in preclinical models (Section 4.3).

RO7589831 will be administered orally (po) [redacted] in 3-week cycles based upon the DLT observation period, planned schedule of clinical assessments and potential for combination with other anti-PD1/PDL1 therapies.

Dose escalation will be guided by a modified Continual Reassessment Method (mCRM) with an Escalation with Overdose Control (EWOC) (Neuenschwander et al. 2008; Appendix 13).

If the [redacted] regimen shows unacceptable toxicity or unfavorable PK characteristics, the dosing schedule may be changed, e.g., [redacted] The cohorts with the new dosing schedule will use the total daily dose equivalent proposed by the mCRM EWOC model.

During dose escalation, backfill cohorts may be enrolled to further characterize the safety profile, PK, PD and preliminary clinical activity of RO7589831 and to support the selection of the RDE. The selection of dose levels for backfill cohorts and the number of participants allocated to each cohort will be based on emerging PK/PD modeling, safety data and potential early signs of clinical response. The dose levels selected for the backfill cohorts will not be higher than the highest dose cleared in the dose escalation. If dose escalation cohorts and backfill cohorts are open in parallel, the Sponsor will decide where to enroll each participant. Depending on emerging data, these decisions might aim at enrichment of certain participant subgroups within the backfill cohorts, e.g., by selection for certain tumor types. If two or more backfill cohorts are open in parallel, enrollment into these cohorts *may* be randomized.

Use of this document is governed by the terms of use on the first page of this document.

[redacted]

[redacted]

- [redacted]
- [redacted]

[redacted]

[redacted]

4.1.3 Assessment of Food Effect

[redacted]

[redacted]

- [redacted]
- [redacted]

[redacted]

[redacted]

Use of this document is governed by the terms of use on the first page of this document.

[redacted]

[redacted]

[redacted]

[redacted]

[redacted]

[redacted]

[redacted]

[redacted]

Use of this document is governed by the terms of use on the first page of this document.

4.1.6 Dose Escalation

4.1.6.1 Dose-Escalation Decision Criteria *for Part I*

Part I will enroll at least 3 participants in each dose escalation cohort. The starting dose of RO7589831 will be [redacted] po [redacted] and the dose escalation will be guided by a mCRM with EWOC (Neuenschwander et al. 2008) until any of the stopping criteria described in Section 4.1.6.4 are fulfilled. Participants enrolled in the backfill cohorts will be considered for dose decisions guided by the mCRM with EWOC (Appendix 13).

To allow for the assessment of any severe and unexpected RO7589831 acute or subacute study toxicity, participants will be enrolled in a staggered manner, as follows:

- For the first two dose cohorts, the second participant enrolled in each dose cohort, will receive the initial RO7589831 dose at least three days after the first participant.
- For subsequent dose escalation cohorts (i.e., from cohort 3 and beyond) the requirement of staggering will be based on the observed safety data in previous cohorts and will be decided between the Sponsor and the study Investigators (as described in Section 4.1.6.5).

The dose-limiting toxicity (DLT) observation period will be 21 days (i.e., Cycle 1) starting from C1D1 until C1D21, and the subsequent dose cohort may be opened after at least 3 DLT-evaluable participants have completed their DLT window.

Participants who discontinue from study treatment prior to completing the DLT assessment window for reasons other than a DLT will be considered non-evaluable for dose-escalation decisions and MTD assessments, and may be replaced by an additional participant at that same dose level. Participants who take less than 75% of planned doses of RO7589831 during the DLT assessment window for reasons other than DLT, will be considered non-evaluable for dose-escalation decisions and MTD assessments and may be replaced.

For each new cohort, the mCRM EWOC model may recommend increasing the dose, decreasing the dose, or expanding the cohort at the same dose, depending on the DLT events observed thus far.

The final decision for the dose of the next cohort will be made by the Dose Decision Team (DDT) which includes Sponsor representatives and Investigators, taking all available relevant safety, clinical, PK, PD data (from dose escalation and backfill cohorts) and the model-recommendation into account. The DDT will be able to overrule the mCRM EWOC model recommendation based on all of the data available, including the totality of safety data from all dose cohorts, and also safety events that occur outside of the DLT assessment window. Additional information about the dose escalation process for the study will be documented in the separate dose escalation plan (DEP).

*In Part I of the study, i*ntra-participant dose escalation *may be* allowed *if participants have experienced disease progression (per RECIST v1.1) but the Investigator deems the*

Use of this document is governed by the terms of use on the first page of this document.

participant might benefit from higher dose of RO7589831 upon the Investigator's request after discussion with the Medical Monitor and careful assessment and discussion of risk versus benefit with the participant by the Investigator (Appendix 1). The dose will not exceed the highest dose level that was deemed safe during dose-escalation.

Intra-participant dose-escalation decisions will be made based on available safety data from the participant being considered as well as the totality of safety data available at the time of decision making. Participants who have had a DLT, will not be eligible for intra-participant dose escalation.

For analysis purposes, participants will be included in the cohort assigned at Cycle 1 Day 1, regardless of intra-participant dose escalation.

[redacted]

- [redacted]
- [redacted]
- [redacted]
- [redacted]

[redacted]

Use of this document is governed by the terms of use on the first page of this document.

[redacted]

[redacted]

4.1.6.3 Dose-Limiting Toxicities

For the assessment of DLTs, all AEs will be graded according to National Cancer Institute Common Terminology Criteria for Adverse Events (NCI CTCAE) v5.0.

DLTs will be treated according to clinical practice and will be monitored through to their resolution.

A DLT will be defined as any of the following adverse events occurring during the 21 days DLT assessment period, unless such events are attributed by the Investigator to another clearly identifiable cause (e.g., documented disease progression, concomitant or preexisting medication, or intercurrent illness):

- Grade 5 event
- Any study treatment-related toxicity that causes the participant to permanently discontinue treatment during C1
- Any study treatment-related toxicity that results in a prolonged interruption of treatment for more than 14 days. [redacted]
- **Hematological DLTs:**
  - Grade 4 neutropenia (absolute neutrophil count (ANC) $<$ 500 cells/mL) lasting $>$ 7 days [(a)]
  - Grade 3 or 4 febrile neutropenia
  - Grade 3 thrombocytopenia associated with clinically significant bleeding
  - Grade 4 thrombocytopenia of any duration
  - Grade 4 anemia
- **Any Grade 3 or Grade 4 non-hematologic laboratory value excluding:**
  - Grade $\geq$ 3 electrolyte abnormality that is not clinically complicated, lasts $<$ 72 hours and resolves spontaneously or responds to appropriate medical intervention per SoC[(a)]
  - Grade $\geq$ 3 amylase or lipase that is not associated with symptoms or clinical *or imaging* manifestations of pancreatitis
  - Grade 3 laboratory abnormalities that are asymptomatic and considered by the Investigator not to be clinically significant.

Use of this document is governed by the terms of use on the first page of this document.

- **Laboratory abnormality that results in a drug-induced liver injury**:
  - Aminotransferase (alanine aminotransferase (ALT) and/or aspartate aminotransferase (AST)) > 3 × upper limit of normal (ULN) (or > 5 ULN for participants with liver metastases); and
  - Total bilirubin > 2 × ULN, without initial findings of cholestasis (elevated serum alkaline phosphatase); and
  - No other reason can be found to explain the combination of increased ALT/AST and total bilirubin
- **Non-hematological clinical DLTs:**
  - Any Grade ≥ 3 non-hematologic clinical (non-laboratory) toxicity <u>excluding</u>:
    - Grade 3 arthralgia and/or Grade 3 myalgia that can be adequately managed with supportive care or that improves/resolves to Grade ≤ 1 within 7 days [(a)]
    - Grade 3 nausea or vomiting that lasts < 48 hours, and resolves to Grade ≤ 1 either spontaneously or with the use of anti-emetics per standard of care (SoC) [(a)]
    - Grade 3 diarrhea that lasts < 72 hours, does not result in hospitalization and resolves to Grade ≤ 1 either spontaneously or with anti-diarrheal per SoC [(a)]
    - Grade 3 fatigue for less than 7 days [(a)]
    - [redacted]
      - [redacted]
      - [redacted]
- Any other study treatment-related toxicity considered significant enough to qualify as a DLT in the opinion of the Investigator and after discussion with the Medical Monitor.

[a] AEs related to laboratory, vital, or clinical abnormalities must be assessed prior to the end of the applicable time window to determine if a DLT has occurred

Definition of DLT-evaluable participants and possible participant replacement, can be found in Section 9.3 and Section 7.1.3, respectively.

4.1.6.4 Stopping Rules Criteria

The dose range tested during dose escalation is described in Section 4.3.

Dose escalation *in Part I* will be halted according to the rules for the mCRM EWOC model defined in the Appendix 13. In addition, if sufficient characterization of RO7589831 with regards to safety, PK, and/or PD is obtained and the RDE(s) is defined

Use of this document is governed by the terms of use on the first page of this document.

before reaching MTD (e.g., if by clinical judgment no clinical benefit can be assumed to be gained from additional cohorts), dose escalation will be stopped.

[redacted]

4.1.6.5 Communication Strategy

Information will be communicated, as follows:

1. Upon completion of all screening evaluations and confirmation that a participant has met all eligibility criteria via the Eligibility Screening Form (see Section 6.3.1), sites will obtain the participant's number from an interactive web response system (IwRS).
2. After each participant receives their first dose of RO7589831 [redacted], the Investigator(s) must confirm to the Sponsor that the participant has been dosed and provide a brief summary of the status of the participant in terms of safety and tolerability to RO7589831 [redacted], communicated by email and/or telephone as soon as reasonably possible and within one business day.
3. In the event of a DLT, the Investigator will contact the Medical Monitor immediately to discuss participant status and action taken or to be taken.
4. The Sponsor will hold a teleconference with the Investigators prior to each dose escalation to discuss the safety and tolerability of RO7589831 and to discuss the dose(s) and the enrollment staggering for the next cohort. [redacted]

In addition to these communications, the Sponsor and Investigators will be in regular contact throughout the study by email/telephone/fax, as per normal interactions during the conduct of a clinical study, and the Sponsor will arrange regular teleconferences and meetings to discuss study status *as needed*.

The Sponsor will be available 24 hours a day to discuss any medical or study-related issues that may arise during the conduct of this study.

Use of this document is governed by the terms of use on the first page of this document.

4.1.7 Administrative Structure

No formal third-party data monitoring committee is planned.

During dose escalation *in Part I* [redacted] the DDT will decide on the dose for the next cohort (see Section 4.1.6). The dose-escalation process for the study is documented in the DEP.

[redacted] medical data review will be performed regularly by an Internal Monitoring Committee (IMC) composed of key Sponsor functional representatives. To ensure the safety of the participant during the study, the IMC will make recommendations regarding study conduct on the basis of trial safety data and emerging benefit-risk, including evaluation of the totality of the data consisting of the available safety, efficacy, PK and PD profile at the time of each interim analysis.

For details, see the IMC charter.

4.2 SCIENTIFIC RATIONALE FOR STUDY DESIGN

The study rationale is provided in Section 2.1.

4.2.1 Rationale for Study Population

Werner helicase is an enzyme required for DNA replication and DNA repair to ensure genomic stability and has been identified as a novel synthetic lethal target in MSI cancer (Chan et al. 2019, Lieb et al. 2019).

MSI is a condition of genetic hypermutability resulting from defective DNA mismatch repair (MMR), and the presence of MSI represents phenotypic evidence that MMR is not functioning normally (Luchini et al. 2019).

The agent investigated under this protocol, RO7589831, is a novel covalent, irreversible inhibitor of WRN helicase that has selective antitumor activity in MSI cell lines and mouse models but not in an MSS background. Based on these preclinical data, therapeutic effect is expected in participants with MSI solid tumors, hence only participants with MSI and/or dMMR solid tumors will be enrolled in this study. Different tests are recommended to determine MMR and MSI status, which have been shown to have a high concordance rate. (Guyot D'Asnières De Salins et al. 2021, Ali-Fehmi et al. 2023).

Therefore in this study, participants' eligibility will be based on available MSI and/or dMMR tumor status determined as part of the routine diagnostic workup and SoC *in a certified laboratory*, such as Luchini et al. 2019, Chakravarty et al. 2022. *MSI and/or dMMR tumor status may also be assessed prospectively with an approved test in a certified laboratory or as part of an approved IVD performance study where applicable.* Based on emerging data, the eligibility requirements might be changed to include participants with MSI status only.

Use of this document is governed by the terms of use on the first page of this document.

MSI is a hallmark of Lynch syndrome, an inherited cancer syndrome that predisposes patients to colon, endometrial, gastric, ovarian, small intestine, liver, hepatobiliary, upper urinary tract, brain, and prostate cancers. Most MSI/dMMR tumors (~80%) are sporadic, as only 15% to 20% of MSI/dMMR tumors can be attributed to Lynch syndrome (Leclerc et al. 2021, Lavacchi et al. 2020). MSI is present in up to 20% of sporadic gastric and colorectal cancers, 20% to 30% endometrial cancers and less than 5% of other tumor types (Hause et al. 2016).

Treatment standards for MSI and/or dMMR advanced tumors have recently shifted with the approval of anti-PD1/PD-L1 therapies in these tumor types. Pembrolizumab (anti-PD1) has been the first United States (U.S.) Food and Drug Administration (FDA)-approved immune checkpoint inhibitor based on a common biomarker rather than the primary cancer type, in this case for the treatment of metastatic chemotherapy-refractory MSI tumors (Marcus et al. 2019). After pembrolizumab, additional immune checkpoint inhibitors such as nivolumab with or without ipilimumab, and dostarlimab have been approved for MSI and/or dMMR tumors across different indications including CRC and endometrial cancer (Oaknin et al. 2020, Lenz et al 2022).

Although responses to immune checkpoint blockade in MSI and/or dMMR solid tumors are often durable, 40% to 70% of patients do not benefit from the current approved immunotherapies (Marabelle et al. 2020, Diaz et al. 2022, Berton et al. 2021, Overman et al. 2018, Lenz et al. 2022, Oaknin et al. 2020, Hochster et al. 2017).

After immune checkpoint inhibitor therapy or for patients not receiving immune checkpoint inhibitors that are refractory to standard therapy in the advanced setting, there is no further therapy directly exploiting the dMMR or MSI status. Additional treatment options, therefore, do not differ for patients with MSI or MSS solid advanced tumors, and vary depending on the tumor type, patient's performance status, and goal of care, and might include chemotherapy, as well as targeted agents (NCCN Guidelines, ESMO Guidelines). These therapies are generally associated with relevant toxicity and limited duration of disease control, resulting in an overall limited benefit to patients (Bui et al. 2022). Hence, novel therapies are needed for patients with MSI and/or dMMR advanced solid tumors.

For a detailed description of MSI and/or dMMR solid tumors, current therapies and unmet medical need, see the RO7589831 Investigator's Brochure.

4.2.2 Rationale for Biomarker Assessments

Archival tumor tissue will be mandatorily collected from all participants for assessment of exploratory biomarkers *and/or eligibility assessment* including, but not limited to, assessment of MSI and/or dMMR status as response prediction biomarkers. In cases where no archival tumor tissue is available a fresh biopsy at baseline must be provided for exploratory analysis.

Use of this document is governed by the terms of use on the first page of this document.

MSI tumor status may be assessed centrally through use of the Foundation Medicine FoundationOne Companion Diagnostic (F1CDx) assay and/or Promega OncoMate™ MSI Dx Analysis System, which may be considered investigational per local regulations.

To investigate the effect of RO7589831, longitudinal circulating tumor DNA (ctDNA) monitoring from blood will be implemented for all participants. For many cancer therapies, ctDNA longitudinal monitoring is used as a surrogate of clinical efficacy to assess treatment response (Bratman et al. 2020, Dasari et al. 2020, Marsavela et al. 2022). Other ctDNA-based assays also include the ability to test for MSI status and MMR gene mutations, potentially enabling the actual identification of mismatch repair and MSI status (Dasari et al. 2020, Snyder et al. 2019, Pabla et al. 2019, Willis et al. 2019).

Additional blood samples will be collected throughout the study for all participants to assess exploratory peripheral biomarkers such as cytokines related to the innate immune response*, amongst others,* that might be used to support future drug development.

For rationale of biomarkers measured from paired tumor tissue in backfill cohorts, see Section 4.2.3.

4.2.3 Rationale for Tumor Biopsy and Backfill Cohorts

Backfill cohorts will enroll participants who are able to provide pre-treatment and on-treatment tumor tissue at defined RO7589831 dose levels (see Section 4.1.1).

Pre-treatment and on-treatment tumor tissue biopsy are mandatory for participants enrolled in Backfill cohorts *in Part I*. Based on emerging PK and PD data from the backfill cohorts, the Sponsor may request mandatory pre-treatment and on-treatment tumor biopsies from approximately up to 8 biopsy evaluable participants per dose level enrolled [redacted]

Pre-treatment and on-treatment tumor tissue will also be collected from participants who have consented to optional tumor biopsy and are enrolled in Part I dose escalation, [redacted]

Based on the expected MoA, the pharmacological activity of RO7589831 is restricted to MSI tumors with no activity anticipated in MSS tissues or blood. Therefore, the assessment of biomarkers to support proof of mechanism and dose-response relationship that might guide dose selection of RO7589831 for expansion, can only be performed using tumor tissue and not in other tissue types such as in peripheral blood.

RO7589831 was designed to specifically bind covalently to and inhibit WRN helicase. Target engagement (TE) of WRN helicase by RO7589831 will be assessed in tumor

Use of this document is governed by the terms of use on the first page of this document.

tissue extracts from paired biopsies. By measuring the degree of TE, the relationship between dose/exposure and WRN helicase inhibition can be investigated.

Mechanistically, WRN helicase deficiency leads to enhanced DSBs, cell cycle arrest, genomic instability, and apoptosis in MSI cells (Chan et al. 2019). Non-clinical studies have demonstrated induction of the DNA damage response (DDR) pathway after WRN helicase inhibition in MSI tissue as well as transcriptional gene expression changes in TP53-related pathways and others (for details see RO7589831 Investigator's Brochure). [redacted] will be monitored in paired tumor biopsies, pre-treatment and on-treatment, to determine further downstream effects of WRN helicase inhibition. To minimize the number of participants enrolled in the backfill cohorts with mandatory tumor tissue collection, the initiation of these cohorts will be guided by emerging PK/PD modeling, safety data, and potential early signs of clinical response.

In summary, assessing the level of TE as well as the induction of the DDR pathway and associated transcriptional changes will allow to confirm the pharmacological target inhibition and associated DDR pathway induction thereby demonstrating local tumor tissue targeted Proof of Mechanism (PoM).

As WRN helicase deficiency leads to accumulation of DSBs and eventual cell death, it is conceivable that DNA fragments might accumulate in the cytoplasm of MSI cells, which in turn could have the potential to stimulate the host immune response. Therefore, secondary effects of WRN helicase inhibition by RO7589831 in MSI cells could potentially include the infiltration and/or activation of tumor-infiltrating T lymphocytes (TILs). Markers related to the activation of TILs as well as their spatial location will also be monitored from paired tumor tissue biopsies.

The biomarker assessments in tumor tissue will be complemented by blood samples to assess exploratory peripheral biomarkers that might be used to support future drug development.

Additional tumor tissue samples may be collected at time of stable disease (SD), partial response (PR) or progressive disease or as part of standard clinical practice to gain insight into possible RO7589831-mediated tumor tissue changes and/or changes to the tumor microenvironment as well as potential resistance or escape mechanisms.

All specimens for the described and additional exploratory biomarker assessments, will be used for research purposes to support the optimal dose/schedule selection of RO7589831 (RP2D), to demonstrate PoM and to investigate response prediction/mechanism of resistance to therapy.

Use of this document is governed by the terms of use on the first page of this document.

4.2.4 Rationale for [^{18}F] FDG-PET Imaging

[^{18}F]-Fluorodeoxyglucose ([^{18}F]-FDG), a glucose analog, is taken up by high metabolic activity tumor cells, phosphorylated, and trapped in the cell. [^{18}F]-FDG positron emission tomography (PET) imaging is increasingly used to monitor tumor response in participants. The quantitative assessment of therapy-induced changes in tumor [^{18}F]-FDG uptake may allow the prediction of tumor response and patient outcome very early in the course of therapy (Weber 2005). Studies have shown that fluorodeoxyglucose (FDG)-PET can predict the biological response to targeted therapies early in the course of treatment, including to inhibitors of PARP, which plays a role in DNA damage repair (Benard et al. 2012, Schettini et al. 2021).

Furthermore, it is reported that higher tumor metabolic burden is associated with MSI in CRC (Song et al. 2022, Liu et al. 2021).

In a preclinical MSI tumor-bearing mouse model, a clear and fast dose-dependent decrease of several tumor metabolic parameters was observed due to the cell cycle arrest and apoptosis of MSI cells caused by WRN helicase inhibitor administration. Metabolic changes preceded tumor growth arrest or tumor shrinkage (RO7589831 Investigator's Brochure).

Baseline and on-treatment FDG PET scans will be incorporated in the assessment and modelling of PK/PD relationships.

Taken together, the available evidence supports the use of FDG-PET as a distal PD marker and possible early efficacy signal for WRN helicase inhibition.

4.2.5 Rationale for Pharmacokinetic Assessments

In this study, RO7589831 will be tested in humans for the first time, and it is important to fully characterize the PK profile of RO7589831 as monotherapy at different doses. The frequent sampling schedule that follows RO7589831 administration is designed to capture data at a sufficient number of timepoints to provide a detailed profile of the absorption, distribution, and elimination of this drug. PK sampling [redacted] postdose is proposed on C1D1 and C1D8 to allow for characterization of the PK profile following single and multiple RO7589831 administrations and assess any effects of accumulation or autoinduction on the PK of RO7589831. Predose (trough concentration) and 2 to 4h postdose (anticipated C_{max}) samples will also be collected on Day 1 (D1) of selected subsequent cycles to evaluate any changes in the PK of RO7589831 over the course of the study. In Part I backfill cohorts, PK samples will be collected before and after the on-treatment tumor biopsy to enable assessment and modelling of PK/PD relationships. Residual PK samples from any of the cohorts may be used for the identification and qualitative PK assessment of circulating RO7589831 metabolites in the plasma.

Use of this document is governed by the terms of use on the first page of this document.

PK data obtained from participants in early cohorts will be used to assess if duration of clinical exposure is in line with that predicted from animal studies. Based on the results, adjustment of the dosing schedule (e.g., [redacted] might be implemented.

Collection of urine will be performed in one or more dose levels to estimate the amount of unchanged RO7589831 eliminated in the urine following administration of RO7589831 and potential assessment of urinary excreted RO7589831 metabolites. For details, see Section 4.2.10.

4.2.6 Rationale for Food Effect Assessment

RO7589831 is a biopharmaceutics classification system class 2 drug and therefore the PK of RO7589831 may be affected to some extent by food intake. [redacted]

4.2.7 Rationale for Central ECG Reading

The potential of non-cardiovascular drugs to delay cardiac repolarization, which may cause cardiac arrhythmias such as torsade de pointes, has emerged as a major concern for sponsors and regulators. Two International Council for Harmonisation of Technical Requirements for Pharmaceuticals for Human Use (ICH) guidelines for nonclinical (S7B) and clinical (E14) testing have been developed.

Cardiovascular toxicity assessments were incorporated in nonclinical studies conducted with RO7589831 based on the testing strategy outlined in the ICH S6 and S7B guidelines. Results from these studies suggest that RO7589831 is unlikely to cause delayed cardiac repolarization, prolongation of the QT corrected for heart rate (QTc) interval, or arrhythmias at anticipated therapeutic concentrations.

The QT/QTc assessment strategy for this trial was based on recommendations in the ICH E14 guideline and the special considerations for evaluating drug effects on the QT/QTc interval in patients with cancer (Fingert and Varterasian 2006). For participants undergoing intensive PK sampling, triplicate digital ECGs time-matched with PK sampling will be collected (Section 1.3). Together, these assessments will allow an evaluation of the relationship between RO7589831 exposure and changes in QT/QTc interval. Meal intake relative to dosing and other factors (e.g., supine position, ECGs collected before PK sampling) will be standardized, as it is well established that numerous external factors such as these may prolong the QT/QTc interval (Bednar et al. 2001).

The timing of ECG collections was based on the predicted RO7589831 PK profile in humans using physiologically based PK modeling of preclinical PK and in vitro hepatocyte turnover data, and a typical small-molecule PK profile. The collection

Use of this document is governed by the terms of use on the first page of this document.

schedule should allow for an adequate characterization of the QT/QTc interval over a range of RO7589831 concentrations. If the observed PK profile is different from the predicted PK profile, additional ECGs may be collected, or the ECG measurement times may be adjusted. Likewise, measurement times that are not informative will be eliminated.

[redacted]

[redacted]

[redacted]

[redacted]

Use of this document is governed by the terms of use on the first page of this document.

[redacted]

4.2.9 Rationale for Drug-Drug Interaction assessments

In vitro studies have shown the potential for RO7589831 to alter the pharmacokinetics of CYP3A and CYP2B6 substrate drugs via induction of cytochrome P450 (CYP) enzymes in the gut and liver and via inhibition of P-gp in the gut. In vitro data also indicate the potential for clearance of RO7589831 to be changed in the presence of inducers or inhibitors of CYP3A4. Consistent with regulatory guidelines (FDA In Vitro Drug Interaction Studies 2020, FDA Clinical Drug Interaction Studies 2020, EMA Guideline on the investigation of drug interactions 2012), it is planned that clinical drug-drug interaction (DDI) studies will be performed to establish whether the potential interactions identified in vitro translate into relevant DDIs in vivo. Details of the drug-drug interaction studies will be added via amendment when relevant RO7589831 dose levels have been established.

4.2.10 *Rationale for Urine Collection and Analysis*

Urine will be collected at one or more dose levels (from participants enrolled in Part I [redacted]*) in order to estimate the amount of administered*

Use of this document is governed by the terms of use on the first page of this document.

RO7589831 that is excreted unchanged in the urine. Urine collection will be performed on Day 1 for each participant.

- [redacted] *of urine collection following the administration of RO7589831 will be used in* [redacted] *or* [redacted] *settings.*
- [redacted] *of urine collection will be performed if in a* [redacted] *dose setting.*

Plasma PK profiles will be assessed in parallel for the participating individuals to measure the systemic exposure during the urine collection period. Remaining urine samples may be used for the identification of renally excreted metabolites.

4.3 JUSTIFICATION FOR DOSE

A starting dose of [redacted] has been selected for this first study in humans. This is anticipated to be a safe dose based on safety margins calculated from the nonclinical toxicokinetic data, the estimated no-observed-adverse-effect level (NOAEL) in the toxicology studies, and the predicted human exposure. The predicted human exposure at this dose corresponds to the exposures observed to cause tumor stasis in the HCT-116 mouse xenograft model.

In the 28-day repeat-dose toxicology study, the STD10 in mice (the more sensitive species) was 600 mg/kg/day (1800 mg/m^2/day) and the NOAEL was 300 mg/kg/day (900 mg/m^2/day). Because the exposures at the STD10 and the NOAEL were the same in this study, the Sponsor used the NOAEL to calculate the starting dose. A mouse-to-human body surface area (BSA) conversion factor of 12.3 and a safety factor of 10 were applied to the 300 mg/kg/day NOAEL dose in the GLP mouse toxicology study to yield the resulting human dose of [redacted] per 60-kg patient.

The projected steady-state AUC_{0-24h} and C_{max} in humans of the [redacted] starting dose are 4500 ng•hr/mL and 620 ng/mL, respectively. These values are at least 3.8- and 4.2-fold lower, respectively, than the C_{max} and AUC_{0-24h} observed in mice at the 300 mg/kg/day NOAEL dose (Table 9).

Using modeling and simulation, the [redacted] dose is predicted to correspond in exposure to a dose of [redacted] in the mouse xenograft studies. A dose of [redacted] resulted in tumor stasis using the HCT-116 xenograft model and partial tumor growth inhibition in other xenograft models such as SW48, indicating that pharmacological activity may be expected at this dose level. Further details can be found in the RO7589831 Investigators' Brochure.

Preclinical pharmacology models showed anti-tumour activity using daily administration, therefore [redacted] dosing [redacted] will be investigated initially in man. If [redacted] schedule shows unacceptable toxicity or unfavorable PK characteristics the schedule may be changed e.g., to [redacted]

Use of this document is governed by the terms of use on the first page of this document.

Collectively, the weight-of-evidence and safety margins support the proposed RO7589831 starting dose of [redacted]

Table 9 Key Study Data Supporting Starting Dose

	[redacted] Starting Dose
Predicted Human $C_{max,\ steady\ state}$	0.62 µg/mL
Predicted Human $AUC_{steady\ state}$	4.5 µg•h/mL
Predicted Human $AUC_{steady\ state}$ compared with Mouse NOAEL Day 28 AUC	4-fold lower
Equivalent dose in mouse PD studies	[redacted]

The anticipated therapeutic dose (ATD) of RO7589831 in humans is predicted to be in the range of [redacted] based on tumor regression observed in different xenograft mouse models (RO7589831 Investigators' Brochure).

The quantitative predictability of these pre-clinical efficacy models is known to be limited, and therefore, the derived clinical ATD only serve as preliminary estimates, which will be further refined, once clinical data emerge.

A listing of predicted exposures at a selection of different potential doses can be seen in Table 10.

Table 10 Simulated Steady State PK Parameters Following Oral Administration of Different QD Doses of RO7589831

[redacted]

The maximum dose *is proposed as* [redacted] in this study (*e.g.,* [redacted]. At *individual* doses above [redacted] AUC and C_{max} are expected to increase less than

Use of this document is governed by the terms of use on the first page of this document.

dose proportionately and solubility limitations are expected restricting additional attainable exposure at *individual* doses above [redacted]. Given the projected reduced exposure increases *with* doses *above* [redacted], *a* maximum daily RO7589831 *dose of* [redacted] *is proposed*.

4.4 DURATION OF PARTICIPATION

The study duration per participant from screening until safety follow-up visit may be up to approximately [redacted] (not including long-term follow-up [LTFU]).

- Screening: Days -28 to -1.
- Treatment period: continuous treatment until disease progression *(unless participants are undergoing intra-participant dose-escalation)*, unacceptable toxicity, participant withdrawal of consent, or death, whichever occurs first. Participants treated beyond progression per RECIST v1.1 [redacted] *may* continue treatment until loss of clinical benefit (Section 7.1.2).
- Safety follow-up visit: within 30 to 37 days after the last RO7589831 dose.
- LTFU: After the safety follow-up visit, participants will be followed up with every 90 (± 14) days after the last RO7589831 dose to *collect* subsequent anti-cancer therapies, and survival status.

4.5 END OF STUDY DEFINITION

The end of the study is defined as the date at which the last data point required from the last participant is received (last participant, last observation), or 1 year after the end-of-treatment (EoT) visit of the last participant enrolled into the study, whichever is earlier.

For an individual participant, the completion of the study (i.e., the last visit) will occur when the participant withdraws consent, has been lost to follow-up, dies, or when the study is stopped.

Because of the exploratory nature of this clinical study, any part of its conduct can be discontinued at any time at the discretion of the Sponsor. The Sponsor will notify the Investigators and Health Authorities if the study is discontinued and/or the development program is terminated.

5. STUDY POPULATION

The study population rationale is provided in Section 4.2.1.

The participants in this study will be patients with:

- Any MSI and/or dMMR advanced solid tumors (Part I)
- MSI and/or dMMR advanced CRC [redacted]

Use of this document is governed by the terms of use on the first page of this document.

- Any MSI and/or dMMR advanced solid tumors except for CRC [redacted]

Details on the participant population enrolled [redacted] will be added via a protocol amendment.

Prospective approval of protocol deviations, also known as protocol waivers or exemptions, is not permitted.

5.1 INCLUSION CRITERIA

Patients are eligible to be included in the study only if all of the following criteria apply:

1. Willing and able to provide written informed consent for the trial
2. Be ≥ 18 years of age on the date of signing informed consent
3. Life expectancy of ≥ 12 weeks
4. Eastern Cooperative Oncology Group (ECOG) performance-status score of 0 or 1
5. Have a MSI and/or dMMR, histologically or cytologically documented advanced (unresectable and/or metastatic):
    - Solid tumor (Part I only), [redacted] and have received and then progressed following or are intolerant to at least 1 standard treatment regimen in the advanced setting including standard chemotherapy or checkpoint inhibitors according to tumor type. Where checkpoint inhibitor is available as the standard of care, all patients should have received and progressed on or following a PD-1 or a PD-L1 inhibitor for the treatment of metastatic (Stage IV) or locally advanced disease, unless such therapies contraindicated for that patient. Reason for contraindication should be documented.
    - [redacted]
        - [redacted]
        - [redacted]
    - Patients will be enrolled based on *documented* MSI and/or dMMR tumor status determined as part of the routine diagnostic workup in a certified laboratory. *MSI and/or dMMR tumor status may also be assessed in a certified laboratory with a*

Use of this document is governed by the terms of use on the first page of this document.

test that is compliant with applicable requirements or as part of an approved performance study with an investigational IVD if applicable. MSI and/or dMMR tumor status will be defined as:

a) dMMR status *as* assessed by immunohistochemistry (IHC) for MMR protein expression (MLH1, MSH2, MSH6, PMS2) where the loss of 1 or more proteins indicates dMMR or

b) MSI phenotype as determined by polymerase chain reaction *(PCR)* or by tissue next-generation sequencing (NGS)

- Based on emerging data, patients may be enrolled based on MSI tumor status only

6. Availability of formaldehyde-fixed paraffin-embedded (FFPE) archival tumor tissue for submission to Sponsor/central laboratory

- Confirmation of available archival tumor tissue sample is required during screening.
- For participants without archival tissue, a biopsy from either primary or metastatic tumor lesion, deemed medically feasible, must be taken.

7. Presence of measurable disease according to Response Evaluation Criteria in Solid Tumors (RECIST) v1.1:

- Previously irradiated lesions can be considered as target lesions only if progressive disease has been unequivocally documented at that site since radiation.
- If a target lesion is selected for biopsies, the target lesion must be of a size that allows RECIST v1.1 assessments over the course of the study.

8. For Part I backfill cohorts only:

- Accessible lesion(s) that permit at least two biopsies (pre-treatment and on treatment) without unacceptable risk of a significant procedural complication. Biopsies will only be conducted if deemed clinically safe and feasible by the Investigator
- *If multiple lesions are available, it is preferable to obtain the on-treatment biopsy from the same lesion (or organ) as the pre-treatment biopsy, to avoid introduction of heterogeneity related to site of metastasis.*
- *Based on emerging data, enrollment in the backfill cohorts might be restricted to certain tumor types*

9. Adequate hematologic and end-organ function, defined using the following laboratory results obtained within 14 days prior to first dose of study treatment:

- Absolute neutrophil count $\geq 1.5 \times 10^9$/L without granulocyte colony-stimulating factor support. Patient with Benign Ethnic Neutropenia (Atallah-Yunes et al. 2019) with ANC $\geq$ 1000 are eligible.

Use of this document is governed by the terms of use on the first page of this document.

- Platelet count $\geq 75 \times 10^9$/L without transfusion (within 14 days prior to first dose of study treatment).
- Hemoglobin $\geq$ 9 g/dL without transfusion (within 14 days prior to first dose of study treatment).
- Total bilirubin $\leq 1.5 \times$ ULN (except patients with Gilbert syndrome).
  - Note: participants with Gilbert's syndrome will not be excluded from the study. The diagnosis of Gilbert's syndrome is suspected in people who have persistent, slightly elevated levels of unconjugated bilirubin levels ($\leq 3 \times$ ULN) without any other apparent cause.
- Aspartate transaminase (AST) and alanine aminotransferase (ALT) $\leq 2.5 \times$ ULN. Participants with documented liver metastases: AST and ALT $\leq 5 \times$ ULN.
- Creatinine clearance ≥ 50 mL/min based on the Cockcroft-Gault estimation.
- For participants not receiving therapeutic anticoagulation: international normalized ratio (INR) or activated partial thromboplastin time (aPTT) $\leq 1.5 \times$ ULN
- Patients receiving therapeutic anticoagulation should be on a stable dose for at least 1 week prior to Cycle 1 Day 1 (C1D1)

10. Adequate cardiovascular function:
    - New York Heart Association (NYHA) Heart Failure Class $\leq$ 2. Patients with NYHA Class 2 must have left ventricular ejection fraction $\geq$45%.
    - Resting systolic blood pressure $\leq$ 150 mmHg and diastolic blood pressure 100 mmHg (average of $\geq$ 3 readings on $\geq$ 2 sessions with short break between sessions) (or no clinically significant hypertension).
    - Resting heart rate (HR) between 45 to 100 bpm (or no clinically significant tachycardia).

Contraception Requirements

The contraception and abstinence requirements are intended to prevent exposure of an embryo to the study treatment. The reliability of sexual abstinence for male and/or female enrollment eligibility needs to be evaluated in relation to the duration of the clinical study and the preferred and usual lifestyle of the participant. Periodic abstinence (e.g., calendar, ovulation, symptothermal, or post ovulation methods) and withdrawal are not acceptable methods of preventing pregnancy.

11. Participants of childbearing potential (POCBP):
    - A POCBP is eligible to participate if they are not pregnant (Appendix 5), not breastfeeding, and at least one of the following conditions applies:
      - A participant of non-childbearing potential (PONCBP), as defined in Appendix 5
      - A POCBP, who

Use of this document is governed by the terms of use on the first page of this document.

- Agree to remain abstinent (refrain from heterosexual intercourse) or use 2 non-hormonal contraceptive methods including at least one method that results in a failure rate of < 1% per year during the treatment period and for at least *1* month after the final dose of RO7589831 [redacted] POCBP must refrain from donating eggs during this same period. Biological female participants must refrain from donating eggs during this same period. The Investigator should evaluate with the participant the best contraceptive method based on anatomy and physiology.
- Examples of contraceptive methods with a failure rate of < 1% per year include bilateral tubal occlusion/ligation, male sexual partner who is sterilized, and copper intrauterine devices. Barrier methods (male or female condom with or without spermicide, cap, diaphragm or sponge with spermicide) are not highly effective contraceptive methods and must be used alongside a highly effective method. Hormonal contraceptive methods are not considered acceptable (Appendix 5).

12. Biological Male PONCBP:
    - During the treatment period and for at least *1* month after the final dose of RO7589831 [redacted] agreement to:
      – Remain abstinent (refrain from heterosexual intercourse) or use contraceptive measures such as a condom with a *female* partner *of childbearing potential* as defined in Appendix 5. The Investigator should evaluate with the participant the best contraceptive method based on anatomy and physiology.
      – With pregnant female partner, remain abstinent (refrain from heterosexual intercourse) or use contraceptive measures such as a condom to avoid exposing the embryo.
      – Refrain from donating semen.

5.2 EXCLUSION CRITERIA

Patients are excluded from the study if any of the following criteria apply:

Medical Conditions

1. Inability or unwillingness to swallow pills.
2. Malabsorption syndrome or other condition that would interfere with enteral absorption.
3. Known hypersensitivity or intolerance to ingredients from the study drug formulation including patients with rare genetic disorders such as galactosaemia, glucose-galactose intolerance or congenital lactase deficiency.

Use of this document is governed by the terms of use on the first page of this document.

4. Known uncontrolled central nervous system (CNS) metastases (progressing or requiring anticonvulsants or corticosteroids for symptomatic control) and/or carcinomatous meningitis.
   - Participants with previously treated brain metastases may participate provided they are stable (without evidence of progression by imaging for at least 4 weeks prior to the first dose of study treatment and any neurologic symptoms have returned to baseline), have no evidence of new or enlarging brain metastases, are clinically stable off corticosteroids for at least 14 days prior to study treatment and have measurable disease, per RECIST v1.1, outside the CNS. This exception does not include carcinomatous meningitis, as subjects with carcinomatous meningitis are excluded regardless of clinical stability.
5. Known active or uncontrolled bacterial, viral, fungal, mycobacterial (including but not limited to tuberculosis and atypical mycobacterial disease), parasitic, or other infection (excluding fungal infections of nail beds), or any major episode of infection requiring treatment with intravenous antibiotics or hospitalization within 2 weeks prior to the start of drug administration (related to the completion of the course of antibiotics, except if for tumor fever) or 6 months for any intracranial abscess.
   - Participants receiving prophylactic antibiotics (e.g., to prevent a urinary tract infection or chronic obstructive pulmonary disease exacerbation) are eligible for the study. Note: Antibiotics known to be moderate and strong CYP3A inhibitors or inducers are not allowed.
6. Has a positive test for hepatitis B virus (HBV; defined as positive for hepatitis B surface antigen or HBV DNA) or hepatitis C virus (HCV; defined as positive for HCV antibody or HCV RNA) per local diagnostic standard and in accordance with local laws and regulations, indicating acute or chronic infection.
7. Positive human immunodeficiency virus (HIV) test at screening per local diagnostic standard and in accordance with local laws and regulations (unless testing is not allowed by local Health Authority) or taking protease inhibitors as antiretroviral therapy for HIV.
8. Uncontrolled diabetes or symptomatic hyperglycemia (i.e., well controlled defined as a screening hemoglobin A1c $<$ 8% and no urinary ketoacidosis)
9. Significant cardiovascular/cerebrovascular disease within 6 months prior to D1 of study drug administration, including any of the following:
   - Hypertensive crisis/encephalopathy
   - Unstable angina
   - NYHA Class III or IV cardiac disease or congestive heart failure requiring medication.
   - Serious cardiac arrhythmia requiring treatment (exceptions are atrial fibrillation, paroxysmal supraventricular tachycardia)
   - Baseline-corrected QT (through use of Fridericia's formula [QTcF]) interval $>$470 ms based on average of the screening triplicate 12-lead ECG.

Use of this document is governed by the terms of use on the first page of this document.

- History of thromboembolic events (such as myocardial infarction, stroke or pulmonary embolism)
    - *Patients with history of pulmonary embolism may be eligible if event is considered resolved in the opinion of the Investigator and as per local guidelines.*

10. History of malignancy other than the one under investigation, within 2 years prior to screening, with the exception of:
    - Malignancies with a negligible risk of metastasis or death (e.g., 5-year overall survival (OS) rate > 90%) and no anticipated requirement for anti-cancer therapy during the duration of the study, such as adequately treated carcinoma in situ of the cervix, non-melanoma skin carcinoma, localized prostate cancer (Gleason score Grade < 7), ductal carcinoma in situ, or Stage I uterine cancer.
    - *Patients with multiple malignancies in the past or at the screening due to Lynch syndrome are not excluded.*
11. Alcohol or drug dependence or abuse
12. Patients with known WRN syndrome
13. Any medical condition or abnormality in clinical laboratory tests that, in the Investigator's judgement, precludes the participant's safe participation in and completion of the study, or which could affect compliance with the protocol or interpretation of results.

Prior and Concomitant Therapy

14. Radiotherapy or major surgical procedure other than for diagnosis within 3 weeks prior to initiation of study treatment, or anticipation of need for a major surgical procedure during the study. Participants should be recovered from the effects of radiation and/or surgery.
    - Palliative radiotherapy is allowed if completed within 2 weeks prior to start of study treatment.
15. Prior treatment with any WRN helicase inhibitor
16. Systemic anti-cancer therapy *beside the one allowed in the protocol,* including but not limited to chemotherapy, investigational drugs, hormonal therapy and antibody-based agents (including checkpoint inhibitors) within 28 days or at least 5 half-lives, whichever is shorter, prior to start of study treatment. [redacted]
17. Treatment-related toxicities from prior cancer therapy that have not resolved to Grade ≤ 1 CTCAE prior to study treatment except for:
    - Immune-related AEs such as hypothyroidism and adrenal insufficiency on a stable dosage of hormone replacement therapy
    - The following Grade 2 toxicities: alopecia, peripheral neuropathy, any laboratory changes that still lie within the inclusion criteria defined above

Use of this document is governed by the terms of use on the first page of this document.

18. Treatment with moderate or strong CYP3A4 inducers within 14 days prior to initiation of study treatment (see Appendix 14)
19. Treatment with moderate or strong CYP3A4 inhibitors within 7 days prior to initiation of study treatment (see Appendix 14)

Other Exclusions

20. Pregnancy, breastfeeding, or intention of becoming pregnant during the study

    Note: POCBP must have a negative serum pregnancy test result within 1 week prior to initiation of study treatment and have a negative urine or serum pregnancy test at C1D1 predose (Appendix 4 Table 1).

[redacted]

21. [redacted]
    - [redacted]
    - [redacted]
    - [redacted]
        - [redacted]
        - [redacted]
        - [redacted]
22. [redacted]
23. [redacted]
        - [redacted]

Use of this document is governed by the terms of use on the first page of this document.

- [redacted]

5.3 Lifestyle Considerations

Participants are expected to adhere to the protocol requirement regarding contraception, but there are no other lifestyle restrictions during the study (for meals and dietary restrictions see Section 5.4).

[redacted]

5.4 Meals and Dietary Restrictions

Participants must not eat grapefruit or drink grapefruit juice from 48 hours before the start of study treatment until after the final dose unless otherwise specified.

5.4.1 <u>For Participants enrolled for Food Effect assessment</u>

[redacted]

[redacted]

[redacted]

[redacted]

[redacted]

[redacted]

Use of this document is governed by the terms of use on the first page of this document.

5.4.2 Alcohol

Participants must abstain from alcohol for 24 hours before scheduled study visit day. During the study, participants are requested to moderate their alcohol intake. Participants will be queried on a regular basis about their alcohol consumption and appropriate comments concerning this intake will be recorded on the electronic case report form (eCRF).

5.5 RECRUITMENT PROCEDURES

To be eligible for enrollment, patients must fulfill all the eligibility criteria described in Section 5.1 and Section 5.2. Prospective approval of protocol deviations, also known as protocol waivers or exemptions, is not permitted.

In case of uncertain or questionable results, any of the tests performed during screening may be repeated before study drug administration to confirm eligibility (or clinical significance).

5.6 SCREEN FAILURES

Screen failures are defined as participants who consent to participate in the study but are not subsequently enrolled in the study.

The Investigator will maintain a screening log to record details of all participants screened and to confirm eligibility or record reasons for screening failure.

If a participant is not enrolled into the study, any tumor sample that was sent to the Sponsor will be returned to the site (preferably, all screening assessments should be confirmed before an archival or biopsy tumor tissue is collected).

Re-screening:

- Participants who do not meet the criteria for participation in this study may be re-screened.
- Re-screening is also allowed for participants who were screened in the study and met study inclusion/exclusion criteria but failed to be enrolled within the screening period because of an administrative reason.
- For re-screened participants, all inclusion and exclusion criteria should be re-evaluated and all applicable screening assessments repeated if not within the allowed screening window before start of study treatment. The following exceptions apply:
  - Archival tumor tissue collection and shipment does not need to be repeated if tumor tissue was already collected and shipped, or is available for shipment, to the Sponsor
  - Fresh tumor tissue biopsy does not need to be repeated if done and if documented during prior screening.

Use of this document is governed by the terms of use on the first page of this document.

Note: For participants in the Backfill cohorts with mandatory paired biopsy and for participants who consented to optional paired biopsy, tumor biopsy does not need to be repeated if it was collected as required per protocol (Section 8.7.1.3) and no systemic anti-cancer treatment was given after the tissue acquistion.

- Re-screened participants will be assigned a different participant number from the one assigned at the initial screening.

6. TREATMENTS

Study treatment is defined as any investigational product (including placebo) or marketed product intended to be administered to a study participant according to the study protocol.

The *I*nvestigational *M*edicinal *P*roduct*s* (IMP*s*) for this study *are* RO7589831 *and pembrolizumab*. Appendix 6 identifies the IMP*s* for this study.

RO7589831 administration will take place both at the study center under *the* supervision of qualified site staff or self-administered at the participant's home or at a different location. [redacted]

Cases of overdose, medication error, drug abuse, or drug misuse, along with any associated AEs, should be reported as described in Appendix 2.

6.1 TREATMENTS ADMINISTERED

The treatments administered are summarized in Table 11. Guidelines for *study treatment administration,* dosage modification and treatment interruption or discontinuation are provided in Section 6.4, 6.6 *and* Section 7. *For pembrolizumab, see the local prescribing information for more details.*

Refer to the RO7589831 Investigator's Brochure and pharmacy manual for more details.

Table 11 Summary of Treatments Administered

**Study treatment name**	RO7589831	*Pembrolizumab*
**Use**	Experimental	*Experimental*
**Dose formulation**	Film-coated tablet	*Refer to local prescribing information*
**Unit dose strengths**	[redacted]	
**Dose and regimen**	Starting dose will be [redacted] [a]	[redacted]

Use of this document is governed by the terms of use on the first page of this document.

**Route of administration**	Oral	*IV infusion*
**Source**	Provided centrally by the Sponsor	*Provided centrally by the Sponsor or locally by the trial site, subsidiary, or designee*
**Packaging and labeling**	Study treatment will be provided in HDPE bottles as a finished kit. Each HDPE bottle will be labeled as required per country requirement.	*Study treatment will be provided in a vial. Each vial will be labeled as required per country requirement.*

[redacted] HDPE=high-density polyethylene; *IV=intravenous;* PK=pharmacokinetic; [redacted]

[a] Regimen and schedule may be adapted (e.g. to [redacted] *or* [redacted]) based on the PK profile and tolerability observed during the course of the dose escalation (Part I)

6.2 PREPARATION, HANDLING, STORAGE AND ACCOUNTABILITY

The packaging and labeling of the IMP will be done in accordance with the Sponsor's standard and local regulations. The IMP packaging will be overseen by the Sponsor's clinical study supplies department and bear a label with the identification required by local law, the protocol number, drug identification, and dosage.

The study site must follow all instructions included with each shipment of IMP. The Investigator or other authorized personnel (e.g., pharmacist) will acknowledge receipt of study treatment and confirm the shipment condition and content. Any damaged shipments will be replaced. The Investigator or designee must confirm that appropriate temperature conditions have been maintained during transit for all IMP received and that any findings, product complaints, or discrepancies have been reported and resolved before use of the IMP. All IMP must be stored in a secure, environmentally controlled, and monitored (manual or automated) area in accordance with the labeled storage conditions, with access limited to the Investigator and authorized staff.

Only participants enrolled in the study may receive IMP.

The Investigator is responsible for IMP accountability (i.e., receipt, reconciliation, and final disposition records). The qualified individual responsible for dispensing the study treatment will dispense the IMP according to the numbers issued by the interactive (voice/web) response system (IxRS). The authorized study personnel (e.g., pharmacist) will maintain records of IMP delivery to the site, IMP inventory at the site, study treatment use by each participant, disposition or return of unused IMP. This will enable reconciliation of all IMP received and ensure that participants are provided the doses which are specified in the protocol.

The IMP will either be disposed of at the study site according to the study site's institutional standard operating procedure (SOP) or returned to the Sponsor with the appropriate documentation. Local or institutional regulations may require immediate

Use of this document is governed by the terms of use on the first page of this document.

destruction of used IMP for safety reasons. The site must obtain written authorization from the Sponsor before any IMP is destroyed, and IMP destruction must be documented on the appropriate form. Accurate records of all IMP received at, dispensed from, returned to, and disposed of by the study site will be recorded on the drug accountability log.

Refer to the pharmacy manual and/or the RO7589831 Investigator's Brochure *or local prescribing information for pembrolizumab* for information on study treatment formulation, study treatment handling (including preparation and storage), and accountability.

6.3 MEASURES TO MINIMIZE BIAS: RANDOMIZATION AND BLINDING

6.3.1 Method of Treatment Assignment

This is an open label study. All participants must provide written informed consent before any study-specific assessments or procedures are performed. Sites will be required to notify the Sponsor of planned participant screening to manage recruitment. A set of screening numbers will be provided to each site by the Sponsor.

The Investigator will ensure that each participant fulfills the protocol's inclusion/exclusion criteria prior to enrollment. The decision to enroll the participant in the study is the responsibility of the Investigator. The Medical Monitor is available to the Investigator to advise and answer any questions related to study inclusion or exclusion criteria and may share risk information pertinent to the participant.

An Eligibility Screening Form (ESF) documenting the Investigator's assessment of each screened participant with regard to the protocol's inclusion and exclusion criteria is to be completed. The ESF will also capture the allocated screening number for the participant.

Participants who meet all the eligibility criteria will be assigned the next available slot in consultation with the Sponsor and sites will contact the IwRS to confirm enrollment and obtain a participant number.

During Part I dose escalation participants will be assigned to dose cohorts in the order in which they are enrolled. If dose escalation cohorts and backfill cohorts are open in parallel, the Sponsor will decide where to enroll each participant. If two or more backfill cohorts are open in parallel, enrollment into these cohorts *may* be randomized.

[redacted] If implemented, the randomization may be stratified with important prognostic factor(s), which will be identified based on the observations in Part I.

Use of this document is governed by the terms of use on the first page of this document.

[redacted]

Full details regarding the use of IwRS will be provided to sites.

Under no circumstances will participants who enroll and receive study medication be permitted to be allocated to a new enrollment number and re-enroll in this study.

6.3.2 Blinding

This is an open-label study and blinding procedures are not applicable.

6.4 TREATMENT *ADMINISTRATION AND* COMPLIANCE

The qualified individual responsible for dispensing the study treatment will prepare the correct doses according to the enrollment schedule. This individual will write the date dispensed and participant number on the study treatment bottle label and on the Drug Accountability Record. This individual will also record the study treatment number received by each participant during the study.

For RO7589831 doses to be administered at home, a sufficient number of tablets should be dispensed to the participant to last until the next visit, or at the Investigator's discretion, through one cycle, unless otherwise stated (Section 1.3). Participants will self-administer RO7589831 as detailed below, except on study visit days when RO7589831 will be administered in the clinic and participants will be instructed to hold the morning dose of RO7589831 until after blood samples have been obtained for PK and/or laboratory assessments and all the required assessments are performed as per the SoA (Section 1.3).

Participants should take RO7589831 at approximately the same time each day [redacted]

To avoid missing a day's dose(s), a variance of:

- *Up to* [redacted] *for* [redacted] *dosing either way is allowed for any given dose, making sure that at least* [redacted] *have passed between two subsequent doses.*
- [redacted] *for* [redacted] *dosing either way is allowed for any given dose, making sure that at least* [redacted] *have passed between two subsequent doses.*
- [redacted] *for* [redacted] *dosing either way is allowed for any given dose, making sure that at least* [redacted] *have passed between two subsequent doses.*

RO7589831 should be taken with a minimum of 240 mL (e.g., 8 fluid ounces) of water unless otherwise instructed, RO7589831 should preferably be taken on an empty

Use of this document is governed by the terms of use on the first page of this document.

stomach, i.e., food intake avoided at least 2 hours before as well as 1 hour after the dose is administered. *Participants should also be instructed to swallow the whole trial medication and not chew the tablet. No tablet should be ingested if it is broken, cracked, or otherwise compromised.*

If a participant vomits up a tablet, they should be instructed to skip that dose (or dose portion), document it in participant's drug diary, and resume dosing with the next scheduled dose. Missed doses or tablets will not be made up.

Participants will be instructed as to the number and strength of tablets to take, according to their assigned dose level and schedule. Participants will be asked to record the time and date that they take each dose in a medication diary.

Participants will be instructed to bring their medication diary to each study visit for assessment of compliance. Participants will also be instructed to bring all unused tablets to each study visit or, at the Investigator's discretion, at the end of each cycle, for drug accountability.

Treatment compliance will be defined as the number of tablets taken divided by the expected number of tablets and reported as a percentage. In case of dose reductions of RO7589831, the number of tablets should reflect the new dose level.

Tablets that are not returned will be considered to have been taken, unless otherwise specified in the participant's eCRF.

Guidelines for dosage modification and treatment interruption or discontinuation are provided in Section 6.6 and Section 7.1.

6.4.1 *Administration of Pembrolizumab*

[redacted]

[redacted]

[redacted]

Use of this document is governed by the terms of use on the first page of this document.

[redacted]

6.5 CONCOMITANT THERAPY

All treatments that the Investigator considers necessary for a participant's welfare may be administered at the discretion of the Investigator in keeping with the community standards of medical care. Any medication or vaccine *or blood product* (including over-the-counter (OTC) or prescription medicines, approved dietary and herbal supplements, nutritional supplements) used by a participant within 30 days prior to screening until the safety follow-up visit must be recorded on the Concomitant Medications eCRF along with reason for use, dates of administration (including start and end dates) and dosage information (including dose and frequency).

If changes occur during the trial period, documentation of drug dosage, frequency, route, and date may also be included on the eCRF.

All medication administered to manage AEs should be recorded on the Adverse Event eCRF. The Medical Monitor should be contacted if there are any questions regarding concomitant or prior therapy.

6.5.1 Rescue Medicine

Not applicable.

6.5.2 Permitted Therapy

Participants who use oral contraceptives (see Section 5.1 and Appendix 5), hormone-replacement therapy, or other maintenance therapy should continue their use.

Participants treated with anti-seizure medications or warfarin should have medication levels or appropriate laboratory tests monitored regularly. Refer to Section 6.5.3 for applicable restrictions.

Bisphosphonate and denosumab therapy for bone metastases or osteopenia/osteoporosis is allowed. Patients on oral bisphosphonate therapy should be without signs of esophagitis.

Pain medications administered per standard clinical practice are acceptable while the patient is enrolled in the study.

Limited field palliative radiotherapy (e.g., treatment of known bony metastases or symptomatic relief of pain) or elective surgery during the clinical study may be permitted after discussion with the Medical Monitor.

Use of this document is governed by the terms of use on the first page of this document.

- For participants enrolled in Part I, palliative radiotherapy should be deferred until completion of the DLT observation window.
- Palliative radiotherapy is permitted after the DLT observation window, provided it does not interfere with the assessment of tumor target lesions (e.g., the lesion to be irradiated must not be *target lesions*).
- *Participants who experience infusion-associated symptoms or other AEs should be treated symptomatically following the institutional standard, pembrolizumab USPI and/or E.U. SmPC or the equivalent country-specific document.*

6.5.2.1 Medications Given with Precaution or with a Time Window Restriction

RO7589831 was shown to be a moderate CYP3A and CYP2B6 inducer in vitro. Medications (for example steroids and contraceptives) predominantly metabolized via CYP3A or CYP2B6 may have decreased efficacy following concomitant administration of RO7589831. Sensitive CYP3A or CYP2B6 substrates should be used with precaution, or selection of an alternate concomitant medicinal product should be considered. A list of such medications is shown in Appendix 14, Table 1. Further information on drugs that are CYP substrates, inhibitors and inducers can be found at the FDA Table of Substrates, Inhibitors and Inducers. The Investigator should consult the prescribing information for any concomitant medication when determining whether a certain medication is a sensitive CYP3A or CYP2B6 substrate.

RO7589831 might inhibit P-glycoprotein (P-gp) in the gastrointestinal tract during its absorption. Comedications known to be sensitive P-gp substrates of narrow therapeutic index should therefore be administered at least 2 hours before or 6 hours after RO7589831 administration to minimize the risk of increased exposure of these co-medications. For a list of co-medications known to be sensitive P-gp substrates, refer to Appendix 14, Table 2. *In the event that* [redacted] *dosing would be implemented, drugs known to be sensitive P-gp substrates of narrow therapeutic index should not be coadministered.*

RO7589831 is partially metabolized via glutathione adduction, a similar pathway as is known for paracetamol. Although no drug-drug interaction is expected, as a precautionary measure paracetamol should not be administered above the locally recommended maximum daily dose.

Chemotherapy (e.g., methotrexate) required for autoimmune disease is to be avoided during the DLT assessment window and caution should be exercised when given outside the DLT window (RO7589831 Investigator's Brochure).

6.5.2.2 Administration of Vaccines Including SARS-CoV-2 (COVID-19) Vaccines

In general, non-live vaccines are allowed during the study. However, Investigators should review the vaccination status of potential study participants before being

Use of this document is governed by the terms of use on the first page of this document.

considered for this study. Investigators should follow the local disease control and prevention guidelines for adult vaccination with non-live vaccines intended to prevent infectious diseases before study treatment, where possible.

There are no data on potential interactions of non-live vaccines, including SARS-CoV-2 vaccines with RO7589831. Ideally, such vaccination should be completed before a study participant starts study treatment.

In addition, it is strongly recommended not to administer non-live vaccine during the DLT observation window, where possible.

Transient unilateral axillary lymphadenopathy has been observed following messenger RNA-based SARS-CoV-2 vaccination and may confound interpretation of tumor assessment using computed tomography (CT), magnetic resonance imaging (MRI), or PET/CT scans (Cohen et al. 2021, Xu and Lu 2021). While COVID-19 vaccination of study participants should not be delayed, consideration should be given to administering the vaccine at a location that avoids potentially confounding imaging findings, for example, in the contralateral arm for localized cancers or in the thigh. Further, details regarding vaccination such as vaccination date, anatomic site, laterality and vaccine manufacturer (if available) should be collected at the time of imaging to facilitate interpretation.

AEs that may overlap with any potential vaccine-related toxicities should have resolved before participants receive non-live vaccines.

Administration of any vaccine to an ongoing study participant remains the decision of the Investigator, in accordance with their medical assessment of the overall risk-benefit for the individual participant. The Medical Monitor may be contacted for questions regarding administration of non-live vaccines.

6.5.2.3 *Medications Given with Precaution due to Effects Related to Pembrolizumab*

Please refer to the local prescribing information for pembrolizumab (Keytruda USPI; Keytruda E.U. SmPC; equivalent country-specific document). All the medications must be recorded on the Concomitant Medications eCRF or in the Adverse Event eCRF if given for treatment of AEs, or for prophylaxis.

Corticosteroids, Immunosuppressive Medications, and Tumor Necrosis Factor Inhibitors

Systemic corticosteroids, immunosuppressive medications, and TNF inhibitors may attenuate potential beneficial immunologic effects of treatment with pembrolizumab. Therefore, after consultation with the medical monitor, concomitant chronic prednisone (or equivalent) may be used at a dose of ≤10 mg/day to manage pre-existing conditions. Higher doses of prednisone (or equivalent) are permitted for limited duration to treat

Use of this document is governed by the terms of use on the first page of this document.

acute conditions that arise during the study as medically indicated. The medical monitor should be notified of subjects requiring systemic steroids (≥10 mg prednisone or equivalent) for longer than 24 hours for management of a treatment-related AE. Chronic use of inhaled or topical steroids are permitted in the absence of active autoimmune disease.

Systemic corticosteroids or immunosuppressive medications are recommended, at the discretion of the investigator, for the treatment of specific adverse events when associated with pembrolizumab therapy.

6.5.3 Prohibited Therapy

For a comprehensive list of prohibited therapies prior to RO7589831 administration, refer to the exclusion criteria (Section 5.2).

Use of the following concomitant therapies is prohibited during study treatment as described below:

- Investigational therapy (other than protocol-mandated study treatment) is prohibited during study treatment.
- Live attenuated vaccines
- Concomitant therapy intended for the treatment of cancer whether approved by the local Health Authority or experimental, including chemotherapy, radiotherapy, immunotherapy, biologic therapy, herbal therapy, or hormonal therapy except for the following:
  - Hormonal therapy with gonadotropin-releasing hormone (GnRH) agonists or antagonists for endocrine sensitive cancers (e.g., prostate, endometrial, hormone receptor-positive breast cancer) is allowed.
  - Radiotherapy.

    Note: Limited field palliative radiotherapy might be allowed as described in Section 6.5.2.
- Initiation of hematopoietic colony-stimulating factors (CSF) during DLT assessment window.
  - After the DLT assessment window has been completed CSFs may be administered per standard clinical practice.
- Any moderate or strong inhibitor of CYP3A taken within 7 days prior to first study drug administration and up to the safety follow-up visit. A listing of these drugs can be found in Appendix 14 Table 3.
- Any moderate or strong inducer of CYP3A taken within 14 days prior to first study drug administration and up to the safety follow-up visit. A listing of these drugs can be found in Appendix 14 Table 3.
- Taking any nutrients known to modulate CYP3A activity (e.g., grapefruit juice) within two days prior to first study drug administration and up to the safety follow-up visit.

Use of this document is governed by the terms of use on the first page of this document.

[redacted]

Participants who require any of these agents might be discontinued from study treatment after discussion with the Medical Monitor. Participants who are discontinued from study treatment will be followed up as described in Section 7.1.

The above list of prohibited medications/prohibited medications with a time window restriction may not be comprehensive. The Investigator should contact the Medical Monitor if questions arise regarding medications not listed above.

6.6 [redacted]

[redacted]

[redacted]

[redacted]

[redacted]

[redacted]

[redacted]

[redacted]

Use of this document is governed by the terms of use on the first page of this document.

6.6.1 Temporary Interruption

For missed doses or tablets, refer to Section 6.4. For missed dosing in relation to DLTs, see Section 4.1.6

In case of treatment-emergent AEs, treatment with RO7589831 can be interrupted up to 21 consecutive days to allow for resolution of toxicity. If the participant is unable to resume treatment with RO7589831 after more than 21 consecutive days, no additional doses will be administered, and the participant will be withdrawn from study treatment. In exceptional cases, RO7589831 administration may be resumed after treatment interruption of >21 consecutive days if the participant is considered to derive clinical benefit from study treatment in the opinion of the Investigator and after discussion with the Medical Monitor.

In case of treatment interruption not due to treatment-emergent AEs, but solely due to logistic reasons (e.g., access to the site, unscheduled site closures, or public transportation issues) treatment interruption of no longer than 14 days might be allowed pending a consultation with the Medical Monitor. Treatment interruptions are strongly discouraged to ensure participant safety and minimize a detrimental impact on study integrity.

[redacted]

[redacted]

[redacted]

[redacted]

Use of this document is governed by the terms of use on the first page of this document.

6.7 TREATMENT AFTER THE END OF THE STUDY

The Sponsor will evaluate whether to continue to provide RO7589831 to participants after the main study is over, in accordance with the Roche Global Policy on Continued Access to IMP, available at the following website:

http://www.roche.com/policy_continued_access_to_investigational_medicines.pdf

7. DISCONTINUATION OF STUDY, STUDY TREATMENT, AND PARTICIPANT DISCONTINUATION/WITHDRAWAL

An excessive rate of participant withdrawals (either participants discontinuing study treatment or withdrawing from the study) can render the study non-interpretable. Therefore, unnecessary withdrawal of participants should be avoided, and efforts should be taken to motivate participants to comply with all the study-specific procedures as outlined in this protocol.

Details on study and site closures are provided in Appendix 1, Section 4.

7.1 DISCONTINUATION OF STUDY TREATMENT

7.1.1 *For Part I* [redacted]

Participants will be treated until they experience any of the following:

- Disease progression *(see exception for participants undergoing intra-participant dose escalation Section 4.1.6.1)*
- Any medical condition that the Investigator or Sponsor determines may jeopardize the participant's safety if he or she continues in the study, including:
  - Any toxicity which is not manageable by temporary interruption or decrease of the dose of RO7589831 (as allowed by protocol) and/or appropriate treatment
  - Immunoglobulin E-mediated hypersensitivity reactions, including anaphylaxis
- See *Management of Adverse events* Section 8.3.9
- Pregnancy
- Investigator or Sponsor determination that treatment discontinuation is in the best interest of the participant
- Use of another anti-cancer therapy (see Section 6.5.2)
- Participant withdrawal of consent *to continue study treatment*

The primary reason for permanent discontinuation of study treatment should be documented on the appropriate page of the eCRF.

Unless consent for study participation is withdrawn, participants who discontinue from study treatment should remain in the study. See the SoA (Section 1.3) for visits and any further evaluations that need to be completed after discontinuation from study treatment.

Use of this document is governed by the terms of use on the first page of this document.

[redacted]

[redacted]

- [redacted]
  - [redacted]
    - [redacted]
    - [redacted]
- [redacted]
  - [redacted]
  - [redacted]
- [redacted]
- [redacted]
- [redacted]
- [redacted]
- [redacted]
- [redacted]

[redacted]

Use of this document is governed by the terms of use on the first page of this document.

- [redacted]
- [redacted]
- [redacted]
- [redacted]
- [redacted]

[redacted]

7.1.3 Participants Replacement

Participants who discontinue for any of the following reasons may be replaced to ensure adequate numbers of evaluable participants:

- Participants who are discontinued from the study prior to RO7589831 first dose may be replaced.
- Participants who fail to complete their DLT-assessment period or who miss RO7589831 doses for reasons other than a DLT as described in Section 4.1.6.

See also Section 9 for further reasons for participant's replacement.

7.2 PARTICIPANT DISCONTINUATION OR WITHDRAWAL FROM THE STUDY

Participants have the right to voluntarily withdraw from the study at any time for any reason and without justification.

In addition, the Investigator can discontinue a participant from the study for medical conditions that the Investigator or Sponsor determines may jeopardize the participant's safety if the participant continues in the study.

If possible, information on the reason for withdrawal from the study should be obtained. The primary reason for withdrawal from the study should be documented on the appropriate eCRF. Participants will not be followed up with for any reason after consent has been withdrawn. This includes the follow-up assessments.

When a participant voluntarily withdraws from the study, or is discontinued by the Investigator, samples collected until the date of withdrawal/discontinuation will be analyzed, unless the participant specifically requests for these to be discarded or local laws require their immediate destruction. However, if samples have been tested prior to

Use of this document is governed by the terms of use on the first page of this document.

withdrawal, results from those tests will be used as part of the overall research data. A participant's withdrawal from this study does not, by itself, constitute withdrawal of samples donated to the Research Biosample Repository (RBR).

Participants withdrawn from study might be replaced as described in Section 7.1.

For data to be collected at the time of study discontinuation and any further evaluations that need to be completed, see the SoA (Section 1.3).

7.3 LOST TO FOLLOW-UP

A participant will be considered lost to follow-up if the participant repeatedly fails to return for scheduled visits and is unable to be contacted by the study site.

The following actions must be taken if a participant fails to return to the clinic for a required study visit:

- The site must attempt to contact the participant and reschedule the missed visit as soon as possible, counsel the participant on the importance of maintaining the assigned visit schedule and ascertain whether or not the participant wishes to and/or should continue in the study.
- Before a participant is deemed lost to follow-up, the Investigator or designee must make every effort to regain contact with the participant. These contact attempts should be documented in the participant's medical record.
- Should the participant continue to be unreachable, the participant will be considered to have withdrawn from the study.

Discontinuation of sites or of study as a whole are handled per Appendix 1.

8. STUDY ASSESSMENTS AND PROCEDURES

Study procedures and their timepoints are summarized in the SoA (Section 1.3).

Blood samples collection for PK analysis and ECG measurements will preferably be taken at the site; however, home nursing services will be available to allow for flexibility in the schedule of visits and to shorten site visits when the visit length depends on blood sampling. Urine samples should be collected at the site.

Protocol waivers or exemptions are not permitted.

Immediate safety concerns should be discussed with the Sponsor immediately upon occurrence or awareness to determine if the participant should continue or discontinue study treatment.

Procedures conducted as part of the participant's routine clinical management (e.g., blood count, imaging) and obtained before signing of the Informed Consent Form (ICF) may be utilized for screening or baseline purposes provided the procedure met the

Use of this document is governed by the terms of use on the first page of this document.

protocol-specified criteria and was performed within the timeframe defined in the SoA.

Based on continuous analysis of the data in this study, any sample type or biomarker evaluation not considered critical may be stopped at any time if the data from the samples collected do not produce useful information.

Instructions and supply kits will be provided for all central laboratory assessments.

[redacted]

8.1 EFFICACY ASSESSMENTS

8.1.1 Tumor and Response Evaluations

Response assessment will be performed according to the RECIST v1.1 (Appendix 8).

Response Assessment schedule and duration:

Response assessments will be performed at the timepoints defined in the SoA (Section 1.3) and must be performed independently of changes to the study treatment administration schedule (i.e., when treatment is withheld). Each tumor assessment should be conducted according to the original schedule based on the date of first study treatment administration (C1D1) independently of the timing of the previous assessment.

Participants who discontinue study treatment (for any reason, including, but not limited to, clinical decline or toxicity) in the absence of radiographic disease progression per investigator-assessed RECIST v1.1 will continue to undergo tumor response assessments at the same frequency until radiographic disease progression per RECIST v1.1, initiation of subsequent anti-cancer therapy, withdrawal of consent, death, or study termination by the Sponsor, whichever occurs first, if feasible.

At the Investigator’s discretion, tumor assessments may be repeated at any time if progressive disease is suspected.

Response Assessment guidelines:

All known sites of disease must be documented at screening and re-assessed at each subsequent tumor evaluation. Measurable lesions that have been previously irradiated should not be considered as target lesions unless there is documented progression following completion of radiation therapy.

If a target lesion is selected for biopsies, the target lesion must be of a size that allows RECIST v1.1 assessments over the course of the study.

Use of this document is governed by the terms of use on the first page of this document.

CT (or MRI) scans should include chest, abdomen, and pelvic scans, with additional anatomical coverage and/or examinations performed if clinically indicated (e.g., brain scans, scans of the neck, bone scans).

The extent of neoplastic disease will be determined by reproducible radiographic techniques, preferably CT or MRI scan. Ultrasound and x-rays are not acceptable for monitoring target lesions. For each participant, the same method of assessment and the same technique must be used consistently throughout the entire study (e.g., the same contrast protocol for CT scans). If a CT scan for tumor assessment is performed using a PET/CT scanner, the CT should be of identical diagnostic quality as a full-contrast diagnostic CT scan.

Assessments should be performed by the same evaluator, if possible, to ensure internal consistency across visits.

The use of oral and intravenous contrast should, as long as it is clinically possible, be kept consistent. MRIs of the abdomen and pelvis with non-contrast CT scan of the chest may be used in participants for whom CT scans with contrast are contraindicated (i.e. participants with contrast allergy or impaired renal clearance).

At the Investigator's discretion, other methods of assessment of measurable disease as per RECIST v1.1 may be used (Appendix 8).

An objective response will be confirmed by two consecutive assessments at least four weeks apart.

In addition to Investigator assessment, tumor assessment may also be performed centrally by an independent reviewer for prospective and retrospective analysis.

8.1.2 [18F] FDG-PET Imaging

[18F]-FDG-PET/CT will be performed in Part I [redacted] participants at the timepoints defined in the SoA (Section 1.3) to determine drug effect on the tumor. FDG-PET might [redacted] Part I data indicate no further need to continue the quantitative assessment of therapy-induced changes in tumor [18F]-FDG uptake. [redacted]

For participants undergoing tumor biopsy at screening, the screening FDG-PET should be performed either before or at least 7 days after the screening biopsy is obtained.

The first on-treatment FDG-PET must occur before RO7589831 administration to avoid potential interactions of the radiotracer and RO7589831 with a window of up to 5 days before C2D1 *and before the planned* on-treatment biopsy if a biopsy is scheduled.

The second on-treatment FDG-PET scan should be performed up to 5 days before the first on-treatment tumor assessment as per the SoA.

Use of this document is governed by the terms of use on the first page of this document.

The imaging acquisition guidelines should be followed as described in the imaging manual. Briefly, the participant should fast for 4 to 6 hours prior to the FDG-PET investigation. The participant will be resting in the waiting room after the tracer administration to be ready for the scanning to start 60 minutes ± 10 minutes after tracer administration. The participant will be examined with a sufficient number of bed positions to ensure coverage over the area from the lower part of the brain (inclusive) to mid-thigh. It is also essential that the participant be examined under the same conditions at screening and follow-up scans, specifically with respect to uptake time and scanning time. It is recommended that the same model of scanner be used or at least that the same spatial resolution be ensured.

Participants with no evidence of FDG uptake on the screening PET scan will not be required to undergo follow-up scans. For participants with suggestion of progressive disease on follow-up FDG-PET assessments, a confirmatory CT/MRI assessment is strongly recommended.

Metabolic response assessment using FDG-PET will be performed at the investigating sites and centrally by an independent reviewer.

8.1.3 Serologic Tumor Markers

For subjects with tumors where appropriate serologic tumor markers are available *for the tumor type under investigation in this* study (e.g., CEA, PSA, CA-125 etc) these markers will be assessed as described in the SoA (Section 1.3) and per discretion of the Investigator.

8.2 SAFETY ASSESSMENTS

Planned timepoints for all safety assessments are provided in the SoA (Section 1.3).

Safety assessments will consist of monitoring and recording AEs, including serious AEs (SAEs) and AEs of special interest (AESIs); measurement of protocol-specified safety laboratory assessments; measurement of protocol-specified vital signs, ECGs; and other protocol-specified tests that are deemed critical to the safety evaluation of the study.

8.2.1 Physical Examinations

Physical examinations will be done at the timepoints specified in the SoA (Section 1.3).

A complete physical examination will include assessments of the cardiovascular, respiratory, gastrointestinal, dermatological, neurological, and musculoskeletal systems in addition to the head, eyes, ears, nose, throat, neck, and lymph nodes.

Targeted physical examination must include, at a minimum, the following body systems: cardiovascular, gastrointestinal, pulmonary and neurological for participants with brain metastases. Further examination of other body systems may be performed in case of

Use of this document is governed by the terms of use on the first page of this document.

evocative symptoms at the Investigator's discretion. Investigators should pay special attention to clinical signs related to previous serious illnesses.

Changes from baseline abnormalities should be recorded in participant's notes. New or worsened clinically significant abnormalities should be recorded as AEs on the Adverse Event eCRF.

Height and weight will also be measured and recorded at the timepoints specified in the SoA (Section 1.3). Body weight measured within 24 hours from start of RO7589831 infusion at planned timepoint is acceptable.

8.2.2 Vital Signs

Vital signs will be recorded at the timepoints specified in the SoA (Section 1.3).

Vital signs will include measurements of systolic and diastolic blood pressure while the participant is in a sitting or semi-supine position, respiratory rate, pulse rate, and body temperature. Every effort should be made to ensure that vital signs are obtained from participants in a consistent manner/position.

Blood pressure and pulse measurements should be preceded by at least 5 minutes of rest for the participant in a quiet setting without distractions (e.g., television, radio, cellular phones, conversation) and must be measured before the participant is announced the outcome of a response assessment (if applicable).

Vital signs will be taken before blood collection for laboratory tests*, if feasible,* but after ECG collection when scheduled at the same timepoint. At the discretion of the Investigator, measurements can be repeated if the values are abnormal or borderline.

8.2.3 Electrocardiograms

All participants will have centralized ECGs performed in accordance with the ECG manual issued by the vendor. Sites will need to utilize the ECG machine and ancillary supplies provided. Triplicate ECGs will be recorded digitally at the sites by using the standard 12-leads and will be obtained as outlines in the SoA (Section 1.3). Three interpretable ECG recordings (e.g., without artifacts) must be obtained at each timepoint Section 1.3)

Paper copies of ECG tracings will be kept as part of the participant's permanent study file at the site. Digital ECGs will be transmitted from the sites electronically to a central laboratory for a measurement of the cardiac intervals and morphologic assessment by a central cardiologist.

To minimize variability, lead placement should be as consistent as possible and ECG recordings should be performed after the participant has been resting in a supine or semi-supine position for at least 10 minutes, and the participant should remain in a

Use of this document is governed by the terms of use on the first page of this document.

supine or semi-supine position during recording. The same positioning should be maintained for each participant throughout the study. Any irregularity observed or occurring during the ECGs (e.g., vomiting, cough) should either induce a repeat of the ECG or be annotated with the description and time of the occurrence. ECG recordings should be performed prior to other procedures scheduled at that same time (e.g., vital sign measurements, blood draws) and should not be performed within 2.5 hours after any meal *if possible, unless otherwise stated. On days involving multiple ECG assessments spread over a period of many hours, meals should be consumed with moderation to minimize any interference with the ECG measurement*. Circumstances that may induce changes in HR, including environmental distractions (e.g., television, radio, conversation), should be avoided during the pre-ECG resting period and during ECG recording.

In some cases, it may be appropriate to repeat abnormal ECGs to rule out improper lead placement as contributing to the ECG abnormality.

Paper copies of all ECG tracings must be reviewed, annotated to indicate any clinical findings, signed, and dated by a medically qualified member of the site staff. For each timepoint, *the ECG interpretation will be captured, along with any other relevant remarks in the eCRF, as all other parameters are collected centrally*. Any morphologic waveform changes or other ECG abnormalities must be documented on the eCRF.

If any values at or beyond the protocol-defined parameters are detected from the machine reading, an expedited review by the central vendor should be requested to confirm the reading.

In case of discrepancy between central and the Investigator ECG readings, the central reading will be used for trial analysis purposes.

If at a particular postdose timepoint the mean QTcF is $>$ 500 msec or $>$ 60 msec longer than the baseline value (i.e., last value prior to initiation of study treatment), another ECG must be recorded, ideally within the next 5 minutes, and assessed by the Investigator to determine if medical intervention is needed.

8.2.4 Clinical Safety Laboratory Assessments

A list of clinical laboratory tests to be performed is provided in Appendix 4. Clinical laboratory assessments must be conducted in accordance with the local laboratories process and must be conducted in accordance with the SoA (Section 1.3).

Normal ranges for the study laboratory parameters must be supplied to the Sponsor before the study starts and any change must be communicated to the Sponsor within maximum 7 days.

Use of this document is governed by the terms of use on the first page of this document.

The Investigator must review the laboratory report, document this review, and record any clinically relevant changes occurring during the study in the AE section of the eCRF. The laboratory reports must be filed with the source documents. Clinically significant abnormal laboratory findings are those which are not associated with the underlying disease, unless judged by the Investigator to be more severe than expected for the participant's condition.

- In the event of unexplained abnormal clinically significant laboratory test values, the tests should be repeated immediately and followed up until they have returned to the normal range and/or an adequate explanation of the abnormality is found.
- If such values do not return to normal/baseline within a period judged reasonable by the Investigator, the etiology should be identified and the Sponsor notified.
- If laboratory values from non-protocol–specified laboratory assessments performed at the local laboratory require a change in participant management or are considered clinically significant by the Investigator (e.g., SAE, AE, or dose modification), then the results must be recorded in the eCRF.

Results of clinical laboratory testing will be recorded on the eCRF.

Additional blood or urine samples may be taken at the discretion of the Investigator if the results of any test fall outside the reference ranges or clinical symptoms necessitate additional testing to monitor participant safety.

Where the clinical significance of abnormal laboratory results at screening is considered uncertain, screening laboratory tests may be repeated before enrollment to confirm eligibility.

On the basis of continuous analysis of the data in this study and other studies, any sample type not considered to be critical for safety may be stopped via a protocol amendment at any time if the data from the samples collected do not produce useful information.

8.2.5 Eastern Cooperative Oncology Group Performance Status

ECOG Performance Status will be assessed as specified in the SoAs (Section 1.3). It is recommended, where possible, that a participant's performance status will be assessed by the same person throughout the study.

8.2.6 Clinical Outcome Assessments

Patient-reported outcome (PRO) instruments will be completed as specified in the SoAs (Section 1.3) to further characterize the clinical profile of RO7589831. PRO data will be collected through use of the following instruments:

- European Organisation for Research and Treatment of Cancer Quality-of-life Questionnaire- Function 17 (EORTC QLQ-F17)

Use of this document is governed by the terms of use on the first page of this document.

- [redacted]
- Patient-Reported Outcomes Common Terminology Criteria for Adverse Events (PRO-CTCAE)
- European Organisation for Research and Treatment of Cancer Item List 46 (EORTC-IL46; treatment bother item)

There will be no formal review or reconciliation of symptoms reported by participants via PRO-CTCAE. AE reports will not be derived from PRO data by the Sponsor. In addition, the Sponsor will make no attempt to reconcile participant reports of treatment-related symptoms (via PRO-CTCAE) with Investigator reports of AEs. Sites are not expected to review the PRO data for AEs.

The EORTC QLQ-F17 includes the Physical Functioning (PF), Role Functioning (RF), Emotional Functioning (EF), Cognitive Functioning (CF), and Social Functioning (SF) scales as well as the Global Health Status/Quality of Life (GHS/QoL) scale in their original wording from the validated EORTC QLQ-C30 (Aaronson et al. 1993, Fitzsimmons et al. 1999, Appendix 11). It consists of 17 questions with a recall period of the previous week. The functioning items are scored on a 4-point scale that ranges from "not at all" to "very much," and the GHS/QoL items are scored on a 7-point scale that ranges from "very poor" to "excellent." Higher scale scores are reflective of better functioning and health-related quality of life.

[redacted]

The PRO-CTCAE is a validated item library that is used to characterize the presence, frequency of occurrence, severity, and/or degree of interference with daily function of 78 participant-reportable symptomatic treatment toxicities (Basch et al. 2014, Dueck et al. 2015). The PRO-CTCAE contains 124 questions that are rated either dichotomously (for determination of presence vs. absence) or on a 5-point Likert scale (for determination of frequency of occurrence, severity, and interference with daily function). Treatment toxicities can occur with observable signs (e.g., vomiting) or non-observable symptoms (e.g., nausea). The standard PRO-CTCAE recall period is the previous 7 days. A subset of 13 symptoms (i.e., nausea, vomiting, diarrhea, constipation, fatigue, arthralgia, pain, rash, neuropathy, decreased appetite, shortness of breath, concentration, and insomnia) deemed most applicable to the current treatment has been selected for this study (Appendix 10). Participants will also be asked if there are any additional symptoms they have experienced during treatment. Over the course of the

Use of this document is governed by the terms of use on the first page of this document.

study, evaluation of these symptoms may result in additional items being added to the list of symptoms being assessed.

The EORTC-IL46 is a validated single-item questionnaire assessing the overall impact of side effects (see Appendix 11). The standard EORTC-IL46 recall period is during the previous week.

To ensure instrument validity and that data standards meet Health Authority requirements, questionnaires scheduled for administration during a clinic visit will be completed in their entirety by the participant prior to receiving any information on disease status, prior to the performance of non-PRO assessments that could bias participants' answers, and prior to the administration of study treatment, unless otherwise specified.

Patient-reported outcomes should be administered as outlined below:

- Participant health status should not be discussed prior to administration of the instruments.
- Sites must administer the official version of each instrument, as provided by the Sponsor. Instruments must not be copied from the protocol.
- Sites should allow sufficient time for participants to complete the instruments
- Sites should administer the instruments in a quiet area with minimal distractions and disruptions.
- Participants should be instructed to answer questions to the best of their ability; there are no right or wrong answers.
- Site staff should not interpret or explain questions but may read questions verbatim upon request.
- Participants should not obtain advice or help from others (e.g., family members or friends) when completing the instruments.
- Site staff should review all completed instruments and should ask the participant to rectify any response that is not clearly marked in the appropriate location. If a response is missing, site staff should ask the participant to complete the item or confirm that the item was intentionally left blank.

In addition, when possible, it is recommended that the PROs are administered before any other procedures (e.g., blood drawing).

8.2.7 Medical History and Demographic Data

Medical history includes clinically significant diseases, surgeries, cancer history (including prior cancer therapies and procedures), reproductive status, smoking history, and use of alcohol and drugs of abuse, and all medications (e.g., prescription drugs, OTC drugs, herbal or homeopathic remedies, nutritional supplements) used by the participant prior to the screening visit (see Section 6.4.1).

Use of this document is governed by the terms of use on the first page of this document.

In addition, any medical condition (signs, symptoms, and diagnosis) occurring prior to first dose of trial treatment and after the ICF is signed, should be documented in the source documents as medical history (this does not apply to SAEs, see Section 8.3.1). Any medical history/current medical condition that worsens after the first dose of study treatment will be documented as an AE. See additional reporting details in Section 8.3.1.Demographic data will include age, sex, and self-reported race/ethnicity.

8.3 ADVERSE EVENTS AND SERIOUS ADVERSE EVENTS

The definitions of an AE or SAE can be found in Appendix 2. AESIs and disease-related events and/or disease-related outcomes that do not qualify as AEs or SAEs are discussed in Sections 8.3.6 and 8.3.8.

The Investigator and any qualified designees are responsible for ensuring that all AEs (including assessment of seriousness, severity, and causality; see Appendix 2) are recorded on the Adverse Event eCRF and reported to the Sponsor in accordance with instructions provided in this section and in Appendix 2.

Procedures used for recording AEs are provided in Appendix 3.

8.3.1 Period and Frequency for Collecting Adverse Event and Serious Adverse Event Information

The method of recording, evaluating, and assessing causality of AEs and SAEs and the procedures for completing and transmitting SAE reports are provided in Appendix 2.

Investigators will seek information on AEs at each participant's contact. All AEs, whether reported by the participant or noted by study personnel, will be recorded in the participant's medical record and on the Adverse Event eCRF as follows:

After informed consent has been obtained **but prior to initiation of study treatment**, only SAEs caused by a protocol-mandated intervention should be reported (e.g., SAEs related to invasive procedures such as biopsies). Any other any medical condition should be reported as part of medical history (see Section 8.2.7).

After initiation of study treatment, all AEs, regardless of relationship to study treatment, will be reported until the 30 days after the final dose of study treatment [redacted] or until the start of another anti-cancer treatment, whichever comes first.

Post-study AEs and SAEs: The Investigator is not required to actively monitor participants for AEs after the end of the AE reporting period, as defined above.

However, if the Investigator learns of any SAE (including an AE leading to death) that are believed to be related to prior study treatment at any time after a participant has been discharged from the study, and the Investigator considers the event to be

Use of this document is governed by the terms of use on the first page of this document.

reasonably related to the study treatment or study participation, the Investigator must promptly notify the Sponsor. For the procedure of reporting, see Appendix 2.

8.3.2 Method of Detecting Adverse Events and Serious Adverse Events

Care will be taken not to introduce bias when detecting AEs and/or SAEs. Open-ended and non-leading verbal questioning of the participant is the preferred method to inquire about AE occurrence.

A consistent methodology of non-directive questioning should be adopted for eliciting AE information at all participant evaluation timepoints.

8.3.3 Follow-up of Adverse Events and Serious Adverse Events

8.3.3.1 Investigator Follow-Up

The Investigator should follow up on each AE until the event has resolved to baseline grade or better, the event is assessed as stable by the Investigator, the event is otherwise explained, the participant is lost to follow-up (Section 7.3), or the participant withdraws consent. Every effort should be made to follow up on all SAEs considered to be related to study treatment or study-related procedures until a final outcome can be reported.

During the study period, resolution of AEs (with dates) should be documented on the Adverse Event eCRF and in the participant's medical record to facilitate source data verification. If, after follow-up, return-to-baseline status or stabilization cannot be established, an explanation should be recorded on the Adverse Event eCRF.

All pregnancies reported during the study should be followed up on until pregnancy outcome and reported according to the instructions provided in Section 8.3.5.

8.3.3.2 Sponsor Follow-Up

For SAEs, AESI, and pregnancies, the Sponsor or a designee may follow up by telephone, fax, electronic mail, and/or a monitoring visit to obtain additional event details and outcome information (e.g., from hospital discharge summaries, consultant reports, and/or autopsy reports) to perform an independent medical assessment of the reported event.

8.3.4 Regulatory Reporting Requirements for Serious Adverse Events

Immediate notification by the Investigator to the Sponsor of an SAE (regardless of relationship to study treatment) is essential so that legal obligations and ethical responsibilities towards the safety of participants and the safety of a study treatment under clinical investigation are met.

Use of this document is governed by the terms of use on the first page of this document.

The Sponsor has a legal responsibility to notify both the local regulatory authority and other regulatory agencies about the safety of a study treatment under clinical investigation. The Sponsor will comply with country-specific regulatory requirements relating to safety reporting to the regulatory authority, IRB/IEC, and Investigators.

Investigator safety reports must be prepared for suspected unexpected serious adverse reactions (SUSAR) according to local regulatory requirements and Sponsor policy and forwarded to Investigators as necessary.

An Investigator who receives an Investigator safety report describing an SAE or other specific safety information (e.g., summary or listing of SAEs) from the Sponsor will review and then file it along with the Investigator's Brochure and notify the Institutional Review Board (IRB)/ Independent Ethics Committee (IEC), if appropriate according to local requirements.

For immediate (i.e., no more than 24 hours after learning of the event) and expedited reporting requirements from Investigator to Sponsor and from Sponsor to Health Authority, Investigators, IRB and Ethics Committee (EC), see Appendix 2, Section 5.

8.3.4.1 Emergency Medical Contacts

To ensure the safety of study participants, access to the Medical Monitors is available 24 hours per day, 7 days per week. Details will be available separately.

8.3.5 Pregnancy

A POCBP will be instructed to immediately inform the Investigator if they become pregnant during the study or within 6 months after the final dose of study treatment.

Male participants will be instructed through the ICF to immediately inform the Investigator if their partner becomes pregnant during the study or within 4 months after the final dose of study treatment.

If a pregnancy is reported, the Investigator should inform the Sponsor within 24 hours of learning of the pregnancy and follow up via the pregnancy reporting process as detailed in Appendix 5. All pregnancies reported during the study should be followed up until there is a pregnancy outcome, with follow-up information on the infant collected according to procedures outlined in Appendix 5.

Abnormal pregnancy outcomes (e.g., spontaneous abortion, fetal death, stillbirth, congenital anomalies, and ectopic pregnancy) are considered SAEs (Appendix 5).

8.3.6 Adverse Events of Special Interest

AESIs are required to be reported by the Investigator to the Sponsor immediately (i.e., no more than 24 hours after learning of the event; see Appendix 2 for reporting instructions).

Use of this document is governed by the terms of use on the first page of this document.

AESI for this study include the following:

General:

- Cases of an elevated ALT or AST value in combination with either an elevated bilirubin value or clinical jaundice, as defined in Appendix 3.
- Suspected transmission of an infectious agent by the study treatment, defined as:
  - Any organism, virus, or infectious particle (e.g., prion protein transmitting transmissible spongiform encephalopathy), pathogenic or non-pathogenic, is considered an infectious agent. A transmission of an infectious agent may be suspected from clinical symptoms or laboratory findings that indicate an infection in a participant exposed to a medicinal product. This term applies only when a contamination of the study treatment is suspected.

RO7589831-specific:

- None have been identified yet.

8.3.7 Dose-Limiting Toxicities (Immediately Reportable to the Sponsor)

AEs identified as DLTs, as defined in Section 4.1.6.3 are required to be reported by the Investigator to the Sponsor immediately (i.e., no more than 24 hours after learning of the event; see Appendix 2, Section 5 for reporting instructions).

8.3.8 Disease-Related Events and/or Disease-Related Outcomes Not Qualifying as Adverse Events or Serious Adverse Events

Deaths that occur during the protocol-specified AE reporting period (see Section 8.3.1) that are attributed by the Investigator solely to disease progression should be recorded only on the Death Attributed to Progressive Disease eCRF, instead of as AE.

8.3.9 Management of Adverse Events

The information of potential safety concerns is based on anticipated pharmacology and mechanism of action results from non-clinical studies and is provided in the RO7589831 Investigator's Brochure. Toxicities associated or possibly associated with treatment should be managed according to standard medical practice and best medical judgment. Measures will be taken to ensure the safety of participants in this study, including the use of stringent inclusion and exclusion criteria and close monitoring of participants during the study *(Section 5.1, Section 5.2, and Section 8.2)*.

8.3.9.1 General recommendations for the management of RO7589831-related AEs

General guidelines for management of RO7589831 treatment related AE and dose modifications are outlined below in Table 12. Clinical judgement, standard clinical practice as well as institutional guidelines may be applied, and risk/benefit considerations may suggest deviating from these guidelines. In such cases, decisions on

Use of this document is governed by the terms of use on the first page of this document.

study treatments will be taken by the Investigator in consultation with the Medical Monitor.

Table 12 General recommendations for the management of RO7589831-related AEs

Events CTCAE	Action to be taken
Grade 1 events	• Continue RO7589831 treatment
Grade 2 Hematological events	• In general, RO7589831 treatment can be continued
Grade 2 Non-hematological events	• Hold RO7589831 treatment until Grade ≤1<br>• Consider restart at the same dose (1)<br>• If the grade 2 event recur, dose reduction may be considered
Grade 3 Hematological events	• Hold RO7589831 treatment until Grade ≤ 2<br>• Consider restart at the same or a lower dose1 (1)<br>• If the grade 3 event recur, dose reduction may be considered
Grade 3 Non-hematological events	• Hold RO7589831 treatment until Grade ≤1<br>• Consider restart at the same or a lower dose (1)<br>• Dose reduction should be considered for events affecting vital organs (such as lung, cardiac and CNS events)<br>• If the grade 3 events recur, permanently discontinue RO7589831 treatment
Grade 4 events	Permanently discontinue RO7589831 treatment (2)
Confirmed DILI (3)	Permanently discontinue RO7589831 treatment (2)

(1) After a temporary dose interruption, the *RO7589831* treatment may be resumed at the same or at a reduced dose (Section 6.6 and Section 6.6.1).

(2) In general, for *RO7589831* treatment related CTCAE Grade 4 events, *RO7589831* treatment should be permanently discontinued, unless continuing the study treatment is deemed in the best interest of the participant as assessed by the Investigator and after consultation with the Medical Monitor.

(3) Please refer to Section 4.1.6.1 for definition of drug-induced liver injury (DILI)

See the RO7589831 Investigator’s Brochure for further details on the potential risks associated with the use of RO7589831.

[redacted]

Use of this document is governed by the terms of use on the first page of this document.

[redacted]

8.4 TREATMENT OF OVERDOSE

8.4.1 *Treatment of RO7589831 overdose*

Study treatment overdose is the accidental administration of RO7589831 in a quantity that is higher than the assigned dose. An overdose or incorrect administration of study treatment is not an AE unless it results in untoward medical effects (see Appendix 2 Sections 5 and 5.2 for further details). Decisions regarding dose-interruptions or modifications (if applicable) will be made by the Investigator in consultation with the Medical Monitor based on the clinical evaluation of the participant.

In the event of an overdose, the Investigator should:

- Provide supportive care to the participant (if required) per local institutional practices
- Contact the Sponsor's Medical Monitor immediately
- Closely monitor the participant for AE/SAE and laboratory abnormalities until resolved
- Obtain a blood sample for PK analysis as soon as possible
- Document the quantity of the excess dose, as well as the duration of the overdose, in the eCRF

[redacted]

8.5 PHARMACOKINETICS

The PK samples will be collected as outlined in the SoA (Section 1.3) and the plasma concentrations of RO7589831 will be measured via a validated PK assay. *Urine samples may be also collected for some subjects in the study, as outlined in the SOA, and RO7589831 concentrations measured by a specific validated LC-MS/MS assay, or other fit for purpose methods, as appropriate.*

Use of this document is governed by the terms of use on the first page of this document.

Details on sampling procedures, sample storage and shipment are documented in the Study Flow Chart and Laboratory Manual.

All blood samples for PK assessments *can* be collected from an intravenous line *if preferred and feasible*. If multiple samples are drawn at a given timepoint, the PK sample should take precedence.

The date and time of each sample collection will be recorded in the eCRF.

During the course of the study, PK sampling timepoints may be modified on the basis of emerging data to ensure the PK of RO7589831 can be adequately characterized. Any changes in the timing or addition of timepoints for any planned study assessments must be documented and approved by the relevant study team member and archived in the Sponsor's and site study files. This will not constitute a protocol amendment.

Additional PK samples will be taken in case of suspected *RO7589831* overdose, of a DLT or in case of AEs leading reduction or interruption of RO7589831 dose or in case of clinically significant ECG changes (Section 6.6).

Circulating *RO7589831* plasma metabolites *and urinary excreted metabolites* may be measured by a specific validated LC-MS/MS assay, or other fit for purpose methods, as appropriate.

Any volume of blood*, plasma or urine* samples remaining after the specified analyses may also be used for additional validation experiments and PK *or PD* measurements related to this study, e.g., *pembrolizumab concentration measurement,* development of new analytical methods for RO7589831, metabolites of RO7589831 or additional biomarkers of RO7589831 activity.

The PK blood *and urine* samples will be destroyed 2 years after the date of the final clinical study report (CSR).

8.6 BIOMARKERS ANALYSES

The specimens collected in this study will be used for research purposes, *including exploratory biomarker research and biomarker assay development*, to identify biomarkers useful to predict and monitor response to RO7589831 *monotherapy and/or RO7589831 and pembrolizumab combination* treatment, assess TE and PD effects of RO7589831, and investigate potential mechanisms of immune-related response. *Specimens may also be used for determining MMR and/or MSI status for eligibility assessment.*

Details on sample types and collection can be found in Section 8.7.

Use of this document is governed by the terms of use on the first page of this document.

8.6.1 Exploratory Markers for PD Analysis and Response Prediction

Tumor tissue and blood will be analyzed from all participants in the study for exploratory biomarker analysis, including but not limited to assessment of genetic aberrations like MSI and MMR status. Additional analyses may include assessment of abundance and/or location of TILs and other tumor markers. These analyses will inform possible associations of MSI and/or MMR status, baseline immune phenotype and other tumor characteristics with treatment efficacy (Section 8.7.1.1).

Blood samples will be collected as specified in the SoA (Section 1.3). These samples will be tested for blood or DNA derived biomarkers relating to response, PD and mechanism of action of RO7589831 *as monotherapy or in combination with pembrolizumab*. Analysis techniques may include, but are not limited to, ctDNA monitoring by NGS and/or PCR or cytokine measurements. Details on processes for collection and shipment of these samples can be found in the Laboratory Manual and Study Flow Chart.

Paired fresh pre-treatment (screening) and on-treatment biopsies are mandatory for all participants in the Backfill cohorts of Part I and optional during Part I Dose escalation. [redacted] (see Section 8.7). *Biopsies* will be collected as specified in the SoA (Section 1.3). Tissue assessments will include but are not limited to, [redacted] Additional exploratory protein, DNA or RNA based analysis to identify potential response prediction, resistance or PD biomarkers may be performed on residual tissue.

Additional biomarkers may be measured in case a strong rationale develops.

For more details on biomarkers rationale see Section 4.2.2 and Section 4.2.3.

8.6.2 Endogenous CYP3A Activity Marker Analyses

Mandatory blood samples to measure concentrations of 4β–hydroxycholesterol (4β–HC), total cholesterol (TC) and/or 4α–hydroxycholesterol (4α–HC) will be collected to assess the CYP3A activity during RO7589831 treatment. The date and time of each sample collection will be recorded in the eCRF. 4β–HC, TC and/or 4α–HC levels will be analyzed by using validated LC-MS/MS assays. The samples will be taken as outlined in the SoA (Section 1.3). During the course of the study, sampling timepoints may be modified on the basis of emerging data to ensure the CYP3A activity during RO7589831 treatment can be adequately characterized.

Use of this document is governed by the terms of use on the first page of this document.

8.6.3 Genetic and Genomic Analyses

8.6.3.1 Clinical Genotyping

DNA may be used to identify biomarkers including but not limited to exploratory predictive biomarkers, biomarkers of response or resistance to treatment with RO7589831 *as monotherapy or in combination with pembrolizumab*, and will help to better understand the pathogenesis, course, and outcome of the studied cancer types. The DNA may be used for, but analysis is not limited to:

- Genetic variants of drug metabolizing enzymes (e.g. cytochrome P450s, UGTs), transporters or receptors which might affect the metabolism, PK, PD, safety of RO7589831.
- Genetic variants of the WRN helicase gene.
- Genetic variants of pathways related to WRN helicase, dMMR, MSI, CRC or other tumor types included in the study, including but not limited to, genes related to disease/efficacy, safety, PK, or PD parameters of RO7589831.

To identify these variants and polymorphisms, genome-wide methods may be used. Given the complexity and exploratory nature of genome-wide analyses, neither genomics data nor analyses will be shared with Investigators or study participants unless required by law. Participants will not be identified by name or any other personally identifying information. Data arising from all biosamples, including samples for analyses of inherited human DNA, will be subject to the confidentiality standards described in the sample documentation and in Appendix 1, Section 1.4.

8.6.3.2 Targeted DNA Analysis

Samples collected for DNA extraction (see Section 8.7.1) may be used for, but not limited to, the detection of mutations in cancer related genes, MSI, TMB, biomarkers associated with common molecular pathways and exploratory research on genetic biomarkers. The samples and/or their derivatives may be sent to one or more laboratories for analysis of germline variants or somatic mutations.

Analysis of ctDNA extracted from the plasma may include, but is not limited to, detection of molecular residual disease, to monitor response to treatment (PD), to assess potential biomarkers linked to response and/or recurrence. In addition, ctDNA analysis upon relapse may also help researchers to understand potential resistance mechanisms in response to the drug. For this purpose, samples and/or their derivatives may be sent to one or more laboratories for analysis by multiplex PCR or other genomic analysis methods.

Tumor tissue and blood samples will be collected as specified in the SoA (Section 1.3).

Genomics is increasingly informing researchers' understanding of disease pathobiology. Target DNA analysis provides a comprehensive characterization of the genome and, along with clinical data collected in this study, may increase the opportunity for

Use of this document is governed by the terms of use on the first page of this document.

developing new therapeutic approaches. Data will be analyzed in the context of this study but may also be explored in aggregate with data from other studies. The availability of a larger dataset will assist in identification of important pathways, guiding the development of new targeted agents.

Given the complexity and exploratory nature of these analyses, data and analyses will not be shared with Investigators or study participants unless required by law. Participants will not be identified by name or any other personally identifying information. Data arising from all biosamples including samples for analyses of inherited DNA will be subject to the confidentiality standards described in the sample documentation.

8.6.3.3 Transcriptome Analysis

Gene expression profiling may be performed on extracted RNA from tumor tissue collected as outlined in the SoA (Section 1.3) to enable:

- Analysis of exploratory PD biomarkers
- Identification of potential response predictive biomarkers
- Assessment of potential treatment response
- Understand RO7589831 mode of action
- Understand possible mechanisms of escape/resistance

It should be noted that assessment of relevant non-coding RNAs is part of transcriptome analysis.

8.6.4 Epigenetic Analysis

Functionally relevant changes to the genome that do not involve a change in the nucleotide sequence, including but not limited to DNA methylation (e.g., MLH1), may be assessed in tumor tissue.

8.6.5 Proteomics and Metabolomics Analyses

8.6.5.1 Proteome Research

Tissue derivatives may be subjected to proteomic profiling studies using a variety of platforms that could include but are not limited to immunoassay, liquid chromatography/mass spectrometry or alternative equivalent procedure. Univariate and multivariate statistical techniques may be used to identify individual proteins exhibiting statistically significantly different changes in their levels between samples and/or between groups of samples. This will enable the evaluation of changes in proteome profiles that may correlate with biological response relating to dMMR and/or MSI solid tumors and medically related conditions or the action of RO7589831 *as monotherapy or in combination with pembrolizumab*. The samples may also be used to confirm findings by application of alternative technologies.

Use of this document is governed by the terms of use on the first page of this document.

8.6.5.2 Metabolomic Research

No metabolomic research is planned.

8.6.6 Additional Analysis on Blood Samples

Exploratory longitudinal assessment of cytokines, chemokines or markers of inflammation may be performed in blood to characterize treatment-induced events. The analysis of the samples collected will be based on emerging data.

Residual blood samples (e.g., from PK and/or PD assessments) may be used for retrospective and longitudinal testing of SARS-CoV-2 or other bacterial or viral infection by serological methods. This testing may be performed for each participant. In addition to serving as an important safety measure, these analyses will inform as to any association of SARS-CoV-2 or other bacterial or viral infection and response to treatment.

8.7 BIOMARKER SAMPLES

Samples will be collected as specified in the SoA (Section 1.3) from participants throughout the whole study for exploratory biomarker analyses. During the course of the study, sampling timepoints may be modified on the basis of emerging data to ensure the TE and/or PD effect of RO7589831 *as monotherapy or in combination with pembrolizumab* can be adequately characterized (but without increasing overall blood collection volume). In addition, based on continuous analysis of the data in this study and other studies, any sample type and/or analysis not considered to be critical for safety may be stopped at any time if the data from the samples collected does not produce useful information. This will be documented in the Study File and a new SoA will be provided. Details on sampling procedures, sample storage and shipment are provided in the Study Flow Chart and Laboratory Manual. The date and time of each sample collection should be recorded in the eCRF.

Samples may be used *for determining MMR and/or MSI status for eligibility evaluation, if applicable, and for research purposes, including biomarker assay development,* to identify biomarkers useful for predicting and monitoring response to RO7589831 *as monotherapy or in combination with pembrolizumab*, identifying biomarkers useful for predicting and monitoring RO7589831 safety, assessing PD effects of RO7589831 mediated WRN helicase inhibition *as monotherapy or in combination with pembrolizumab* and investigating mechanism of therapy resistance. Additional markers may be measured if a strong scientific rationale develops.

Any remaining blood, tumor tissue or any derivatives of these samples (e.g. cells, tissue sections, and nucleic acids) may also be used for additional exploratory biomarker profiling or/and (assay) validation experiments. Samples may be used for research to develop methods, assays, prognostics, and/or companion diagnostics related to WRN helicase, pathways associated with WRN helicase and its MOA. Unless otherwise specified, all samples (including blood, tissue and any derivatives thereof) will be

Use of this document is governed by the terms of use on the first page of this document.

destroyed no later than 5 years after the date of final CSR unless the participant consented to the RBR; for participants who consent to RBR, leftover samples will be transferred to RBR (Section 8.8).

Details on *the process* for collection and shipment of these samples can be found in the separate sample documentation.

All biosamples and their derivatives, as well as all data arising from these samples, will be subject to the confidentiality standards described in Appendix 1.

8.7.1 Mandatory Samples

The following samples for biomarker analysis are required and will be collected from all participants as per the SoA (Section 1.3).

8.7.1.1 Archival Tumor Samples

FFPE archival tumor tissue is to be obtained from all participants. Tissue must be a core needle, excisional, or incisional biopsy, or resected tissue. Not acceptable tissue includes fine needle biopsies, aspirates, cell pellets, cytospins or biopsies of bone lesions without soft tissue component or decalcified bone tumor samples. The latest tumor tissue sample collected should be provided, preferably acquired after failure/stop of last prior treatment or diagnosis of metastatic disease, or if not available, from the primary tumor. Samples will be used for, but not limited to, analysis of MMR and MSI status as described in Section 8.6. The provision of archival FFPE blocks is preferred, but if not possible, a minimum of 22 consecutive, unstained slides is recommended. If multiple archival tissues are available, the most recent one should be submitted preferably acquired after the most recent disease progression.

If no archival tissue prior to treatment is available a fresh pretreatment tumor biopsy must be obtained. *Also, if the provided archival tumor tissue is not evaluable,* a fresh tumor biopsy, if deemed medically feasible by the investigator, must be obtained (see the Section 8.7.1.3 for requirement of fresh tumor tissue). For enrolled participants, remaining archival tissue blocks will be returned to the site upon request or no later than the time of final closure of the study database, whichever occurs first. Archival tumor blocks will be returned to the sites unless the site explicitly requests for Roche to not return the archival block. In the event the archival block is not returned to the site, blocks will be destroyed at Roche's discretion or transferred to RBR if participant provided consent.

8.7.1.2 Blood Sampling

Blood for ctDNA analysis and/or monitoring will be taken for DNA extraction and ctDNA assessment from every participant at screening, on treatment, and at the time of progressive disease as specified in the SoA (Section 1.3). Mandatory blood samples to measure [redacted]

Use of this document is governed by the terms of use on the first page of this document.

A whole blood sample will be taken for DNA extraction and clinical genotyping from every participant. If the sample is missed on D1, it can be collected at any other scheduled visit.

8.7.1.3 Fresh Tumor Biopsies

Participants enrolled in Part I Backfill cohorts will provide mandatory pre-treatment and on treatment tumor biopsies. [redacted] For a rationale of collection of paired tumor biopsy and collection timepoints, refer to Section 4.2.3 and Section 1.3, respectively.

The pre-treatment biopsy should be collected during screening and, if possible, after participant screening assessments have confirmed participant eligibility.

On treatment biopsies should be collected [redacted] Further details on timepoints are described in the SoA (Section 1.3). The time window of the on-treatment biopsy has been selected to maximize the opportunity to measure TE and MOA related PD changes following RO7589831 administration. PK sample collection is planned before and after the biopsy sample collection to enable PK/PD assessment. If emerging data suggest that a different timepoint would be more appropriate, the on-treatment tumor biopsy timepoint will be modified following the agreement between the Investigator and the Sponsor.

A possible alternative SoA table is presented in Section 1.3 detailing the timing of dosing, PK sample collection and biopsy collection where biopsy samples would be taken prior to drug administration.

Samples collected via core needle biopsy are preferred. Collection of tumor biopsies should be guided by ultrasound, CT scan, or other methods according to the location of the selected lesion to provide cores, ideally, [redacted] Cytological, fine needle aspirations (including endobronchial ultrasound-guided transbronchial needle aspiration) or biopsies of bone lesions are not acceptable.

An assessment of biopsy quality by a pathologist is strongly encouraged at the time of the procedure.

The baseline and on-treatment biopsies should be preferably taken from the same tumor lesion to ensure comparability. The location of each biopsy will be documented in relation to each tumor lesion.

Use of this document is governed by the terms of use on the first page of this document.

If feasible, on-treatment biopsies may be repeated if the initial on treatment biopsy did not contain sufficient tumor material for analysis.

A detailed description of tissue quality requirements and procedures for collection, handling, and shipping of tumor tissue samples will be provided in a separate Laboratory Manual and Study Flow Chart.

8.7.2 Optional Samples

A separate ICF will have to be signed to document a participant's agreement to allow collection of optional samples.

Optional samples for biomarker research that should be collected from participants in the study, are the following:

8.7.2.1 Optional Paired Tumor Biopsies

Pre- and on-treatment tumor tissue will also be collected from participants who have consented to these optional tumor biopsies and are enrolled in Part I Dose Escalation, [redacted] For a rationale of collection of paired tumor biopsies please see Section 4.2.3.

If a participant consents to an optional pre-treatment and on-treatment tumor biopsy, the same timepoints and sample collection procedures described for the mandatory biopsies in Part I Backfill cohorts should be followed (see Section 8.7.1.3).

8.7.2.2 Optional Tumor Biopsy as Clinically Feasible

An optional tumor biopsy may be collected if deemed clinically feasible by the Investigator (e.g., at time of PR, long lasting SD or disease progression) for participants who sign the ICF for the optional biopsy. *A biopsy sample of subjects that have confirmed progression is optional, but strongly encouraged for the purposes of understanding mechanisms of resistance to therapy.* The sample collection procedures and details described in Section 8.7.1.3 should be followed.

If any additional tumor tissues (i.e., biopsy or surgical resection) are collected during the trial, that are part of normal clinical practice, an optional tumor sample might be collected and examined as described in Section 8.6.

8.8 SAMPLES FOR RESEARCH BIOSAMPLE REPOSITORY

8.8.1 Overview of the Research Biosample Repository

The Roche RBR is a centrally administered group of facilities for the long-term storage of human biologic samples, including body fluids, solid tissues, and derivatives thereof (e.g., DNA, RNA, proteins, peptides). The collection, storage, and analysis of the RBR samples will facilitate the rationale design of new pharmaceutical agents and the

Use of this document is governed by the terms of use on the first page of this document.

development of diagnostic tests, which may allow for individualized drug therapy for participants in the future.

Samples collected for RBR can be used (but are not thus limited) to achieve the following objectives:

- To study the association of biomarkers with efficacy or progressive disease.
- To identify safety biomarkers that are associated with susceptibility to developing AEs or can lead to improved AE monitoring or investigation.
- To increase knowledge and understanding of disease biology and drug safety.
- To study treatment response, including drug effects and the processes of drug absorption and disposition.
- To develop biomarker or diagnostic assays and establish the performance characteristics of these assays.

8.8.2 Sample Collection

The following samples will be stored in the RBR and used for research purposes, including, but not limited to, research on biomarkers related to RO7589831:

- Leftover plasma samples
- Leftover blood samples
- Leftover blood samples for DNA extraction
- Leftover tumor tissue (archival and fresh tumor tissue)
- Residual derivatives of above samples

The samples collected for DNA extraction include, but is not limited to, genomic analysis and may be sent to one or more laboratories for analysis of germline or somatic mutations via WGS, whole exome sequencing (WES), or other genomic analysis methods.

Genomics is increasingly informing researcher's understanding of disease pathobiology. WGS provides a comprehensive characterization of the genome and, along with clinical data collected in this study, may increase the opportunity for developing new therapeutic approaches. Data will be analyzed in the context of this study but may also be explored in aggregate with data from other studies. The availability of a larger dataset will assist in identification of important pathways, guiding the development of new targeted agents.

Samples may be sent to one or more laboratories for analysis of germline or somatic mutations via WGS or WES or other genomic analysis methods. Genomics is increasingly informing researchers' understanding of disease pathobiology. WGS and WES provide a comprehensive characterization of the genome and exome, respectively, and – along with clinical data collected in this study – may increase the opportunity for

Use of this document is governed by the terms of use on the first page of this document.

developing new therapeutic approaches or new methods for monitoring efficacy and safety or predicting which participants are more likely to respond to a drug or develop AEs.

Participants will not be identified by name or any other personally identifying information. Data generated from RBR samples will be analyzed in the context of this study but may also be explored in aggregate with data from other studies. The availability of a larger dataset will assist in identification and characterization of important biomarkers and pathways to support future drug development.

If allowed by local laws, the participant may request access to the non-interpreted WGS and WES raw data from blood samples. Such a request must be conveyed to the Sponsor by the Investigator, using the email address: global.return-genomics-results@roche.com. The information, if available at the time of the request, would be shared with the Investigator in the form of a data file of raw genomic sequencing data. The Sponsor will not provide any interpretation of this raw genomic sequencing data. The interpretation of these data will require expertise in genomic and genetic analysis and an understanding of the variability of the technology used for data generation.

For all samples, dates of consent and sample collection should be recorded on the associated RBR page of the eCRF. Details on processes for collection and shipment of these samples can be found in separate sample documentation.

RBR samples will be stored and used until no longer needed or until they are exhausted. The RBR storage period will be in accordance with the IRB/EC‑approved ICF and applicable laws (e.g., Health Authority requirements).

The repository samples will be subject to the confidentiality standards (as described under “Confidentiality” in Appendix 1).

8.9 MEDICAL RESOURCE UTILIZATION AND HEALTH ECONOMICS

Health economic/medical resource utilization and health economic parameters are not evaluated in this study.

8.10 TIMING OF STUDY ASSESSMENTS

8.10.1 Screening and Pre-treatment Assessments

Written informed consent for participation in the study must be obtained before performing any study-specific screening tests or evaluations. ICFs for enrolled participants and for participants who are not subsequently enrolled will be maintained at the study site.

Use of this document is governed by the terms of use on the first page of this document.

All screening and pretreatment assessments (related to entry criteria) must be completed and reviewed by the Investigator to confirm that participants meet all eligibility criteria. The Investigator will maintain a screening log to record details of all participants screened and to confirm eligibility or record reasons for screening failure.

An Eligibility Screening Form documenting the Investigator's assessment of each screened participant with regard to the protocol's inclusion and exclusion criteria is to be completed by the Investigator and kept at the investigational site.

Screening and pretreatment assessments will be performed within 28 days prior to start of study treatment, unless otherwise specified. Results of standard-of-care tests or examinations performed prior to obtaining informed consent and within this period may be used (and do not need to be repeated for screening).

8.10.2 Assessments During Treatment

All assessments must be performed as per the SoA (Section 1.3). Assessments scheduled on the day of study treatment administration should be performed prior to administration of study treatment [redacted] unless otherwise noted in the SoA.

PROs assessment should be performed prior to the completion of other study assessments.

Exceptional measures during the COVID-19 pandemic, such as adjustments in study visits, may be considered (see Section 8.10.6).

Under no circumstances will participants who enroll in this study and have completed treatment as specified be permitted to be allocated a new participant number and re-enroll in the study.

8.10.3 Assessments at End of Treatment Visit

[redacted]

8.10.4 Follow-Up Assessments

After the EoT visit, participants will be asked to return to the clinic within 30 to 37 days after the last dose of study treatment [redacted] for a safety follow-up visit. Participants will then have LTFU visits in person or via phone call every 90 days ± 14 after the last dose of study treatment [redacted]. AEs should be followed as outlined in Sections 8.3.1 and 8.3.3.

Use of this document is governed by the terms of use on the first page of this document.

8.10.5 Assessments at Unscheduled Visits

If clinically indicated, any assessment or sample specified in the SoAs (Section 1.3) can be performed any time as unscheduled assessment or sample at the discretion of the Investigator. Unscheduled eCRFs will be used to record any assessments performed during an unscheduled visit.

For activities that are required to be performed at unscheduled visit in case of DLT, Grade ≥3 AE leading to dose reduction or dose delay, or clinically significant ECG changes, refer to the SoA (Section 1.3).

8.10.6 Exceptional Measures During COVID-19 Pandemic

Exceptional measures during the COVID-19 pandemic, such as adjustments in study visits, may be considered if in the overall best interest of the participant. Adjustments may include:

- Use of alternative facility for assessments (e.g. local laboratory or imaging centers).
- Replacement of a study visit with alternative methods for assessments (such as phone contacts or virtual visits to assess safety).
- Postponement of a study visit or of individual assessments.
- Temporary suspension of sample collection.

A robust benefit-risk assessment should be performed by the Investigator and discussed with the Medical Monitor. This assessment will be fully documented, and any deviations to the protocol will be recorded in accordance with the Sponsor standard procedure.

9. STATISTICAL CONSIDERATIONS

9.1 STATISTICAL HYPOTHESES

Not applicable.

9.2 SAMPLE SIZE DETERMINATION

For Part I dose escalation and backfill cohorts, the exact number of participants will depend on the occurrence of DLTs and the number of dose-levels required to determine the MTD and/or RDE. A maximum of 60 DLT-evaluable participants will be needed for Part I dose escalation cohorts. This maximum sample size has been shown to provide a safe and accurate estimation of the MTD (Appendix 13, Section 3.1.2).

A maximum of 40 biopsy-evaluable participants will be required to characterize the dose/PD relationship (Section 4.1.1). It is expected that backfill cohorts will be opened at up to 4 different dose levels for this purpose. Based on preclinical data it is expected that a sample size of 10 biopsy evaluable patients per dose would likely allow detection of a reduction in the free WRN helicase compared to baseline. The number of biopsy evaluable participants in each backfill cohort may be adjusted upward or downward (without changing the total number of patients across all backfill cohorts) depending on

Use of this document is governed by the terms of use on the first page of this document.

the dose at which the backfill cohort is opened and on the number of backfill cohorts.

Participants who are not DLT-evaluable or biopsy-evaluable may be replaced (Section 9.3 and Section 4.1.6.1).

[redacted]

These expansions may be extended, via amendment, to a larger number of participants for further characterization of the RO7589831 safety and efficacy profile.

[redacted]

[redacted]

[redacted]

[redacted]

[redacted]

Use of this document is governed by the terms of use on the first page of this document.

The overall maximum number of participants will be *295* including the Part I *(Dose escalation and Backfill cohorts, 100 participants)* [redacted]

9.3 ANALYSIS SETS

For purposes of analysis, the following populations are defined in Table 13.

Table 13 Analysis Sets

Participant Analysis Set	Description
Safety	All participants assigned who received at least one dose of the study treatment will be included in the safety analysis.
Response evaluable	All participants who have received at least one dose of study drug and have a valid baseline and post baseline tumor assessment *and are MSI based on local or central testing.*
PK	All participants who have received at least one dose of study treatment and who have data from at least one postdose sample will be included in the PK analysis population. Participants will be excluded from the PK analysis population if they significantly violate the inclusion or exclusion criteria, deviate significantly from the protocol, or if data are unavailable or incomplete which may influence the PK analysis. Excluded cases will be documented with the reason for exclusion. All decisions on exclusions from the analysis will be made prior to data analysis.
DLT evaluable	DLT evaluable participants are those who have completed the DLT period without DLT, or participants reported with a DLT.<br>This population will be used in the determination of the MTD.
Biopsy evaluable	All participants from safety analysis set who have a valid baseline and on-study biopsy

Abbreviations: DLT = dose-limiting toxicity; MTD = maximum tolerated dose; PK = pharmacokinetics.

9.4 STATISTICAL ANALYSES

9.4.1 Demographics and Baseline Characteristics

Demography and baseline characteristics (including but not restricted to age, sex, biomarker status, previous therapies, and medical history) will be analyzed using descriptive statistics.

Participant disposition will be reported.

Exposure to study medication will be summarized by total duration of study medication, number of cycles started, and cumulative dose using descriptive statistics. Dose interruptions/modifications and their reasons will be presented by schedule and dose level.

Use of this document is governed by the terms of use on the first page of this document.

The above analyses will be performed on the safety dataset.

9.4.2 Efficacy Analyses

Participants will be assessed for response by the Investigators based on the RECIST v1.1. Participants with missing or no response assessments will be classified as “not evaluable”. Responses will need to be confirmed by a second assessment at least four weeks after the first assessment showing response. Only the confirmed response will be analyzed and used for futility analysis.

A participant will be considered as “responder” if the best response on treatment is equal to “complete response” (CR), or PR. The ORR will be defined as the proportion of responder out of the number of response-evaluable participants. The disease control rate (DCR) will be defined as the proportion of participants being either responder or in SD at least once at the first post-baseline tumor assessment performed a minimum of 4 weeks after study entry.

Duration of response (DOR) will be calculated for “responder” participants (i.e., best [confirmed] overall response of CR or PR) and will be defined as the time from first occurrence of a documented response until the time of documented disease progression or death from any cause, whichever occurs first.

Progression-free survival (PFS) will be defined as the time from first dose of study treatment to the first occurrence of documented disease progression or death from any cause, whichever occurs first.

For participants who do not have documented progressive disease or death until the last response assessment during the study, PFS and DOR will be censored at the day of the last on study tumor assessment. Participants without any post-baseline assessments or with all post-baseline assessments having unknown result/response but known to be alive at the clinical cut off for the analysis will be censored at the date of study treatment initiation plus one day.

OS is defined as the time from the first dose of study treatment to the time of death from any cause on study. Participants who are still alive at the time of analysis will be censored at the time of their last study assessment (for active participants) or at the last date known alive (for participants in follow-up).

Response and DOR will be based on the response-evaluable population. PFS and OS analyses will be based on the safety population.

Efficacy statistical analysis methods are shown in Table 14.

Use of this document is governed by the terms of use on the first page of this document.

Table 14 Efficacy Statistical Analysis Methods

Endpoint	Statistical Analysis Methods
ORR, DCR	Exact 95% confidence intervals (CIs) using the Clopper-Pearson method for ORR will be provided
DOR, PFS, OS	Duration of response, PFS, OS will be analyzed using Kaplan-Meyer methods

DCR = disease control rate; DOR = duration of response; ORR = objective response rate; OS = overall survival; PFS = progression-free survival.

9.4.3 Safety Analyses

Safety statistical analysis methods are shown in Table 15.

Table 15 Safety Statistical Analysis Methods

Endpoint	Statistical Analysis Methods
Adverse events	The original terms recorded on the eCRF by the Investigator for AEs will be coded by the Sponsor.<br>AEs will be summarized by mapped term and appropriate thesaurus level.
Clinical laboratory tests	All clinical laboratory data will be stored on the database in the units in which they were reported. Laboratory test values will be presented in International System of Units (SI units by individual outputs with flagging of abnormal results.<br>Summary tables of change from baseline over time will be displayed. Shifts in NCI CTCAE grades v5.0 from baseline to the worst grade observed during treatment will be presented for selected laboratory parameters. For details on standard reference ranges and data transformation and the definition of laboratory abnormalities, see Appendix 4.
Vital signs	Vital sign data will be presented with flagging of values outside the normal ranges and flagging of abnormalities.
ECG data analysis	ECG data will be presented using listings (and tables if necessary) of standards ECG parameters
Concomitant medications	The original terms recorded on the participants' eCRF by the Investigator for concomitant medications will be standardized by the Sponsor by utilizing a mapped term and appropriate drug dictionary level.<br>Concomitant medications will be presented using appropriate outputs.

Abbreviations: AE = adverse events; ECG = electrocardiogram; eCRF = electronic case report form; NCI CTCAE = National Cancer Institute Common Terminology Criteria for Adverse Events; SI = Système International d'Unités.

9.4.4 Pharmacokinetic Analyses

PK parameters for RO7589831 will be derived from the plasma concentrations determined after single and multiple dosing and will be listed and summarized using descriptive statistics (such as mean, standard deviation, coefficient of variation, median, minimum, and maximum). Actual PK sampling times are presented in the SoA (Section 1.3).The PK parameters following single and multiple dosing will include, but are not limited to, area under the curve (AUC), maximum concentration (C_{max}), time of

Use of this document is governed by the terms of use on the first page of this document.

maximum concentration observed (T_{max}) RO7589831 and, apparent oral clearance (CL/F), volume of distribution (V/F), and terminal half-life ($T_{1/2}$).

Estimation of PK parameters may be performed using standard non-compartmental methods and/or population PK modeling. If data allow, other methods may be used for data analysis, e.g., PK and PD data may be used to develop a population PK/PD model. Individual and mean plasma RO7589831 concentration versus time data will be tabulated and plotted by dose levels. Graphical displays of PK data may also be provided. The linearity of PK (AUC and C_{max}) will be investigated.

The effect of food on the PK of RO7589831 will be assessed as described in Section 5.4.1. To assess the FE on the PK of RO7589831, PK parameters (such as AUC_{tau}, C_{max}, T_{max} and $T_{1/2}$) will be analyzed following oral administration of RO7589831 while either fed (high-fat meal) or fasted. Estimation of PK parameters may be performed using standard non-compartmental methods and/or population PK modeling.

In addition, the relationship between RO7589831 exposure, safety, PD, and efficacy endpoints will be explored for establishing an exposure-response relationship. Additional PK analyses for RO7589831 (and RO7589831 metabolite(s), if available and appropriate) will be conducted as appropriate on the basis of available data.

Results of PK and/or any exposure-response relationship may be reported outside the CSR.

9.4.5 Pharmacodynamic Analyses

PD parameters will be presented by listings and descriptive summary statistics separately by group or cohorts. Graphical techniques may be employed to better understand the relationship of the PD markers with dose and time.

9.4.6 Pharmacokinetic/ Pharmacodynamic Relationships

Correlations between PK parameters, PD markers, and clinical response will be assessed through data tabulations and graphical techniques. If data allows, other methods may be used for data analysis, e.g., PK and PD data may be used to develop a population PK/PD model. The population PK analysis and PD analyses will be presented separately from the main CSR.

9.4.7 Other Analyses

9.4.7.1 Endogenous CYP3A Activity Marker Analysis

For the analysis of CYP3A activity during RO7589831 administration, the plasma concentrations of 4β–HC, TC and/or 4α–HC will be determined and the percent change from baseline of 4β–HC with and without normalization by TC and/or 4α–HC will be derived. If data allow, other exploratory methods may be used for data analysis, e.g., PK and CYP3A activity data may be used to develop a population PK/CYP3A activity model.

Use of this document is governed by the terms of use on the first page of this document.

9.4.7.2 [^{18}F] FDG-PET Imaging

For the analysis of FDG-PET data, participants with baseline and at least one postbaseline scan will be considered evaluable. Uptake of [^{18}F]-FDG-PET performed at baseline and on-treatment timepoints will be summarized descriptively. Change and percentage of change of FDG-PET parameters (such as maximum standard uptake value, mean standardized uptake value, total lesion glycolysis, etc.) from baseline will be described and evaluated as evidence of response to treatment.

9.4.7.3 Analyses of EORTC QLQ-F17 [redacted]

The change from baseline in physical functioning, role functioning, social functioning, emotional functioning, cognitive functioning, and global health status/quality of life (GHS/QoL) as assessed through the use of the EORTC QLQ-F17*, as well as disease related symptoms of CRC* [redacted]

Completion rates will be summarized by listing the number and proportion of participants who complete the PRO questionnaires (EORTC QLQ-F17, [redacted] PRO-CTCAE, EORTC-IL46) at each timepoint.

9.4.7.4 Analyses of PRO-CTCAE and EORTC-IL46

PRO-CTCAE analyses will be descriptive, with a focus on characterizing the pattern of symptomatic treatment toxicities over the course of the study. Results from these analyses will be presented separately from the other safety analyses. PRO-CTCAE data will be analyzed at the item level in line with current NCI recommendations for data handling (Basch et al. 2014).

PRO-CTCAE data will be summarized over time. The proportion of missing data at each assessment timepoint will also be summarized to facilitate interpretation of data.

The number and percentage of participants reporting each symptom and the change from baseline by category (frequency of occurrence, severity, interference) will be summarized at each assessment timepoint by treatment arm. For items that are rated on a 5-point Likert scale, the maximum post-baseline score and change from baseline will be summarized by treatment arm.

The number and proportion of participants in each category reporting they are troubled by treatment symptoms, as assessed through the single-item EORTC-IL46 will be summarized by visit.

Both the PRO-CTCAE and IL46 will use the safety evaluable population. Graphical representation of PRO-CTCAE data over time can also be provided.

Use of this document is governed by the terms of use on the first page of this document.

9.5 INTERIM ANALYSES

[redacted]

[redacted]

[redacted]

[redacted]

[redacted]

9.6 SUMMARIES OF CONDUCT OF STUDY

All reportable major protocol deviations will be listed.

Use of this document is governed by the terms of use on the first page of this document.

10. REFERENCES

Aaronson NK, Ahmedzai S, Bergman B, et al. The European Organization for Research and Treatment of Cancer QLQ-C30: a quality-of-life instrument for use in international clinical trials in oncology. J Natl Cancer Inst 1993;85:365–76.

Ali-Fehmi R, Oberley MJ, Kraus H, et al. Analysis of concordance between microsatellite instability by next generation sequencing (NGS-MSI) and mismatch repair deficiency by immunohistochemistry (IHC-MMR) in >28,000 colorectal tumors. [Meeting Abstract] J Clin Oncol.2023; DOI:10.1200/JCO.2023.41.4_suppl.30.

Amato M, Franco R, Facchini G, et al. Microsatellite Instability: From the Implementation of the Detection to a Prognostic and Predictive Role in Cancers. Int J Mol Sci. 2022;23(15):8726.

Andre T, Elez E, Cutsem Van E et al. Nivolumab (NIVO) plus ipilimumab (IPI) vs chemotherapy (chemo) as first-line (1L) treatment for microsatellite instability-high/mismatch repair-deficient (MSI-H/dMMR) metastatic colorectal cancer (mCRC): First results of the CheckMate 8HW study. [Abstract LBA768] J Clin Onc. 2024;42, Number 3_suppl

Andre T, Shiu K-K, Kim TW et al. Final overall survival for the phase III KN177 study: Pembrolizumab versus chemotherapy in microsatellite instability-high/mismatch repair deficient (MSI-H/dMMR) metastatic colorectal cancer (mCRC). J Clin. Onc. 2020.39.15_suppl [abstract]. [resource on the Internet] Avaialbe at: https://ascopubs.org/doi/10.1200/JCO.2021.39.15_suppl.3500 (Date cited: 22 May 2024)

Atallah-Yunes SA, Ready A, Newburger PE. Benign ethnic neutropenia. Blood Rev. 2019;37:100586.

Basch E, Reeve BB, Mitchell SA, et al. Development of the National Cancer Institute's patient-reported outcomes version of the common terminology criteria for adverse events (PRO-CTCAE). J Natl Cancer Inst 2014;106(9):1.11.

Basch E. The missing voice of patients in drug-safety reporting. N Engl J Med. 2010;362:865.9.

Bednar MM, Harrigan EP, Anziano RJ, et al. The QT interval. Prog Cardiovasc Dis. 2001;43(5 Suppl 1):1-45.

Benard F, Roy F-N, Wilson D et al. FDG PET early metabolic response to the PARP inhibitor olaparib (AZD2281) in BRCA-deficient or recurrent high-grade ovarian carcinoma and BRCA-deficient or triple-negative breast cancer. J Nucl Med. 2012;53 (supplement 1) 63.

Berton D, Banerjee SN, Curigliano G ety al. Antitumor activity of dostarlimab in patients with mismatch repair-deficient/microsatellite instability–high tumors: A combined analysis of two cohorts in the GARNET study. [Abstract] DOI:

Use of this document is governed by the terms of use on the first page of this document.

10.1200/JCO.2021.39.15_suppl.2564 Journal of Clinical Oncology 39, no. 15_suppl (May 20, 2021) 2564–2564.

Bonneville R, Krook MA, Kautto EA, et al. Landscape of Microsatellite Instability Across 39 Cancer Types. JCO Precis Oncol. 2017;2017:PO.17.00073.

Bratman SV, Yang SYC, Iafolla MAJ et al.Personalized circulating tumor DNA analysis as a predictive biomarker in solid tumor patients treated with pembrolizumab. Nat Cancer. 2020;1(9):873–81.

Bui QL, Mas L, Hollebecque A, et al. Treatments after Immune Checkpoint Inhibitors in Patients with dMMR/MSI Metastatic Colorectal Cancer. Cancers (Basel). 2022;14(2):406.

Chabanon RM, Rouanne M, Lord CJ, et al. Targeting the DNA damage response in immuno-oncology: developments and opportunities. Nat Rev Cancer. 2021;21(11):701-7.

Chakravarty D, Johnson A, Sklar J, et al. Genomic Testing in Patients With Metastatic or Advanced Cancer: ASCO Provisional Clinical Opinion. J Clin Oncol. 2022 Apr 10 ;40(11) :1231–8.

Chan EM, Shibue T, McFarland JM, et al. WRN helicase is a synthetic lethal target in microsatellite unstable cancers. Nature. 2019;568:551–6.

Cohen D, Krauthammer SH, Wolf I, et al. Hypermetabolic lymphadenopathy following administration of BNT162b2 mRNA Covid-19 vaccine: incidence assessed by [18F]FDG PET-CT and relevance to study interpretation. Eur J Nucl Med Mol Imaging. 2021;48(6):1854–63.

Dai M, Liu D, Liu M, et al. Patients with cancer appear more vulnerable to SARS-CoV-2: a multicenter study during the COVID-19 outbreak. Cancer Discov. 2020;10(6):783–91.

Dasari A, Morris VK, Allegra CJ, et al. ctDNA applications and integration in colorectal cancer: an NCI Colon and Rectal–Anal Task Forces whitepaper Nat Rev Clin Oncol. 2020;17(12):757–70.

Diaz LA Jr, Shiu KK, Kim TW, et al. Pembrolizumab versus chemotherapy for microsatellite instability-high or mismatch repair-deficient metastatic colorectal cancer (KEYNOTE-177): final analysis of a randomised, open-label, phase 3 study. Lancet Oncol. 2022;23:659–70.

Dueck AC, Mendoza TR, Mitchell SA, et al. Validity and reliability of the US National Cancer Institute's patient-reported outcomes version of the Common Terminology Criteria for Adverse Events (PRO-CTCAE). JAMA Oncol 2015;1:1051.9.

Ellegren H. 2004. Microsatellites: simple sequences with complex evolution. Nature Reviews Genetics 5:435–45.

Use of this document is governed by the terms of use on the first page of this document.

EMA Guideline on the investigation of drug interactions. CPMP/EWP/560/95/Rev. 1 Corr. 2. 2012. Available at: https://www.ema.europa.eu/en/documents/scientific-guideline/guideline-investigation-drug-interactions-revision-1_en.pdf. Date cited: 14-Mar-2023.

European Society For Medical Oncology [resource on the Internet]. Available at: https://www.esmo.org/guidelines/guidelines-by-topic. Date cited: 17 February 2023.

FDA Clinical Drug Interaction Studies — Cytochrome P450 Enzyme- and Transporter-Mediated Drug Interactions. Guidance for Industry. 2020. *[resource on the Internet]* Available at: https://www.fda.gov/regulatory-information/search-fda-guidance-documents/clinical-drug-interaction-studies-cytochrome-p450-enzyme-and-transporter-mediated-drug-interactions. Date cited: 14-Mar-2023.

FDA Drug Development and Drug Interactions | Table of Substrates, Inhibitors and Inducers. Content current as of: 08/24/2022. *[resource on the Internet]* Available at: https://www.fda.gov/drugs/drug-interactions-labeling/drug-development-and-drug-interactions-table-substrates-inhibitors-and-inducers. Date cited: 02-Feb-2023.

FDA In Vitro Drug Interaction Studies — Cytochrome P450 Enzyme- and Transporter-Mediated Drug Interactions. Guidance for Industry. 2020. *[resource on the Internet]* Available at: https://www.fda.gov/regulatory-information/search-fda-guidance-documents/in-vitro-drug-interaction-studies-cytochrome-p450-enzyme-and-transporter-mediated-drug-interactions. Date cited: 14-Mar-2023.

Fingert H, Varterasian M. Safety biomarkers and the clinical development of oncology therapeutics: considerations for cardiovascular safety and risk management. AAPS J. 2006;8(1):E89–94.

Fitzsimmons D, Johnson CD, George S, et al. Development of a disease specific quality of life (QoL) questionnaire module to supplement the EORTC core cancer QoL questionnaire, the QLQ-C30 in patients with pancreatic cancer. EORTC Study Group on Quality of Life. Eur J Cancer. 1999;35(6):939–41.

Gujral S, Conroy T, Fleissner C, et al. An update of the EORTC quality of life questionnaire. European Journal of Cancer. 2007;43:1564–73.

Guyot D'Asnières De Salins A, Tachon G, Cohen R, et al. Discordance between immunochemistry of mismatch repair proteins and molecular testing of microsatellite instability in colorectal cancer. ESMO Open. 2021;6(3):100120.

Hause RJ, Pritchard CC, Shendure J, et al. Classification and characterization of microsatellite instability across 18 cancer types. Nat Med. 2016;22:1342–50.

Hoong B. Yi Da, Gan Y et al. Sin cGAS-STING pathway in oncogenesis and cancer therapeutics. Oncotarget. 2020;11: 2930-55.

Use of this document is governed by the terms of use on the first page of this document.

Hochster HS, Bendell JC, Cleary JM et al. Efficacy and safety of atezolizumab (atezo) and bevacizumab (bev) in a phase Ib study of microsatellite instability (MSI)-high metastatic colorectal cancer (mCRC). 2017.DOI: 10.1200/JCO.2017.35.4_suppl.673.

Kaelin WJ. The concept of synthetic lethality in the context of anticancer therapy. Nat Rev Cancer. 2005;5(9):689–98.

Keytruda U.S. Prescribing Information. [resource on the Internet] Avaialbe at: https://www.accessdata.fda.gov/drugsatfda_docs/label/2020/125514s066lbl.pdf. Date cited: 22-May-2024.

Keytruda E.U. Summary of Product Charateristics. [resource on the Internet] Avaialble at: https://www.ema.europa.eu/en/documents/product-information/keytruda-epar-product-information_en.pdf. Date cited: 22-May-2024.

Lavacchi D, Roviello G, D'Angelo A. Tumor-Agnostic Treatment for Cancer: When How is Better than Where. Clin Drug Investig. 2020;40(6):519–27.

Le DT, Durham JN, Smith KN, et al. Mismatch repair deficiency predicts response of solid tumors to PD-1 blockade. Science. 2017;357:409–13.

Leclerc J, Vermaut C, Buisine MP. Diagnosis of Lynch Syndrome and Strategies to Distinguish Lynch-Related Tumors from Sporadic MSI/dMMR Tumors. Cancers (Basel). 2021;13(3):467.

Lenz HJ, Van Cutsem E, Luisa Limon M, et al. First-Line Nivolumab Plus Low-Dose Ipilimumab for Microsatellite Instability-High/Mismatch Repair-Deficient Metastatic Colorectal Cancer: The Phase II CheckMate 142 Study. J Clin Oncol. 2022;40(2):161–70.

Lieb S, Blaha-Ostermann S, Kamper E, et al. Werner syndrome helicase is a selective vulnerability of microsatellite instability-high tumor cells. Elife. 2019;8:e43333.

Liu H, Ye Z, Yang T et al. Predictive Value of Metabolic Parameters Derived From ^{18}F-FDG PET/CT for Microsatellite Instability in Patients With Colorectal Carcinoma. Front Immunol. 2021;12:724464.

Luchini C, Bibeau F, Ligtenberg MJL, et al. ESMO recommendations on microsatellite instability testing for immunotherapy in cancer, and its relationship with PD-1/PD-L1 expression and tumour mutational burden: a systematic review-based approach. Ann Oncol. 2019;30(8):1232–43.

Marabelle A, Le DT, Ascierto PA, et al. Efficacy of Pembrolizumab in Patients With Noncolorectal High Microsatellite Instability/Mismatch Repair-Deficient Cancer: Results From the Phase II KEYNOTE-158 Study. J Clin Oncol. 2020;38:1–10.

Marcus L, Lemery SJ, Keegan P, et al. FDA Approval Summary: Pembrolizumab for the Treatment of Microsatellite Instability-High Solid Tumors. Clin Cancer Res. 2019;25(13):3753–8.

Use of this document is governed by the terms of use on the first page of this document.

Marsavela G, McEvoy AC, Pereira MR et al.Detection of clinical progression through plasma ctDNA in metastatic melanoma patients: a comparison to radiological progression. Br J Cancer. 2022;126(3):401–8.

National Comprehensive Cancer Network. [resource on the Internet]. Available at: https://www.nccn.org/guidelines/category_1. Date cited: 17 February 2023.

Neuenschwander B, Branson M, Gsponer T. Critical Aspects of the Bayesian Approach to Phase I Cancer Trials. Statistics in Medicine. 2008;27(13):2420–39.

O'Neil NJ, Bailey ML, Hieter P. Synthetic lethality and cancer. Nat Rev Genet. 2017;18(10):613–23.

Oaknin A, Gilbert L, Tinker AV, et al. Safety and antitumor activity of dostarlimab in patients with advanced or recurrent DNA mismatch repair deficient/microsatellite instability-high (dMMR/MSI-H) or proficient/stable (MMRp/MSS) endometrial cancer: interim results from GARNET-a phase I, single-arm study. J Immunother Cancer. 2022;10(1):e003777.

Oaknin A, Tinker AV, Gilbert L, et al. Clinical activity and safety of the anti-programmed death 1 monoclonal antibody dostarlimab for patients with recurrent or advanced mismatch repair-deficient endometrial cancer: a nonrandomized phase 1 clinical trial. JAMA Oncol. 2020;6(11):1766–72.

Overman MJ, Lonardi S, Wong KYM, et al. Durable clinical benefit with nivolumab plus ipilimumab in DNA mismatch repair-deficient/microsatellite instability-high metastatic colorectal cancer. J Clin Oncol. 2018;36:773–9.

Pabla S, Andreas J, Lenzo FL, et al. Development and analytical validation of a next-generation sequencing based microsatellite instability (MSI) assay. Oncotarget. 2019;10(50):5181–93.

RO7589831 Investigator's Brochure.

Robilotti EV, Babady NE, Mead PA, et al. Determinants of COVID-19 disease severity in patients with cancer. Nat Med. 2020;26(8):1218–23.

Schettini F, Corona SP, Giudici F, et al. Clinical, Radiometabolic and Immunologic Effects of Olaparib in Locally Advanced Triple Negative Breast Cancer: The OLTRE Window of Opportunity Trial. Front Oncol. 2021;11:686776.

Schneider BJ, Naidoo J, Santomasso BD, et al. Management of Immune-Related Adverse Events in Patients Treated With Immune Checkpoint Inhibitor Therapy: ASCO Guideline Update. J Clin Oncol. 2021;39(36):4073-126.

Shah V, Ko Ko T, Zuckerman M, et al. Poor outcome and prolonged persistence of SARS-CoV-2 RNA in COVID-19 patients with haematological malignancies; King's College Hospital experience. Br J Haematol. 2020;190(5):e279–82.

Song J, Li Z, Yang L et al. Metabolic activity via ^{18}F-FDG PET/CT is predictive of microsatellite instability status in colorectal cancer. BMC Cancer. 2022;22(1):808.

Use of this document is governed by the terms of use on the first page of this document.

Snyder A, Morrissey MP, Hellmann MD. Use of Circulating Tumor DNA for Cancer Immunotherapy. Clin Cancer Res. 2019;25(23):6909–15.

Umar A, Boland CR, Terdiman JP, et al. Revised Bethesda Guidelines for hereditary nonpolyposis colorectal cancer (Lynch syndrome) and microsatellite instability. J Natl Cancer Inst. 2004;96(4):261–8.

Weber WA. Use of PET for monitoring cancer therapy and for predicting outcome. J Nucl Med. 2005;46(6):983–95.

Willis J, Lefterova MI, Artyomenko A, et al. Validation of Microsatellite Instability Detection Using a Comprehensive Plasma-Based Genotyping Panel, Clin Cancer Res. 2019 Dec 1;25(23):7035–45.

Xu G, Lu Y. COVID-19 mRNA Vaccination-Induced Lymphadenopathy Mimics Lymphoma Progression on FDG PET/CT. Clin Nucl Med. 2021;46(4):353–4.

Yang K, Sheng Y, Huang C, et al. Clinical characteristics, outcomes, and risk factors for mortality in patients with cancer and COVID-19 in Hubei, China: a multicentre, retrospective, cohort study. Lancet Oncol. 2020;21(7):904–13.

Use of this document is governed by the terms of use on the first page of this document.

11. SUPPORTING DOCUMENTATION AND OPERATIONAL CONSIDERATIONS

The following section includes standard appendices.

Appendix 1	Regulatory, Ethical, and Study Oversight Considerations
Appendix 2	Adverse Events: Definitions and Procedures for Evaluating, Follow-up and Reporting
Appendix 3	Procedures for Recording Adverse Events
Appendix 4	Clinical Laboratory Tests
Appendix 5	Contraceptive and Barrier Methods Guidance
Appendix 6	Investigational Medicinal Product and Non-Investigational Medicinal Product Designations
Appendix 7	Guidance for Fluoro-deoxy-glucose Positron Emission Tomography
Appendix 8	Response Evaluation Criteria in Solid Tumors, Version 1.1: Excerpt from Original Publication
Appendix 9	European Organization for Research and Treatment of Cancer-Function 17
Appendix 10	Patient-Reported Outcomes Common Terminology Criteria for Adverse Events
Appendix 11	European Organisation for Research and Treatment of Cancer Item List 46
[redacted]	
Appendix 13	Modified Continual Reassessment Method Escalation with Overdose Control Design Description
Appendix 14	Drugs that are Prohibited or Should be Administered with Caution and Monitoring
Appendix 15	Composition of High Fat Meal
Appendix 16	*Preexisting Autoimmune Diseases and Immune Deficiencies*
Appendix 17	*Anaphylaxis Precautions*
Appendix 18	Abbreviations

Use of this document is governed by the terms of use on the first page of this document.

Appendix 1
Regulatory, Ethical, and Study Oversight Considerations

1. REGULATORY AND ETHICAL CONSIDERATIONS

1.1. COMPLIANCE WITH LAWS AND REGULATIONS

This study will be conducted in full conformance with the International Council for Harmonisation of Technical Requirements for Pharmaceuticals for Human Use (ICH) E6 guideline for Good Clinical Practice (GCP) and the principles of the Declaration of Helsinki, or the laws and regulations of the country in which the research is conducted, whichever affords the greater protection to the individual. The study will comply with the requirements of the ICH E2A guideline (Clinical Safety Data Management: Definitions and Standards for Expedited Reporting) *and with International Standard (ISO) 20916 on In Vitro Diagnostic Medical Devices–Clinical Performance Studies Using Specimens from Human Subjects–Good Study Practice and Regulation (EU) 2017/746 on in vitro diagnostic medical devices requirements for medical device adverse event collection and reporting for events associated with the use of FoundationOne Companion Diagnostic (F1CDx) assay and/or Promega OncoMate™ MSI Dx Analysis System if applicable*. Studies conducted in the United States (U.S.) or under a U.S. Investigational New Drug (IND) application will comply with U.S. Food and Drug Administration (FDA) regulations and applicable local, state, and federal laws. Studies conducted in the European Union (E.U.)/European Economic Area (EEA) will comply with the E.U. Clinical Trial Directive (2001/20/EC) or Clinical Trials Regulation (536/2014) (EEA sites only), and all other applicable local regulations.

1.2. INSTITUTIONAL REVIEW BOARD OR ETHICS COMMITTEE

This protocol, the Master Informed Consent Forms (ICFs), any information to be given to the participant (e.g. advertisements, diaries etc.), and relevant supporting information must be submitted to the Institutional Review Board (IRB)/Ethics Committee (EC) by the Principal Investigator and reviewed and approved by the IRB/EC before the study is initiated. In addition, any participant recruitment materials must be approved by the IRB/EC.

The Principal Investigator is responsible for providing written summaries of the status of the study to the IRB/EC annually or more frequently in accordance with the requirements, policies, and procedures established by the IRB/EC. Investigators are also responsible for promptly informing the IRB/EC of any protocol amendments (Section 2.3.1 of this appendix).

The Investigator should follow the requirements for reporting all adverse events (AEs) to the Sponsor. Investigators may receive written IND safety reports or other safety-related communications from the Sponsor. Investigators are responsible for ensuring that such reports are reviewed and processed in accordance with Health Authority requirements

Use of this document is governed by the terms of use on the first page of this document.

and the policies and procedures established by their IRB/EC, and archived in the site's study file.

1.3. INFORMED CONSENT

The Sponsor's Master ICF (and ancillary sample ICFs such as a Child's Assent or Caregiver's ICF, if applicable) will be provided to each site. If applicable, it will be provided in a certified translation of the local language. Participants must be informed that their participation is voluntary. Participants will be required to sign a statement of informed consent that meets the requirements of 21 Code of Federal Regulations (CFR) Part 50, local regulations, ICH guidelines, Health Insurance Portability and Accountability Act of 1996 (HIPAA) requirements, where applicable, and the IRB/IEC or study center. The Sponsor or its designee must review and approve any proposed deviations from the Sponsor's sample ICFs or any alternate consent forms proposed by the site (collectively, the "Consent forms") before IRB/EC submission. The final IRB/EC‑approved Consent Forms must be provided to the Sponsor for Health Authority submission purposes according to local requirements. Participants must be re-consented to the most current version of the ICF(s) during their participation in the study. A copy of the ICF(s) signed by all parties must be provided to the participant.

The ICFs must be signed and dated by the participant before his or her participation in the study. The case history or clinical records for each participant shall document the informed consent process and that written informed consent was obtained prior to participation in the study.

The ICFs should be revised whenever there are changes to study procedures or when new information becomes available that may affect the willingness of the participant to take part. The final revised IRB/EC-approved ICFs must be provided to the Sponsor for Health Authority submission purposes if required as per local regulations.

If the ICFs are revised (through an amendment or an addendum) while a participant is participating in the study, the participant may be re-consented by signing the most current version of the ICFs or the addendum, in accordance with applicable laws and IRB/EC policy. For any updated or revised ICFs, the case history or clinical records for each participant shall document the informed consent process and that written informed consent was obtained using the updated/revised ICFs for continued participation in the study. The study team will provide guidance for which participants need to re-consent in the event of an update to the ICF.

A copy of each signed ICF must be provided to the participant. All signed and dated ICFs must remain in each participant's study file or in the site file and must be available for verification by study monitors at any time.

Participants who are re-screened are required to sign a new ICF.

Use of this document is governed by the terms of use on the first page of this document.

Consents to Optional Tissue Biopsy

An optional ICF will be provided to participants enrolled in Part I Dose Escalation, [redacted] of the study to request pre-treatment and on treatment tumor biopsy (Section 8.7.2.1).

An additional optional ICF will be provided to all participants to request a tumor biopsy at time of stable disease (SD), partial response (PR) or progressive disease (PD) if deemed clinically feasible by the Investigator (Section 8.7.2.2).

Consent to Participate in the Research Biosample Repository

The ICF will contain a separate section that addresses participation in the Research Biosample Repository (RBR). The Investigator or authorized designee will explain to each participant the objectives, methods, and potential hazards of participation in the RBR. Participants will be told that they are free to refuse to participate and may withdraw their samples at any time and for any reason during the storage period. A separate, specific signature will be required to document a participant's agreement to provide optional RBR samples. Participants who decline to participate will not provide a separate signature.

The Investigator should document whether or not the participant has given consent to participate by completing the RBR Sample Informed Consent electronic case report form (eCRF).

In the event of death or loss of competence of a participant who is participating in the research, the participant's samples and data will continue to be used as part of the RBR.

For sites in the United States, each ICF may also include participant authorization to allow use and disclosure of personal health information in compliance with the U.S. HIPAA. If the site utilizes a separate Authorization Form for participant authorization for use and disclosure of personal health information under the HIPAA regulations, the review, approval, and other processes outlined above apply, except that IRB review and approval may not be required per study site policies.

Consents to Continue treatment beyond progression

Participants must provide written consent to acknowledge deferring other treatment options in favor of continuing study treatment at the time of initial progression.

Approval by the Institutional Review Board or Ethics Committee

Collection, storage, and analysis of RBR samples is contingent upon the review and approval of the exploratory research and the RBR portion of the ICF by each site's IRB/EC and, if applicable, an appropriate regulatory body. If a site has not been granted approval for RBR sampling, this section of the protocol will not be applicable at that site.

Use of this document is governed by the terms of use on the first page of this document.

Withdrawal from the Research Biosample Repository

Participants who give consent to provide samples for the RBR have the right to withdraw their samples at any time for any reason. If a participant wishes to withdraw consent to the testing of his or her samples, the Investigator must inform the Medical Monitor and Site Monitor in writing of the participant's wishes using the RBR Withdrawal Form and, if the study is ongoing, must enter the date of withdrawal on the RBR Withdrawal of Informed Consent eCRF. The participant will be provided with instructions on how to withdraw consent after the study is closed. A participant's withdrawal from Study BP44474 does not, by itself, constitute withdrawal of samples from the RBR. Likewise, a participant's withdrawal from the RBR does not constitute withdrawal from Study BP44474. Data already generated before time of withdrawal of consent to RBR will still be used.

1.4. CONFIDENTIALITY

Information technology systems used to collect, process, and store study-related data are secured by technical and organizational security measures designed to protect such data against accidental or unlawful loss, alteration, or unauthorized disclosure or access. In the event of a data security breach, appropriate mitigation measures will be implemented.

Participants will be assigned a unique identifier by the Sponsor. Any participant records or datasets that are transferred to the Sponsor will contain the identifier only; participant names or any information which would make the participant identifiable will not be transferred.

The participant must be informed that his/her personal study-related data will be used by the Sponsor in accordance with local data protection law. The level of disclosure must also be explained to the participant.

Medical information may be given to a participant's personal physician or other appropriate medical personnel responsible for the participant's welfare, for treatment purposes.

The participant must be informed that his/her medical records may be examined by Clinical Quality Assurance auditors or other authorized personnel appointed by the Sponsor, by appropriate IRB/EC members, and by inspectors from regulatory authorities.

Study data which may include data on germline mutations, may be submitted to government or other health research databases or shared with researchers, government agencies, companies, or other groups that are not participating in this study. These data may be combined with or linked to other data and used for research purposes, to advance science and public health, or for analysis, development, and commercialization

Use of this document is governed by the terms of use on the first page of this document.

of products to treat and diagnose disease. In addition, redacted CSRs and other summary reports will be provided upon request.

Confidentiality for Research Biosample Repository

Data generated from RBR samples must be available for inspection upon request by representatives of national and local Health Authorities, and Roche monitors, representatives, and collaborators, as appropriate.

Participant medical information associated with RBR samples is confidential and may only be disclosed to third parties as permitted by the ICF (or separate authorization for use and disclosure of personal health information) signed by the participant, unless permitted or required by law.

Data derived from RBR sample analysis on individual participants will generally not be provided to study Investigators unless a request for research use is granted. The aggregate results of any conducted research will be available in accordance with the effective Roche policy on study data publication.

Genetic research data and associated clinical data may be shared with researchers who are not participating in the study or submitted to government or other health research databases for broad sharing with other researchers. Participants will not be identified by name or any other personally identifying information. Given the complexity and exploratory nature of these analyses, genetic data and analyses will not be shared with Investigators or participants unless required by law.

Any inventions and resulting patents, improvements, and/or know-how originating from the use of the RBR sample data will become and remain the exclusive and unburdened property of the Sponsor, except where agreed otherwise.

Monitoring and Oversight of the Research Biosample Repository

Samples collected for the RBR will be tracked in a manner consistent with GCP by a quality-controlled, auditable, and appropriately validated laboratory information management system, to ensure compliance with data confidentiality as well as compliance to authorized use of samples as specified in this protocol and in the ICF. The Sponsor's monitors and auditors will have direct access to appropriate parts of records relating to participant participation in the RBR for the purposes of verifying the data provided to the Sponsor. The site will permit monitoring, audits, IRB/EC review, and Health Authority inspections by providing direct access to source data and documents related to the samples.

1.5. FINANCIAL DISCLOSURE

Investigators will provide the Sponsor with sufficient, accurate financial information in accordance with local regulations to allow the Sponsor to submit complete and accurate financial certification or disclosure statements to the appropriate Health Authorities.

Use of this document is governed by the terms of use on the first page of this document.

Investigators are responsible for providing information on financial interests during the course of the study and for one year after completion of the study (see definition of end of study in Section 4.5).

2. DATA HANDLING AND RECORD

2.1. DATA COLLECTION AND MANAGEMENT RESPONSIBILITIES

2.1.1. Data Quality Assurance

All participant data relating to the study will be recorded on printed or eCRF unless transmitted to the Sponsor or designee electronically (e.g., laboratory data). The Investigator is responsible for verifying that data entries are accurate and correct by physically or electronically signing the case report form (CRF).

The Investigator must maintain accurate documentation (source data) that supports the information entered in the CRF.

The Investigator must permit study-related monitoring, audits, IRB/EC review, and regulatory agency inspections and provide direct access to source data documents.

The Sponsor or designee is responsible for the data management of this study including quality checking of the data.

Study monitors will perform ongoing source data verification to confirm that data entered into the CRF by authorized site personnel are accurate, complete, and verifiable from source documents; that the safety and rights of participants are being protected; and that the study is being conducted in accordance with the currently approved protocol and any other study agreements, ICH GCP, and all applicable regulatory requirements.

2.1.2. Clinical Outcome Assessment Data

2.1.2.1 Electronic Clinical Outcome Assessment Data

An electronic clinical outcome assessment (eCOA) device will be used to capture clinical outcome assessment (COA) data. The data will be transmitted via a device automatically after entry if connected to the internet or, when site performs a transmission to a centralized database at the eCOA vendor. The data may be reviewed by site staff via a secure access to a web portal.

The eCOA data will be collected using an electronic device provided by an eCOA vendor. The device is designed for entry of data in a way that is attributable, secure, and accurate, in compliance with U.S. FDA regulations for electronic records (21 CFR Part 11). System backups for data stored by the Sponsor and records retention for the study data will be consistent with the Sponsor's standard procedures.

Use of this document is governed by the terms of use on the first page of this document.

2.1.3. Source Data Records

Source documents (paper or electronic) are those in which participant data are recorded and documented for the first time. They include, but are not limited to, hospital records, clinical and office charts, laboratory notes, memoranda, COAs (paper or eCOA), evaluation checklists, pharmacy dispensing records, recorded data from automated instruments, copies of transcriptions that are certified after verification as being accurate and complete, microfiche, photographic negatives, microfilm or magnetic media, x-rays, participant files, and records kept at pharmacies, laboratories, and medico‑technical departments involved in a clinical study.

Before study initiation, data to be entered directly into the eCRFs (i.e., no prior written or electronic record of the data) and considered source data must be defined in the Trial Monitoring Plan.

Source documents that are required to verify the validity and completeness of data entered into the eCRFs must not be obliterated or destroyed and must be retained per the policy for retention of records described below.

To facilitate source data verification, the Investigators and institutions must provide the Sponsor direct access to applicable source documents and reports for trial‑related monitoring, Sponsor audits, and IRB/EC review. The investigational site must also allow inspection by applicable Health Authorities.

2.1.4. Use of Computerized Systems

When clinical observations are entered directly into an investigational site's computerized medical record system (i.e., in lieu of original hardcopy records), the electronic record can serve as the source document if the system has been validated in accordance with Health Authority requirements pertaining to computerized systems used in clinical research. An acceptable computerized data collection system allows preservation of the original entry of data. If original data are modified, the system should maintain a viewable audit trail that shows the original data as well as the reason for the change, name of the person making the change, and date of the change.

2.1.5. Safety Biomarker Data

Adverse event (AE) reports will not be derived from safety biomarker data by the Sponsor, and safety biomarker data will not be included in the formal safety analyses for this study. In addition, safety biomarker data will not inform decisions on participant management.

2.2. RETENTION OF RECORDS

Records and documents, including signed ICFs, pertaining to the conduct of this study must be retained by the Investigator for at least 15 years after study completion or discontinuation of the study, or for the length of time required by relevant national or

Use of this document is governed by the terms of use on the first page of this document.

local Health Authorities, whichever is longer. After that period, the documents may be destroyed, subject to local regulations. No records may be destroyed during the retention period without the written approval of the Sponsor. No records may be transferred to another location or party without written notification to the Sponsor.

The Sponsor will retain study data for 25 years after the final study results have been reported or for the length of time required by relevant national or local Health Authorities.

2.3. STUDY RECORDS

The Investigator must maintain adequate and accurate records to enable the conduct of the study to be fully reconstructed, including but not limited to the protocol, protocol amendments, ICFs, and documentation of IRB/EC and governmental approval.

Roche shall also submit an Annual Safety Report once a year to the EC and competent authorities according to local regulatory requirements and timelines of each country participating in the study.

2.3.1. Protocol Amendments

Any protocol amendments will be prepared by the Sponsor. Protocol amendments will be submitted to the IRB/EC and to regulatory authorities in accordance with local regulatory requirements.

Approval must be obtained from the IRB/EC and regulatory authorities (as locally required) before implementation of any changes, except for changes necessary to eliminate an immediate hazard to participants or any non-substantial changes, as defined by regulatory requirements.

2.3.2. Publication Policy

The results of this study may be published or presented at scientific meetings. If this is foreseen, the Investigator agrees to submit all manuscripts or abstracts to the Sponsor for approval prior to submission. This allows the Sponsor to protect proprietary information and to provide comments on the basis of information from other studies that may not yet be available to the Investigator.

The Sponsor will comply with the requirements for publication of study results. In accordance with standard editorial and ethical practice, the Sponsor will generally support publication of multicenter studies only in their entirety and not as individual center data. In this case, a coordinating Investigator will be designated by mutual agreement.

Any formal publication of the study in which contribution of Sponsor personnel exceeded that of conventional monitoring will be considered as a joint publication by the Investigator and the appropriate Sponsor personnel.

Use of this document is governed by the terms of use on the first page of this document.

Authorship will be determined by mutual agreement and in line with International Committee of Medical Journal Editors authorship requirements.

Any inventions and resulting patents, improvements, and/or know-how originating from the use of data from this study will become and remain the exclusive and unburdened property of the Sponsor, except where agreed otherwise.

2.3.3. Dissemination of Clinical Study Data

Study data which may include imaging data and data on genomic variants, may be submitted to government or other health research databases or shared with researchers, government agencies, companies, or other groups that are not participating in this study. These data may be combined with or linked to other data and used for research purposes, to advance science and public health, or for analysis, development, and commercialization of products to treat and diagnose disease. In addition, redacted CSRs and/or other summaries of clinical study results may be available in health authority databases for public access, as required by local regulation, and will be provided upon request. For more information, refer to the Roche Global Policy on Sharing of Clinical Study Information at the following website:

https://www.roche.com/innovation/process/clinical-trials/data-sharing/

Given the complexity and exploratory nature of exploratory biomarker analyses, data derived from these analyses will generally not be provided to Study Investigators or participants unless required by law. The aggregate results of any conducted research will be available in accordance with the effective Roche policy on study data publication.

2.3.4. Management of Study Quality

The Sponsor will implement a system to manage the quality of the study, focusing on processes and data that are essential to ensuring participant safety and data integrity. Prior to first participant entry into the study, the Sponsor will identify and evaluate potential risks associated with critical study processes and data, as well as implement controls for the communication, review, and reporting of these risks. Details regarding the applied approach for the study will be provided in the integrated Risk Based Quality Management Plan.

2.3.5. Site Inspections

Site visits will be conducted by the Sponsor or an authorized representative for inspection of study data, participants' medical records, and eCRFs. The Investigator will permit national and local Health Authorities, Sponsor monitors, representatives, and collaborators, and the IRBs/ECs to inspect facilities and records relevant to this study.

Use of this document is governed by the terms of use on the first page of this document.

3. ADMINISTRATIVE STRUCTURE

The Investigator may delegate certain trial-related duties to appropriately qualified study site personnel in compliance with ICH GCP and a delegation of authority log will appropriately documented in the site study files.

See Section 4.1.7 of the protocol for additional details (Administrative Structure).

4. STUDY AND SITE CLOSURE

The Sponsor (or designee) has the right to close the study site or terminate this study at any time. Reasons for terminating the study may include, but are not limited to, the following:

- The incidence or severity of AEs in this or other studies indicates a potential health hazard to participants.
- Participant enrollment is unsatisfactory.

If general study stopping criteria are met, the study will be paused and the Sponsor will conduct a full review of all available safety data. The study will resume if the Sponsor and Investigator agree and after informing and receiving agreement from relevant Health Authorities and Ethics Committees.

If the study is placed on hold, or if the Sponsor decides to discontinue the study or the development program, the Sponsor shall promptly inform the Investigators, the IRBs/ECs, the Health Authorities, and any contract research organizations used for the study of the reason for termination or suspension, as specified by the applicable regulatory requirements. The Investigators shall promptly inform the participants and should ensure appropriate participant therapy and/or follow-up.

Study sites will be closed upon study completion. A study site is considered closed when all required documents and study supplies have been collected and a study-site closure visit has been performed.

The Investigator may initiate study-site closure at any time, provided there is reasonable cause and sufficient notice is given in advance of the intended termination.

Reasons for the early closure of a study site by the Sponsor or Investigator may include but are not limited to:

- Failure of the Investigator to comply with the protocol, the requirements of the IRB/EC or local Health Authorities, the Sponsor's procedures, or GCP guidelines.
- Inadequate recruitment of participants by the Investigator.
- Discontinuation of further study treatment development.

Use of this document is governed by the terms of use on the first page of this document.

Appendix 2
Adverse Events: Definitions and Procedures for Evaluating, Follow-up and Reporting

1. DEFINITION OF ADVERSE EVENTS

According to the E2A International Council for Harmonisation of Technical Requirements for Pharmaceuticals for Human Use (ICH) guideline for Good Clinical Practice (GCP), an **adverse event** (AE) is any untoward medical occurrence in a participant or clinical investigation participant administered a pharmaceutical product and that does not necessarily have to have a causal relationship with this treatment.

An AE can therefore be:

- Any unfavorable and unintended sign (including an abnormal laboratory finding), symptom, or disease temporally associated with the use of a medicinal product, whether or not considered related to the medicinal product.

Events Meeting the AE Definition:

- Deterioration in a laboratory value (hematology, clinical chemistry, or urinalysis) or other clinical test (e.g., ECG, x-ray) that is associated with symptoms or leads to a change in study treatment or concomitant treatment or discontinuation from study treatment (see Appendix 3 Section 4).
- Exacerbation of a chronic or intermittent preexisting condition, including either an increase in frequency and/or intensity of the condition.
- New conditions detected or diagnosed after study treatment administration even though it may have been present before the start of the study.
- AEs that are related to a protocol-mandated intervention, including those that occur prior to assignment of study treatment (e.g., screening invasive procedures such as biopsies).
- "Lack of efficacy" or "failure of expected pharmacological action" per se will not be reported as an AE or serious AE (SAE). Such instances will be captured in the efficacy assessments. However, the signs, symptoms, and/or clinical sequelae resulting from lack of efficacy will be reported as an AE or SAE if they fulfill the definition of an AE or SAE.

Events NOT Meeting the AE Definition:

- Any clinically significant abnormal laboratory findings or other abnormal safety assessments that are associated with the underlying disease, unless judged by the Investigator to be more severe than expected for the participant's condition.
- The disease/disorder being studied or expected progression, signs, or symptoms of the disease/disorder being studied, unless more severe than expected for the participant's condition.

Use of this document is governed by the terms of use on the first page of this document.

- Medical or surgical procedure (e.g., endoscopy, appendectomy): The condition that leads to the procedure is an AE.
- Situations where an untoward medical occurrence did not occur (social and/or convenience admission to a hospital).
- Anticipated day-to-day fluctuations of preexisting disease(s) or condition(s) present or detected at the start of the study that do not worsen.

2. DEFINITION OF SERIOUS ADVERSE EVENTS

If an event is not an AE per definition above, then it cannot be an SAE even if serious conditions are met (e.g., hospitalization for signs/symptoms of the disease under study, death due to progression of disease).

An SAE is defined as any untoward medical occurrence that at any dose:

- **Results in death.**
- **Is life-threatening.**
- The term “life-threatening” in the definition of “serious” refers to an event during which the participant was at risk of death at the time of the event; it does not refer to an event which hypothetically might have caused death if it was more severe.
- **Requires inpatient hospitalization or prolongation of existing hospitalization** (see Appendix 3).

  In general, hospitalization signifies that the participant has been detained (usually involving at least an overnight stay) at the hospital or emergency ward for observation and/or treatment that would not have been appropriate in the physician’s office or outpatient setting. Complications that occur during hospitalization are AEs. If a complication prolongs hospitalization or fulfills any other serious criteria, the event is serious. When in doubt as to whether “hospitalization” occurred or was necessary, the AE should be considered serious.

- **Results in persistent or significant disability/incapacity**
  - Disability means substantial disruption of the participant’s ability to conduct normal life functions.
  - This definition is not intended to include experiences of relatively minor medical significance such as uncomplicated headache, nausea, vomiting, diarrhea, influenza, and accidental trauma (e.g., sprained ankle) which may interfere with or prevent everyday life functions but do not constitute a substantial disruption.
- **Is a congenital anomaly/birth defect.**
- **Other significant events:**
  - Medical or scientific judgment should be exercised in deciding whether SAE reporting is appropriate in other situations such as important medical events that may not be immediately life-threatening or result in death or hospitalization

Use of this document is governed by the terms of use on the first page of this document.

but may jeopardize the participant or may require medical or surgical intervention to prevent one of the other outcomes listed in the above definition. These events should usually be considered serious.

– Examples of such events include invasive or malignant cancers, intensive treatment in an emergency room or at home for allergic bronchospasm, blood dyscrasias or convulsions that do not result in hospitalization, or development of drug dependency or drug abuse.

<u>If Applicable based on local requirements: Definition of Device Adverse Event</u>

This definition is applicable to Foundation Medicine FoundationOne Companion Diagnostic (F1CDx) assay and/or Promega OncoMate™ MSI Dx Analysis System if used as investigational devices.

Any untoward medical occurrence, inappropriate patient management decision, unintended disease or injury, or untoward clinical signs in subjects, users, or other persons, with any connection to study related activities, whether or not related to the IVD medical device under investigation. Adverse events can be caused by, for instance, insufficient or inadequate instructions for use, deployment, installation, operation, or any malfunction of the IVD medical device under investigation. This definition includes the malfunction or deterioration of a device which has not yet caused death or serious injury, but which could lead to death or serious injury. This definition is not intended to be used in determining whether an event is reportable to a regulatory authority. For users or other persons, this definition is restricted to events related (defined as possible, probable, or causal relationship) with the investigational IVD medical device or device-related procedures. False negative or false positive results are not considered an adverse event unless in an interventional study, inappropriate patient management decisions are made based on those false results.

<u>If Applicable based on local requirements: Definition of Device Deficiency</u>

This definition is applicable to Foundation Medicine FoundationOne Companion Diagnostic (F1CDx) assay and/or Promega OncoMate™ MSI Dx Analysis System if used as investigational devices.

A device deficiency is any inadequacy of a medical device with respect to its identity, quality, durability, reliability, usability, safety, or performance. Device deficiencies include malfunctions, use errors, and inadequacy in the information supplied by the manufacturer including labeling. This definition includes device deficiencies related to the investigational medical device or the comparator. The investigator is not responsible for reporting device deficiency; however, the investigator is responsible for reporting adverse events associated with a device deficiency (Appendix 3).

Use of this document is governed by the terms of use on the first page of this document.

3. RECORDING OF ADVERSE EVENT AND/OR SERIOUS ADVERSE EVENT

When an AE/SAE occurs, it is the responsibility of the Investigator to review all documentation (e.g., hospital progress notes, laboratory reports, and diagnostics reports) related to the event.

The Investigator will then record all relevant AE/SAE information in the electronic case report form (eCRF).

It is **not** acceptable for the Investigator to send photocopies of the participant's medical records to Medical Monitor in lieu of completion of the eCRF.

There may be instances when copies of medical records for certain cases are requested by Sponsor. In this case, all participant identifiers, with the exception of the participant number, will be redacted on the copies of the medical records before submission to Sponsor.

The Investigator will attempt to establish a diagnosis of the event on the basis of signs, symptoms, and/or other clinical information. Whenever possible, the diagnosis (not the individual signs/symptoms) will be documented as the AE/SAE.

The Investigator will be informed of any device deficiencies (defined in Appendix 2) detected at the testing site that might have an impact on clinical study participants (e.g., a test result leading to inappropriate participant management decision). This will enable the investigator to assess and report any adverse events associated with the device deficiency and therefore related to the IVD. If the investigator becomes aware of any user or other person that experiences an IVD related adverse event, these must be reported as described in Appendix 2, Table 1.

Table 1 Timeline and Mechanism for Collecting and Reporting of Medical Device Adverse Events

Adverse Event (Initial and Follow-Up Information)	***Collection Period***		***Reporting Timeline (Investigator's Awareness to Sponsor)***	***Collection Mechanism***	
	Start	***Stop***		***Primary***	***Back-Up*** [a]
AE related to IVD protocol-mandated procedure in a user or other person [b]	*First participant screened*	*End of study*	*5 days*	*Medical Device Adverse Event and Device Deficiency Form*	*NA*

Use of this document is governed by the terms of use on the first page of this document.

Adverse Event (Initial and Follow-Up Information)	*Collection Period*		*Reporting Timeline (Investigator's Awareness to Sponsor)*	*Collection Mechanism*	
	Start	*Stop*		*Primary*	*Back-Up* [a]
SAE related to IVD protocol-mandated procedure in a user or other person [b]	*First participant screened*	*End of study*	*Immediately (no later than 24 hours)*	*Medical Device Adverse Event and Device Deficiency Form*	*NA*
AE related to any protocol-mandated IVD procedure in a participant	*Signed ICF*	*Initiation of study treatment*	*5 days*	*Screening Adverse Event eCRF*	*Medical Device Adverse Event and Device Deficiency Form*
SAE related to any protocol-mandated procedure (including an IVD procedure) in a participant	*Signed ICF*	*Initiation of study treatment*	*Immediately (no later than 24 hours)*	*Screening Adverse Event eCRF*	*Clinical Trial Adverse Event/ Special Situations Form*
AE related to any protocol-mandated IVD procedure in a participant	*Initiation of study treatment*	*30 days after final dose of study treatment*	*5 days*	*Adverse Event eCRF*	*Medical Device Adverse Event and Device Deficiency Form*
SAE related to a protocol-mandated procedure in a participant	*Signed ICF*	*Initiation of study treatment*	*Immediately (no later than 24 hours)*	*Clinical Trial Adverse Event/ Special Situations Form*	*NA*

AE =adverse event; eCRF =electronic Case Report Form; ICF =Informed Consent Form; IVD =in vitro diagnostic; NA =not applicable; SAE =serious adverse event.

[a] *Back-up mechanism should only be used if the EDC system is not available. Once the EDC system is available, all information will need to be entered as soon as possible and submitted via the EDC system.*

[b] *Any person other than the clinical trial participant.*

3.1. ASSESSMENT OF SEVERITY

The AE severity grading scale for the National Cancer Institute (NCI) Common Terminology Criteria for Adverse Events (CTCAE) v5.0 will be used for assessing AE severity. Table 2 will be used for assessing severity for AEs that are not specifically listed in the NCI CTCAE.

Use of this document is governed by the terms of use on the first page of this document.

Table 2 Adverse Event Severity Grading Scale

Grade	Severity
1	Mild; asymptomatic or mild symptoms; clinical or diagnostic observations only; or intervention not indicated
2	Moderate; minimal, local, or non-invasive intervention indicated; or limiting age-appropriate instrumental activities of daily living[a]
3	Severe or medically significant, but not immediately life-threatening; hospitalization or prolongation of hospitalization indicated; disabling; or limiting self-care activities of daily living[b,c]
4	Life-threatening consequences or urgent intervention indicated[d]
5	Death related to adverse event[d]

Note: Based on the National Cancer Institute Common Terminology Criteria for Adverse Events v5.0, which can be found at: https://ctep.cancer.gov/protocolDevelopment/electronic_applications/docs/CTCAE_v5_Quick_Reference_8.5x11.pdf

[a] Instrumental activities of daily living refer to preparing meals, shopping for groceries or clothes, using the telephone, managing money, etc.

[b] Examples of self-care activities of daily living include bathing, dressing and undressing, feeding one self, using the toilet, and taking medications, as performed by participants who are not bedridden.

[c] If an event is assessed as a "significant medical event," it must be reported as a serious adverse event (see Section 6 of this appendix for reporting instructions), per the definition of serious adverse event in Section 2.

[d] Grade 4 and 5 events must be reported as serious adverse events (see Section 6 for reporting instructions), per the definition of serious adverse event in Section 2. Grade 4 laboratory abnormalities would only be reported as SAEs if these meets one or more of the conditions outlined in Section 2 (Definition of Serious Adverse Events) of Appendix 2.

3.2. ASSESSMENT OF CAUSALITY

Investigators should use their knowledge of the participant, the circumstances surrounding the event, and an evaluation of any potential alternative causes to determine whether or not an AE is considered to be related to the study treatment, indicating "yes" or "no" accordingly. The following guidance should be taken into consideration:

- Temporal relationship of event onset to the initiation of study treatment.
- Course of the event, considering especially the effects of dose reduction, discontinuation of study treatment, or reintroduction of study treatment.
- Known association of the event with the study treatment or with similar treatments.
- Known association of the event with the disease under study.
- Presence of risk factors in the participant or use of concomitant medications known to increase the occurrence of the event.
- Presence of non-treatment-related factors that are known to be associated with the occurrence of the event.

Use of this document is governed by the terms of use on the first page of this document.

For participant receiving combination therapy, causality will be assessed individually for each protocol-mandated therapy.

Causality Assessment for IVD Adverse Events

The circumstances surrounding the event, an evaluation of any potential alternative causes, and any knowledge about the clinical study participant, user, or other person should be used to determine whether an adverse event is considered to be related to the use of Foundation Medicine FoundationOne Companion Diagnostic (F1CDx) assay and/or Promega OncoMate™ MSI Dx Analysis System if applicable (including any device-related procedures). The guidance provided below should be used to assess the causality of these adverse events.

Relationship	***Criteria for Classification***
Not related	*The relationship of the event to the device or device-related procedures can be excluded when:*<br>• *The event has no temporal relationship with the use of the investigational device, or the procedures related to application of the investigational device; the serious adverse event does not follow a known response pattern to the medical device (if the response pattern is previously known) and is biologically implausible.*<br>• *The adverse event can be attributed to another cause (e.g., an underlying or concurrent illness or clinical condition; an effect of another device, drug, or treatment; or other risk factors).*<br>• *The event does not depend on a false result given by the investigational device used for diagnosis, when applicable.*<br><br>*In order to establish the non-relatedness, not all the criteria listed above might be met at the same time, depending on the type of device or procedures and the serious adverse event.*
Possible	*The relationship with the use of the investigational device or comparator, or the relationship with procedures, is weak but cannot be ruled out completely. Alternative causes are also possible (e.g., an underlying or concurrent illness or clinical condition and/or an effect of another device, drug, or treatment). Cases where relatedness cannot be assessed, or no information has been obtained, should also be classified as possible.*
Probable	*The relationship with the use of the investigational device or comparator, or the relationship with procedures, seems relevant and/or the event cannot be reasonably explained by another cause.*

Use of this document is governed by the terms of use on the first page of this document.

Relationship	*Criteria for Classification*
Causal	*The adverse event or serious adverse event is associated with the investigational device, comparator, or with procedures beyond reasonable doubt when:*<br>• *The event is a known side effect of the specimen collection procedures for the investigational IVD.*<br>• *The event has a temporal relationship with investigational device use or application or procedures.*<br>• *The adverse event follows a known response pattern to the medical device (if the response pattern is previously known).*<br>• *Other possible causes (e.g., an underlying or concurrent illness or clinical condition and/or an effect of another device, drug, or treatment) have been adequately ruled out.*<br>• *Harm to the subject is due to error in use.*<br>• *The event depends on a false result given by the investigational device used for diagnosis, when applicable.*<br><br>*In order to establish the relatedness, not all the criteria listed above might be met at the same time, depending on the type of device or procedures and the serious adverse event.*

IVD =in vitro diagnostic.

Note: Events associated with a procedure that would have been performed in a clinical study participant regardless of whether the device was used or not should be classified as not related.

4. FOLLOW-UP OF ADVERSE EVENTS AND SERIOUS ADVERSE EVENTS

The Investigator is obligated to perform or arrange for the conduct of supplemental measurements and/or evaluations as medically indicated or as requested by Sponsor to elucidate the nature and/or causality of the AE or SAE as fully as possible. This may include additional laboratory tests or investigations, histopathological examinations, or consultation with other health care professionals.

If a participant dies during participation in the study or during a recognized follow-up period, when possible, the Investigator will provide Sponsor with a copy of any post-mortem findings, including histopathology.

New or updated information will be recorded in the originally completed eCRF.

The Investigator will submit any updated SAE data to the Sponsor within 24 hours of receipt of the information.

Use of this document is governed by the terms of use on the first page of this document.

5. IMMEDIATE REPORTING REQUIREMENTS FROM INVESTIGATOR TO SPONSOR

Certain events require immediate reporting to allow the Sponsor to take appropriate measures to address potential new risks in a clinical study. The Investigator must report such events to the Sponsor immediately; under no circumstances should reporting take place more than 24 hours after the Investigator learns of the event. The following is a list of events that the Investigator must report to the Sponsor within 24 hours after learning of the event, regardless of relationship to study treatment:

- SAEs
- AESIs (Section 8.3.6)
- Pregnancies (Section 8.3.5)
- Dose-limiting toxicities (DLTs) during the DLTs assessment window (see Section 4.1.6.3)

The Investigator must report new significant follow-up information for these events to the Sponsor immediately (i.e., no more than 24 hours after becoming aware of the information). New significant information includes the following:

- New signs or symptoms or a change in the diagnosis.
- Significant new diagnostic test results.
- Change in causality on the basis of new information.
- Change in the event’s outcome, including recovery.
- Additional narrative information on the clinical course of the event.

Investigators must also comply with local requirements for reporting SAEs to the local Health Authority and Institutional Review Board (IRB)/Ethics Committee (EC).

5.1 REPORTING REQUIREMENTS OF SERIOUS ADVERSE EVENTS, ADVERSE EVENTS OF SPECIAL INTEREST AND DOSE-LIMITING TOXICITIES

Events that Occur Prior to Study Treatment Initiation

After informed consent has been obtained but prior to initiation of study treatment, only SAEs caused by a protocol-mandated intervention should be reported. The Clinical Trial Adverse Event/Special Situations Form provided to Investigators should be completed and submitted to the Serious Adverse Event Responsible immediately (i.e., no more than 24 hours after learning of the event).

Events that Occur After Study Treatment Initiation

Use of this document is governed by the terms of use on the first page of this document.

For reports of SAEs and AESI (Section 8.3.6) that occur after initiation of study treatment (Section 8.3.1), Investigators should record all case details that can be gathered immediately (i.e., within 24 hours after learning of the event) on the appropriate Adverse Event of Special Interest/Serious Adverse Event eCRF form and submit the report via the electronic data capture (EDC) system. A report will be generated and sent to the Sponsor's Safety Risk Management department.

In the event that the EDC system is unavailable, the Clinical Trial Adverse Event/Special Situations Form provided to Investigators should be completed and submitted to the Serious Adverse Event Responsible immediately (i.e., no more than 24 hours after learning of the event).

Once the EDC system is available, all information will need to be entered and submitted via the EDC system.

Reporting of Post-Study Adverse Events and Serious Adverse Events

After the end of the AE reporting period (see Section 8.3.1) all deaths, regardless of cause, should be reported through use of the Long-Term Survival Follow-Up eCRF.

In addition, if the Investigator becomes aware of an SAE that is believed to be related to prior study treatment, the event should be reported directly to the Sponsor or its designee, either by faxing or by scanning and emailing the Clinical Trial Adverse Event/Special Situations Form using the fax number or email address provided to Investigators.

After the Adverse Event Collection Period

The Sponsor should also be notified if the investigator becomes aware of a serious adverse event that has a possible, probable, or causal relationship with an investigational IVD medical device, a device-related procedure, or a device deficiency related to an IVD. These events must be reported directly to the Sponsor or its designee, using the Investigational Medical Device Adverse Event and Device Deficiency Form.

5.2 REPORTING REQUIREMENTS FOR CASES OF OVERDOSE, MEDICATION ERROR, DRUG ABUSE, OR DRUG MISUSE

Overdose (accidental or intentional), medication error, drug abuse, and drug misuse (hereafter collectively referred to as "special situations") are defined as follows:

- Accidental overdose: accidental administration of a drug in a quantity that is higher than the assigned dose
- Intentional overdose: intentional administration of a drug in a quantity that is higher than the assigned dose

Use of this document is governed by the terms of use on the first page of this document.

- Medication error: accidental deviation in the administration of a drug (e.g., wrong drug, expired drug, accidental overdose, underdose, wrong dosing schedule, incorrect route of administration)
- Drug abuse: intentional excessive use of a drug that may lead to addiction or dependence, physical harm, and/or psychological harm
- Drug misuse: intentional deviation in the administration of a drug that does not qualify as drug abuse

In cases where drug is to be self-administered by the participant, drug misuse could involve the drug being administered to someone other than the participant.

After initiation of study drug, special situations associated with RO7589831 and any associated adverse events will be reported until 30 days after the final dose of study drug.

Special situations, regardless of whether they result in an adverse event, should be reported on the Special Situations eCRF. If there are any associated adverse events, each event should be recorded separately on the Adverse Event eCRF.

Special situations and any associated adverse events should be reported within 30 days after the investigator becomes aware of the situation. However, if an associated adverse event fulfills seriousness criteria or qualifies as an adverse event of special interest, both the event and the special situation should be reported to the Sponsor immediately (i.e., no more than 24 hours after the Investigator becomes aware of the event), as described in Appendix 2 Section 5.1.

6. EXPEDITED REPORTING TO HEALTH AUTHORITIES, INVESTIGATORS, INSTITUTIONAL REVIEW BOARDS, AND ETHICS COMMITTEES

The Sponsor will promptly evaluate all SAEs and AESI against cumulative product experience to identify and expeditiously communicate possible new safety findings to Investigators, IRBs/ECs, and applicable Health Authorities on the basis of applicable legislation.

To determine reporting requirements for single AE cases, the Sponsor will assess the expectedness of these events through use of the RSI in the document listed below:

Drug	Document
RO7589831<br>*Pembrolizumab*	RO7589831 Investigator's Brochure<br>*EU-SmPC*

The Sponsor will compare the severity of each event and the cumulative event frequency reported for the study with the severity and frequency reported in the applicable reference document.

Use of this document is governed by the terms of use on the first page of this document.

Reporting requirements will also be based on the Investigator's assessment of causality and seriousness, with allowance for upgrading by the Sponsor as needed.

Use of this document is governed by the terms of use on the first page of this document.

Appendix 3
Procedures for Recording Adverse Events

Investigators should use correct medical terminology/concepts when recording adverse events (AEs) on the Adverse Event electronic case report form (eCRF). Avoid colloquialisms and abbreviations.

Only one AE term should be recorded in the event field on the Adverse Event eCRF.

1. DIAGNOSIS VERSUS SIGNS AND SYMPTOMS

For AEs, a diagnosis (if known) should be recorded on the Adverse Event eCRF rather than individual signs and symptoms (e.g., record only liver failure or hepatitis rather than jaundice, asterixis, and elevated transaminases). However, if a constellation of signs and/or symptoms cannot be medically characterized as a single diagnosis or syndrome at the time of reporting, each individual event should be recorded on the Adverse Event eCRF. If a diagnosis is subsequently established, all previously reported AEs based on signs and symptoms should be nullified and replaced by one AE report that is based on the single diagnosis, with a starting date that corresponds to the starting date of the first symptom of the eventual diagnosis.

2. ADVERSE EVENTS OCCURRING SECONDARY TO OTHER EVENTS

In general, AEs occurring secondary to other events (e.g., cascade events or clinical sequelae) should be identified by their primary cause, with the exception of severe or serious secondary events. However, medically significant AEs occurring secondary to an initiating event that are separated in time should be recorded as independent events on the Adverse Event eCRF. For example:

- If vomiting results in mild dehydration with no additional treatment in a healthy adult, only vomiting should be reported on the eCRF.
- If vomiting results in severe dehydration, both events should be reported separately on the eCRF.
- If a severe gastrointestinal hemorrhage leads to renal failure, both events should be reported separately on the eCRF.
- If dizziness leads to a fall and subsequent fracture, all three events should be reported separately on the eCRF.

All AEs should be recorded separately on the Adverse Event eCRF if it is unclear as to whether the events are associated.

3. PERSISTENT OR RECURRENT ADVERSE EVENTS

A persistent AE is one that extends continuously, without resolution, between participant evaluation timepoints. Such events should only be recorded once on the Adverse Event

Use of this document is governed by the terms of use on the first page of this document.

eCRF. The initial severity of the event should be recorded, and the severity should be updated to reflect the most extreme severity any time the event worsens. If the event becomes serious, the Adverse Event eCRF should be updated to reflect this.

A recurrent AE is one that resolves between participant evaluation timepoints and subsequently recurs. Each recurrence of an AE should be recorded separately on the Adverse Event eCRF.

4. ABNORMAL LABORATORY VALUES

Not every laboratory abnormality qualifies as an AE. A laboratory test result should be reported as an AE if it meets any of the following criteria:

- Accompanied by clinical symptoms.
- Results in a change in study treatment (e.g., dosage modification, treatment interruption, or treatment discontinuation).
- Results in a medical intervention (e.g., potassium supplementation for hypokalemia) or a change in concomitant therapy.
- Clinically significant in the Investigator's judgment.

It is the Investigator's responsibility to review all laboratory findings. Medical and scientific judgment should be exercised in deciding whether an isolated laboratory abnormality should be classified as an AE.

If a clinically significant laboratory abnormality is a sign of a disease or syndrome (e.g., alkaline phosphatase and bilirubin 5 times the upper limit of normal [ULN] associated with cholecystitis), only the diagnosis (i.e., cholecystitis) should be recorded on the Adverse Event eCRF.

If a clinically significant laboratory abnormality is not a sign of a disease or syndrome, the abnormality itself should be recorded on the Adverse Event eCRF, along with a descriptor indicating if the test result is above or below the normal range (e.g., "elevated potassium," as opposed to "abnormal potassium"). If the laboratory abnormality can be characterized by a precise clinical term per standard definitions, the clinical term should be recorded as the AE. For example, an elevated serum potassium level of 7.0 mEq/L should be recorded as "hyperkalemia."

Observations of the same clinically significant laboratory abnormality from visit to visit should not be repeatedly recorded on the Adverse Event eCRF, unless the etiology changes. The initial severity of the event should be recorded, and the severity or seriousness should be updated any time the event worsens.

Use of this document is governed by the terms of use on the first page of this document.

5. ABNORMAL VITAL SIGN VALUES

Not every vital sign abnormality qualifies as an AE. A vital sign result should be reported as an AE if it meets any of the following criteria:

- Accompanied by clinical symptoms.
- Results in a change in study treatment (e.g., dosage modification, treatment interruption, or treatment discontinuation).
- Results in a medical intervention or a change in concomitant therapy.
- Clinically significant in the Investigator's judgment.

It is the Investigator's responsibility to review all vital sign findings. Medical and scientific judgment should be exercised in deciding whether an isolated vital sign abnormality should be classified as an AE.

If a clinically significant vital sign abnormality is a sign of a disease or syndrome (e.g., high blood pressure), only the diagnosis (i.e., hypertension) should be recorded on the Adverse Event eCRF.

Observations of the same clinically significant vital sign abnormality from visit to visit should not be repeatedly recorded on the Adverse Event eCRF, unless the etiology changes. The initial severity of the event should be recorded, and the severity or seriousness should be updated any time the event worsens.

6. ABNORMAL LIVER FUNCTION TESTS

For participants without liver metastases, the finding of an elevated alanine aminotransferase (ALT) or aspartate aminotransferase (AST) ($> 3 \times$ ULN) in combination with either an elevated total bilirubin ($> 2 \times$ ULN) or clinical jaundice in the absence of cholestasis or other causes of hyperbilirubinemia is considered to be an indicator of severe liver injury (as defined by Hy's Law). Therefore, Investigators must report as an AE the occurrence of either of the following:

- Treatment-emergent ALT or AST $> 3 \times$ ULN in combination with total bilirubin $> 2 \times$ ULN.
- Treatment-emergent ALT or AST $> 3 \times$ ULN in combination with clinical jaundice.

For participants with liver metastases, the finding of an elevated ALT or AST in combination with either an elevated total bilirubin or clinical jaundice in the absence of cholestasis or other causes of hyperbilirubinemia is considered to be an indicator of potential severe liver injury. Therefore, Investigators must report as an AE the occurrence of either of the following:

- Treatment-emergent ALT or AST $> 5 \times$ ULN value in combination with total bilirubin $> 2 \times$ ULN (of which $\geq 35\%$ is direct bilirubin).

Use of this document is governed by the terms of use on the first page of this document.

- Treatment-emergent ALT or AST > 5 × ULN value in combination with clinical jaundice.

The most appropriate diagnosis or (if a diagnosis cannot be established) the abnormal laboratory values should be recorded on the Adverse Event eCRF (see Appendix 2) and reported to the Sponsor immediately (i.e., no more than 24 hours after learning of the event), either as a serious AE (SAE) or a AE of special interest (see Section 8.3.6).

7. DEATHS

For this protocol, mortality is an efficacy endpoint. Deaths that occur during the protocol-specified AE reporting period (see Section 5 of Appendix 2) that are attributed by the Investigator solely to progression of disease under study should be recorded only on the Death Attributed to Progressive Disease eCRF. All other on-study deaths, regardless of relationship to study treatment, must be recorded on the Adverse Event eCRF and immediately reported to the Sponsor (see Section 5 of Appendix 2).

Death should be considered an outcome and not a distinct event. The event or condition that caused or contributed to the fatal outcome should be recorded as the single medical concept on the Adverse Event eCRF. Generally, only one such event should be reported. If the cause of death is unknown and cannot be ascertained at the time of reporting, “unexplained death” should be recorded on the Adverse Event eCRF. If the cause of death later becomes available (e.g., after autopsy), “unexplained death” should be replaced by the established cause of death. The term “sudden death” should not be used unless combined with the presumed cause of death (e.g., “sudden cardiac death”).

8. PREEXISTING MEDICAL CONDITIONS

A preexisting medical condition is one that is present at the screening visit for this study. Such conditions should be recorded on the General Medical History and Baseline Conditions eCRF.

A preexisting medical condition should be recorded as an AE only if the frequency, severity, or character of the condition worsens during the study. When recording such events on the Adverse Event eCRF, it is important to convey the concept that the preexisting condition has changed by including applicable descriptors (e.g., “more

9. LACK OF EFFICACY OR WORSENING OF UNDERLYING DISEASE

Events that are clearly consistent with the expected pattern of progression of the underlying disease should not be recorded as AEs. These data will be captured as efficacy assessment data only. In most cases, the expected pattern of progression will be based on RECIST v1.1. In rare cases, the determination of clinical progression will be based on symptomatic deterioration. However, every effort should be made to document

Use of this document is governed by the terms of use on the first page of this document.

progression using objective criteria. If there is any uncertainty as to whether an event is due to progressive disease, it should be reported as an AE.

10. HOSPITALIZATION OR PROLONGED HOSPITALIZATION

Any AE that results in hospitalization or prolonged hospitalization should be documented and reported as an SAE (per the definition of SAEs in Appendix 2), except as outlined below.

An event that leads to hospitalization under the following circumstances should not be reported as an AE or an SAE:

- Hospitalization for respite care.
- Planned hospitalization required by the protocol e.g., for study treatment administration or insertion of access device for study treatment administration
- Hospitalization for a preexisting condition, provided that all of the following criteria are met:
  - The hospitalization was planned prior to the study or was scheduled during the study (e.g., when elective surgery became necessary because of the expected normal progression of the disease).
  - The participant has not suffered an AE.
- Hospitalization due solely to progression of the underlying cancer.

An event that leads to hospitalization under the following circumstances is not considered to be an SAE, but should be reported as an AE instead:

- Hospitalization for an AE that would ordinarily have been treated in an outpatient setting had an outpatient clinic been available.

11. PATIENT-REPORTED OUTCOME DATA (CLINICAL OUTCOME ASSESSMENTS DATA REPORTED DIRECTLY BY PARTICIPANTS)

AE reports will not be derived from patient-reported outcome (PRO) data by the Sponsor, and safety analyses will not be performed using PRO data. Sites are not expected to review the PRO data.

Use of this document is governed by the terms of use on the first page of this document.

Appendix 4
Clinical Laboratory Tests

The tests detailed in Table 1 will be performed by the local laboratory in accordance with local laws and regulations. The results of these clinical laboratory tests must be captured in source documentation and entered into the electronic case report form (eCRF).

Protocol-specific requirements for inclusion or exclusion of participants are detailed in Sections 5.1 and 5.2, respectively, of the protocol.

Additional tests may be performed at any time during the study as determined necessary by the Investigator or required by local regulations.

Table 1 Protocol-Required Safety Laboratory Assessments

Laboratory Assessments	Parameters
Hematology	• Leucocytes, erythrocytes, hemoglobin, hematocrit, platelets, differential count (neutrophils, eosinophils, basophils, monocytes, lymphocytes, other cells).
Clinical Chemistry	• Sodium, potassium, chloride, bicarbonate, glucose, urea, creatinine, creatine kinase, protein, albumin, phosphate, calcium, magnesium, GGT, total and direct bilirubin, alkaline phosphatase, ALT, AST, urate, LDH, CRP, ferritin*, lipase and amylase*.
[redacted]	
Lipid Panel	• Total cholesterol, HDL, LDL, and triglycerides
Coagulation	• INR, aPTT, PT.
Viral Serology	• HIV (specific tests HIV-1 antibody, HIV-1/2 antibody, HIV-2 antibody), hepatitis B surface antigen (hBsAg) *or DNA*, total hepatitis B core antibody (hBcAb), hepatitis C virus (HCV) antibody *or RNA*.
Pregnancy Test	• All women of childbearing potential will have a blood pregnancy test at screening within 7 days before first dose of study treatment. Urine or serum pregnancy tests will be performed at specified subsequent visits. If a urine pregnancy test is positive, it must be confirmed by a blood pregnancy test.

Use of this document is governed by the terms of use on the first page of this document.

Laboratory Assessments	Parameters
Urinalysis	• Specific gravity<br>• Dipstick: pH, glucose, protein, blood, ketones, bilirubin, urobilinogen, nitrite, leukocyte esterase<br>• If there is a clinically significant positive result (confirmed by a positive repeated sample), urine will be sent to the laboratory for microscopy and culture. If there is an explanation for the positive dipstick results (e.g., menses), it should be recorded and there is no need to perform microscopy and culture.<br>• Microscopic examination (sediment, RBCs, WBCs, casts, crystals, epithelial cells, bacteria), if blood or protein is abnormal.

ALT=alanine aminotransferase; aPTT=activated partial thromboplastin time; AST=aspartate aminotransferase; CRP=C-reactive protein; CRS=cytokine release syndrome; HDL=high-density lipoproteins; HIV=human immunodeficiency virus; INR=international normalized ratio; IRR=infusion related reactions; LDH = lactate dehydrogenase; LDL=low-density lipoproteins; PT=prothrombin time; RBC=red blood cell; WBC=white blood cell.

The results of each test must be entered into the eCRF.

Investigators must document their review of each laboratory safety report.

Additional Statistical Considerations for Clinical Laboratory Data

Standard Reference Ranges and Transformation of Data

- Potential analysis considerations for analyzing laboratory data includes the use of standard reference ranges and potential transformation of data for specific laboratory tests.
- In this scenario, Roche standard reference ranges, rather than the reference ranges of the Investigator, can be used for specific parameters. For these parameters, the measured laboratory test result will be assessed directly using the Roche standard reference range. Certain laboratory parameters will be transformed to Roche's standard reference ranges.

A transformation will be performed on certain laboratory tests that lack sufficiently common procedures and have a wide range of Investigator ranges, e.g., enzyme tests that include AST, ALT, alkaline phosphatase, and total bilirubin. The standard reference ranges for these parameters have a lower limit of zero; therefore, only the upper limits of the ranges will be used in transforming the data.

Use of this document is governed by the terms of use on the first page of this document.

Definition of Laboratory Abnormalities

For all laboratory parameters included in the analysis described above, there exists a Roche predefined standard reference range. Laboratory values falling outside this standard reference range will be labeled “H” for high or “L” for low in participant statistical outputs of laboratory data.

In addition to the standard reference range, a marked reference range has been predefined by Roche for these laboratory parameters. The marked reference range is broader than the standard reference range. Values falling outside the marked reference range that also represent a defined change from baseline will be considered marked laboratory abnormalities (i.e., potentially clinically relevant). If a baseline value is not available for a participant, the midpoint of the standard reference range will be used as the participant’s baseline value for the purposes of determining marked laboratory abnormalities. Marked laboratory abnormalities will be labeled in the participant listings as “HH” for very high or “LL” for very low.

Use of this document is governed by the terms of use on the first page of this document.

Appendix 5
Contraceptive and Barrier Methods Guidance

1. DEFINITIONS

Participant of Childbearing Potential

A participant is considered fertile following menarche and until becoming postmenopausal unless permanently sterile. The definition of childbearing potential may be adapted for alignment with local guidelines or requirements.

Participants in the following categories are considered to be of Non-Childbearing Potential

- Pre-menarchal
- Premenopausal females with one of the following:
  - Documented hysterectomy.
  - Documented bilateral salpingectomy.
  - Documented bilateral oophorectomy.

  Note: Documentation can come from the site personnel's: review of participant's medical records, medical examination, or medical history interview.

- Postmenopausal participants:
  - A postmenopausal state is defined as no menses for ≥ 12 months without an alternative medical cause other than menopause. A high follicle-stimulating hormone (FSH) level in the postmenopausal range may be used to confirm a postmenopausal state in participants not using hormonal contraception or hormonal replacement therapy (HRT). However, in the absence of 12 months of amenorrhea, a single FSH measurement is insufficient.
  - Participants on HRT and whose menopausal status is in doubt will be required *to follow the contraceptive methods requirements as per the eligibility criteria, in Section 5.1.*

2. *DEFINITION OF HIGHLY/EFFECTIVE CONTRACEPTIVE METHOD*

Note: not all methods listed below are acceptable in the study, participants should follow the contraception methods requirements as per the eligibility criteria in Section 5.1.

Per International Council for Harmonisation (ICH) M3(R2), highly effective methods of birth control are defined as those, alone or in combination, that result in a low failure rate (i.e., less than 1% per year) when used consistently and correctly as described in Table 1 below. Effective or acceptable methods of birth control are defined as those,

Use of this document is governed by the terms of use on the first page of this document.

alone or in combination, which result in failure rate of > 1% per year when used consistently and correctly, as described in Table 1 below.

Table 1 *Overview of Effective and* **Highly Effective Contraceptive Methods** *as per International Council for Harmonisation (ICH) M3(R2)*

**Highly Effective Contraceptive Methods That Are User-Dependent[a]**<br>(Failure rate of < 1% per year when used consistently and correctly)
• Combined (estrogen- and progestogen-containing) hormonal contraception associated with inhibition of ovulation:<br>○ Oral<br>○ Intravaginal<br>○ Transdermal<br>• Progestogen-only hormonal contraception associated with inhibition of ovulation:<br>○ Oral<br>○ Injectable
**Highly Effective Methods That Are User-Independent**<br>(Failure rate of < 1% per year)
• Implantable progestogen-only hormonal contraception associated with inhibition of ovulation[a]<br>• Intrauterine device<br>• Intrauterine hormone-releasing system<br>• Bilateral tubal occlusion/ligation<br>**Azoospermic partner (vasectomized or due to medical cause)**<br>A vasectomized partner is a highly effective contraception method provided that the partner is the sole male sexual partner of the POCBP and the absence of sperm has been confirmed. If not, an additional highly effective method of contraception should be used.<br>**Sexual abstinence**<br>Sexual abstinence is considered a highly effective method only if defined as refraining from heterosexual intercourse during the entire period of risk associated with the study treatment. The reliability of sexual abstinence needs to be evaluated in relation to the duration of the study and the preferred and usual lifestyle of the participant.
**Effective Birth Control Methods Which May Not Be Considered As Highly Effective**<br>(Failure rate of ≥ 1% per year when used consistently and correctly)
• Progestogen-only oral hormonal contraception, where inhibition of ovulation is not the primary mode of action<br>• Male or female condom with or without spermicide[b]<br>• Cap, diaphragm, or sponge with spermicide[b]

Use of this document is governed by the terms of use on the first page of this document.

Abbreviations: POCBP = Participant of childbearing potential.

a) Hormonal contraception may be susceptible to interaction with the study treatment, which may reduce the efficacy of the contraception method.

Typical use failure rates may differ from those when used consistently and correctly. Use should be consistent with local regulations regarding the use of contraceptive methods for participants participating in clinical studies.

b) A combination of male condom with either cap, diaphragm, or sponge with spermicide (double-barrier methods) are also considered acceptable, but not highly effective, birth control methods (i.e., when the risk of teratogenicity and genotoxicity is unlikely).

3. PREGNANCY TESTING

For POCBP enrolled in the study, blood sample and urine pregnancy tests will be performed according to the SoA (see Section 1.3). If a highly sensitive urine pregnancy test is positive, it must be confirmed by a blood pregnancy test.

Pregnancy testing will be performed whenever a menstrual cycle is missed or when pregnancy is otherwise suspected and according to local practice.

4. COLLECTION OF PREGNANCY INFORMATION

Male Participants with Partners who Become Pregnant

The Investigator will attempt to collect pregnancy information on any male participant's partner who becomes pregnant while the male participant is in this study or within 4 months after the final dose of study treatment (see Section 8.3.5 Pregnancy). This applies only to male participants who receive RO7589831.

Attempts should be made to collect and report details of the course and outcome of any pregnancy in the partner of a male participant exposed to study treatment. The Investigator will record pregnancy information on the Clinical Trial Pregnancy Reporting Form and submit it to the Sponsor within 24 hours of learning of the partner's pregnancy. When permitted by the site, the pregnant partner would need to sign an Authorization for Use and Disclosure of Pregnancy Health Information and an Authorization for Use and Disclosure of Infant Health Information to allow for follow-up on her pregnancy and the infant health information respectively. If the authorization has been signed, the Investigator should update the Clinical Trial Pregnancy Reporting Form with additional information on the course and outcome of the pregnancy when available.

An Investigator who is contacted by the male participant or his pregnant partner may provide information on the risks of the pregnancy and the possible effects on the fetus, to support an informed decision in cooperation with the treating physician and/or obstetrician. The partner will be followed to determine the outcome of the pregnancy. Information on the status of the mother and child will be forwarded to the Sponsor. Monitoring of the participant's partner should continue until conclusion of the pregnancy. Any termination of the pregnancy will be reported, regardless of fetal status (presence or absence of anomalies) or indication for procedure.

Use of this document is governed by the terms of use on the first page of this document.

Information on the status of the infant health at the 6th and 12th month after the date of birth will be forwarded to the Sponsor by using Infant Clinical Trial Follow-up Reporting Form when the authorization for infant health follow-up has been obtained.

Participants who Become Pregnant

The Investigator will collect pregnancy information on any participant who becomes pregnant while participating in this study (see Section 8.3.5 Pregnancy). Information will be recorded on the Clinical Trial Pregnancy Reporting Form submitted to the Sponsor within 24 hours of learning of a participant's pregnancy. The participant will be followed to determine the outcome of the pregnancy. Monitoring of the participant should continue until conclusion of the pregnancy. Any termination of pregnancy will be reported, regardless of fetal status (presence or absence of anomalies) or indication for procedure. The Investigator will collect follow-up information on the participant and the neonate which will be forwarded to the Sponsor. When permitted by the site, the pregnant participant would need to sign an Authorization for the Use and Disclosure of Infant Health Information to allow for follow-up on the infant. Information on the status of the infant health at the 6th and 12th month after the date of birth will be forwarded to the Sponsor by using Infant Clinical Trial Follow-up Reporting Form when the authorization for infant health follow-up has been obtained.

While pregnancy itself is not considered to be an adverse event (AE) or serious AE (SAE) and should not be recorded on the AE eCRF, any pregnancy complication will be reported as an AE or SAE. A spontaneous abortion is always considered to be an SAE and will be reported as such. Any post-study, pregnancy-related SAE considered reasonably related to the study treatment by the Investigator will be reported to the Sponsor as described in Appendix 2. While the Investigator is not obligated to actively seek this information in former study participants, he/she may learn of an SAE through spontaneous reporting.

Any participant who becomes pregnant while participating in the study will discontinue study treatment.

Additionally, attempts should be made to collect and report infant health information. When permitted by the site, an Authorization for the Use and Disclosure of Infant Health Information would need to be signed by one or both parents (as per local regulations) to allow for follow-up on the infant. If the authorization has been signed, the infant's health status at birth should be recorded on the Clinical Trial Pregnancy Reporting Form. In addition, the Sponsor may collect follow-up information on the infant's health status at 6 and 12 months after birth.

5 ABORTIONS

Any spontaneous abortion in a participant exposed to study treatment or the partner of a male participant exposed to study treatment should be classified as an SAE (as the

Use of this document is governed by the terms of use on the first page of this document.

Sponsor considers spontaneous abortions to be medically significant events), recorded on the Adverse Event eCRF, and reported to the Sponsor immediately (i.e., no more than 24 hours after learning of the event; see Section 5 of Appendix 2).

Any induced abortion due to maternal toxicity and/or embryofetal toxicity should also be classified as an SAE, recorded on the Adverse Event eCRF, and reported to the Sponsor immediately (i.e., no more than 24 hours after learning of the event; see Section 5 of Appendix 2).

Elective or therapeutic abortion not associated with an underlying maternal or embryofetal toxicity (e.g., induced abortion for personal reasons) does not require expedited reporting is not considered an AE but should be reported as outcome of pregnancy on the Clinical Trial Pregnancy Reporting Form.

All abortions should be reported as pregnancy outcomes on the paper Clinical Trial Pregnancy Reporting Form.

6 CONGENITAL ANOMALIES/BIRTH DEFECTS

Any congenital anomaly/birth defect in a child born to a participant or partner of a male participant exposed to study treatment should be classified as an SAE, recorded on the Adverse Event eCRF, and reported to the Sponsor immediately (i.e., no more than 24 hours after learning of the event).

Use of this document is governed by the terms of use on the first page of this document.

Appendix 6
Investigational Medicinal Product and Non-Investigational Medicinal Product Designations for Use in United Kingdom

Table 1 Investigational *and Non-investigational* Medicinal Product Designations for *United Kingdom*

Product Name	IMP Designation	Marketing Authorization Status in *UK*	Used within Marketing Authorization
RO7589831	IMP (test product)	Unauthorized	Not applicable
Pembrolizumab[a]	*IMP (test product)*	*Authorized*	*No*

IMP = investigational medicinal product; *UK = United Kingdom.*

[a] *Pembrolizumab is approved for the treatment of microsatellite instability high (MSI-H) or mismatch repair deficient (dMMR) cancers, but not in combination with RO7589831.*

Use of this document is governed by the terms of use on the first page of this document.

Appendix 7
Guidance for Fluoro-deoxy-glucose Positron Emission Tomography

Introduction

[^{18}F] fluoro-deoxy-glucose(FDG) positron emission tomography (PET) will be performed according to the Schedule of Activities (Section 1.3) as a non-invasive method for determining drugs effect on the metabolic activity of tumor.

The participant needs to fast for at least 4 hours prior to the FDG-PET scan. The participant can drink water freely during the fast and should be encouraged to do so. Blood glucose level will be checked (typically at the PET center) on the day of the FDG-PET scan and results assessed prior to the administration of FDG. The participant should have a blood glucose level ≤ 180 mg/dL (≤ 10 mmol/mL) to have the FDG-PET scan. If the level is higher, the scan should be rescheduled if possible. Regular diet can be resumed after the scan. The interval between FDG administration and scanning must be 60 minutes ± 10 minutes and it is particularly important that the time interval between injection and start of the scan is the same at follow-up compared to baseline.

Importantly, PET must be performed before biopsy to avoid potential false positive findings.

All participants should be encouraged to increase fluid intake for a few hours after the scan to promote excretion of the FDG. A diuretic (furosemide, typically 20-40 mg intravenous) may be administered at the discretion of the Investigator before or during the FDG-PET scan in order to accelerate elimination of the [^{18}F]-FDG from the renal collecting system. Diazepam may be used to promote muscle relaxation and reduce muscular uptake if tumor deposits are in the neck or shoulder girdle area. Diazepam administration and diuretic administration must be recorded in the Concomitant Medications eCRF page.

Participants who have had a hypersensitivity reaction to FDG will be excluded from [^{18}F]-FDG-PET. However, the participant will be eligible for the remainder of the study.

Participants with no evidence of FDG uptake at the screening PET assessment will not be required to undergo the on-study assessment. For participants with suggestion of progressive disease on follow-up [^{18}F]-FDG-PET assessments, a confirmatory CT/MRI assessment is strongly encouraged.

Acquisition and Processing

Scan Acquisition

Use of this document is governed by the terms of use on the first page of this document.

Attenuation corrected FDG-PET scans will be performed. The participant will be administered 370–740 MBq (10-20 mCi) FDG IV (dose is dependent on local practice and scanner type). The administered activity and time of FDG administration and body weight on the day of scanning must be recorded for subsequent calculation of maximum tumor standardized uptake value (SUV_{max}). Approximately one hour following the administration of FDG, a whole-body PET scan (typically from base of skull to thighs) is performed and the time of the commencement of the scanning is recorded. The sequence of the scan must be the same for the initial scan and all follow-up scans, and the interval between FDG administration and scanning should be as similar as practical. Within the restrictions of hardware and software differences in the scanners at the sites, the FDG-PET acquisition protocol will be standardized across sites and will utilize a PET/CT scanner where possible (as opposed to a dedicated PET scanner) to allow for correction of the emission scan by a low dose CT transmission scan. Preferably, the same scanner should be used for all participants at a given site, and for a given participant the same scanner should be used at all time-points.

Local Scan Interpretation

The PET/CT scans will be analyzed for body weight corrected SUV_{max} uptake by an experienced reader (the same reader should interpret all scans for each participant). Additionally, total lesion glycolysis (TLG) and metabolic tumor volume (MTV) may be measured. Also, a visual assessment of extent, intensity and changes in FDG pattern of uptake, including appearance of new FDG lesions will be made.

Central Scan Interpretation

In addition to the site review of FDG-PET scans, an independent central review will be performed by a designated imaging contract research organization (CRO).

The FDG-PET images will be de-identified at the site and securely transmitted so that the images can be analyzed centrally by an experienced reader at the CRO.

Use of this document is governed by the terms of use on the first page of this document.

Appendix 8
Response Evaluation Criteria in Solid Tumors, Version 1.1: Excerpt from Original Publication

Selected sections from the Response Evaluation Criteria in Solid Tumors, Version 1.1 (RECIST v1.1) (Eisenhauer et al. 2009) are presented below, with the addition of explanatory text as needed for clarity.

Tumor Measurability

At baseline, tumor lesions/lymph nodes will be categorized as measurable or non-measurable as described below. All measurable and non-measurable lesions should be assessed at screening and at subsequent protocol-specified tumor assessment timepoints. Additional assessments may be performed as clinically indicated for suspicion of progression.

Measurable Tumor Lesions

Tumor Lesions

Tumor lesions must be accurately measured in at least one dimension (longest diameter in the plane of measurement is to be recorded) with a minimum size as follows:

- 10 mm by computed tomography (CT) or magnetic resonance imaging (MRI) scan (CT/MRI scan slice thickness/interval no greater than 5 mm)
- 10-mm caliper measurement by clinical examination (lesions that cannot be accurately measured with calipers should be recorded as non-measurable)
- 20 mm by chest X-ray

Malignant Lymph Nodes

To be considered pathologically enlarged and measurable, a lymph node must be ≥ 15 mm in the short axis when assessed by CT scan (CT scan slice thickness recommended to be no greater than 5 mm). At baseline and follow-up, only the short axis will be measured and followed. Additional information on lymph node measurement is provided below (see “Identification of Target and Non-Target Lesions” and “Calculation of Sum of Diameters”).

Non-Measurable Tumor Lesions

Non-measurable tumor lesions encompass small lesions (longest diameter < 10 mm or pathological lymph nodes with ≥ 10 to < 15 mm short axis) as well as truly non-measurable lesions. Lesions considered truly non-measurable include leptomeningeal disease, ascites, pleural or pericardial effusion, inflammatory breast disease, lymphangitic involvement of skin or lung, peritoneal spread, and abdominal

Use of this document is governed by the terms of use on the first page of this document.

mass/abdominal organomegaly identified by physical examination that is not measurable by reproducible imaging techniques.

Special Considerations Regarding Lesion Measurability

Bone lesions, cystic lesions, and lesions previously treated with local therapy require particular comment, as outlined below.

Bone Lesions

- Technetium-99m bone scans, sodium fluoride positron emission tomography scans, and plain films are not considered adequate imaging techniques for measuring bone lesions. However, these techniques can be used to confirm the presence or disappearance of bone lesions.
- Lytic bone lesions or mixed lytic-blastic lesions with identifiable soft tissue components that can be evaluated by cross-sectional imaging techniques such as CT or MRI can be considered measurable lesions if the soft tissue component meets the definition of measurability described above.
- Blastic bone lesions are non-measurable.

Cystic Lesions

- Lesions that meet the criteria for radiographically defined simple cysts should not be considered malignant lesions (neither measurable nor non-measurable) since they are, by definition, simple cysts.
- Cystic lesions thought to represent cystic metastases can be considered measurable lesions if they meet the definition of measurability described above. However, if non-cystic lesions are present in the same participant, these are preferred for selection as target lesions.

Lesions with Prior Local Treatment

- Tumor lesions situated in a previously irradiated area or in an area subjected to other loco-regional therapy are usually not considered measurable unless there has been demonstrated progression in the lesion.

Methods for Assessing Lesions

All measurements should be recorded in metric notation, using calipers if clinically assessed. All baseline evaluations should be performed as close as possible to the treatment start and never more than 4 weeks before the beginning of the treatment.

The same method of assessment and the same technique should be used to characterize each identified and reported lesion at baseline and during the study. Imaging-based evaluation should always be the preferred option.

Use of this document is governed by the terms of use on the first page of this document.

Clinical Lesions

Clinical lesions will only be considered measurable when they are superficial and ≥ 10 mm in diameter as assessed using calipers (e.g., skin nodules). For the case of skin lesions, documentation by color photography, including a ruler to estimate the size of the lesion, is suggested.

Chest X-Ray

Chest CT is preferred over chest X-ray, particularly when progression is an important endpoint, since CT is more sensitive than X-ray, particularly in identifying new lesions. However, lesions on chest X-ray may be considered measurable if they are clearly defined and surrounded by aerated lung.

CT/MRI

CT is the best currently available and reproducible method to measure lesions selected for response assessment. In this guideline, the definition of measurability of lesions on CT scan is based on the assumption that CT slice thickness is 5 mm or less. When CT scans have slice thickness greater than 5 mm, the minimum size for a measurable lesion should be twice the slice thickness. MRI is also acceptable.

If prior to enrollment it is known that a participant is unable to undergo CT scans with intravenous contrast because of allergy or renal insufficiency, the decision as to whether a non-contrast CT or MRI (without intravenous contrast) will be used to evaluate the participant at baseline and during the study should be guided by the tumor type under investigation and the anatomic location of the disease. For participants who develop contraindications to contrast after baseline contrast CT is done, the decision as to whether non-contrast CT or MRI (enhanced or non-enhanced) will be performed should also be based on the tumor type and the anatomic location of the disease and should be optimized to allow for comparison with the prior studies if possible. Each case should be discussed with the radiologist to determine if substitution of these other approaches is possible and, if not, the participant should be considered not evaluable from that point forward. Care must be taken in measurement of target lesions and interpretation of non-target disease or new lesions on a different modality, since the same lesion may appear to have a different size using a new modality.

Endoscopy, Laparoscopy, Ultrasound, Tumor Markers, Cytology, and Histology

The utilization of these techniques for objective tumor evaluation cannot generally be advised.

Use of this document is governed by the terms of use on the first page of this document.

TUMOR RESPONSE EVALUATION

ASSESSMENT OF OVERALL TUMOR BURDEN AND MEASURABLE DISEASE

To assess objective response or future progression, it is necessary to estimate the overall tumor burden at baseline and use this as a comparator for subsequent measurements.

BASELINE DOCUMENTATION OF TARGET AND NON-TARGET LESIONS

When more than one measurable lesion is present at baseline, all lesions up to a maximum of five lesions total (and a maximum of two lesions per organ) representative of all involved organs should be identified as target lesions and will be recorded and measured at baseline. This means that, for instances in which participants have only one or two organ sites involved, a maximum of two lesions (one site) and four lesions (two sites), respectively, will be recorded. Other lesions (albeit measurable) in those organs will be recorded as non-measurable lesions (even if the size is >10 mm by CT scan).

Target lesions should be selected on the basis of their size (lesions with the longest diameter) and should be representative of all involved organs, but in addition should lend themselves to reproducible repeated measurements. It may be the case that, on occasion, the largest lesion does not lend itself to reproducible measurement, in which circumstance the next largest lesion that can be measured reproducibly should be selected.

Lymph nodes merit special mention since they are normal anatomical structures that may be visible by imaging even if not involved by tumor. As noted above, pathological nodes that are defined as measurable and may be identified as target lesions must meet the criterion of a short axis of ≥15 mm by CT scan. Only the short axis of these nodes will contribute to the baseline sum. The short axis of the node is the diameter normally used by radiologists to judge if a node is involved by solid tumor. Lymph node size is normally reported as two dimensions in the plane in which the image is obtained (for CT, this is almost always the axial plane; for MRI, the plane of acquisition may be axial, sagittal, or coronal). The smaller of these measures is the short axis. For example, an abdominal node that is reported as being 20 mm × 30 mm has a short axis of 20 mm and qualifies as a malignant, measurable node. In this example, 20 mm should be recorded as the node measurement. All other pathological nodes (those with short axis ≥10 mm but <15 mm) should be considered non-target lesions. Nodes that have a short axis of <10 mm are considered non-pathological and should not be recorded or followed.

All lesions (or sites of disease) not selected as target lesions (measurable or non-measurable), including pathological lymph nodes, should be identified as non-target lesions and should also be recorded at baseline. Measurements are not required. It is possible to record multiple non-target lesions involving the same organ as a single item

Use of this document is governed by the terms of use on the first page of this document.

on the Case Report Form (CRF) (e.g., “multiple enlarged pelvic lymph nodes” or “multiple liver metastases”).

Calculation of Sum of Diameters

A sum of the diameters (longest diameter for non-lymph node lesions, short axis for lymph node lesions) will be calculated for all target lesions at baseline and at each subsequent tumor assessment as a measure of tumor burden.

Measuring Lymph Nodes

Lymph nodes identified as target lesions should always have the actual short axis measurement recorded (measured in the same anatomical plane as the baseline examination), even if the node regresses to $<$10 mm during the study. Thus, when lymph nodes are included as target lesions, the sum of diameters may not be zero even if complete response criteria are met, since a normal lymph node is defined as having a short axis of $<$10 mm.

Target Lesions that Become too Small to Measure

During the study, all target lesions (lymph node and non-lymph node) recorded at baseline should have their actual measurements recorded at each subsequent evaluation, even when very small (e.g., 2 mm). However, sometimes lesions or lymph nodes that are recorded as target lesions at baseline become so faint on CT scan that the radiologist may not feel comfortable assigning an exact measurement and may report them as being too small to measure. When this occurs, it is important that a value be recorded on the eCRF, as follows:

- If it is the opinion of the radiologist that the lesion has likely disappeared, the measurement should be recorded as 0 mm.
- If the lesion is believed to be present and is faintly seen but too small to measure, a default value of 5 mm should be assigned and “too small to measure” should be ticked. (Note: It is less likely that this rule will be used for lymph nodes since they usually have a definable size when normal and are frequently surrounded by fat such as in the retroperitoneum; however, if a lymph node is believed to be present and is faintly seen but too small to measure, a default value of 5 mm should be assigned in this circumstance as well and “too small to measure” should also be ticked).

<u>To reiterate: If the radiologist is able to provide an actual measure, this measurement should be recorded, even if it is below 5 mm, and, in that case, too small to measure box should not be ticked.</u>

Use of this document is governed by the terms of use on the first page of this document.

Lesions that Split or Coalesce on Treatment

When non-nodal lesions fragment, the longest diameters of the fragmented portions should be added together to calculate the sum of diameters. Similarly, as lesions coalesce, a plane between them may be maintained that would aid in obtaining maximal diameter measurements of each individual lesion. If the lesions have truly coalesced such that they are no longer separable, the vector of the longest diameter in this instance should be the maximum longest diameter for the coalesced lesion.

Evaluation of Non-Target Lesions

Measurements are not required for non-target lesions, except that malignant lymph node non-target lesions should be monitored for reduction to $<$10 mm in short axis. Non-target lesions should be noted at baseline and should be identified as “present” or “absent” and (in rare cases) may be noted as “indicative of progression” at subsequent evaluations. In addition, if a lymph node lesion shrinks to a non-malignant size (short axis $<$10 mm), this should be captured on the CRF as part of the assessment of non-target lesions.

<u>Response Criteria</u>

Criteria for Target Lesions

Definitions of the criteria used to determine objective tumor response for target lesions are provided below:

- Complete response (CR):
  Disappearance of all target lesions
  Any pathological lymph nodes must have reduction in short axis to $<$10 mm.
- Partial response (PR):
  At least a 30% decrease in the sum of diameters of all target lesions, taking as reference the baseline sum of diameters, in the absence of CR
- Progressive disease:
  At least a 20% increase in the sum of diameters of target lesions, taking as reference the smallest sum of diameters at prior timepoints (including baseline).
- In addition to the relative increase of 20%, the sum of diameters must also demonstrate an absolute increase of at least5 mm.
- Stable disease (SD):
  Neither sufficient shrinkage to qualify for CR or PR nor sufficient increase to qualify for Progressive Disease

Use of this document is governed by the terms of use on the first page of this document.

Criteria for Non-Target Lesions

Definitions of the criteria used to determine the tumor response for the group of non-target lesions are provided below. While some non-target lesions may actually be measurable, they need not be measured and instead should be assessed only qualitatively at the timepoints specified in the SoA.

- CR: Disappearance of all non-target lesions. All lymph nodes must be non-pathological in size (< 10 mm short axis).
- Non-CR/Non-Progressive Disease: Persistence of one or more non-target lesions
- Progressive Disease: Unequivocal progression of existing non-target lesions

Special Notes on Assessment of Progression of Non-Target Lesions

Participants with Measurable and Non-Measurable Disease

For participants with both measurable and non-measurable disease to achieve unequivocal progression on the basis of the non-target lesions, there must be an overall level of substantial worsening in non-target lesions in a magnitude that, even in the presence of SD or PR in target lesions, the overall tumor burden has increased sufficiently to merit discontinuation of therapy. A modest increase in the size of one or more non-target lesions is usually not sufficient to qualify for unequivocal progression status. The designation of overall progression solely on the basis of change in non-target lesions in the face of SD or PR in target lesions will therefore be extremely rare.

New Lesions

The appearance of new malignant lesions denotes disease progression; therefore, some comments on detection of new lesions are important. There are no specific criteria for the identification of new radiographic lesions; however, the finding of a new lesion should be unequivocal, that is, not attributable to differences in scanning technique, change in imaging modality, or findings thought to represent something other than tumor (for example, some "new" bone lesions may be simply healing or flare of preexisting lesions). This is particularly important when the participant's baseline lesions show PR or CR. For example, necrosis of a liver lesion may be reported on a CT scan report as a "new" cystic lesion, which it is not.

A lesion identified during the study in an anatomical location that was not scanned at baseline is considered a new lesion and will indicate disease progression.

If a new lesion is equivocal, for example because of its small size, continued therapy and follow-up evaluation will clarify if it represents truly new disease. If repeat scans confirm there is definitely a new lesion, progression should be declared using the date of the initial scan.

Use of this document is governed by the terms of use on the first page of this document.

Criteria for Overall Response at a Single Timepoint

Table 1 provides a summary of the overall response status calculation at each response assessment timepoint for participants who have measurable disease at baseline.

When participants have non-measurable (therefore non-target) disease only, is to be used.

Table 1 Timepoint Response: Participants with Target Lesions (with or without Non-Target Lesions)

Target Lesions	Non-Target Lesions	New Lesions	Overall Response
CR	CR	No	CR
CR	Non-CR/non-PD	No	PR
CR	Not evaluated	No	PR
PR	Non-PD or not all evaluated	No	PR
SD	Non-PD or not all evaluated	No	SD
Not all evaluated	Non-PD	No	NE
PD	Any	Yes or no	PD
Any	PD	Yes or no	PD
Any	Any	Yes	PD

CR = complete response; NE = not evaluable; PD = progressive disease; PR = partial response; SD = stable disease.

Table 2 Timepoint Response: Participants with Non-Target Lesions Only

Non-Target Lesions	New Lesions	Overall Response
CR	No	CR
Non-CR/non-PD	No	Non-CR/non-PD [a]
Not all evaluated	No	NE
Unequivocal PD	Yes or no	PD
Any	Yes	PD

CR = complete response; NE = not evaluable; PD = progressive disease.

[a] "Non-CR/non-PD" is preferred over "stable disease" for non-target disease because stable disease is increasingly used as an endpoint for assessment of efficacy in some trials; thus, assigning "stable disease" when no lesions can be measured is not advised.

Use of this document is governed by the terms of use on the first page of this document.

Missing Assessments and Not-Evaluable Designation

When no imaging/measurement is done at all at a particular timepoint, the patient is not evaluable at that timepoint. If measurements are made on only a subset of target lesions at a timepoint, usually the case is also considered not evaluable at that timepoint, unless a convincing argument can be made that the contribution of the individual missing lesions would not change the assigned timepoint response (Table 3). This would be most likely to happen in the case of Progressive Disease. For example, if a patient had a baseline sum of 50 mm with three measured lesions and during the study only two lesions were assessed, but those gave a sum of 80 mm, the patient will have achieved Progressive Disease status, regardless of the contribution of the missing lesion.

Table 3 Best Overall Response When Confirmation is Required

Overall Response at First Timepoint	Overall Response at Subsequent Timepoint	Best Overall Response
CR	CR	CR
CR	PR	SD, PD, or PR [a]
CR	SD	SD, provided minimum duration for SD was met; otherwise, PD
CR	PD	SD, provided minimum duration for SD was met; otherwise, PD
CR	NE	SD, provided minimum duration for SD was met; otherwise, NE
PR	CR	PR
PR	PR	PR
PR	SD	SD
PR	PD	SD, provided minimum duration for SD was met; otherwise, PD
PR	NE	SD, provided minimum duration for SD was met; otherwise, NE
NE	NE	NE

CR = complete response; NE = not evaluable; PD = progressive disease; PR = partial response; SD = stable disease.

[a] If a CR is truly met at the first timepoint, any disease seen at a subsequent timepoint, even disease meeting PR criteria relative to baseline, qualifies as PD at that point (because disease must have reappeared after CR). Best response would depend on whether the minimum duration for SD was met. However, sometimes CR may be claimed when subsequent scans suggest small lesions were likely still present and in fact the participant had PR, not CR, at the first timepoint. Under these circumstances, the original CR should be changed to PR and the best response is PR.

Use of this document is governed by the terms of use on the first page of this document.

<u>Special notes on response assessment</u>

When nodal disease is included in the sum of target lesions and the nodes decrease to "normal" size (< 10 mm), they may still have a measurement reported on scans. This measurement should be recorded even though the nodes are normal in order not to overstate progression should it be based on increase in size of the nodes. As noted earlier, this means that participants with CR may not have a total sum of "zero" on the eCRF.

Participants with a global deterioration in health status requiring discontinuation of treatment without objective evidence of disease progression at that time should be reported as "symptomatic deterioration". Every effort should be made to document objective progression even after discontinuation of treatment. Symptomatic deterioration is not a descriptor of an objective response; it is a reason for stopping study therapy. The objective response status of such participants is to be determined by evaluation of target and non-target lesions as shown in Table 4.

For equivocal findings of progression (e.g., very small and uncertain new lesions; cystic changes or necrosis in existing lesions), treatment may continue until the next scheduled assessment. If at the next scheduled assessment, progression is confirmed, the date of progression should be the earlier date when progression was suspected.

In studies for which participants with advanced disease are eligible (i.e., primary disease still or partially present), the primary tumor should also be captured as a target or non-target lesion, as appropriate. This is to avoid an incorrect assessment of CR if the primary tumor is still present but not evaluated as a target or non-target lesion.

FDG PET is **not validated** for the use in clinical trials to determine the response but can identify new lesions and benefit clinical decisions.

Table 4 Use of FDG-PET Imaging to Identify New Lesions

**Baseline FDG-PET**	**Post-baseline FDG-PET**	**Determination**
Negative FDG-PET	Positive FDG-PET	New lesion (PD)
None	Positive FDG-PET corresponds to a new site of disease confirmed by CT/MRI	New lesion (PD)
None	Positive FDG-PET not confirmed as a new site of disease on CT/MRI	Additional follow-up CT/MRI scans are needed to determine if there is truly progression occurring at that site.<br>If so: New lesion (PD) with date of PD being the date of the initial abnormal FDG-PET scan date<br>If not: Not a new lesion

Use of this document is governed by the terms of use on the first page of this document.

Baseline FDG-PET	Post-baseline FDG-PET	Determination
None	Positive FDG-PET which corresponds to a pre-existing site of disease on CT/MRI that is not progressing on the basis of the anatomic images	Not a new lesion

CT/MRI = computed tomography/magnetic resonance imaging; FDG PET = fluoro deoxy glucose positron emission tomography; PD = progressive disease.

Reference

Eisenhauer EA, Therasse P, Bogaerts J, et al. New response evaluation criteria in solid tumors: revised RECIST guideline (version 1.1). Eur J Cancer 2009;45:228–47.

Use of this document is governed by the terms of use on the first page of this document.

Appendix 9 European Organization for Research and Treatment of Cancer- Function 17

ENGLISH

EORTC QLQ-F17

We are interested in some things about you and your health. Please answer all of the questions yourself by circling the number that best applies to you. There are no "right" or "wrong" answers. The information that you provide will remain strictly confidential.

	Not at All	A Little	Quite a Bit	Very Much
1. Do you have any trouble doing strenuous activities, like carrying a heavy shopping bag or a suitcase?	1	2	3	4
2. Do you have any trouble taking a long walk?	1	2	3	4
3. Do you have any trouble taking a short walk outside of the house?	1	2	3	4
4. Do you need to stay in bed or a chair during the day?	1	2	3	4
5. Do you need help with eating, dressing, washing yourself or using the toilet?	1	2	3	4

**During the past week:**	Not at All	A Little	Quite a Bit	Very Much
6. Were you limited in doing either your work or other daily activities?	1	2	3	4
7. Were you limited in pursuing your hobbies or other leisure time activities?	1	2	3	4
8. Have you had difficulty in concentrating on things, like reading a newspaper or watching television?	1	2	3	4
9. Did you feel tense?	1	2	3	4
10. Did you worry?	1	2	3	4
11. Did you feel irritable?	1	2	3	4
12. Did you feel depressed?	1	2	3	4
13. Have you had difficulty remembering things?	1	2	3	4
14. Has your physical condition or medical treatment interfered with your family life?	1	2	3	4
15. Has your physical condition or medical treatment interfered with your social activities?	1	2	3	4

For the following questions please circle the number between 1 and 7 that best applies to you

16. How would you rate your overall health during the past week?

1	2	3	4	5	6	7
Very poor						Excellent

17. How would you rate your overall quality of life during the past week?

1	2	3	4	5	6	7
Very poor						Excellent

© Copyright 2019 EORTC Quality of Life Group. All rights reserved.

SPECIMEN

Use of this document is governed by the terms of use on the first page of this document.

Appendix 10 Patient-Reported Outcomes Common Terminology Criteria for Adverse Events

NCI-PRO-CTCAE® CUSTOM SURVEY
National Cancer Institute (NCI)-Patient-Reported Outcomes (PRO)- Common Terminology Criteria for Adverse Events (CTCAE)® Item Library Version 1.0
English
Form Created on 01-December-2022
https://healthcaredelivery.cancer.gov/pro-ctcae/builder.html

As individuals go through treatment for their cancer they sometimes experience different symptoms and side effects. For each question, please select the one response that best describes your experiences over the past 7 days.

**1a.** In the last 7 days, what was the SEVERITY of your DECREASED APPETITE at its WORST?				
O None	O Mild	O Moderate	O Severe	O Very severe
**1b.** In the last 7 days, how much did DECREASED APPETITE INTERFERE with your usual or daily activities?				
O Not at all	OA little bit	O Somewhat	O Quite a bit	O Very much

**2a.** In the last 7 days, how OFTEN did you have NAUSEA?				
O Never	O Rarely	O Occasionally	O Frequently	O Almost constantly
**2b.** In the last 7 days, what was the SEVERITY of your NAUSEA at its WORST?				
O None	O Mild	O Moderate	O Severe	O Very severe

**3a.** In the last 7 days, how OFTEN did you have VOMITING?				
O Never	O Rarely	O Occasionally	O Frequently	O Almost constantly
**3b.** In the last 7 days, what was the SEVERITY of your VOMITING at its WORST?				
O None	O Mild	O Moderate	O Severe	O Very severe

**4a.** In the last 7 days, what was the SEVERITY of your CONSTIPATION at its WORST?				
O None	O Mild	O Moderate	O Severe	O Very severe

Use of this document is governed by the terms of use on the first page of this document.

**5a.** In the last 7 days, how OFTEN did you have LOOSE OR WATERY STOOLS (DIARRHEA/DIARRHOEA)?				
O Never	O Rarely	O Occasionally	O Frequently	O Almost constantly

**6a.** In the last 7 days, what was the SEVERITY of your SHORTNESS OF BREATH at its WORST?				
O None	O Mild	O Moderate	O Severe	O Very severe
**6b.** In the last 7 days, how much did your SHORTNESS OF BREATH INTERFERE with your usual or daily activities?				
O Not at all	O A little bit	O Somewhat	O Quite a bit	O Very much

**7a.** In the last 7 days, did you have any RASH?	
O Yes	O No

**8a.** In the last 7 days, what was the SEVERITY of your NUMBNESS OR TINGLING IN YOUR HANDS OR FEET at its WORST?				
O None	O Mild	O Moderate	O Severe	O Very severe
**8b.** In the last 7 days, how much did NUMBNESS OR TINGLING IN YOUR HANDS OR FEET INTERFERE with your usual or daily activities?				
O Not at all	O A little bit	O Somewhat	O Quite a bit	O Very much

**9a.** In the last 7 days, what was the SEVERITY of your PROBLEMS WITH CONCENTRATION at their WORST?				
O None	O Mild	O Moderate	O Severe	O Very severe
**9b.** In the last 7 days, how much did PROBLEMS WITH CONCENTRATION INTERFERE with your usual or daily activities?				
O Not at all	O A little bit	O Somewhat	O Quite a bit	O Very much

**10a.** In the last 7 days, how OFTEN did you have PAIN?				
O Never	O Rarely	O Occasionally	O Frequently	O Almost constantly
**10b.** In the last 7 days, what was the SEVERITY of your PAIN at its WORST?				
O None	O Mild	O Moderate	O Severe	O Very severe
**10c.** In the last 7 days, how much did PAIN INTERFERE with your usual or daily activities?				
O Not at all	O A little bit	O Somewhat	O Quite a bit	O Very much

Use of this document is governed by the terms of use on the first page of this document.

**11a.** In the last 7 days, how OFTEN did you have ACHING JOINTS (SUCH AS ELBOWS, KNEES, SHOULDERS)?				
O Never	O Rarely	O Occasionally	O Frequently	O Almost constantly
**11b.** In the last 7 days, what was the SEVERITY of your ACHING JOINTS (SUCH AS ELBOWS, KNEES, SHOULDERS) at their WORST?				
O None	O Mild	O Moderate	O Severe	O Very severe
**11c.** In the last 7 days, how much did ACHING JOINTS (SUCH AS ELBOWS, KNEES, SHOULDERS) INTERFERE with your usual or daily activities?				
O Not at all	O A little bit	O Somewhat	O Quite a bit	O Very much

**12a.** In the last 7 days, what was the SEVERITY of your INSOMNIA (INCLUDING DIFFICULTY FALLING ASLEEP, STAYING ASLEEP, OR WAKING UP EARLY) at its WORST?				
O None	O Mild	O Moderate	O Severe	O Very severe
**12b.** In the last 7 days, how much did INSOMNIA (INCLUDING DIFFICULTY FALLING ASLEEP, STAYING ASLEEP, OR WAKING UP EARLY) INTERFERE with your usual or daily activities?				
O Not at all	O A little bit	O Somewhat	O Quite a bit	O Very much

**13a.** In the last 7 days, what was the SEVERITY of your FATIGUE, TIREDNESS, OR LACK OF ENERGY at its WORST?				
O None	O Mild	O Moderate	O Severe	O Very severe
**13b.** In the last 7 days, how much did FATIGUE, TIREDNESS, OR LACK OF ENERGY INTERFERE with your usual or daily activities?				
O Not at all	O A little bit	O Somewhat	O Quite a bit	O Very much

Use of this document is governed by the terms of use on the first page of this document.

OTHER SYMPTOMS	
Do you have any other symptoms that you wish to report?	
O Yes	O No
**Please list any other symptoms:**	
1.	In the last 7 days, what was the SEVERITY of this symptom at its WORST?<br>O None O Mild O Moderate O Severe O Very Severe
2.	In the last 7 days, what was the SEVERITY of this symptom at its WORST?<br>O None O Mild O Moderate O Severe O Very Severe
3.	In the last 7 days, what was the SEVERITY of this symptom at its WORST?<br>O None O Mild O Moderate O Severe O Very Severe
4.	In the last 7 days, what was the SEVERITY of this symptom at its WORST?<br>O None O Mild O Moderate O Severe O Very Severe
5.	In the last 7 days, what was the SEVERITY of this symptom at its WORST?<br>O None O Mild O Moderate O Severe O Very Severe

Use of this document is governed by the terms of use on the first page of this document.

Appendix 11 European Organisation for Research and Treatment of Cancer Item List 46

ENGLISH

EORTC IL46

Patients sometimes report that they have the following symptoms or problems. Please indicate the extent to which you have experienced these symptoms or problems during the past week. Please answer by circling the number that best applies to you.

During the past week:	Not at All	A Little	Quite a Bit	Very Much
1. To what extent have you been troubled with side-effects from your treatment?	1	2	3	4

SPECIMEN

© Copyright 2019 EORTC Quality of Life Group. All rights reserved.

Use of this document is governed by the terms of use on the first page of this document.

[redacted]

Use of this document is governed by the terms of use on the first page of this document.

Appendix 13 Modified Continual Reassessment Method Escalation with Overdose Control Design Description

This appendix provides details of the design that will guide the monotherapy dose-escalation stage of this study and of its operating characteristics through simulations. All analyses were performed using the crmPack library (Sabanés-Bové et al. 2019) in the R programming environment R version 4.0.3 (2020-10-10) (R Core Team 2017).

1 RATIONALE FOR MODEL BASED DESIGN

The modified continuous reassessment method (mCRM) is a model-based approach implemented in studies designed to identify a maximum tolerated dose (MTD). The toxicity data from all enrolled evaluable participants are used to update the prior dose-toxicity curve, and to make recommendations on the next dose to be tested in the model as well as to evaluate whether the dose escalation could be stopped since (for instance) sufficient information to define the MTD has already been collected. This type of models locates the MTD efficiently and minimizes the number of participants treated at possibly pharmacological inactive dose levels.

Model-based designs have been successfully applied in many Phase I dose escalation studies (Schöffski et al. 2004; Neuenschwander et al. 2008; Le Tourneau et al. 2009; Sverdlov et al. 2014). The version used in this protocol is the one proposed by Neuenschwander et al. 2008), which introduces a tighter overdose control: in this design, the MTD is defined as the dose maximizing the posterior probability that the dose-limiting toxicity (DLT) rate, $\pi(MTD) \in [0.2, 0.3]$ while keeping the probability of overdose $P\{p(MTD) > 0.3\} < 0.25$.

2 STATISTICAL MODEL

A two-parameter logistic model will be used to fit the dose-toxicity relationship. The probability of DLT at dose d_j, $p(d_j)$ is defined as (1)

$$p(d_j) = \frac{exp(\alpha + \beta x_j)}{1 + exp(\alpha + \beta x_j)} \qquad (1)$$

where

$$x_j = \ln\left(\frac{d_j}{d^*}\right)$$

and d^* is the reference dose (in this case $d^* =$ [redacted].

The model (1) thus can be rewritten as (2):

Use of this document is governed by the terms of use on the first page of this document.

$$ln\left(\frac{p(d_j)}{1-p(d_j)}\right) = \alpha + \beta x_j \qquad (2)$$

where α and $\ln\beta$ are the parameters to be estimated and assumed to follow a bivariate normal distribution.

2.1 MODEL PRIOR

A prior based on the minimally informative bivariate normal prior (Neuenschwander et al. 2008) for the parameters of the DLT-dose response curve $(\alpha, \ln\beta)$ is constructed in order to have a weak impact on the final MTD determination.

The parameters of the minimally informative prior are listed below (3):

$$\mu = (\alpha, \ln\beta) \quad = (-3, -0.1)$$
$$\Sigma = \begin{pmatrix} \sigma_\alpha^2 & \sigma_{\alpha\ln\beta} \\ \sigma_{\alpha\ln\beta} & \sigma_{\ln\beta}^2 \end{pmatrix} = \begin{pmatrix} 3.11 & -0.55 \\ -0.55 & 0.61 \end{pmatrix} \qquad (3)$$

where μ and Σ are the parameters of the bivariate normal distribution.

The prior distribution used to determine the dose-escalation decision for this study is shown in Figure 1.

Figure 1 Minimally informative prior

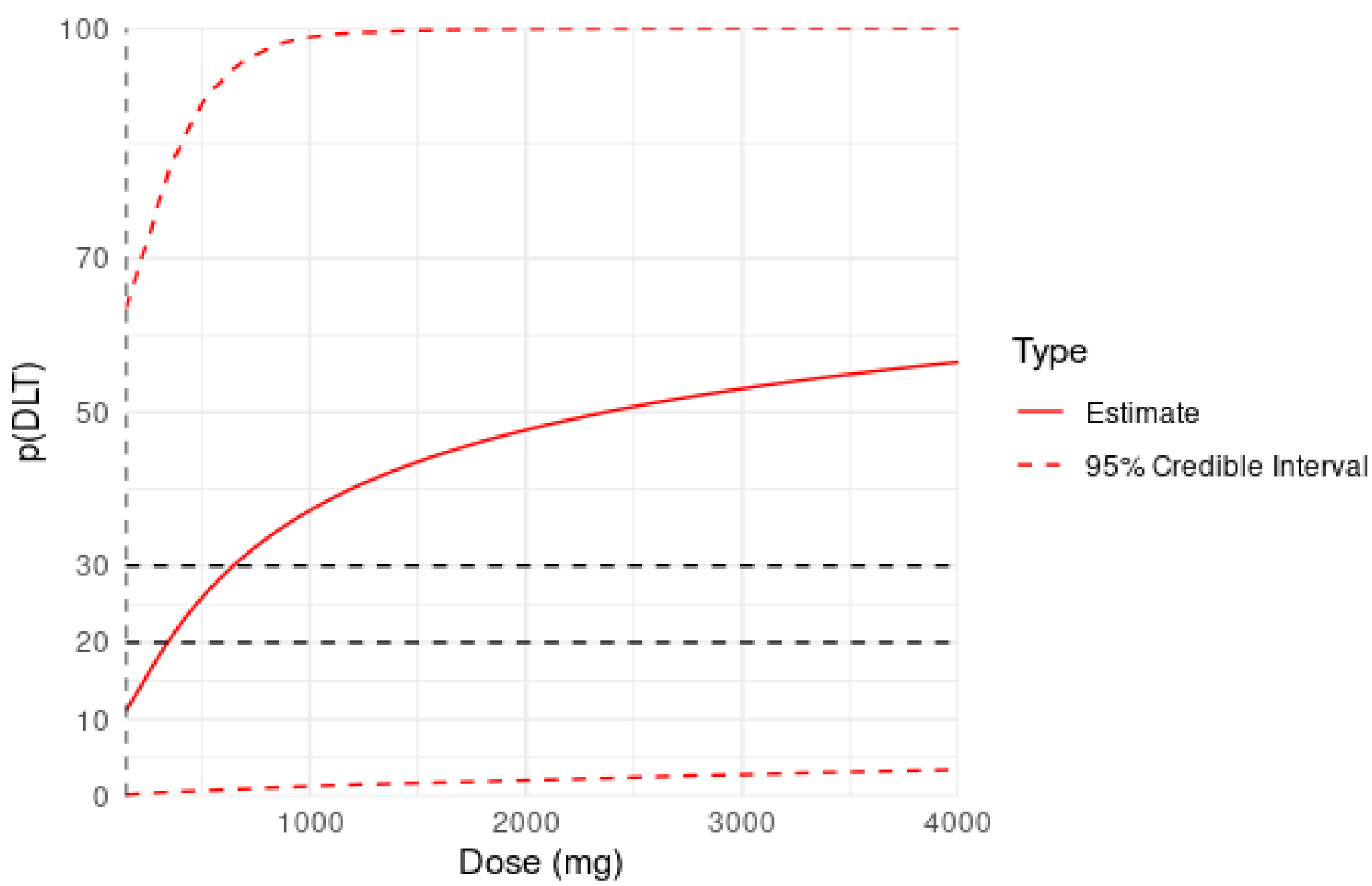

Use of this document is governed by the terms of use on the first page of this document.

2.2 MAXIMUM DOSE INCREMENTS

The following rules for selecting the maximum allowed dose increment will be applied.

- Maximum Dose Increments Relative to Dose Levels:

Maximum increments are limited to increase of 100% (maximum 2 $\times$ change) on the whole dose range.

2.3 NEXT DOSE RECOMMENDATION

When recommending the dose for a following cohort, the model will exclude doses for which $P\{p(MTD) > 0.3\} > 0.25$. Then, it will only consider doses which are admissible according to the allowable maximum increment rules and select – among these admissible doses present in the dose grid – the one with the highest probability $\pi(DLT) \in [0.2, 0.3]$.

2.4 STOPPING RULES

The algorithm will recommend ending the dose escalation if any of the following criteria applies:

- Enough information on MTD:
  - At least a minimum of 12 participants evaluated *and* at least 6 participants have been accrued near the MTD dose (where near means differing from the MTD by at most 20%) *and* the posterior probability that the MTD dose lies within the target toxicity interval is above 50% OR
- Maximum dose is safe:
  - At least 6 participants have been observed at the maximum dose or near (differing from the maximum dose by at most 20%) *and* it is at least 50% likely that the probability of a DLT for the maximum dose is below 0.2. OR
- Maximum number of participants:
  - The maximum sample size of 60 DLT-evaluable participants has been reached. Biopsy cohort participants will not be accounted in the number of participants in the stopping rule.

2.5 DOSE GRID

The following dose grid is used in the search for the optimal dose: the dose grid is composed of doses from [redacted] by steps of [redacted] and is based on the minimum tablet strength of [redacted] Rounding to the closer convenient dosage, based on tablet strength available, will be applied on the dose proposed by the model.

3 MODEL PERFORMANCE EVALUATION

To illustrate how the design will perform, different escalation scenarios are explored and results are tabulated in Table 1. Each column represents four different situations,

Use of this document is governed by the terms of use on the first page of this document.

showing which dose would the model recommend, after seeing no DLTs in previous cohorts and when 0, 1, 2, or 3 DLTs are observed in the current cohort. The evaluation is based on a cohort size = 3, NA indicates that the model would stop escalating and the trial would be halted.

Table 1 Dose Escalation Mock Runs

Dose Level	Dose [redacted]	Next dose (% increment) if no DLT	Next dose (% increment) if 1 DLT	Next dose (% increment) if 2 DLTs	Next dose (% increment) if 3 DLTs
1	[redacted]				
2					
3					
4					
5					

DLT = dose-limiting toxicity; NA = not applicable.

As can be seen from the table, in general, in presence of no DLTs, the model will suggest escalating at the max increments allowed, while in presence of one DLT the dose mainly decreased (except for a minimal increase at the starting dose). Then, with 2 or 3 DLTs the model always recommends to de-escalate or STOP. Therefore, the results show that the design will adequately adapt the recommended next dose in the presence of observed DLTs.

3.1 SIMULATION STUDY

A simulation study is conducted to evaluate the operating characteristics for the chosen design parameters (prior, reference dose, stopping rule) under various dose-toxicity scenarios. The different scenarios have been selected in order to cover a wide range of dose-toxicity possibilities and to be able to quantify the risk and benefit, should these scenarios actually occur.

3.1.1 Dose-Toxicity Scenarios

The different scenarios have been selected in order to cover a wide range of dose-toxicity possibilities and to be able to quantify the risk and benefit, should these scenarios actually occur.

As shown in Figure 2, we explored 8 scenarios. The first 7 represent decreasing levels of toxicity, while the last one considers the toxicity depicted in the prior as true toxicity. Some scenarios represent the extreme with a very low likelihood to reflect the truth, but still they are informative on how the model would eventually perform.

Use of this document is governed by the terms of use on the first page of this document.

Figure 2 True Dose-toxicity Scenarios: [Scenario number]: Dose Range Corresponding to Target Toxicity

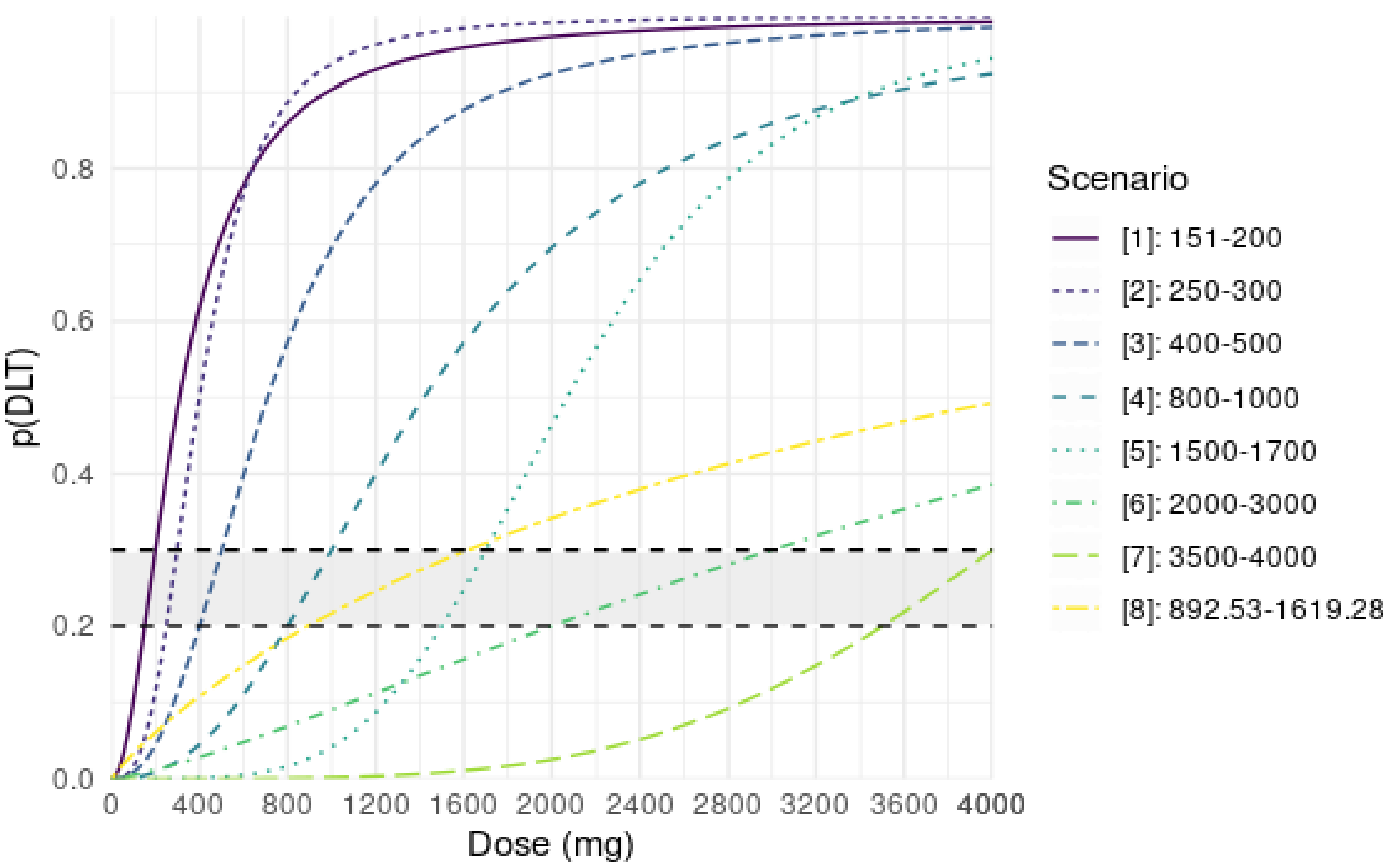

3.1.2 Simulation Results

For each of the scenarios, 1000 trials were simulated.

The design is evaluated using the following criteria: the MTD chosen, the number of participants treated at doses higher than the MTD and the total number of participants treated. For each criterion the median (with the 10th and 90th percentiles) value from the 1000 simulations is reported in Table 2.

Table 2 Summary of Simulations Results

Scenario	True Target Dose Range	Overall N of Participants	N of Participants Treated Above Target Toxicity	Proportion of DLTs in the Trials	Dose Selected as MTD
1	*151-200*	*15 (3, 45)*	*6 (0, 33)*	*33.3% (26.7%, 66.7%)*	*0 (0, 225)*
2	*250-300*	*39 (21, 51)*	*15 (3, 33)*	*26.2% (21.6%, 33.3%)*	*275 (0, 325)*
3	*400-500*	*39 (36, 54)*	*12 (3, 27)*	*24.4% (19.6%, 28.2%)*	*450 (350, 525)*
4	*800-1000*	*42 (36, 54)*	*9 (3, 24)*	*22.2% (18.5%, 25.6%)*	*875 (700, 1050)*
5	*1500-1700*	*45 (39, 54)*	*9 (3, 18)*	*20% (17.6%, 23.1%)*	*1500 (1300, 1750)*
6	*2000-3000*	*45 (27, 60)*	*6 (0, 15)*	*18.3% (8.3%, 22.2%)*	*2375 (1575, 4000)*

Use of this document is governed by the terms of use on the first page of this document.

Scenario	True Target Dose Range	Overall N of Participants	N of Participants Treated Above Target Toxicity	Proportion of DLTs in the Trials	Dose Selected as MTD
7	*3500-4000*	*24 (21, 51)*	*0 (0, 0)*	*8.3% (0%, 17.8%)*	*4000 (3272.5, 4000)*
8	*892.53-1619.28*	*45 (36, 60)*	*3 (0, 24)*	*20.4% (15%, 25.6%)*	*1100 (622.5, 1825)*

DLT = dose-limiting toxicities; MTD = maximum tolerated dose.

From these simulations, we see that the design is able to provide a reliable estimate of the MTD: the median of the doses selected as MTD is within the target toxicity dose range. To be noted, in case of very low toxicity (dose range of target toxicity between 3500 and 4000 mg), the maximum dose is selected as MTD.

This is also shown in Figure 3, where the distribution of MTDs identified in the various simulation runs is plotted against the true target toxicity range (red bars) for each scenario.

Figure 3 Results From Simulations: Box Plot of MTD From Simulations Versus True Toxicity Dose Range

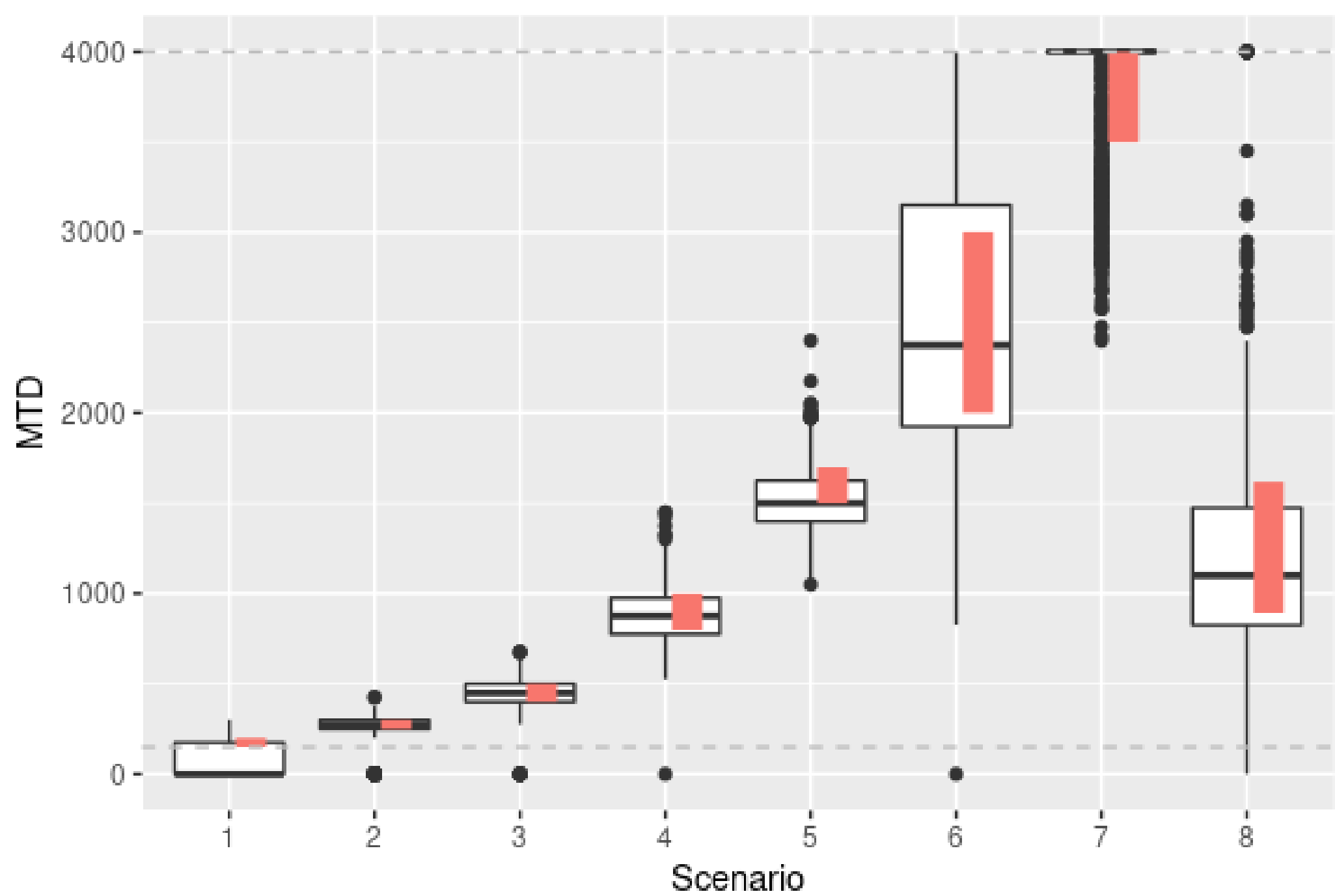

MTD = maximum tolerated dose.

In addition, the median number of participants treated over the dose toxicity interval is quite limited with 12 participants maximum.

Use of this document is governed by the terms of use on the first page of this document.

The good performances of the algorithm are also confirmed by the median number of DLTs observed in the simulations, which is $<31\%$.

Finally, also the projected sample size required is reasonable: the median number of participants required to give an MTD recommendation is maximum 45 participants across all scenarios. In case of high toxicity, the required number of treated participants is even lower.

References

Le Tourneau C, Lee JJ, and Siu LL. “Dose Escalation Methods in Phase I Cancer Clinical Trials.” Journal of the National Cancer Institute 2009;101: 708–20.

Neuenschwander B, Branson M, and Gsponer T. “Critical Aspects of the Bayesian Approach to Phase I Cancer Trials.” Statistics in Medicine, 2008;27: 2420–39.

R Core Team. 2017. R: A Language and Environment for Statistical Computing. Vienna, Austria: R Foundation for Statistical Computing. https://www.R-project.org/.

Sabanés-Bové D, Yeung WY, Palermo G, et al. Model Based Dose Escalation Designs in r with crmPack. Journal of Statistical Software. 2019;Vol. 89.

Schöffski P, Riggert S, Fumoleau P, et al.. “Phase I Trial of Intravenous Aviscumine (rViscumin) in Patients with Solid Tumors: A Study of the European Organization for Research and Treatment of Cancer New Drug Development Group.” Annals of Oncology 2004;15: 1816–24.

Sverdlov O, Wong WK, and Ryeznik Y. Adaptive Clinical Trial Designs for Phase I Cancer Studies. Statistics Surveys. 2014;Vol. 8.

Use of this document is governed by the terms of use on the first page of this document.

Appendix 14 Drugs that Should be Administered with Caution and Monitoring, Should be Administered with Time Separation or are Prohibited

Table 1 Sensitive Cytochrome P450 3A4 and Cytochrome P450 2B6 Substrates to be Used with Caution and Monitoring

Restrictions for RO7589831 dosing	Enzymes affected	Enzyme Modulation	Potentially Affected Comedications
To be used with caution and monitoring	CYP2B6	Potential induction over the duration of RO7589831 treatment and for 14 days following RO7589831 treatment reducing exposure of the comedicant drug.	Bupropion, efavirenz
To be used with caution and monitoring	CYP3A4/5	Potential induction over the duration of RO7589831 treatment and for 14 days following RO7589831 treatment affecting comedicant exposure	alfentanil, alprazolam, aprepitant, atorvastatin, avanafil, buspirone, Cannabis products colchicine, conivaptan, darifenacin, darunavir, budesonide, dasatinib, dronedarone, ebastine, eletriptan, eliglustat, eplerenone, everolimus, felodipine, ibrutinib, isavuconazole, ivabradine, lemborexant, lomitapide, lovastatin, lurasidone, maraviroc, midazolam, mobocertinib, naloxegol, nisoldipine, pimozide, quetiapine, rilpivirine, rivaroxaban, sildenafil, simvastatin, sirolimus, tacrolimus, tadalafil, ticagrelor, tolvaptan, triazolam, vardenafil, venetoclax

Table 2 P-glycoprotein Substrate Drugs to be Administered with Time Separation from RO7589831

Restrictions for RO7589831 dosing	Drug Transporter affected	Transporter Modulation	Affected Comedications
Prohibited 2 hours before and 6 hours following RO7589831 administration	P-glycoprotein	Inhibition in the gastrointestinal tract by RO7589831.<br>Not to be taken 2 hours before or 6 hours following RO7589831 administration.	Dabigatran etexilate, digoxin, edoxaban, fexofenadine

Use of this document is governed by the terms of use on the first page of this document.

Table 3 Prohibited Strong and Moderate CYP3A Inhibitors and Inducers

Restrictions for RO7589831 dosing	Enzymes affected	Enzyme Modulation	Example Comedications
Prohibited	CYP3A4/5	Inhibition of CYP3A4/5 by the comedicant affecting RO7589831 exposure.	Aprepitant, ceritinib, ciprofloxacin, clarithromycin, cobicistat, conivaptan, crizotinib, cyclosporine, diltiazem, dronedarone, erythromycin, fluconazole, fluvoxamine, grapefruit juice, idelalisib, indinavir, imatinib, isavuconazole, itraconazole, ketoconazole, nefazodone, posaconazole, telithromycin, tofisopam, troleandomycin, verapamil, voriconazole
Prohibited	CYP3A4/5	Induction of CYP3A4/5 by the comedicant affecting RO7589831 exposure.	Apalutamide, bosentan, carbamazepine, cenobamate, dabrafenib, efavirenz, enzalutamide, etravirine, ivosidenib, lorlatinib, lumacaftor, mitotane, pexidartinib, phenobarbital, phenytoin, rifampin, St. John's wort, primidone, sotorasib

Note: medications shown are not an exhaustive list of inhibitors and substrates of drug metabolizing enzymes and P-glycoprotein but are listed as examples and for guidance.

References

FDA Drug Development and Drug Interactions | Table of Substrates, Inhibitors and Inducers. Content current as of: 08/24/2022. Available at: https://www.fda.gov/drugs/drug-interactions-labeling/drug-development-and-drug-interactions-table-substrates-inhibitors-and-inducers. (last accessed: 02 February 2023)

Use of this document is governed by the terms of use on the first page of this document.

Appendix 15 Composition of High Fat Meal

Composition of a high-fat meal [1] (example):

Total Calories	800 to 1000
Calories from Protein	150
Calories from Carbohydrates	250
Calories from Fat	500-600
Percent Calories from Fat	≥ 50
An Example of a High-Fat Breakfast	Two eggs fried in butter<br>Two strips of bacon<br>Two slices of toast with butter<br>Four ounces of hash brown potatoes<br>Eight ounces of whole milk

1 50% of calories are derived from fat. Substitutions can be made to this meal if the content, volume, and viscosity are maintained.

Reference

Assessing the Effects of Food on Drugs in INDs and NDAs — Clinical Pharmacology Considerations; Draft Guidance for Industry; June 2022. Available at: https://www.fda.gov/regulatory-information/search-fda-guidance-documents/assessing-effects-food-drugs-inds-and-ndas-clinical-pharmacology-considerations. (last accessed: 15 February 2023).

Use of this document is governed by the terms of use on the first page of this document.

Appendix 16
Preexisting Autoimmune Diseases and Immune Deficiencies for Eligibility

Participants should be carefully questioned regarding their history of acquired or congenital immune deficiencies or autoimmune disease. Participants with any history of immune deficiencies or autoimmune disease listed in the table below are excluded from participating in the study. Possible exceptions to this exclusion could be participants with a medical history of such entities as atopic disease or childhood arthralgias where the clinical suspicion of autoimmune disease is low. Participants with a history of autoimmune-related hypothyroidism on a stable dose of thyroid replacement hormone may be eligible for this study. In addition, transient autoimmune manifestations of an acute infectious disease that resolved upon treatment of the infectious agent are not excluded (e.g., acute Lyme arthritis). Caution should be used when considering pembrolizumab for participants who have previously experienced a severe or life-threatening skin adverse reaction while receiving another immunostimulatory anti-cancer agent. The Medical Monitor is available to advise on any uncertainty about autoimmune exclusions.

Autoimmune Diseases and Immune Deficiencies

• *Acute disseminated encephalomyelitis*<br>• *Addison disease*<br>• *Ankylosing spondylitis*<br>• *Anti-phospholipid antibody syndrome*<br>• *Aplastic anemia*<br>• *Autoimmune hemolytic anemia*<br>• *Autoimmune hepatitis*<br>• *Autoimmune hypoparathyroidism*<br>• *Autoimmune hypophysitis*<br>• *Autoimmune myocarditis*<br>• *Autoimmune oophoritis*<br>• *Autoimmune orchitis*<br>• *Autoimmune thrombocytopenic purpura*<br>• *Behçet disease*<br>• *Bullous pemphigoid*<br>• *Chronic fatigue syndrome*<br>• *Chronic inflammatory demyelinating polyneuropathy*<br>• *Churg-Strauss syndrome*<br>• *Crohn disease*	• *Dermatomyositis*<br>• *Diabetes mellitus, type 1*<br>• *Dysautonomia*<br>• *Epidermolysis bullosa acquisita*<br>• *Gestational pemphigoid*<br>• *Giant cell arteritis*<br>• *Goodpasture syndrome*<br>• *Granulomatosis with polyangiitis*<br>• *Graves disease*<br>• *Guillain-Barré syndrome*<br>• *Hashimoto disease*<br>• *IgA nephropathy*<br>• *Inflammatory bowel disease*<br>• *Interstitial cystitis*<br>• *Kawasaki disease*<br>• *Lambert-Eaton myasthenia syndrome*<br>• *Lupus erythematosus*<br>• *Lyme disease, chronic*<br>• *Meniere syndrome*<br>• *Mooren ulcer*<br>• *Morphea*<br>• *Multiple sclerosis*<br>• *Myasthenia gravis*	• *Neuromyotonia*<br>• *Opsoclonus myoclonus syndrome*<br>• *Optic neuritis*<br>• *Ord thyroiditis*<br>• *Pemphigus*<br>• *Pernicious anemia*<br>• *Polyarteritis nodosa*<br>• *Polyarthritis*<br>• *Polyglandular autoimmune syndrome*<br>• *Primary biliary cholangitis*<br>• *Psoriasis*<br>• *Reiter syndrome*<br>• *Rheumatoid arthritis*<br>• *Sarcoidosis*<br>• *Scleroderma*<br>• *Sjögren syndrome*<br>• *Stiff-Person syndrome*<br>• *Takayasu arteritis*<br>• *Ulcerative colitis*<br>• *Vitiligo*<br>• *Vogt-Koyanagi-Harada disease*

Use of this document is governed by the terms of use on the first page of this document.

Appendix 17 *Anaphylaxis Precautions*

These guidelines are intended as a reference and should not supersede pertinent local or institutional standard operating procedures.

REQUIRED EQUIPMENT AND MEDICATION

The following equipment and medication are needed in the event of a suspected anaphylactic reaction during study treatment administration in a clinical setting:

1. *Monitoring devices: ECG monitor, blood pressure monitor, oxygen saturation monitor, and thermometer*
2. *Oxygen*
3. *Epinephrine for intramuscular (preferred route), subcutaneous, intravenous, or endotracheal administration in accordance with institutional guidelines*
4. *Antihistamines*
5. *Corticosteroids*
6. *Intravenous infusion solutions, tubing, catheters, and tape*

PROCEDURES

In the event of a suspected anaphylactic reaction during study treatment administration, the following procedures should be performed:

1. *Stop the study treatment administration, if possible.*
2. *Call for additional medical assistance.*
3. *Maintain an adequate airway.*
4. *Ensure that appropriate monitoring is in place, with continuous ECG and pulse oximetry monitoring if possible.*
5. *Administer antihistamines, epinephrine, or other medications and IV fluids as required by participant status and as directed by the physician in charge.*
6. *Continue to observe the participant and document observations.*

Use of this document is governed by the terms of use on the first page of this document.

Appendix 18 Abbreviations

Abbreviation	Definition
**4α–HC**	4α–hydroxycholesterol
**4β–HC**	4β–hydroxycholesterol
**AE**	adverse event
**AESI**	adverse event of special interest
**ALP**	alkaline phosphatase
**ALT**	alanine aminotransferase
**ANC**	absolute neutrophil count
**aPTT**	activated partial thromboplastin time
**AST**	aspartate aminotransferase
**ATD**	anticipated therapeutic dose
**AUC**	area under the curve
**BID**	twice-daily
BRCA	*Breast Cancer Gene*
**C**	Cycle
**C1D1**	Cycle 1 Day 1
**CFR**	Code of Federal Regulations
**CL**	clearance
**CL/F**	apparent clearance
**C_{max}**	maximum concentration
**CNS**	central nervous system
**COA**	clinical outcome assessments
**COVID-19**	coronavirus disease 2019
**CR**	complete response
**CRC**	colorectal adenocarcinoma
**CRO**	contract research organization
**CSF**	colony-stimulating factors
**CSR**	clinical study report
**CT**	computed tomography
**CTCAE**	Common Terminology Criteria for Adverse Events
**ctDNA**	circulating tumor DNA
**CYP**	cytochrome P540s
**CYP3A**	cytochrome P450 3A4
**CYP2B6**	cytochrome P450 2B6
**D**	Day
**DCR**	disease control rate

Use of this document is governed by the terms of use on the first page of this document.

**DDR**	DNA damage response
**DDT**	Dose Decision Team
**DEP**	dose escalation plan
**DLT**	dose-limiting toxicity
**dMMR**	deficient mismatch repair
**DNA**	Deoxyribonucleic acid
**DOR**	duration of response
**DSB**	double-strand breaks
**EC**	Ethics Committee
**ECG**	electrocardiogram
**eCOA**	electronic clinical outcome assessment
**ECOG**	Eastern Cooperative Oncology Group
**eCRF**	electronic case report form
**EDC**	electronic data capture
**EEA**	European Economic Area
**EiH**	entry-into-human
**EORTC-IL46**	European Organisation for Research and Treatment of Cancer Item List 46
**EORTC QLQ-F17**	European Organisation for Research and Treatment of Cancer Quality-of-life Questionnaire-Function 17
**EoS**	end of study
**EoT**	end-of-treatment
**E.U.**	European Union
**EWOC**	escalation with overdose control
**FDA**	Food and Drug Administration
**FDG**	fluoro deoxy glucose
**[^{18}F]-FDG-PET**	fluoro deoxy glucose positron emission tomography
**FE**	food effect
**FFPE**	formaldehyde-fixed paraffin-embedded
**FSH**	follicle-stimulating hormone
**GCP**	Good Clinical Practice
**HBV**	hepatitis C virus
**HCV**	hepatitis C virus
**HDL**	high-density lipoproteins
**HIPAA**	Health Insurance Portability and Accountability Act of 1996
**HIV**	human immunodeficiency virus
**HR**	heart rate
**HRT**	hormonal replacement therapy

Use of this document is governed by the terms of use on the first page of this document.

**IB**	Investigator's Brochure
**ICF**	Informed Consent Form
**ICH**	International Council for Harmonisation
**IEC**	Independent Ethics Committee
**IHC**	immunohistochemistry
**IMC**	Internal Monitoring Committee
**IMP**	investigational medicinal product
**IND**	Investigational New Drug (application)
**INR**	international normalized ratio
**IRB**	Institutional Review Board
**IRC**	Independent Review Committee
**IwRS**	interactive web response system
**IxRS**	interactive (voice/web) response system
**LDH**	lactate dehydrogenase
**LDL**	low-density lipoproteins
**LH**	luteinizing hormone
**LPLV**	last participant, last visit
**LPLO**	last participant, last observation
**LTFU**	long-term follow-up
**mCRM**	modified continual reassessment method
**MMR**	mismatch repair
**MOA**	mode of action
**MRI**	magnetic resonance imaging
**MSI**	microsatellite instability
**MSS**	microsatellite stable
**MTD**	maximum tolerated dose
**MUGA**	Multiple-gated acquisition
**NCI**	National Cancer Institute
**NGS**	next-generation sequencing
**NOAEL**	no-observed-adverse-effect level
**NYHA**	New York Heart Association
**ORR**	objective response rate
**OS**	overall survival
**OTC**	over-the-counter
**P-gp**	P-glycoprotein
**PARP**	poly adenosine diphosphate-ribose polymerase
**PBMC**	peripheral blood mononuclear cell
**PD**	pharmacodynamic

Use of this document is governed by the terms of use on the first page of this document.

**PD-1**	programmed death-1
**PD-L1**	programmed death-ligand 1
**PET**	positron emission tomography
**PFS**	progression-free survival
**PK**	pharmacokinetic
**po**	orally
**POCBP**	participant of child bearing potential
**PoM**	Proof of Mechanism
**PONCBP**	participant of non-childbearing potential
**PR**	partial response
**PRO**	patient-reported outcome (also refers to participants)
**PT**	prothrombin time
[redacted]	[redacted]
**QD**	once daily
**QRS**	QRS complex
**QT**	QT interval
**QTc**	QT corrected for heart rate
**QTcF**	QT corrected for heart rate using the Fridericia's correction factor
**RBR**	Research Biosample Repository
**RECIST**	Response Evaluation Criteria in Solid Tumors
**RNA**	ribonucleic acid
**RP2D**	Recommended Phase 2 dose
**RR**	RR interval
**SAD**	single-ascending dose
**SAE**	serious adverse event
**SAP**	Statistical Analysis Plan
**SARS CoV 2**	severe acute respiratory syndrome coronavirus 2
**SC**	subcutaneous
**SD**	single dose
**SoA**	schedule of activities
**SoC**	standard of care
**SOP**	standard operating procedure
**TC**	total cholesterol
**TE**	target engagement
[redacted]	[redacted]
**TILs**	T lymphocytes
**T_{max}**	time of maximum concentration observed
**TQT**	Thorough QT

Use of this document is governed by the terms of use on the first page of this document.

**TSH**	thyroid-stimulating hormone
**ULN**	upper limit of normal
**U.S.**	United States
**VAS**	visual analog scale
**V/F**	apparent volume of distribution
**WES**	whole exome sequencing
**WGS**	whole genome sequencing
**WRN**	Werner syndrome

Use of this document is governed by the terms of use on the first page of this document.

Signature Page for Protocol - BP44474 - WRN COVALENT INH - V3 - Published
System identifier: RIM-CLIN-537530

Approval Task	[redacted]<br>Scientific content approver<br>18-Jun-2024 14:28:55 GMT+0000

Use of this document is governed by the terms of use on the first page of this document.

BP44474 study protocol: Summary of amendments to Version 3

Changes from Version 1 (not approved) to Version 2 (approved in US/Canada/UK)

Protocol BP44474 has been amended [redacted] Changes to the protocol, along with a rationale for each change, are summarized below.

- Window of safety follow-up visit has been modified in the Schedule of Assessments (SoA) Table 1, in the synopsis, Section 4.4 and Section 8.10.4.
- Clarifications added regarding the collection of tumor biopsies that should be conducted only if considered safe for the study participants. This includes changes in footnote of Table 3 and Table 4, and Section 5.1.
- [redacted]. This includes changes in Section 4.1.2 and Section 9.2.
- The dose-limiting toxicity (DLT) definition criteria have been updated for those related to non-hematological laboratories values in Section 4.1.6.2.
- The inclusion criterion 9 (Section 5.1) referring to adequate renal function has been modified.
- The exclusion criterion 9 (Section 5.2) referring to cardiac function has been modified.
- Dose modification guidance (Section 6.6) has been amended to provide specific dose reduction levels and maximum numbers of allowed dose reductions.
- Clarification on plans to continue to provide study drug after completion of the study in Section 6.7.
- Clarification on treatment modifications/discontinuation in case of treatment related CTCAE Grade 3 or 4 toxicity. This includes changes in Section 8.3.9 and cross-reference in Section 6.6 and Section 7.1.

Additional minor changes have been made to improve clarity (i.e., clarification is added on the rationale for tumor biopsy (Section 4.2.3) to further explain the rationale for mandatory biopsies in the backfill cohorts) and consistency (i.e., prohibited medication corrected (Section 6.5.3) to align with eligibility criteria; SoA Table 5 was corrected to align with protocol text).

Use of this document is governed by the terms of use on the first page of this document.

Changes from Version 2 (not approved in EU) to Version 2/EEA 2 (approved in the EU)

Protocol BP44474 has been amended [redacted] Changes to the protocol, along with a rationale for each change, are summarized below.

- Inclusion criterion 5 (Section 5.1) has been amended to indicate that only patients who have exhausted all alternative standard of care options may be considered for this study.
- Exclusion criterion 5 (Section 5.2) has been amended to indicate that patients cannot be enrolled if they had a major episode of infection within 2 weeks from start of study treatment requiring antibiotics given systemically, and not only if administered intravenously.
- Rules for dose reductions that apply to [redacted] of the study have been added in Section 6.6.
- The management of adverse events section (Section 8.3.9) has been amended to provide additional guidance on the management of hematological and non hematological toxicities, including dose reduction recommendations based on the type and grade of toxicity as well as the requirement to permanently discontinue study treatment in case of drug-induced liver injury (DILI) and recurrent non hematological Grade 3 toxicity.

Additional minor changes have been made to improve clarity and consistency.

Changes from Version 2 to Version 3

With this amendment the details on the design and conduct of [redacted]

- Pembrolizumab was specified as the combination treatment and replaced PD-1/PD-L1 therapy throughout the document (Section 1, Section 3, Section 4.1.5, Section 6, Section 6.4.1 (including in the study title).
- The Schematic of study design has been [redacted]
- SoA sampling for RO7589831 in combination with pembrolizumab treatment added (Section 1.3).
- [redacted]
- [redacted]

Use of this document is governed by the terms of use on the first page of this document.

- [redacted]
- Communication strategy in Section 4.1.6.5, Administrative Structure in Section 4.1.7, [redacted]
- The rationale and benefit-risk for the combination treatment of RO7589831 with pembrolizumab was included (Section 2.3 and 4.2.8, respectively).
- Inclusion criteria were updated to include information regarding [redacted]
- Additional Exclusion criteria [redacted]
- [redacted]
- Medications given with precaution due to effects related to pembrolizumab were included (Section 1.1 and Section 6.5.2.3).
- [redacted]
- [redacted]
- [redacted]
- [redacted]
- [redacted]
- [redacted]
- [redacted]
- [redacted]
- Pembrolizumab was added to the list of investigational medicinal product and non-investigational medicinal product designations for use in European Economic Area and United Kingdom (Appendix 6).

Additional changes to the protocol are summarized below:

- The protocol has been aligned with the IB v2.0 where the genotoxicity potential risk, which

Use of this document is governed by the terms of use on the first page of this document.

was previously added based on the positive results from an in vitro Ames assay with short-lived metabolite (RO7589830) and a positive in vitro micronucleus test with RO7589831, has been removed, supported by the negative in vivo genotoxicity results. The corresponding changes in the protocol are reflected in the Benefit -Risk assessment Section 2.3 and in the contraception requirements in the eligibility criteria Section 5.1.

- Small modifications to the PK collection timepoints have been made in order to better define the absorption phase of the PK profile. These changes have no impact on the number of samples collected or blood volume collected (Section 1.3).
- The RBR specific samples are no longer required to be collected because the sample derivates can be obtained from existing samples (Section 1.3 and 8.8.2).
- The collection of urine (PK) samples on Day 1 has been included to enable renal clearance assessment (Section 1.3). The Rationale for Urine Collection and Analysis has also been added (Section 4.2.10 and Section 4.2.5).
- [redacted] dosing is now explicitly defined as an option throughout the protocol. Minor modifications have been made to the SoAs and considerations for potential comedications that are sensitive P-GP substrates with narrow therapeutic index have been added in case [redacted] dosing would be implemented (Section 1.3 and throughout the protocol as applicable).
- Hospitalization after Cycle 1 Day 1 dose has been made optional to be implemented at Investigator discretion to allow completion of the visit and sampling scheduled on Cycle 1 Day 1 (Section 1.3).
- Clarification that the ICF can be signed by the patient before the 28 days screening window (Section 1.3).
- To minimize the loss of relevant pre-dose and post dose PK samples and assessment on Cycle 1 Day 8 visit day, the following were added in Section 1.3: recommendation to the site to contact the patient on the day before the clinic visit as reminder to ensure the morning dose of RO7589831 is held on the clinic visit Day; possibility to reschedule the Cycle 1 Day 8 visit and all associated assessments the day after in case patient does not hold morning dose until clinic visit day as scheduled.
- Clarification that the tumor assessment visit where radiographic disease progression has been documented can be used as the treatment discontinuation visit (Section 1.3).
- The dose limiting toxicities criteria Section 4.1.6.3 has been updated to add a clarification to the dose-limiting toxicities criteria exception for pancreatitis and removal of Alopecia Grade 3 (because not applicable per CTCAE criteria).

Use of this document is governed by the terms of use on the first page of this document.

- Dose escalation decision criteria for Part I was updated to allow patients to undergo intra-patient dose escalation if the patient experiences disease progression during study treatment. This option is only available if the Investigator deems the patient might benefit from higher dose of RO7589831 and the benefit/risk of this option has been thoroughly discussed with the patient (Section 4.1.1).
- Clarifications were added to the discontinuation for study treatment for Part I [redacted] (Section 7.1).
- Rationale for biomarker assessment was updated to include information regarding the assessment of MSI tumor status as required by local regulations (Section 4.2.2, Section 8.6) (Note: Change not included in Version 3/EEA2).
- [redacted]
- The proposed maximum daily RO7589831 dose has been raised from [redacted] (Section 4.3) and the modified continual reassessment method escalation with overdose control design description was updated to account for the change in Max dose (Appendix 12).
- Inclusion criteria have been updated to 1) clarify that for Backfill cohorts the same tumor lesion should be biopsied if possible to avoid introduction of heterogeneity related to site of metastasis; 2) change the calculated creatinine clearance threshold from 60 to 50 mL/min (for rationale please refer to separate memo) (Section 5.1).
- Exclusion criteria have been updated to 1) clarify that patients with pulmonary embolism within 6 months from enrollment in the study may be eligible if event is considered resolved in the opinion of the Investigator and per local guidelines; 2) clarify that patients with Lynch syndrome with multiple tumors are not excluded (Section 5.2).
- Clarification on the administration of RO7589831 and allowed time window has been added in Section 6.4.
- Clarification on the continuation of tumor assessments in participants who discontinue study treatment in the absence of radiographic disease progression has been added (Section 8.1.1).
- The time point for the first on-treatment [18F]-FDG PET scan has been modified to decouple the scan window from the on-treatment biopsy time point and keep the window more consistent allowing for better cross-cohort comparisons (Section 8.1.2).
- Information regarding the management of adverse events was updated (Section 8.3.9).

Use of this document is governed by the terms of use on the first page of this document.

- Information regarding collection and reporting of medical device adverse events was added as needed per local regulations (Appendix 1, Appendix 2).
- The list of clinical laboratory test was updated (Appendix 4).
- Contraceptive and barrier methods guidance was updated (Appendix 5).

Additional minor changes have been made to improve clarity and consistency.

Use of this document is governed by the terms of use on the first page of this document.
