# Supplementary material for: Werner helicase inhibitor for advanced microsatellite instability solid tumors: a phase 1 trial
Source: Nat Med. 2026 Sep 29;32(11):4068–78. doi: 10.1038/s41591-026-04657-3 (PMC13768166; doi:10.1038/s41591-026-04657-3)
Supplement: Supplementary file 2 [file 41591_2026_4657_MOESM2_ESM.pdf]

nature portfolio

Corresponding author(s): Timothy A Yap

Last updated by author(s): Jul 30, 2026

Reporting Summary

Nature Portfolio wishes to improve the reproducibility of the work that we publish. This form provides structure for consistency and transparency in reporting. For further information on Nature Portfolio policies, see our Editorial Policies and the Editorial Policy Checklist.

Statistics

For all statistical analyses, confirm that the following items are present in the figure legend, table legend, main text, or Methods section.

n/a	Confirmed	
☐	☒	The exact sample size (*n*) for each experimental group/condition, given as a discrete number and unit of measurement
☒	☐	A statement on whether measurements were taken from distinct samples or whether the same sample was measured repeatedly
☐	☒	The statistical test(s) used AND whether they are one- or two-sided<br>*Only common tests should be described solely by name; describe more complex techniques in the Methods section.*
☒	☐	A description of all covariates tested
☒	☐	A description of any assumptions or corrections, such as tests of normality and adjustment for multiple comparisons
☐	☒	A full description of the statistical parameters including central tendency (e.g. means) or other basic estimates (e.g. regression coefficient) AND variation (e.g. standard deviation) or associated estimates of uncertainty (e.g. confidence intervals)
☒	☐	For null hypothesis testing, the test statistic (e.g. *F*, *t*, *r*) with confidence intervals, effect sizes, degrees of freedom and *P* value noted<br>*Give P values as exact values whenever suitable.*
☒	☐	For Bayesian analysis, information on the choice of priors and Markov chain Monte Carlo settings
☒	☐	For hierarchical and complex designs, identification of the appropriate level for tests and full reporting of outcomes
☒	☐	Estimates of effect sizes (e.g. Cohen's *d*, Pearson's *r*), indicating how they were calculated

Our web collection on statistics for biologists contains articles on many of the points above.

Software and code

Policy information about availability of computer code

Data collection	The Medidata RAVE electronic data capture system was used to capture, manage, clean and report the trial data. No custom computer code or algorithms were used.
Data analysis	Tables were generated using SAS (v9.4). Figures were generated using R (v4.5.1). Population pharmacokinetic modelling was performed using Monolix Suite (2024R1). No custom computer code or algorithms were used.

For manuscripts utilizing custom algorithms or software that are central to the research but not yet described in published literature, software must be made available to editors and reviewers. We strongly encourage code deposition in a community repository (e.g. GitHub). See the Nature Portfolio guidelines for submitting code & software for further information.

Data

Policy information about availability of data

All manuscripts must include a data availability statement. This statement should provide the following information, where applicable:

- Accession codes, unique identifiers, or web links for publicly available datasets
- A description of any restrictions on data availability
- For clinical datasets or third party data, please ensure that the statement adheres to our policy

For eligible studies, qualified researchers may request access to individual patient-level clinical data via the cross-industry request site https://vivli.org. Full details of Roche’s approach to sharing individual patient-level clinical data via the Vivli site can be found at https://vivli.org/ourmember/roche. In summary, access requests

are assessed by an Independent Review Panel managed by Vivli. The panel considers the scientific merit of each application and compliance with patient consent and data-sharing policies, with the timeline for request approval (usually 2–5 months) influenced by the number of data contributors, the number of studies and the requestor's availability to respond to comments. Once approved, the data is anonymized and made available for a minimum of 12 months. For up-to-date details on Roche's Global Policy on the Sharing of Clinical Information and how to request access to related clinical study documents, see here: https://go.roche.com/data_sharing. Anonymized records for individual patients across more than one data source external to Roche cannot, and should not, be linked due to a potential increase in risk of patient re-identification.

Research involving human participants, their data, or biological material

Policy information about studies with human participants or human data. See also policy information about sex, gender (identity/presentation), and sexual orientation and race, ethnicity and racism.

Reporting on sex and gender	Sex was based on self-reported demographics obtained at screening. No sex-based analyses were performed, as this was a Phase I study.
Reporting on race, ethnicity, or other socially relevant groupings	Race was based on self-reported demographics obtained at screening. No race-based analyses were performed, as this was a Phase I study.
Population characteristics	Among 88 patients with MSI/dMMR advanced solid tumors (determined as part of the routine diagnostic work-up in a certified local laboratory), median age was 60 years (range: 27–86), 50 (56.8%) were male and 77 (87.5%) were white. Sixty-nine (78.4%) patients had confirmed MSI/dMMR tumors by central laboratory methods. Overall, 57 (64.8%) patients had advanced CRC, eight (9.1%) had advanced endometrial cancer, and 23 (26.1%) had other tumor types. The median number of prior lines of therapy was three (range: 1–12). Eighty-four (95.5%) patients had received ≥1 prior ICI.
Recruitment	Between January 25, 2024 (first patient in) and June 30, 2025 (last patient in), 88 patients with MSI and/or dMMR advanced solid tumors (determined as part of the routine diagnostic work-up in a certified local laboratory) were enrolled at 17 centers in eight countries.
Ethics oversight	NCT06004245 was conducted in accordance with the International Council for Harmonisation guideline for Good Clinical Practice and the principles of the Declaration of Helsinki. The protocol was approved by the institutional review board/ethics committee (IRB/EC) at each participating center. All patients provided written informed consent and were not compensated.

Note that full information on the approval of the study protocol must also be provided in the manuscript.

Field-specific reporting

Please select the one below that is the best fit for your research. If you are not sure, read the appropriate sections before making your selection.

☒ Life sciences ☐ Behavioural & social sciences ☐ Ecological, evolutionary & environmental sciences

For a reference copy of the document with all sections, see nature.com/documents/nr-reporting-summary-flat.pdf

Life sciences study design

All studies must disclose on these points even when the disclosure is negative.

Sample size	The sample sizes for the dose escalation and backfill cohorts were based on the occurrence of DLTs and the number of dose levels required to determine the MTD and/or RP2D. A maximum of 60 patients was needed to provide a safe and accurate estimation of the MTD. A maximum of 40 patients were required to characterize the dose/pharmacodynamic relationship.
Data exclusions	Safety analyses were performed in all patients who received ≥1 dose of study treatment. Pharmacokinetic analyses were carried out using data from all patients who received ≥1 dose of study treatment and had data from ≥1 post-dose sample. PK data points inconsistent with the individuals PK profile were considered outliers and removed manually. Five PK profiles on C1D1 and 8 PK profiles on C1D8 lacked sufficient data points for PK parameter determination via non-compartmental analysis. Efficacy analyses were performed in all patients with MSI tumors (by central testing and/or preexisting local testing) who had a valid baseline tumor assessment and ≥1 post-baseline tumor assessment.
Replication	Not applicable. NCT06004245 was a first-in-human, Phase I study.
Randomization	Not applicable. NCT06004245 was a non-randomized, Phase I study.
Blinding	Not applicable. NCT06004245 was an open-label, Phase I study.

Reporting for specific materials, systems and methods

We require information from authors about some types of materials, experimental systems and methods used in many studies. Here, indicate whether each material, system or method listed is relevant to your study. If you are not sure if a list item applies to your research, read the appropriate section before selecting a response.

Materials & experimental systems

n/a	Involved in the study
☐	☒ Antibodies
☐	☒ Eukaryotic cell lines
☒	☐ Palaeontology and archaeology
☒	☐ Animals and other organisms
☐	☒ Clinical data
☒	☐ Dual use research of concern
☒	☐ Plants

Methods

n/a	Involved in the study
☒	☐ ChIP-seq
☒	☐ Flow cytometry
☒	☐ MRI-based neuroimaging

Antibodies

Antibodies used	VENTANA MMR RxDx Panel is a set of qualitative immunohistochemistry tests intended for laboratory use in assessing mismatch repair (MMR) proteins (MLH1, PMS2, MSH2 and MSH6) in formalin-fixed, paraffin-embedded (FFPE) tissue specimens by light microscopy. The VENTANA MMR RxDx panel includes the following primary antibody kits: VENTANA anti-MLH1 (M1) Mouse Monoclonal Primary Antibody (package insert 790-5091), VENTANA anti-PMS2 (A16-4) Mouse Monoclonal Primary Antibody (package insert 790-5094), VENTANA anti-MSH2 (G219-1129) Mouse Monoclonal Primary Antibody (package insert 790-5093), and VENTANA anti-MSH6 (SP93) Rabbit Monoclonal Primary Antibody (package insert 790-5092).
Validation	The qualitative immunohistochemistry tests used in the VENTANA MMR RxDx Panel were developed and validated by VENTANA.

Eukaryotic cell lines

Policy information about cell lines and Sex and Gender in Research

Cell line source(s)	HCT116 is an adherent cell line isolated from the colon of an adult male patient with colorectal cancer. HCT116 cells were sourced from American Type Culture Collection (ATCC). See https://www.atcc.org/products/ccl-247 for full information.
Authentication	Authentication was carried out by the supplier (American Type Culture Collection; ATCC). See https://www.atcc.org/products/ccl-247 for full information.
Mycoplasma contamination	Mycoplasma contamination was not detected by the supplier (American Type Culture Collection; ATCC). See https://www.atcc.org/products/ccl-247 for full information.
Commonly misidentified lines (See ICLAC register)	Not applicable.

Clinical data

Policy information about clinical studies

All manuscripts should comply with the ICMJE guidelines for publication of clinical research and a completed CONSORT checklist must be included with all submissions.

Clinical trial registration	NCT06004245
Study protocol	The study protocol was included in the Nature Medicine submission package to inform the peer review and has been included in the Supplementary Information.
Data collection	Between January 25, 2024 (first patient in) and June 30, 2025 (last patient in), 88 patients with MSI and/or dMMR advanced solid tumors (determined as part of the routine diagnostic work-up in a certified local laboratory) were enrolled at 17 centers in eight countries.
Outcomes	Primary objectives were to evaluate safety, including determination of the maximum tolerated dose (MTD) via assessment of dose-limiting toxicities, and identify the recommended Phase 2 dose and schedule for RO7589831 monotherapy. Secondary objectives were to evaluate pharmacokinetics and anti-tumor activity (endpoints: disease control rate, objective response rate, duration of response, progression-free survival, and overall survival). Exploratory objectives included evaluation of pharmacodynamic effects and biomarkers associated with tumor response.

Plants

Seed stocks	*Report on the source of all seed stocks or other plant material used. If applicable, state the seed stock centre and catalogue number. If plant specimens were collected from the field, describe the collection location, date and sampling procedures.*
Novel plant genotypes	*Describe the methods by which all novel plant genotypes were produced. This includes those generated by transgenic approaches, gene editing, chemical/radiation-based mutagenesis and hybridization. For transgenic lines, describe the transformation method, the number of independent lines analyzed and the generation upon which experiments were performed. For gene-edited lines, describe the editor used, the endogenous sequence targeted for editing, the targeting guide RNA sequence (if applicable) and how the editor was applied.*
Authentication	*Describe any authentication procedures for each seed stock used or novel genotype generated. Describe any experiments used to assess the effect of a mutation and, where applicable, how potential secondary effects (e.g. second site T-DNA insertions, mosiacism, off-target gene editing) were examined.*
